# A multicenter phase 2 trial of camrelizumab plus famitinib for women with recurrent or metastatic cervical squamous cell carcinoma

**DOI:** 10.1038/s41467-022-35133-4

**Published:** 2022-12-08

**Authors:** Lingfang Xia, Qi Zhou, Yunong Gao, Wenjing Hu, Ge Lou, Hong Sun, Jianqing Zhu, Jin Shu, Xianfeng Zhou, Rong Sun, Xiaohua Wu

**Affiliations:** 1 Department of Gynecological Oncology, Fudan University Shanghai Cancer Center, Shanghai, China; 2 Department of Gynaecology, Affiliated Tumor Hospital of Chongqing University, Chongqing, China; 3 Department of Gynaecology, Beijing Cancer Hospital, Beijing, China; 4 Department of Oncology, Nanjing Drum Tower Hospital, Nanjing, China; 5 Department of Gynaecology, Affiliated Tumor Hospital of Harbin Medical University, Harbin, China; 6 Department of Gynecologic Oncology, Obstetrics & Gynecology Hospital of Fudan University, Shanghai, China; 7 Department of Gynecological Oncology, Cancer Hospital of the University of Chinese Academy of Sciences (Zhejiang Cancer Hospital), Hangzhou, China; 8 Department of Clinical Development, Jiangsu Hengrui Pharmaceuticals Co., Ltd., Shanghai, China

**Keywords:** Cervical cancer, Drug development

## Abstract

This phase 2 study assesses the efficacy and safety of camrelizumab (an anti-PD-1 antibody) plus famitinib (anti-angiogenic agent) in women with pretreated recurrent or metastatic cervical cancer (ClinicalTrials.gov NCT03827837). Patients with histologically or cytologically confirmed cervical squamous cell carcinoma experiencing relapse or progression during or after 1–2 lines of systemic therapy for recurrent or metastatic disease are enrolled. Eligible patients receive camrelizumab 200 mg intravenously on day 1 of each 3-week cycle plus famitinib 20 mg orally once daily. The primary endpoint is the objective response rate. Secondary endpoints are duration of response, disease control rate, time to response, progression-free survival, overall survival, and safety. The trial has met pre-specified endpoint. Thirty-three patients are enrolled; median follow-up lasts for 13.6 months (interquartile range: 10.0–23.6). Objective responses are observed in 13 (39.4%, 95% confidence interval [CI]: 22.9–57.9) patients; the 12-month duration of response rate is 74.1% (95% CI: 39.1–90.9). Median progression-free survival is 10.3 months (95% CI: 3.5–not reached) and the 12-month overall survival rate is 77.7% (95% CI: 58.9–88.7). All patients experience treatment-related adverse events; grade ≥3 events occur in 26 (78.8%) patients. Treatment-related serious adverse events and deaths are observed in 9 (27.3%) and 2 (6.1%) patients, respectively. Camrelizumab plus famitinib shows promising antitumor activity with a manageable and tolerable safety profile in patients with pretreated recurrent or metastatic cervical squamous cell carcinoma. This combination may represent a treatment option for this population.

## Abstract (web summary)

Patients with recurrent or metastatic cervical cancer have limited therapeutic options. Here the authors report efficacy and safety of camrelizumab (anti-PD-1 antibody) in combination with famitinib (anti-angiogenic agent) in pre-treated recurrent or metastatic squamous cervical cancer.

## Introduction

Cervical cancer is the fourth leading cause of cancer-related deaths among women worldwide^1^. Increasing rates of human papillomavirus vaccination in recent years has led to a decline in the incidence and mortality of cervical cancer. However, these improvements have not been observed in vulnerable populations without access to health care, particularly in developing countries and regions. In 2020, approximately 604,127 new cases of cervical cancer were diagnosed globally, resulting in 341,831 cancer deaths^1^.

For patients with recurrent or metastatic cervical cancer which is not amenable to radical local excision or regional radiation, the preferred first-line treatment is platinum-containing combination chemotherapy plus bevacizumab^2^. However, most patients with disease recurrence or metastasis inevitably develop disease progression.

Based on the findings of the KEYNOTE-158 study, which showed a modest objective response rate of 14.3%, the Food and Drug Administration approved the use of pembrolizumab on June 12, 2018 for patients with PD-L1 expressing (combined positive score ≥1) recurrent or metastatic cervical cancer having disease progression on or after chemotherapy^3^. Monotherapy with cemiplimab and nivolumab also demonstrated efficacy in previously treated recurrent or metastatic cervical cancer, but the effectiveness remained minimal (objective response rate: 16.4% and 26.3%, respectively)^4,5^. Tisotumab vedotin, a tissue factor-directed antibody and microtubule inhibitor conjugate, showed better antitumor efficacy than certain single immunotherapy agents and was granted accelerated approval for patients with recurrent or metastatic cervical cancer with disease progression on or after chemotherapy; however, the objective response (24%) and duration of response (8.3 months) remained limited^6^. In recent years, combination of anti-PD-1/PD-L1 drugs with other agents has been proposed as a promising strategy for extending the efficacy of PD-1/PD-L1 antibodies. Anti-PD-1 drugs in combination with anti-CTLA-4 drugs (balstilimab plus zalifrelimab or nivolumab plus ipilimumab) have shown notable efficacy in patients with pretreated cervical cancer^7,8^. In the LIO-1 trial, nivolumab plus lucitanib led to an objective response in 31.8% of patients with pretreated metastatic or recurrent cervical cancer^9^. The KEYNOTE-826 trial showed that pembrolizumab in combination with chemotherapy and with or without bevacizumab significantly improved progression-free and overall survival than placebo in patients with persistent, recurrent, or metastatic cervical cancer who had not received systemic chemotherapy^10^. This supported the potential use of anti-PD-1/PD-L1 drugs in combination with other agents in the later-line setting of cervical cancer.

Vascular endothelial growth factor (VEGF) is a key mediator of angiogenesis^11^. This prompted researchers to focus on this pathway as a therapeutic target, with the objective of inducing vascular regression; this could induce a lack of oxygen, glucose, and other essential metabolites in tumors, thereby inhibiting tumor growth^12^. The anti-VEGF antibody, bevacizumab, is a widely prescribed antiangiogenic drug; bevacizumab-containing regimens have been recommended by guidelines for the treatment of recurrent or metastatic cervical cancer, both in first and later line settings (objective response: 10.9%)^13–15^. Blockade of the VEGF pathway by VEGF or VEGF receptor inhibition improves immunotherapeutic responsiveness, partially by normalizing tumor vasculature; this facilitates infiltration of immune effector cells into tumors, and reprograms the immunosuppressive tumor microenvironment to an immunosupportive one^12^. Reports suggest that the combination of immunotherapy and antiangiogenic therapy could improve the effectiveness of immunotherapy and mitigate the risk of immune-related adverse events^12,16–18^.

Camrelizumab is a humanized, high-affinity, selective IgG4-κ monoclonal antibody against PD-1, that exerts antitumor activity across a wide range of tumors^19–24^. Famitinib, a structural analog of sunitinib, is a multi-targeted receptor tyrosine kinase inhibitor targeting the stem-cell factor receptor, VEGF receptor 2/3, platelet-derived growth factor receptor β, FMS-like tyrosine kinase-1/3 receptor, and proto-oncogene tyrosine-protein kinase receptor^25,26^. Based on these findings, we conducted a phase 2 basket study to assess the efficacy and safety of camrelizumab plus famitinib. Here we report the results of camrelizumab combined with famitinib in patients with recurrent or metastatic cervical squamous cell carcinoma, who had relapsed or progressed during or after 1–2 lines of systemic therapy.

## Results

### Patients

Between April 4, 2019 and June 8, 2020, 33 patients with recurrent or metastatic cervical squamous cell carcinoma were enrolled. All patients received camrelizumab plus famitinib treatment and were included in efficacy analysis. The baseline characteristics are presented in Table 1.

**Table 1:** Demographics and baseline characteristics

	Patients (*N* = 33)
Age, years, median (IQR)	50 (43–55)
ECOG performance status	
0	5 (15.2)
1	28 (84.8)
No. of organs of metastases	
1	9 (27.3)
2	11 (33.3)
>2	13 (39.4)
Histological grade	
G2	10 (30.3)
G3	4 (12.1)
Unknown	5 (15.2)
Undetermined	14 (42.4)
FIGO stage at initial diagnosis^a^	
I	12 (36.4)
II	14 (42.4)
III	5 (15.2)
IV	1 (3.0)
Unknown	1 (3.0)
PD-L1 expression	
Positive	10 (30.3)
Negative	9 (27.3)
Undetermined	14 (42.4)
No. of prior systemic therapy lines	
1	21 (63.6)^b^
2	9 (27.3)
≥3	3 (9.1)
Prior systemic therapy	
Platinum	33 (100)
Paclitaxel	32 (97.0)
Bevacizumab	9 (27.3)
Prior radiation	32 (97.0)
Prior hysterectomy	22 (66.7)
Time from initial cancer diagnosis to study enrollment, months, median (IQR)	22.4 (12.8–38.5)

Data are presented as *n* (%), unless otherwise specified. *IQR* interquartile range, *ECOG* Eastern Cooperative Oncology Group, *FIGO* International Federation of Gynecology and Obstetrics.

^a^Patients were classified according to the 2009 FIGO staging for carcinoma of the cervix.

^b^Five patients who received neoadjuvant or adjuvant therapy and developed disease relapse and progression within one year after surgery or six months after radiotherapy were included.

As of data cutoff on June 8, 2021, the median duration of follow-up was 13.6 months (interquartile range [IQR]: 10.0–23.6). A total of 22 (66.7%) patients discontinued treatment owing to the following reasons: disease progression (13 patients, 39.4%), adverse events (3 patients, 9.1%), patient decision (2 patients, 6.1%), investigator decision (1 patient, 3.0%), clinical progression (1 patient, 3.0%), consent withdrawal (1 patient, 3.0%), and death (1 patient, 3.0%; vaginal hemorrhage). Twelve (36.4%) patients received post-discontinuation systemic anti-tumor therapy; this included 6 (18.2%) patients who received chemotherapy and 4 (12.1%) patients who received immunotherapy.

### Antitumor activity

**Fig. 1:**
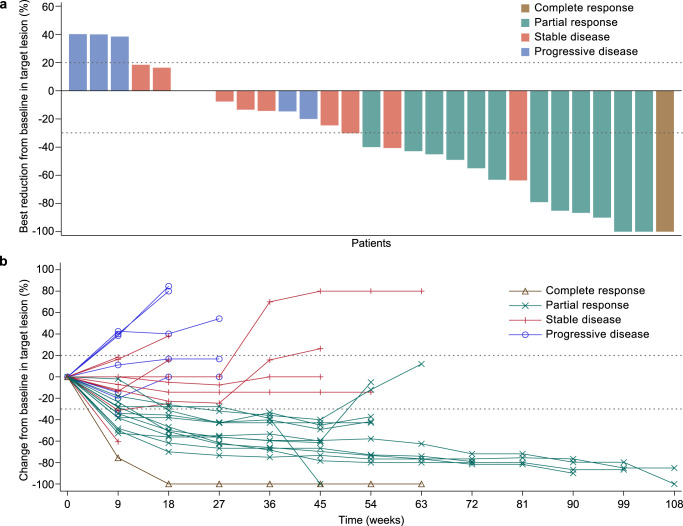
Tumor responses after camrelizumab plus famitinib treatment. **a** Waterfall plot demonstrating the best percentage change from baseline in target tumor lesion size. Each bar represents a patient. The dashes indicate a 20% increase or 30% reduction. **b** Spider plot presenting the percentage change from baseline in target tumor lesion size over time. The waterfall and spider diagrams showed the tumor responses in 29 patients who had at least one post-baseline scan of target lesion. In one patient, the target lesion had not been measured at post-baseline but a new lesion appeared during treatment. This patient was determined to have progressive disease and was not included in the waterfall and spider diagrams.

**Fig. 2:**
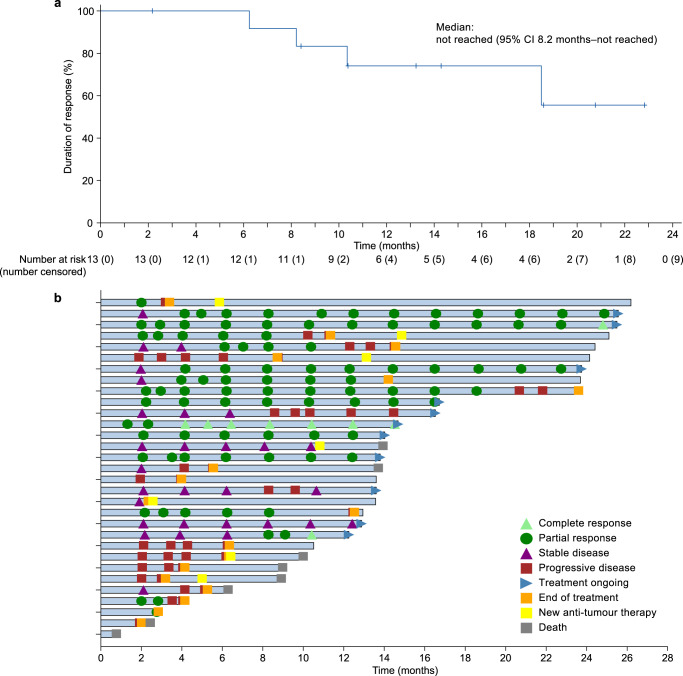
Duration of response. **a** Kaplan–Meier curve of duration of response. Data are for the 13 patients with confirmed objective responses. **b** Swimming plot demonstrating the treatment exposure and duration of tumor response in the full analysis population. Two patients who did not have tumor evaluation results at post-baseline and did not die during the study treatment were not included in the swimming plot. The length of the bars corresponds with time from treatment initiation to the last tumor assessment or death.

Among the first 22 patients who were enrolled at stage 1, confirmed responses were reported in 7 patients. Therefore, the threshold was reached for the objective response rate at stage 1; 11 patients were additionally recruited at stage 2. In the full analysis set, 13 (39.4%, 95% confidence interval [CI]: 22.9–57.9) of the 33 patients achieved confirmed objective responses, including 1 (3.0%) patient with complete response and 12 (36.4%) patients with partial responses (Table 2). Disease control was achieved in 23 (69.7%, 95% CI: 51.3–84.4) of the 33 patients. Targeted lesions were reduced relative to baseline in 22 (75.9%) of 29 patients who had at least one post-baseline scan of target lesion. The tumor response in each patient is presented in a waterfall plot (Fig. 1a) and spider plot (Fig. 1b). Among the 13 patients with confirmed objective responses, the median time to response was 2.2 months (IQR: 2.1–4.1). Tumor response was ongoing in 9 (69.2%) of the 13 patients, and the Kaplan–Meier estimated median duration of response was not reached (95% CI: 8.2–not reached; Fig. 2a). The duration of response probabilities at 6, 9, and 12 months were 100.0% (95% CI: 100.0–100.0), 83.3% (95% CI: 48.2–95.6), and 74.1% (95% CI: 39.1–90.9), respectively. The swimming plots demonstrating the durability of response are shown in Fig. 2b.

**Table 2:** Tumor responses

	Patients (*N* = 33)
Best overall response	
Complete response	1 (3.0)
Partial response	12 (36.4)
Stable disease	10 (30.3)
Progressive disease^a^	7 (21.2)
Not evaluable^b^	3 (9.1)
Objective response	39.4% (22.9-57.9)
Disease control	69.7% (51.3–84.4)
Time to response, months	2.2 (2.1-4.1)

Data are presented as *n* (%), % (95% confidence intervals), or medians (interquartile range). Responses were evaluated by investigators according to the Response Evaluation Criteria in Solid Tumors version 1.1.

^a^The target lesion of one patient had not been measured at post-baseline but a new lesion appeared during treatment, so the overall response was determined as progressive disease.

^b^“Not evaluable” indicates patients who did not undergo post-baseline tumor response evaluation.

**Fig. 3:**
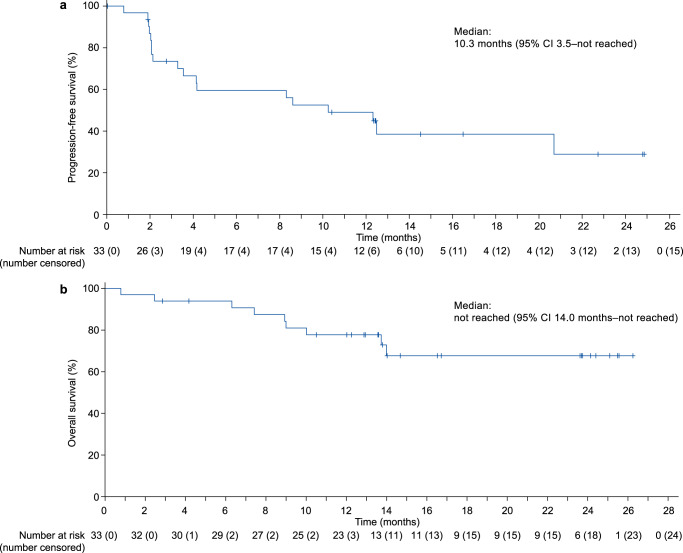
Kaplan–Meier curves of progression-free survival and overall survival. **a** Progression-free survival. **b** Overall survival. Data are for the full analysis population (*N* = 33).

As of data cutoff, 18 (54.5%) patients developed disease progression or died, and the estimated median progression-free survival was 10.3 months (95% CI: 3.5–not reached; Fig. 3a). The probabilities of progression-free survival at 6, 9, and 12 months were 59.5% (95% CI: 39.8–74.7), 52.5% (95% CI: 33.3–68.6), and 49.0% (95% CI: 30.1–65.4), respectively. As only 9 (27.3%) patients had died, the overall survival data were immature (Fig. 3b). The Kaplan–Meier estimated overall survival probabilities at 6, 9, and 12 months were 93.9% (95% CI: 77.9–98.4), 84.2% (95% CI: 66.1–93.1), and 77.7% (95% CI: 58.9–88.7), respectively.

Efficacy outcomes were analyzed by PD-L1 expression levels. Among the 19 patients who underwent PD-L1 expression detection, 10 and 9 were PD-L1 positive and negative, respectively. Camrelizumab plus famitinib conferred an objective response rate of 40.0% (4/10, 95% CI: 12.2–73.8) in those with positive PD-L1 expression and 33.3% (3/9, 95% CI: 7.5–70.1) in those with negative PD-L1 expression; the corresponding disease control rates were 80.0% (8/10, 95% CI: 44.4–97.5) and 55.6% (5/9, 95% CI: 21.2–86.3), respectively.

### Safety

The median number of treatment cycles (3-week cycle) of camrelizumab was 16.0 (IQR: 6.0–21.0) and the relative dose intensity was 90.0% (IQR: 80.0–100.0). The median duration of exposure to famitinib was 42.6 weeks (IQR: 17.3–63.1), with a relative dose intensity of 77.8% (IQR: 60.7–94.8).

All 33 patients received at least 1 dose of study drugs and underwent at least 1 post-baseline assessment; all patients were therefore included for safety analysis. In total, 33 (100%) patients experienced treatment-related adverse events (Table 3); the most common treatment-related adverse events of any grade were anemia (*n* = 22, 66.7%), decreased platelet count (*n* = 22, 66.7%), and decreased white blood cell count (*n* = 20, 60.6%). Treatment-related adverse events of grade 3 or higher were reported in 26 (78.8%) patients; the events occurring in more than 10% of patients included anemia (*n* = 9, 27.3%), decreased neutrophil count (*n* = 7, 21.2%), decreased white blood cell count (*n* = 5, 15.2%), hypertension (*n* = 5, 15.2%), and hypertriglyceridemia (*n* = 4, 12.1%). Reactive capillary endothelial proliferation, an adverse event commonly associated with camrelizumab, was reported in 3 (9.1%) patients receiving camrelizumab plus famitinib; all of these events were of grade 1 severity.

**Table 3:** Treatment-related adverse events

	Patients (*N* = 33)				
	Any grade	Grade 3–5	Grade 3	Grade 4	Grade 5
Any	33 (100.0)	26 (78.8)	23 (69.7)	1 (3.0)	2 (6.1)
Anemia	22 (66.7)	9 (27.3)	9 (27.3)	0	0
Platelet count decreased	22 (66.7)	2 (6.1)	1 (3.0)	1 (3.0)	0
White blood cell count decreased	20 (60.6)	5 (15.2)	5 (15.2)	0	0
Neutrophil count decreased	19 (57.6)	7 (21.2)	7 (21.2)	0	0
Proteinuria	18 (54.5)	1 (3.0)	1 (3.0)	0	0
Diarrhea	18 (54.5)	0	0	0	0
Hypothyroidism	18 (54.5)	0	0	0	0
Urinary tract infection	17 (51.5)	0	0	0	0
Hypertension	16 (48.5)	5 (15.2)	5 (15.2)	0	0
Hypertriglyceridemia	16 (48.5)	4 (12.1)	4 (12.1)	0	0
Gamma-glutamyltransferase increased	16 (48.5)	3 (9.1)	3 (9.1)	0	0
Alanine aminotransferase increased	16 (48.5)	2 (6.1)	2 (6.1)	0	0
Hand-foot syndrome	15 (45.5)	2 (6.1)	2 (6.1)	0	0
Hypercholesterolemia	15 (45.5)	0	0	0	0
Aspartate aminotransferase increased	14 (42.4)	1 (3.0)	1 (3.0)	0	0
Hyperglycemia	13 (39.4)	1 (3.0)	1 (3.0)	0	0
Hyperuricemia	13 (39.4)	0	0	0	0
Blood alkaline phosphatase increased	11 (33.3)	0	0	0	0
Weight decreased	10 (30.3)	0	0	0	0
Decreased appetite	7 (21.2)	0	0	0	0
Asthenia	7 (21.2)	0	0	0	0
Vaginal hemorrhage	3 (9.1)	2 (6.1)	1 (3.0)	0	1 (3.0)
Blood pressure increased	2 (6.1)	2 (6.1)	2 (6.1)	0	0

Data are presented as *n* (%). Any grade treatment-related adverse events reported in at least 20% of patients and grade 3–5 treatment-related adverse events reported in at least 5% of patients are listed.

Treatment-related serious adverse events were reported in 9 (27.3%) patients (Supplementary Table 1); all of these events (except for death) either improved or resolved. Deaths due to treatment-related adverse events were reported in 2 (6.1%) patients; 1 each died of vaginal hemorrhage and unknown cause, respectively.

Ten (30.3%) patients experienced treatment-related adverse events leading to interruption of camrelizumab (Supplementary Table 2). Treatment-related adverse events led to famitinib interruption in 25 (75.8%) patients and famitinib dose reduction in 17 (51.5%) patients (Supplementary Table 3 and Supplementary Table 4). Three (9.1%) patients discontinued camrelizumab due to treatment-related adverse events that included urogenital fistula, rash, vaginal hemorrhage, and mouth ulceration (*n* = 1 for each, 3.0%); 5 (15.2%) patients discontinued famitinib owing to treatment-related adverse events including urogenital fistula (*n* = 2, 6.1%), rash, vaginal hemorrhage, mouth ulceration, and embolism (*n* = 1 for each, 3.0%).

Immune-related adverse events of any grade were reported in 12 (36.4%) patients, with hypothyroidism (*n* = 8, 24.2%) being the most common (Supplementary Table 5).

## Discussion

This phase 2 trial was designed to assess the efficacy and safety of camrelizumab plus famitinib for the treatment of patients with cervical squamous cell carcinoma who had experienced relapse or progression during or after one to two lines of systemic therapy. This study met its primary endpoint; camrelizumab combined with famitinib achieved a substantial number of objective responses (39.4%, 95% CI: 22.9–57.9) in previously treated recurrent or metastatic cervical squamous cell carcinoma. Reductions in tumor size were observed in 75.9% of patients who had at least one post-baseline scan of target lesion. The response was durable, with a probability of response duration at 12 months of 74.1% (95% CI: 39.1–90.9) and a progression-free survival of 10.3 months (95% CI: 3.5–not reached). Although the median overall survival was immature, the probability of 12-month overall survival was as high as 77.7% (95% CI: 58.9–88.7). The adverse events of this combination treatment were manageable and tolerable. Given that recurrent or metastatic cervical cancer is associated with limited overall survival of less than one year, the findings from this study indicated that the combination of camrelizumab with famitinib could offer clinically meaningful benefits in this population.

The antibody-drug conjugate, tisotumab vedotin, has demonstrated a limited response rate (24%) and duration (8.3 months, 95% CI: 4.2–not reached)^6^; immuno-monotherapy agents including pembrolizumab, nivolumab, and cemiplimab have also shown unsatisfactory response rates^3–5^. Camrelizumab combined with famitinib showed numerical superiority over pembrolizumab, nivolumab, cemiplimab, and tisotumab vedotin monotherapy in terms of the objective response rate (39.4% vs. 14.3–26.3%), progression-free survival (10.3 months vs. 2.1–5.1 months), and probability of overall survival at 12-month (77.7% vs. 41.4–77.5%)^3–6^. The objective response rate of the camrelizumab plus famitinib combination exceeded that of other reported combinations (nivolumab plus lucitanib [objective response: 31.8%], balstilimab plus zalifrelimab [27.4%], nivolumab plus ipilimumab [23.1%], and atezolizumab plus bevacizumab [0%])^7–9,17^. An investigator-initiated study demonstrated that camrelizumab combined with apatinib (another tyrosine kinase inhibitor) offered a response rate of 56%, a 12-month duration of response rate of 66.8%, and a 9-month overall survival rate of 69.2% in advanced cervical cancer^16^. Apatinib is a small-molecule antiangiogenic agent that selectively inhibits VEGFR-2 and also mildly inhibits c-Kit and c-Src tyrosine kinases^27^. With a longer median follow-up period than camrelizumab plus apatinib study (13.6 vs. 11.3 months), the compelling antitumor activity of camrelizumab plus famitinib was further supported by an encouraging response duration, which may have translated into longer overall survival (12-month duration of response rate, 74.1%; 9-month overall survival rate, 84.2%). This indicated that the combination of camrelizumab and famitinib retained the advantage of durable tumor response offered by immunotherapy. Notably, cross-trial comparisons should be interpreted with caution, as different patient populations are included. However, our data suggested that camrelizumab combined with famitinib may be an efficacious treatment option for recurrent or metastatic cervical cancer.

There is currently no definite consensus on the relationship between PD-L1 expression and treatment efficacy^3,16^. In our study, objective responses were observed in both PD-L1 positive and negative patients, with similar proportions in the two PD-L1 subcohorts (40.0% vs. 33.3%). It is worth noting that the sample size of patients who underwent PD-L1 measurements in this study was limited; the results generated were therefore insufficient for drawing clear inferences regarding the correlation of PD-L1 expression level with efficacy. Further investigations in larger cohorts may provide additional evidence for the role of PD-L1 expression on efficacy.

The combination of camrelizumab and famitinib demonstrated a tolerable and manageable safety profile in patients with recurrent or metastatic cervical squamous cell carcinoma. Reactive capillary endothelial proliferation is a common skin toxicity associated with camrelizumab^28^; hand-foot syndrome, hypertension, and proteinuria are likely to be associated with famitinib^25^. The incidence of reactive capillary endothelial proliferation in this study was substantially reduced compared with that of camrelizumab monotherapy; this finding was in line with those from other studies that combined camrelizumab with anti-angiogenic agents^27,29–31^. This indicates that the camrelizumab-regulated immune response may destroy the dynamic balance between pro- and anti-angiogenic factors. No new or unexpected adverse events were identified in relation to camrelizumab or famitinib.

In a phase 3 study on lenvatinib in combination with pembrolizumab for advanced endometrial cancer, adverse events of grade 3 and higher were observed in 88.9% of patients who received the combination; the median time to the first dose reduction of lenvatinib was 1.9 months (range: 0.1–22.8), and lenvatinib dose reduction owing to adverse events was needed in 66.5% of patients^32^. Prospective studies on lenvatinib have demonstrated no noticeable reduction in toxicities with lower starting doses^33,34^. In our study, the incidence of grade 3 and higher adverse events (84.8%), median time to the first dose reduction of famitinib (median: 2.8 months [range: 1.1–13.9]), and incidence of famitinib dose reduction owing to adverse events (51.5%) were all similar to those observed in the study on lenvatinib combined with pembrolizumab. Therefore, the daily starting dose of famitinib for the follow-up study remains at 20 mg; toxicities will be closely monitored.

Two treatment-related deaths were reported in this study. One patient died due to vaginal hemorrhage, who were enrolled in this study owing to recurrent disease of the vaginal stump and experienced grade 5 vaginal hemorrhage after receipt of one course of camrelizumab plus famitinib. Other adverse events in this patient were all of grade 1 or 2 severity. As vaginal hemorrhage is a common clinical manifestation of advanced cervical cancer, timely intervention using formalin-soaked packs, interventional radiology procedures, or radiotherapy techniques for palliation of vaginal bleeding may improve outcomes of these patients. The patient who died from unknown cause had discontinued the study due to disease progression after treatment with 3 cycles of the study drug; she died 13 days later. As no definite causes were identified for the death, the relationship between the death and study treatment was considered to be undetermined. In the follow-up study, it will be necessary to monitor for the occurrence of adverse events such as female genital tract fistulas, urogenital fistulas, and vaginal hemorrhage, as these complications are related to advanced cervical cancer and the study drugs.

This phase 2 study is limited by its small sample size and the nonrandomized single-arm nature. The associations between baseline characteristics and tumor response were not evaluated in the study due to the limited sample size. In addition, the proportion of positive PD-L1 expression is higher in squamous cell carcinoma than in adenocarcinoma of the cervix, and high expression of PD-L1 is commonly associated with better efficacy of anti-PD-1/PD-L1 treatment^35–37^; the promising efficacy observed with the combination treatment in this study may be partly due to the fact that the tumors in all enrolled patients had a squamous cell carcinoma component. An ongoing randomized, open-label, 3-arm phase 2 trial including squamous cell carcinoma, adenocarcinoma, and adenosquamous cell carcinoma of the cervix is assessing the efficacy and safety of camrelizumab combined with famitinib (NCT 04680988).

In conclusion, the results from this study suggest that camrelizumab combined with famitinib offers durable antitumor activity with a manageable safety profile in patients with previously treated relapsed or metastatic cervical squamous cell carcinoma. Further phase 3 randomized trials on this combination strategy are warranted.

## Methods

### Study design and participants

This study was a multicenter, open-label, single-arm, phase 2 trial on camrelizumab plus famitinib as monotherapy or combination therapy in patients with advanced genitourinary or gynecological cancers. The study was registered at ClinicalTrials.gov (NCT03827837) on February 4, 2019. Here we report the data from cohort 5, which was conducted across 7 study centers in China (Supplementary Table 6). The first patient was enrolled on April 4, 2019, and the last patient on June 8, 2020. The protocol of this study is available as Supplementary Note 1 in the Supplementary Information file.

Eligible patients were aged between 18 and 75 years; had histologically or cytologically confirmed cervical squamous cell carcinoma; had experienced relapse or progression during or after 1–2 lines of systemic therapy for recurrent or metastatic disease (excluding radiotherapy sensitized chemotherapy); had at least one measurable lesion according to the Response Evaluation Criteria in Solid Tumors (RECIST) version 1.1; had an Eastern Cooperative Oncology Group performance status of 0 or 1; had a life expectancy of at least 12 weeks; and had adequate hematological, hepatic, and renal function. If the disease relapsed and progressed within 1 year after standard surgery or 6 months after radiotherapy, neoadjuvant or adjuvant therapy (excluding radiotherapy sensitized chemotherapy) was considered as first-line systemic treatment. A key protocol amendment was made based on the following update: “relapsed or progressed during or after at least 1 line of systemic therapy for recurrent or metastatic disease” (November 30, 2018; version 1.1) to “relapsed or progressed during or after 1–2 lines of systemic therapy for recurrent or metastatic disease” (amendment date August 6, 2019; version 2.0).

The key exclusion criteria were as follows: any active autoimmune disease; history of autoimmune disease, immunosuppressive medication intake, or systemic corticosteroid administration within 2 weeks before study drug administration; history of untreated central nervous system metastases, coagulation abnormalities, bleeding event of grade ≥2 according to the Common Terminology Criteria for Adverse Events (CTCAE) version 4.03 within 4 weeks before study drug administration; history of treatment with PD-1 or PD-L1 antagonists or famitinib; and known additional malignancies within the last 5 years.

The study was conducted in accordance with the Declaration of Helsinki and Good Clinical Practice guidelines. The protocol and all amendments were approved by the Ethics Committee of each study center (Supplementary Table 6). All patients provided written informed consent. Patients were offered minor compensation for their participation in the study (that is, travel costs).

### Procedures

Patients received camrelizumab 200 mg (intravenously) every 3 weeks on day 1 of each 3-week cycle plus famitinib 20 mg (orally) once daily, according to the tolerability of this combination treatment in previous studies^29–31^. Treatment was continued until confirmed disease progression, unacceptable toxicity, patient decision or withdrawal of consent, investigator decision, death, loss to follow-up, or 2 years of treatment, whichever occurred first. Interruption of camrelizumab was permitted for up to 12 consecutive weeks, but dose reduction was not allowed. Interruption and dose reduction were both allowed for famitinib.

### Assessments

Tumor responses were assessed by the investigator at baseline and every 3 cycles (9 weeks) after the initiation of treatment using computed tomography or magnetic resonance imaging according to RECIST version 1.1. Complete and partial responses were confirmed by subsequent repeat imaging at least 4 weeks after initial response assessment. Disease progression had to be confirmed by imaging examination 4–6 weeks later, and survival was assessed every two months until death.

Safety evaluation included assessment for adverse events, vital signs, 12-lead electrocardiograms, and laboratory tests, which were evaluated on days 1 and 7 of the first cycle and on day 1 of every 3-week cycle thereafter. Adverse events were monitored throughout treatment and for up to 30 days after the last dose, and graded according to the CTCAE version 4.03. Only treatment-related serious adverse events were recorded in cases where subsequent anti-cancer treatment was initiated. PD-L1 expression in tumor samples was assessed at a central laboratory (Guangzhou KingMed Center for Clinical Laboratory Co., Ltd., Guangzhou, China) using a PD-L1 immunohistochemistry kit (22C3 pharmDx test, Dako, Carpinteria, CA, USA) according to the manufacturer’s instructions, and was characterized by the combined positive score. PD-L1 positivity was defined as a combined positive score of at least 1.

### Outcomes

The primary endpoint was the objective response rate, defined as the proportion of patients with a best overall response of complete or partial response. Secondary endpoints included duration of response (the time from the first complete or partial response to death or progression, whichever occurred first), disease control rate (the proportion of patients with a best overall response of complete response, partial response, or stable disease), time to response (duration between the first dose and first documented tumor response), progression-free survival (duration between the first dose and the first documented radiographic progression or death from any cause), overall survival (duration between the first dose to death from any cause), probability of 12-month overall survival, and safety.

Pharmacokinetics (PK) of camrelizumab combined with famitinib and anti-camrelizumab antibodies (ADA) in subjects receiving combination therapy were also pre-specified secondary endpoints for the first 12 subjects enrolled in Cohorts 1–5. Since only 2 of the first 12 subjects were from the cervical cancer cohort presented here (cohort 5), the PK and ADA of all the 12 subjects will be analyzed and published separately.

Pre-specified exploratory endpoints included the correlations between study treatment efficacy and PD-L1 expression or other biomarkers.

### Statistical analysis

An adaptive two-stage design was adopted for patient enrollment^38^. An objective response rate of 15% was assumed as ineffective, 25% was assumed to represent a low response rate, and 35% was assumed to represent a high response rate. Planned sample sizes of 53 and 33 patients were estimated to provide 70% and 80% power for demonstrating low and high response rates, respectively, with a two-sided α level of 0.1. At stage 1, 22 patients were enrolled. Recruitment of stage 2 was planned as follows: if there were ≤2 responders in stage 1, further recruitment would be terminated; in the event of 3–6 responders, an additional 31 patients would be recruited at stage 2 to achieve a total of 53 patients. In the event of ≥7 responders, an additional 11 patients would be recruited at stage 2 to achieve a total of 33 patients.

Efficacy was assessed in the full-analysis set, which included all patients who received at least one dose of the study drugs. The safety analysis set included all patients who received at least one dose of the study drugs and had at least one post-baseline safety assessment. The objective response rate and disease control rate and their 95% CIs were calculated using the Clopper–Pearson method. Time-to-event endpoints were estimated using the Kaplan–Meier method and their 95% CIs were calculated with the Brookmeyer Crowley method. The 95% CIs for survival probabilities were calculated using log–log transformation based on normal approximation followed by back transformation. Data were collected using HRTAU EDC (version 2.8.4). All statistical analyses were performed using SAS (version 9.4 or higher) software.

### Reporting summary

Further information on research design is available in the Nature Portfolio Reporting Summary linked to this article.

## Supplementary information

Supplementary Information

Reporting Summary

## Data Availability

Individual de-identified participant data that underlie the results reported in this article is subject to controlled access. Data may be requested after the product and indication has been approved by major health authorities and 24 months after completion of all the arms of the NCT03827837 trial. Qualified researchers should submit a proposal to the corresponding author (wu.xh@fudan.edu.cn) outlining the reasons for requiring the data. The leading clinical site and sponsor will check whether the request is subject to any intellectual property or confidentiality obligations. Use of data must also comply with the requirements of Human Genetics Resources Administration of China. A signed data access agreement with the sponsor is required before accessing shared data. The study protocol is provided as Supplementary Note 1 in the Supplementary Information. The remaining data are available within the Article and Supplementary Information.
