# Supplementary material for: A multicenter phase 2 trial of camrelizumab plus famitinib for women with recurrent or metastatic cervical squamous cell carcinoma
Source: Nat Commun. 2022 Dec 8;13:7581. doi: 10.1038/s41467-022-35133-4 (PMC9732039; doi:10.1038/s41467-022-35133-4)
Supplement: Supplementary file 1 — Supplementary Information [file 41467_2022_35133_MOESM1_ESM.pdf]

**Supplementary Information**

Supplement to: Lingfang Xia, et al. A multicenter phase 2 trial of camrelizumab plus famitinib for women with recurrent or metastatic cervical squamous cell carcinoma

**Table of contents**

Supplementary Table 1. Treatment-related serious adverse events ..............................2
Supplementary Table 2. Treatment-related adverse events leading to interruption of camrelizumab ..........................................................................................................3
Supplementary Table 3. Treatment-related adverse events leading to interruption of famitinib ...............................................................................................................4
Supplementary Table 4. Treatment-related adverse events leading to dose reduction of famitinib ...............................................................................................................6
Supplementary Table 5. Immune-related adverse events .............................................7
Supplementary Table 6. Participating sites ..................................................................8
Supplementary Note 1. Study protocol .........................................................................9

**Supplementary Table 1. Treatment-related serious adverse events**

| | All patients (N=33) | |
|---|---|---|
| | **Any grade** | **Grade 3 or higher** |
| Any | 9 (27.3) | 8 (24.2) |
| Vaginal hemorrhage | 3 (9.1) | 2 (6.1) |
| Female genital tract fistula | 1 (3.0) | 1 (3.0) |
| White blood cell count decreased | 1 (3.0) | 1 (3.0) |
| Platelet count decreased | 1 (3.0) | 1 (3.0) |
| Anemia | 2 (6.1) | 2 (6.1) |
| Hyperglycemia | 1 (3.0) | 1 (3.0) |
| Conjunctivitis | 1 (3.0) | 0 |
| Rash | 1 (3.0) | 1 (3.0) |
| Death | 1 (3.0) | 1 (3.0) |
| Urogenital fistula | 1 (3.0) | 1 (3.0) |
| Mouth ulceration | 1 (3.0) | 1 (3.0) |

Data are n (%).

**Supplementary Table 2. Treatment-related adverse events leading to interruption of camrelizumab**

| | All patients (N=33) | |
|---|---|---|
| | **Any grade** | **Grade 3 or higher** |
| Any | 10 (30.3) | 7 (21.2) |
| Gamma-glutamyltransferase increased | 3 (9.1) | 2 (6.1) |
| Alanine aminotransferase increased | 3 (9.1) | 2 (6.1) |
| Aspartate aminotransferase increased | 3 (9.1) | 1 (3.0) |
| White blood cell count decreased | 1 (3.0) | 0 |
| Platelet count decreased | 1 (3.0) | 1 (3.0) |
| Neutrophil count decreased | 1 (3.0) | 0 |
| Pneumonia | 1 (3.0) | 0 |
| Conjunctivitis | 1 (3.0) | 0 |
| Pelvic inflammatory disease | 1 (3.0) | 1 (3.0) |
| Large intestine perforation | 1 (3.0) | 1 (3.0) |
| Abdominal pain | 1 (3.0) | 0 |
| Mouth ulceration | 1 (3.0) | 1 (3.0) |
| Hyperthyroidism | 1 (3.0) | 0 |
| Autoimmune thyroiditis | 1 (3.0) | 0 |
| Dermatitis bullous | 1 (3.0) | 0 |
| Rash | 1 (3.0) | 1 (3.0) |
| Hyperglycemia | 1 (3.0) | 1 (3.0) |
| Hematuria | 1 (3.0) | 1 (3.0) |
| Female genital tract fistula | 1 (3.0) | 1 (3.0) |
| Sinus tachycardia | 1 (3.0) | 0 |

Data are n (%).

**Supplementary Table 3. Treatment-related adverse events leading to interruption of famitinib**

| | All patients (N=33) | |
|---|---|---|
| | **Any grade** | **Grade 3 or higher** |
| Any | 25 (75.8) | 19 (57.6) |
| Neutrophil count decreased | 6 (18.2) | 5 (15.2) |
| White blood cell count decreased | 5 (15.2) | 3 (9.1) |
| Platelet count decreased | 5 (15.2) | 1 (3.0) |
| Gamma-glutamyltransferase increased | 4 (12.1) | 2 (6.1) |
| Alanine aminotransferase increased | 4 (12.1) | 2 (6.1) |
| Aspartate aminotransferase increased | 4 (12.1) | 1 (3.0) |
| Hematochezia | 3 (9.1) | 0 |
| Abdominal pain | 2 (6.1) | 0 |
| Gingival bleeding | 2 (6.1) | 0 |
| Large intestine perforation | 1 (3.0) | 1 (3.0) |
| Noninfective gingivitis | 1 (3.0) | 0 |
| Diarrhea | 1 (3.0) | 0 |
| Mouth ulceration | 1 (3.0) | 1 (3.0) |
| Vomiting | 1 (3.0) | 0 |
| Glossitis | 1 (3.0) | 0 |
| Lower gastrointestinal hemorrhage | 1 (3.0) | 0 |
| Rectal ulcer | 1 (3.0) | 0 |
| Hypertension | 6 (18.2) | 5 (15.2) |
| Anemia | 6 (18.2) | 6 (18.2) |
| Proteinuria | 4 (12.1) | 0 |
| Hematuria | 1 (3.0) | 1 (3.0) |
| Hypertriglyceridemia | 2 (6.1) | 2 (6.1) |
| Hyperglycemia | 1 (3.0) | 1 (3.0) |
| Decreased appetite | 1 (3.0) | 0 |
| Cystitis | 1 (3.0) | 0 |
| Pneumonia | 1 (3.0) | 0 |
| Conjunctivitis | 1 (3.0) | 0 |
| Pelvic inflammatory disease | 1 (3.0) | 1 (3.0) |
| Gingivitis | 1 (3.0) | 1 (3.0) |
| Hand-foot syndrome | 2 (6.1) | 2 (6.1) |
| Rash | 1 (3.0) | 1 (3.0) |
| Epistaxis | 1 (3.0) | 0 |
| Hemoptysis | 1 (3.0) | 0 |

| | | |
|---|---|---|
| Hyperthyroidism | 1 (3.0) | 0 |
| Autoimmune thyroiditis | 1 (3.0) | 1 (3.0) |
| Vaginal hemorrhage | 2 (6.1) | 1 (3.0) |
| Female genital tract fistula | 1 (3.0) | 1 (3.0) |
| Cystitis radiation | 1 (3.0) | 0 |
| Bone pain | 1 (3.0) | 0 |
| Tumor hemorrhage | 1 (3.0) | 0 |
| Tumor exudation | 1 (3.0) | 0 |
| Asthenia | 1 (3.0) | 0 |
| Sinus tachycardia | 1 (3.0) | 0 |

Data are n (%).

**Supplementary Table 4. Treatment-related adverse events leading to dose reduction of famitinib**

| | All patients (N=33) | |
|---|---|---|
| | **Any grade** | **Grade 3 or higher** |
| Any | 17 (51.5) | 4 (12.1) |
| Gamma-glutamyltransferase increased | 4 (12.1) | 0 |
| Platelet count decreased | 3 (9.1) | 2 (6.1) |
| White blood cell count decreased | 2 (6.1) | 0 |
| Alanine aminotransferase increased | 2 (6.1) | 0 |
| Aspartate aminotransferase increased | 1 (3.0) | 0 |
| Neutrophil count decreased | 1 (3.0) | 0 |
| Proteinuria | 4 (12.1) | 1 (3.0) |
| Urogenital fistula | 1 (3.0) | 0 |
| Hand-foot syndrome | 2 (6.1) | 0 |
| Hypertension | 2 (6.1) | 1 (3.0) |
| Bone pain | 1 (3.0) | 0 |
| Diarrhea | 1 (3.0) | 0 |

Data are n (%).

**Supplementary Table 5. Immune-related adverse events**

| | All patients (N=33) | |
|---|---|---|
| | **Any grade** | **Grade 3 or higher** |
| Any | 12 (36.4) | 1 (3.0) |
| Hypothyroidism | 8 (24.2) | 0 |
| Autoimmune thyroiditis | 1 (3.0) | 1 (3.0) |
| Rash | 1 (3.0) | 1 (3.0) |
| Mouth ulceration | 1 (3.0) | 1 (3.0) |
| Immune-mediated hypothyroidism | 2 (6.1) | 0 |
| Hyperthyroidism | 1 (3.0) | 0 |
| Dermatitis bullous | 1 (3.0) | 0 |
| Conjunctivitis | 1 (3.0) | 0 |

Data are n (%). Immune-related adverse events indicates adverse events which related to the immunological mechanism of action of immunotherapy.

**Supplementary Table 6. Participating sites**

| Investigators | Participating sites | Number of patients |
|---|---|---|
| Xiaohua Wu / Lingfang Xia | Fudan University Shanghai Cancer Center, Shanghai, China | 20 |
| Qi Zhou/ Jin Shu | Affiliated Tumor Hospital of Chongqing University, Chongqing, China | 6 |
| Yunong Gao | Beijing Cancer Hospital, Beijing, China | 2 |
| Wenjing Hu | Nanjing Drum Tower Hospital, Nanjing, China | 2 |
| Ge Lou | Affiliated Tumor Hospital of Harbin Medical University, Harbin, China | 1 |
| Hong Sun | Obstetrics & Gynecology Hospital of Fudan University, Shanghai, China | 1 |
| Jianqing Zhu | Cancer Hospital of the University of Chinese Academy of Sciences (Zhejiang Cancer Hospital), Hangzhou, China | 1 |

**Supplementary Note 1. Study protocol**

# AN OPEN-LABEL, MULTICENTER PHASE II CLINICAL STUDY OF ANTI-PD-1 ANTIBODY SHR-1210 COMBINED WITH FAMITINIB MALATE IN PATIENTS WITH ADVANCED URINARY SYSTEM TUMORS AND GYNECOLOGICAL TUMORS

| | |
|---|---|
| Protocol No.: | SHR-1210-II-213 |
| Study Phase: | II |
| Compound Code: | SHR-1210 |
| | SHR-1020 |
| Compound Name: | Camrelizumab |
| | Famitinib Malate |
| Medical Director: | Quanren Wang |
| Leading Site of Clinical Study: | Fudan University Shanghai Cancer Center |
| Principal Investigators: | Prof. Dingwei Ye; Prof. Xiaohua Wu |
| Version No.: | V4.0 |
| Version Date: | 5 Nov., 2020 |

**Sponsor:** Jiangsu Hengrui Pharmaceuticals Co., Ltd.

No. 7 Kunlunshan Road, Lianyungang Economic and Technological Development Zone, Jiangsu 222047, China

**Confidentiality Statement**

The information contained in this protocol is confidential and is intended for use by clinical investigators only. Any disclosure is not permitted unless requested by current laws or regulations. The copyright is owned by Jiangsu Hengrui Pharmaceuticals Co., Ltd. or its subsidiaries. Any copy or distribution of information herein to any individuals not participating in this clinical study is not allowed, unless a confidentiality agreement has been signed with Jiangsu Hengrui Pharmaceuticals Co., Ltd. or its subsidiaries.

# VERSION HISTORY/REVISION HISTORY

| Document | Version Date | Amendment Rationale and Summary of Changes |
|---|---|---|
| Version 1.0 | 25 Jul., 2018 | Not applicable |
| Version 1.1 | 30 Sep., 2018 | Revised wording and detailed description based on the review comments of the ethics committee |
| Version 2.0 | 6 Aug., 2019 | 1. Revised the requirements for previous systemic treatment in the inclusion criteria for renal cancer, urothelial carcinoma, cervical cancer, endometrial cancer, and ovarian cancer, such that the inclusion criteria are more precise, to further meet the needs of efficacy exploration;<br>2. Modified and added exclusion criteria: patients with CTCAE Grade ≥ 2 hemorrhage within 4 weeks prior to the study treatment; patients with radiation-induced enteritis after receiving pelvic radiotherapy within 12 months prior to the study treatment;<br>3. Based on the results of previous studies and the preliminary safety results of this study, after full discussion with the investigators, it was decided to add the dose level of famitinib at 20 mg combined with SHR-1210 at a fixed dose of 200 mg q2w. Observation of clinically significant toxicity and pharmacokinetic (PK) blood sampling will be first performed in 12 subjects at this dose level. Also, the corresponding safety and efficacy visit points in the Schedule of Activities were revised;<br>4. Added some biomarker detection parameters in the exploratory endpoints: detection of MMR or MSI-H for ovarian cancer and endometrial cancer and detection of proportion of abnormal FGFR2/3 for urothelial carcinoma;<br>5. Added "B-mode ultrasonography of lower extremity veins" in the examinations of screening period and the end of treatment;<br>6. Revised the wording of the statistics part, and changed the description of exploratory analysis according to the revisions made;<br>7. Revised wording and logic. |
| Version 2.1 | 8 Oct., 2019 | 1. Revised the detection methods and sample collection for biomarkers MSI and FGFR2/3;<br>2. Revised wording. |

| Document | Version Date | Amendment Rationale and Summary of Changes |
|---|---|---|
| Version 3.0 | 8 Jul., 2020 | 1. Added Cohorts 6 and 7 to evaluate the efficacy of SHR-1210 monotherapy in urothelial carcinoma and famitinib monotherapy in cervical cancer and urothelial carcinoma;<br>2. Revised wording;<br>3. Deleted the content related to SHR-1210 q2w administration. |
| Version 4.0 | 5 Nov., 2020 | 1. Added Cohort 8 to evaluate the efficacy of SHR-1210 combined with famitinib for the treatment of advanced renal cancer that has progressed after previous anti-PD-1 or PD-L1 antibody therapy;<br>2. Added the description that subjects in Cohorts 6 and 7 can continue treatment with SHR-1210 combined with famitinib after PD;<br>3. Revised logic. |

# Sponsor's Signature Page

I have read and confirmed this clinical study protocol (protocol no.: SHR-1210-II-213, version no.: 4.0, version date: 5 Nov., 2020). I agree to fulfill my duties in accordance with Chinese laws, the Declaration of Helsinki, the Good Clinical Practice (GCP) in China, and this study protocol.

**Sponsor:** Jiangsu Hengrui Pharmaceuticals Co., Ltd.

| Quanren Wang | | |
|---|---|---|
| Study Director (print) | Study Director (signature) | Signature Date (DD/MM/YYYY) |

# Principal Investigator's Signature Page (Leading Site)

I will carefully execute the duties as an investigator in accordance with the Good Clinical Practice (GCP) in China, and personally participate in or directly lead this clinical study. I have received the Investigator's Brochure for the investigational products; I have read the materials of preclinical studies of the investigational products and the protocol for this clinical study. I agree to fulfill my duties in accordance with Chinese laws, the Declaration of Helsinki, the Chinese GCP, and this study protocol. I agree that any modifications to the protocol must be reviewed and approved by the sponsor, and can only be implemented upon approval by the ethics committee, unless measures must be taken to protect the safety, rights, and interests of the subjects. It is my responsibility to make clinically relevant medical decisions to ensure appropriate and timely treatments in subjects experiencing adverse events during the study period, and to document and report such adverse events in accordance with relevant state regulations. I will document all data in a truthful, accurate, complete, and timely manner. I agree to be monitored and audited by the clinical research associate or auditor assigned by the sponsor, and to be inspected by the drug regulatory authorities, to ensure the quality of the clinical study. I will keep the personal information of and matters related to the subjects confidential. I agree to disclose my full name and occupation to the sponsor, and the expenses related to the clinical study upon request. I agree not to engage in any commercial and economic activities related to this study. I agree for the study results to be used for drug registration and publication. I will provide a resume before the start of the study, submit it to the ethics committee, and to the drug regulatory authorities for filing purposes.

**Study Site:** Fudan University Shanghai Cancer Center

| Prof. Dingwei Ye | | |
|---|---|---|
| Principal Investigator (print) | Principal Investigator (signature) | Signature Date (DD/MM/YYYY) |

# Principal Investigator's Signature Page (Leading Site)

I will carefully execute the duties as an investigator in accordance with the Good Clinical Practice (GCP) in China, and personally participate in or directly lead this clinical study. I have received the Investigator's Brochure for the investigational products; I have read the materials of preclinical studies of the investigational products and the protocol for this clinical study. I agree to fulfill my duties in accordance with Chinese laws, the Declaration of Helsinki, the Chinese GCP, and this study protocol. I agree that any modifications to the protocol must be reviewed and approved by the sponsor, and can only be implemented upon approval by the ethics committee, unless measures must be taken to protect the safety, rights, and interests of the subjects. It is my responsibility to make clinically relevant medical decisions to ensure appropriate and timely treatments in subjects experiencing adverse events during the study period, and to document and report such adverse events in accordance with relevant state regulations. I will document all data in a truthful, accurate, complete, and timely manner. I agree to be monitored and audited by the clinical research associate or auditor assigned by the sponsor, and to be inspected by the drug regulatory authorities, to ensure the quality of the clinical study. I will keep the personal information of and matters related to the subjects confidential. I agree to disclose my full name and occupation to the sponsor, and the expenses related to the clinical study upon request. I agree not to engage in any commercial and economic activities related to this study. I agree for the study results to be used for drug registration and publication. I will provide a resume before the start of the study, submit it to the ethics committee, and to the drug regulatory authorities for filing purposes.

**Study Site:** Fudan University Shanghai Cancer Center

| Prof. Xiaohua Wu | | |
|---|---|---|
| Principal Investigator (print) | Principal Investigator (signature) | Signature Date (DD/MM/YYYY) |

# Principal Investigator's Signature Page (Study Site)

I will carefully execute the duties as an investigator in accordance with the Good Clinical Practice (GCP) in China, and personally participate in or directly lead this clinical study. I have received the Investigator's Brochure for the investigational products; I have read the materials of preclinical studies of the investigational products and the protocol for this clinical study. I agree to fulfill my duties in accordance with Chinese laws, the Declaration of Helsinki, the Chinese GCP, and this study protocol. I agree that any modifications to the protocol must be reviewed and approved by the sponsor, and can only be implemented upon approval by the ethics committee, unless measures must be taken to protect the safety, rights, and interests of the subjects. It is my responsibility to make clinically relevant medical decisions to ensure appropriate and timely treatments in subjects experiencing adverse events during the study period, and to document and report such adverse events in accordance with relevant state regulations. I will document all data in a truthful, accurate, complete, and timely manner. I agree to be monitored and audited by the clinical research associate or auditor assigned by the sponsor, and to be inspected by the drug regulatory authorities, to ensure the quality of the clinical study. I will keep the personal information of and matters related to the subjects confidential. I agree to disclose my full name and occupation to the sponsor, and the expenses related to the clinical study upon request. I agree not to engage in any commercial and economic activities related to this study. I agree for the study results to be used for drug registration and publication. I will provide a resume before the start of the study, submit it to the ethics committee, and to the drug regulatory authorities for filing purposes.

**Study Site:** ______________________

| ______________________ | ______________________ | ______________________ |
|---|---|---|
| Principal Investigator (print) | Principal Investigator (signature) | Signature Date (DD/MM/YYYY) |

# TABLE OF CONTENTS

VERSION HISTORY/REVISION HISTORY ..... 1
SCHEDULE OF ACTIVITIES ..... 13
ABBREVIATIONS ..... 22
1. INTRODUCTION: BACKGROUND AND SCIENTIFIC RATIONALE ..... 24
1.1. BACKGROUND ..... 24
1.2. SCIENTIFIC RATIONALE ..... 33
1.2.1. Study rationale ..... 34
1.2.2. Rationale for dosing regimen design ..... 36
1.3. POTENTIAL RISKS AND BENEFITS ..... 38
1.3.1. Known potential risks ..... 38
1.3.2. Known potential benefits ..... 40
1.4. STUDY OBJECTIVES ..... 40
1.4.1. Primary objectives ..... 40
1.4.2. Secondary objectives ..... 40
1.4.3. Exploratory objective ..... 41
1.5. STUDY ENDPOINTS ..... 41
1.5.1. Primary endpoint ..... 41
1.5.2. Secondary endpoints ..... 41
1.5.3. Exploratory endpoints ..... 42
2. STUDY DESIGN ..... 42
2.1. OVERVIEW OF STUDY DESIGN ..... 42
2.1.1. Clinically significant toxicity ..... 44
2.1.2. Follow-up management of subjects with clinically significant toxicity ..... 45
2.1.3. Randomization ..... 45
2.2. BLOOD SAMPLING FOR PK AND ADA ANALYSIS ..... 46
2.2.1. Study procedure and arrangement of blood sampling points ..... 46
2.2.2. Blood sample processing and testing ..... 47
2.3. ACQUISITION, COLLECTION, AND PROCESSING OF BIOMARKERS ..... 47
3. SELECTION AND WITHDRAWAL OF SUBJECTS ..... 48
3.1. INCLUSION CRITERIA ..... 48
3.2. EXCLUSION CRITERIA ..... 51
3.3. LIFESTYLE REQUIREMENTS ..... 53
3.3.1. Contraception ..... 53
3.3.2. Exposure to sunlight ..... 53
3.4. DISCONTINUATION/WITHDRAWAL OF SUBJECTS ..... 53

3.4.1. Criteria for treatment discontinuation.....53
3.4.2. Study withdrawal criteria.....54
3.4.3. Procedures for withdrawal from study or treatment discontinuation.....54
3.5. PREMATURE TERMINATION OR SUSPENSION OF STUDY.....55
3.6. DEFINITION OF END OF STUDY.....55

4. STUDY MEDICATION.....56

4.1. DRUG ALLOCATION.....56
4.2. DOSAGE FORM, APPEARANCE, PACKAGING, AND LABEL.....56
4.2.1. Packaging and labeling.....57
4.3. STORAGE OF INVESTIGATIONAL PRODUCTS.....58
4.4. DRUG PREPARATION, DISPENSATION, AND RETURN.....58
4.4.1. Disposal of investigational products.....59
4.5. ADMINISTRATION OF INVESTIGATIONAL PRODUCTS.....59
4.5.1. Precautions for special drug delivery devices.....59
4.6. DOSING REGIMEN.....59
4.7. DOSE MODIFICATION.....60
4.8. CONCOMITANT TREATMENT.....65
4.8.1. Other anti-tumor/cancer or investigational products.....65
4.8.2. Supportive care.....65
4.8.3. Immunological agents.....66
4.8.4. Drugs that may have drug-drug interactions with famitinib.....66
4.8.5. Drugs that prolong the QT interval of the heart.....66
4.8.6. Surgery or palliative radiotherapy.....67

5. STUDY PROCEDURES.....67

5.1. SCREENING.....67
5.2. ENROLLMENT.....70
5.3. TREATMENT PERIOD.....71
5.4. END OF TREATMENT/WITHDRAWAL.....73
5.5. FOLLOW-UP PERIOD.....73
5.6. CONTINUING TREATMENT AFTER PROGRESSIVE DISEASE.....74
5.6.1. Criteria for continuing treatment.....74
5.6.2. Other considerations for continuing treatment.....75

6. EVALUATIONS.....75

6.1. EFFICACY EVALUATION.....75
6.1.1. Efficacy endpoints.....75
6.1.2. Criteria for efficacy evaluation.....77
6.2. SAFETY EVALUATION.....78
6.2.1. Pregnancy test.....78

6.2.2. Adverse event ... 79
6.2.3. Laboratory safety evaluation ... 79
6.2.4. Vital signs and physical examination ... 80
6.2.5. 12-lead ECG ... 80
6.3. PHARMACOKINETIC AND SHR-1210 ADA EVALUATION ... 81
6.4. BIOMARKER EVALUATION ... 81
7. ADVERSE EVENT REPORTING ... 82
7.1. ADVERSE EVENT (AE) ... 82
7.1.1. AE severity grading criteria ... 82
7.1.2. Causality assessment ... 83
7.2. SERIOUS ADVERSE EVENT (SAE) ... 83
7.2.1. Definition of SAE ... 83
7.2.2. Hospitalization ... 83
7.2.3. Progressive disease ... 85
7.2.4. SAE reporting ... 85
7.3. PREGNANCY ... 86
7.4. SPECIAL INTEREST EVENT ... 86
7.5. COLLECTION AND FOLLOW-UP OF AES/SAES ... 87
8. MANAGEMENT OF ADVERSE EVENTS ... 88
8.1. IMMUNE-RELATED ADVERSE EVENT (IRAE) ... 88
8.2. INFUSION REACTION ... 92
8.3. SYMPTOMATIC TREATMENT FOR FAMITINIB-RELATED ADVERSE REACTIONS ... 93
9. CLINICAL MONITORING ... 97
10. DATA ANALYSIS/STATISTICAL METHODS ... 98
10.1. STATISTICAL HYPOTHESIS AND DISCRIMINATORY RULES ... 98
10.2. SAMPLE SIZE ... 99
10.3. STATISTICAL ANALYSIS PLAN ... 101
10.4. ANALYSIS POPULATION ... 102
10.5. STATISTICAL METHODS ... 102
10.5.1. Basic methods ... 103
10.5.2. Analysis of primary efficacy endpoint ... 103
10.5.3. Analysis of secondary efficacy endpoints ... 104
10.5.4. Handling of missing data ... 105
10.5.5. Safety analysis ... 105
10.5.6. Pharmacokinetic analysis ... 106
10.5.7. Immunogenicity analysis ... 106
10.5.8. Multiple comparison/multiplicity ... 106

10.5.9. Exploratory analysis ... 106
10.6. INTERIM ANALYSIS ... 107

11. DATA MANAGEMENT METHOD ... 108

11.1. DATA RECORDING ... 108
11.1.1. Filing of study medical records ... 108
11.1.2. eCRF entry ... 108
11.1.3. eCRF review ... 109
11.2. DATA MONITORING ... 109
11.3. DATA MANAGEMENT ... 109
11.3.1. EDC database establishment ... 109
11.3.2. Data entry and verification ... 109
11.3.3. Database lock ... 110
11.3.4. Data archiving ... 110
11.3.5. Protocol deviation ... 110

12. SOURCE DATA AND DOCUMENTS ... 110

13. QUALITY ASSURANCE AND QUALITY CONTROL ... 111

14. REGULATORY ETHICS, INFORMED CONSENT, AND SUBJECT PROTECTION ... 112

14.1. REGULATORY CONSIDERATIONS ... 112
14.2. ETHICAL STANDARDS ... 112
14.3. INDEPENDENT ETHICS COMMITTEE ... 113
14.4. INFORMED CONSENT ... 113
14.4.1. Informed consent form and other written information for subjects ... 113
14.4.2. Informed consent process and records ... 114
14.5. CONFIDENTIALITY OF SUBJECT INFORMATION ... 114

15. PUBLICATION OF STUDY RESULTS ... 115

16. CLINICAL STUDY PROGRESS ... 115

17. REFERENCES ... 116

APPENDIX I PERFORMANCE STATUS (ECOG) ... 117

APPENDIX II RESPONSE EVALUATION CRITERIA IN SOLID TUMORS (RECIST V1.1) 118

APPENDIX III PERCENT BONE MARROW CONTENT IN HUMAN SKELETON ... 132

APPENDIX IV MANAGEMENT PRINCIPLES FOR IMMUNE-RELATED ADVERSE EVENTS ... 133

# LIST OF TABLES

Table 1. Clinical studies of marketed PD-1/PD-L1 inhibitor monotherapies in advanced urothelial carcinoma. 27
Table 2. Comparison of the inhibitory activity of sunitinib, famitinib, and famitinib metabolite against receptor tyrosine kinases. 31
Table 3. Combination of anti-PD-1/PD-L1 inhibitors and VEGF/VEGFR inhibitors for the treatment of tumors. 33
Table 4. Acquisition and collection of biomarker samples. 48
Table 5. Dose modifications. 62
Table 6. Time limits of AE/SAE collection. 88
Table 7. Recommendations for treatment of infusion reactions. 93
Table 8. Sample size by cohort - Stage I/II. 100
Table 9. Sample size by cohort and in Stage I/II. 100
Table 10. Cohorts 6-7: Calculation of sample size by Simon's (minimax) two-stage method. 101
Table 11. Cohorts 8: Calculation of sample size by Simon's (minimax) two-stage method. 101

# PROTOCOL SYNOPSIS

| | |
|---|---|
| **Study Title** | An Open-Label, Multicenter Phase II Clinical Study of Anti-PD-1 Antibody SHR- 1210 Combined with Famitinib Malate in Patients with Advanced Urinary System Tumors and Gynecological Tumors |
| **Protocol No.** | SHR-1210-II-213 |
| **Version No.** | 4.0 |
| **Version Date** | 5 Nov., 2020 |
| **Investigational Products** | Recombinant humanized anti-PD-1 monoclonal antibody for injection (drug code: SHR-1210)<br>(Manufacturer: Suzhou Suncadia Biopharmaceuticals Co., Ltd.)<br>Famitinib malate capsules<br>(Manufacturer: Jiangsu Hengrui Pharmaceuticals Co., Ltd.) |
| **Sponsor** | Jiangsu Hengrui Pharmaceuticals Co., Ltd. |
| **Leading Site** | Fudan University Shanghai Cancer Center |
| **Principal Investigators** | Prof. Dingwei Ye<br>Prof. Xiaohua Wu |
| **Participating Study Sites** | Approximately 30 sites |
| **Study Objectives** | **Primary objectives**<br>• To evaluate the efficacy of SHR-1210 combined with famitinib in subjects with advanced renal cell carcinoma, urothelial carcinoma, advanced cervical cancer, recurrent ovarian cancer, and endometrial cancer;<br>• To evaluate the efficacy of SHR-1210 monotherapy in subjects with urothelial carcinoma;<br>• To evaluate the efficacy of famitinib monotherapy in subjects with urothelial carcinoma and cervical cancer.<br>**Secondary objectives**<br>• To evaluate the safety and tolerability of SHR-1210 combined with famitinib in the treatment of various tumors;<br>• To evaluate the safety and tolerability of SHR-1210 monotherapy in subjects with urothelial carcinoma;<br>• To evaluate the safety and tolerability of famitinib monotherapy in subjects with urothelial carcinoma and cervical cancer;<br>• To evaluate the PK of SHR-1210 combined with famitinib in subjects with advanced solid tumors and famitinib monotherapy in subjects with urothelial carcinoma and cervical cancer;<br>• To investigate the anti-SHR-1210 antibodies (ADAs) in subjects receiving combination therapy.<br>**Exploratory objective**<br>To explore biomarkers for response prediction. |

| | |
|---|---|
| **Study Endpoints** | **Primary endpoint**<br>Objective response rate (ORR) as per RECIST 1.1<br>**Secondary endpoints**<br>**Efficacy:**<br>• Duration of response (DOR);<br>• Disease control rate (DCR);<br>• Time to response (TTR);<br>• Progression-free survival (PFS) as per RECIST 1.1;<br>• Overall survival (OS) and 12-month overall survival rate<br>**Safety and tolerability:**<br>• Adverse events (AEs): including type, incidence, grade (according to NCI-CTCAE v4.03 criteria), severity, duration, and causality with the investigational products;<br>• Laboratory abnormalities: including type, incidence, and grade (according to NCI-CTCAE v4.03 criteria);<br>• Vital signs: including blood pressure, pulse, respiratory rate, body temperature, ECG, and ECOG PS score;<br>• Incidence of dose interruption, reduction, and discontinuation due to treatment-related AEs.<br>**Pharmacokinetics:**<br>• Plasma concentrations and PK parameters (if applicable) of famitinib and its main metabolites for combination therapy and monotherapy, including $C_{max}$, $T_{max}$, $AUC_{0-24\ h}$, CL/F, and V/F;<br>• Blood concentrations of anti-PD-1 antibody SHR-1210 during combination therapy.<br>**Others:**<br>• Proportion of subjects with anti-SHR-1210 antibodies (ADAs) during combination therapy.<br>**Exploratory endpoints**<br>The proportion of PD-L1-positive cells in tumor tissue (for subjects receiving SHR-1210 combination therapy or monotherapy only), proportion of subjects with dMMR or MSI-H (for endometrial cancer and ovarian cancer only), and proportion of abnormal FGFR2/3 gene (for urothelial carcinoma treated with combination therapy only) will be evaluated to explore the relationship of PD-L1 expression and/or other biomarkers with efficacy (such as ORR/PFS). |
| **Study Population** | Patients with advanced urinary system tumors or gynecological tumors (including advanced renal cell carcinoma, urothelial carcinoma, recurrent ovarian cancer, endometrial cancer, and cervical cancer) |
| **Study Design** | This study is an open-label, multicenter phase II clinical study to observe and evaluate the efficacy and safety of anti-PD-1 antibody SHR-1210 combined with famitinib malate in subjects with advanced urinary system tumors and gynecological tumors, and the efficacy and safety of SHR-1210 monotherapy in treatment of urothelial carcinoma, and famitinib monotherapy in treatment of urothelial carcinoma and cervical cancer.<br>The subjects will be divided into 8 cohorts according to tumor type or investigational products: |

| | |
|---|---|
| | Cohort 1: renal cell carcinoma (no prior anti-PD-1/PD-L1/CTLA-4 antibody treatment)<br>Cohort 2: urothelial carcinoma<br>Cohort 3: recurrent ovarian cancer<br>Cohort 4: endometrial cancer<br>Cohort 5: cervical cancer<br>Cohort 6: SHR-1210 monotherapy (urothelial carcinoma)<br>Cohort 7: famitinib monotherapy (urothelial carcinoma and cervical cancer)<br>Cohort 8: renal cell carcinoma (progression after prior anti-PD-1/PD-L1/CTLA-4 antibody treatment)<br>➢ **Cohorts 1-5**:<br>A combination therapy of oral famitinib 20 mg, qd and SHR-1210 200 mg fixed dose, intravenous infusion (iv) once every 3 weeks (q3w) will be selected in this study, to observe its efficacy and safety in 5 types of tumors, i.e., renal cell carcinoma, urothelial carcinoma, recurrent ovarian cancer, endometrial cancer, and cervical cancer. 22-53 subjects will be enrolled in each of Cohorts 1-5. After preliminary enrollment and efficacy observation, further enrollment will be adjusted depending on the preliminary efficacy of each cohort. If a certain cohort shows an ideal ORR, further expansion study will be discussed for the cohort.<br>Safety data from observation of the first 12 subjects who are enrolled in Cohorts 1- 5 after completion of the first two cycles of combination therapy will be summarized and analyzed. If clinically significant toxicity is observed in ≥ 4 out of these 12 subjects, or if > 30% subjects (for Cohorts 1-5 only) requires a dose reduction of famitinib, the combination of SHR-1210 with famitinib 20 mg, qd will be deemed to be poorly tolerated. Subjects enrolled subsequently should be given a combination of famitinib 15 mg, qd and SHR-1210 200 mg, q3w for observation.<br>The first 12 subjects enrolled in Cohorts 1-5 will also undergo blood sampling for PK analysis to explore the blood concentrations and PK parameters of famitinib and SHR-1210 in combination therapy.<br>➢ **Cohorts 6-7**:<br>In addition, subjects with urothelial carcinoma and cervical cancer will be screened for monotherapy study of Cohorts 6 and 7. Among them, subjects with urothelial carcinoma will be randomly assigned to Cohort 6 (SHR-1210 monotherapy group, 200 mg fixed dose, intravenous infusion (iv), q3w) or Cohort 7 (famitinib monotherapy group, 25 mg, qd, continuous oral administration) to receive corresponding pharmacological treatment, and the efficacy and safety will be observed. Subjects with cervical cancer will be directly enrolled in Cohort 7 to receive famitinib monotherapy (25 mg, qd, continuous oral administration), and the efficacy and safety will be observed. 14-23 subjects with urothelial carcinoma and cervical cancer will be enrolled in each of Cohorts 6 and 7. After Stage I enrollment and efficacy observation, whether to proceed to Stage II enrollment will be decided according to the number of subjects with response in Stage I.<br>Subjects in Cohort 7 will also undergo blood sampling for PK analysis (only at sites with conditions for PK blood sampling and processing) to analyze the PK of famitinib in subjects with urothelial carcinoma and cervical cancer.<br>➢ **Cohort 8**:<br>Subjects with advanced renal cell carcinoma will be screened to Cohort 8 to receive SHR-1210 (200 mg, q3w, iv) combined with famitinib (20 mg, qd, continuous oral administration), and the efficacy and safety will be observed. 21 subjects will be enrolled in Stage I. After Stage I enrollment and efficacy observation, whether to proceed to Stage II enrollment will be decided according to the number of subjects with response in Stage I. |

| | |
|---|---|
| | Subjects in Cohort 8 will undergo blood sampling for PK analysis (only at sites with conditions for PK blood sampling and processing) to explore the PK of famitinib in combination therapy of renal cancer.<br>All subjects will be treated until the criteria for treatment discontinuation specified in the protocol are met. After the end of treatment, subjects will continue to undergo safety visits and survival follow-ups. Subjects who discontinue the treatment due to reasons other than progressive disease (PD)/death will also be followed up for PD after the end of treatment.<br>For Cohorts 6 and 7, if subjects discontinue treatment (SHR-1210 monotherapy or famitinib monotherapy) due to PD, with the informed consent of the subjects, SHR-1210 combined with famitinib can be used to continue treatment until the study treatment discontinuation criteria specified in the study are met.<br>After enrollment in the study, subjects will undergo safety follow-ups prior to administration on D1 and D7 (± 1 d) of Cycle 1, as well as D1 (± 3 d) of each subsequent cycle. Imaging assessments should be conducted once every 3 cycles (9 weeks) to evaluate efficacy from the start of treatment until radiographic progression, commencement of new anti-tumor treatment, withdrawal of informed consent, loss to follow-up, or death. The visit cycle and efficacy evaluation of subjects who continue treatment after PD in Cohorts 6 and 7 are the same as those before PD. Existing paraffin-embedded tumor tissue sections will be acquired/collected during the screening period. Prior to the first dose, it is also recommended to collect fresh biopsy specimens (core needle biopsy), prepare 3 tumor sections with a thickness of 4-5 μm after fixation and embedding, and collect 5 mL of whole blood for biomarker detection, including but not limited to: proportion of PD-L1-positive cells in tumor tissue (for subjects receiving SHR-1210 combination therapy or monotherapy only), MMR (for endometrial cancer and ovarian cancer only), and FGFR2/3 mutation (for urothelial carcinoma treated with combination therapy only). The proportion of PD-L1-positive cells in tumor tissue (for subjects receiving SHR-1210 combination therapy or monotherapy only), proportion of subjects with dMMR or MSI-H (for endometrial cancer and ovarian cancer only), and proportion of abnormal FGFR2/3 (for urothelial carcinoma treated with combination therapy only) will be evaluated to explore the relationship of PD-L1 expression and/or other biomarkers with efficacy (such as ORR/PFS). |
| **Dosing Regimen** | SHR-1210: administered via intravenous infusion (premedication not required) at a fixed dose of 200 mg within 30 min (not less than 20 min, not more than 60 min), once every 3 weeks; each cycle contains 3 weeks and the longest dosing period is 2 years.<br>Famitinib malate capsules: administered orally once a day before or after a meal (recommended at a fixed time: within 0.5 h after a meal) at a dose of 20 mg (for subjects in Cohorts 1-5 and 8 and for subjects who continue treatment after PD in Cohort 6) or 25 mg (for Cohort 7). The drug should be administered continuously in cycles of 3 weeks. In Cohort 7, the dose and frequency of famitinib for subjects who continue treatment after PD are the same as those before PD. If the dose of famitinib before PD is 25 mg, the dose of famitinib should be adjusted to 20 mg (continuous administration) for continuing treatment. Subjects in Cohorts 7 and 8 who participate in the blood sampling for famitinib PK analysis should take famitinib after blood sampling before C2D1 and C3D1 administration. Subjects in the PK study should take famitinib orally before breakfast on the day of blood sampling for PK study. |

| | |
|---|---|
| **Definition of Clinically Significant Toxicity** | The definition is limited to events observed in the first 12 subjects enrolled in Cohorts 1-5 within the first 2 cycles that are deemed related to the investigational products by the investigators and meet the following:<br>1. Grade 4 hematologic toxicity that lasts ≥ 3 days, Grade ≥ 3 thrombocytopenia with hemorrhage, or Grade ≥ 3 neutropenia with fever and infection;<br>2. Grade ≥ 3 non-hematologic toxicity (except for laboratory abnormalities), Grade 3 hypertension, rash, diarrhea, nausea, and vomiting that cannot be effectively controlled after symptomatic treatment;<br>3. Grade ≥ 3 laboratory abnormalities that lead to hospitalization or last ≥ 7 days;<br>4. Related toxicity resulting in failure to complete 2 administrations of SHR- 1210 within the first two cycles or normal SHR-1210 administration in Cycle 3 (treatment delay > 7 days);<br>5. Related toxicity resulting in famitinib interruption for > 14 days.<br>If any of the first 12 subjects fails to complete treatment observation for two cycles due to non-drug-related toxicity, the subject must be replaced. |
| **Blood Sampling for PK and ADA Analysis** | ➢ **Cohorts 1-5**:<br>The first 12 subjects enrolled in Cohorts 1-5 will undergo blood sampling for PK and ADA analysis.<br>For SHR-1210 PK analysis, blood samples will be collected within 30 min before administration and within 5 min after administration (including flushing) of the first SHR-1210 dose; within 30 min before administration and within 5 min after administration (including flushing) of SHR-1210 on administration days of Cycles 2, 3, and 4; within 30 min pre-administration every 4 cycles thereafter; at the end of treatment or upon withdrawal from study (the documented time of last SHR-1210 administration prior to the end of study); at 30 days after the end of SHR-1210 treatment. (If subject completes the treatment before scheduled blood sampling is completed, the analysis should be based on actual completed sampling).<br>ADA blood samples will be simultaneously collected along with PK samples before each administration, at the end of SHR-1210 treatment, and at 30 days after the end of SHR-1210 treatment.<br>For SHR-1210, 4 mL should be collected for PK analysis and 4 mL for ADA analysis at each blood sampling point. The serum should be separated.<br>For famitinib PK analysis, 3 mL of blood sample will be collected on D1 (± 3 d) of Cycle 3 at each of the following time points: within 30 min pre-administration, and at 2 h ± 5 min, 4 h ± 5 min, 6 h ± 5 min, 8 h ± 5 min, 10 h ± 5 min, and 24 h ± 30 min post-administration (before D2 administration), and the plasma will be separated.<br>➢ **Cohorts 7 and 8**:<br>For famitinib PK analysis, 3 mL of blood sample will be collected (only at sites with conditions for PK blood sampling and processing) from subjects of Cohorts 7 and 8 at each of the following time points: at 6 (± 1) h post-administration on C1D1, within 30 min pre-administration and at 6 (± 1) h post-administration on C2D1 and C3D1, and the plasma will be separated. If famitinib administration is interrupted on the day of sampling, pre-administration sampling should be continued but post-administration sampling should be skipped. On the sampling day, it is also necessary to record the previous administration time. |

| | |
|---|---|
| **Inclusion Criteria** | Subjects must meet all of the following criteria to be eligible for this study:<br>1. Voluntarily participate in this study and sign the informed consent form (ICF);<br>2. Male or female aged 18-75 years old;<br>3. Advanced renal cancer, urothelial carcinoma, cervical cancer, recurrent ovarian cancer, and endometrial cancer:<br>- Renal cell carcinoma: Histologically or cytologically confirmed advanced clear cell renal cell carcinoma (in the case of mixed tumors, predominant clear cell renal cell carcinoma is required);<br>1) Cohort 1: Have been previously treated with at most one targeted anti-angiogenic drug and failed the treatment (if any);<br>- Cohort 8: Progression after prior anti-PD-1/PD-L1/CTLA-4 antibody monotherapy or combination therapy (at least 2 doses of anti-PD-1/PD- L1/CTLA-4 antibody). Urothelial carcinoma: Histologically or cytologically confirmed unresectable urothelial carcinoma, including renal pelvis cancer, ureteric cancer, bladder cancer, and urethral cancer (in the case of mixed tumors, transitional cell carcinoma as the predominant histological subtype is required) that progressed or recurred after platinum-based therapies and have been treated with no more than 2 previous systemic treatments;<br>Cohorts 6 and 7: Have received at least one platinum-based therapy in the recurrence/metastasis stage, and the disease progressed or recurred.<br>Note: Neoadjuvant or adjuvant therapies, with recurrence and progression within 12 months after the end of treatment, are counted as one systemic treatment.<br>- Cervical cancer: Histologically or cytologically confirmed squamous cell carcinoma of the cervix; for recurrent/metastatic cervical cancer that has been treated with 1 or 2 lines of previous systemic treatment (except radiation-enhanced chemotherapy), recurrence or progression during or after previous treatment is required;<br>Note: Neoadjuvant or adjuvant therapies (except radiation-enhanced chemotherapy), with recurrence and progression within 1 year after first-line standard surgery, or within 6 months after radiotherapy, are included in the first- line systemic treatment.<br>- Recurrent ovarian cancer: Histopathologically confirmed recurrent epithelial ovarian cancer, fallopian tube cancer, or primary peritoneal cancer that has been treated with platinum-based therapy and recurred/progressed during or within 6 months after the last platinum-based therapy (completing 4 or more treatment cycles);<br>Note: No more than 1 non-platinum-based therapy between the last 2 platinum-based therapies; no other anti-tumor treatments except endocrine therapy, PARP inhibitor maintenance therapy, or traditional Chinese medicine and modern Chinese medicinal preparations after the last platinum-based therapy.<br>- Endometrial cancer: Histopathologically confirmed endometrial cancer (excluding carcinosarcoma) that recurred/metastasized after previous treatment; for recurrent/metastatic cancer that has been treated with 1 or 2 lines of previous systemic treatment (except radiation-enhanced chemotherapy), recurrence or progression during or after previous treatment is required. |

| | |
|---|---|
| | 4. At least one measurable lesion that meets RECIST v1.1 (must be ≥ 10 mm in long-axis diameter by spiral CT or ≥ 15 mm in short-axis diameter for enlarged lymph nodes, as per RECIST v1.1);<br>5. Able to swallow tablets;<br>6. ECOG PS score: 0-1 (refer to Appendix I for ECOG scoring criteria);<br>7. Life expectancy ≥ 12 weeks;<br>8. Major organ functions must meet the following requirements (No blood components or growth factor corrective therapy is allowed within 14 days prior to the start of study treatment):<br>• Absolute neutrophil count ≥ 1.5 × $10^9$/L;<br>• Platelets ≥ 90 × $10^9$/L;<br>• Hemoglobin ≥ 90 g/L;<br>• Serum albumin ≥ 30 g/L;<br>• Thyroid stimulating hormone (TSH) ≤ 1 × ULN (In case of abnormalities, FT3 and FT4 levels should be measured at the same time. If FT3 and FT4 levels are normal, the subject can be enrolled);<br>• Bilirubin ≤ 1 × ULN (within 7 days prior to the first dose);<br>• ALT and AST ≤ 3 × ULN (within 7 days prior to the first dose);<br>• AKP ≤ 2.5 × ULN (< 5 × ULN if accompanied by bone metastasis);<br>• Serum creatinine ≤ 1.25 × ULN;<br>9. Female patients of childbearing potential or female patients who are not sterilized by surgical operations are required to take two medically approved contraceptive measures (such as intrauterine device, oral contraceptive, or condom) during the study treatment period and within 3 months after the end of the study treatment; female patients of childbearing potential who are not surgically sterilized must have a negative serum HCG test result within 72 h prior to the first dose, and must not be on breast-feeding; male patients with partners of childbearing potential should take two effective contraceptive measures during the study and within 3 months after the end of study treatment. |
| **Exclusion Criteria** | Patients meeting any of the following are ineligible to participate in this study:<br>1. Any active autoimmune diseases or history of autoimmune diseases (including but not limited to the following: autoimmune hepatitis, interstitial pneumonia, uveitis, enteritis, hepatitis, hypophysitis, vasculitis, nephritis, and hyperthyroidism; vitiligo; adult patients with completely relieved childhood asthma can be enrolled if no intervention is required; patients with asthma requiring medical intervention with bronchodilators cannot be enrolled);<br>2. Currently using immunosuppressants, or systemic hormonal therapy for immunosuppression (> 10 mg/day of prednisone or an equivalent dose of other therapeutic hormones) within 2 weeks prior to the first dose;<br>3. Severe allergic reactions to other monoclonal antibodies;<br>4. Untreated metastases to central nervous system,<br>- Asymptomatic patients who have received prior systemic and radical treatment for metastases to the brain or meninges (radiotherapy or surgery) may be enrolled if they are stable for at least 1 month as confirmed by imaging and have stopped systemic hormonal therapy (> 10 mg/day of prednisone or equivalent) for more than 2 weeks; |

5. Hypertension not adequately controlled with antihypertensive therapy (systolic blood pressure ≥ 140 mmHg or diastolic blood pressure ≥ 90 mmHg);
6. Uncontrolled cardiac diseases or symptoms, such as: (1) NYHA Class II or above heart failure, (2) unstable angina, (3) myocardial infarction within the past year, (4) clinically significant supraventricular or ventricular arrhythmia requiring treatment or intervention, or (5) QTc > 450 ms (males) or QTc > 470 ms (females);
7. Abnormal coagulation function (INR > 2.0, PT > 16 s), bleeding tendency or receiving thrombolytics or anticoagulant therapy. Prophylactic use of low-dose aspirin or low molecular weight heparin is allowed;
8. Any Grade ≥ 2 bleeding as per CTCAE v4.03 within 4 weeks before the first dose;
9. Radiographically confirmed significant vascular invasions or a high possibility of significant vascular invasions that may cause fatal bleeding as determined by the investigators during treatment;
10. Events of arterial/venous thrombosis within 6 months prior to the first dose, such as cerebrovascular accidents (including transient ischemic attacks, cerebral hemorrhage, and brain infarction), deep vein thrombosis, and pulmonary embolism;
11. Known hereditary or acquired hemorrhage and thrombophilia (such as hemophilia, coagulopathy, and thrombocytopenia);
12. The routine urinalysis indicates that urine protein is ≥ ++ and confirms that 24 h urine protein is > 1.0 g;
13. Prior chemotherapy which ended (last dose) within 4 weeks prior to the start of this study treatment; prior surgery or palliative radiotherapy within 2 weeks prior to study treatment; molecular targeted therapy (including oral targeted drugs in other clinical trials) within < 5 drug half-lives from the first study dose; or AEs caused by previous treatment (except for alopecia) that have not returned to CTCAE Grade ≤ 1;
14. Radiation-induced enteritis after receiving pelvic radiotherapy within 12 months prior to the study treatment;
15. Active infection, unexplained fever ≥ 38.5 °C within 7 days prior to the first study dose, or baseline white blood cell count > 15 × $10^9$/L;
16. Known history or evidence of interstitial lung disease or non-infectious pneumonitis that has been treated with corticosteroids; or conditions that may interfere with the testing or management of suspected drug-related pulmonary toxicity;
17. Congenital or acquired immunodeficiency (such as HIV positive);
18. Active hepatitis (hepatitis B: positive HBsAg and HBV DNA ≥ 500 IU/ mL; hepatitis C: positive HCV antibody and HCV virus copy number > upper limit of normal);
19. Other malignancies currently or within the past 5 years (except for cured basal cell carcinoma and cervical cancer *in situ*; for recurrent ovarian cancer previously accompanied by breast cancer, patients with no breast cancer recurrence for > 3 years after radical mastectomy can be included);
20. Prior treatment with anti-PD-1/PD-L1 antibodies (except for cervical cancer in Cohort 7 and Cohort 8) or famitinib;
21. Have received live vaccines within 4 weeks before the first dose or may possibly receive live vaccines during the study;
22. Other potential factors that may affect the study results or result in premature discontinuation as determined by the investigators, such as alcoholism, drug abuse, other serious diseases (including mental illness) requiring concomitant treatment, serious laboratory abnormalities, or family or social factors that could affect the safety of the patients.

| | |
|---|---|
| **Criteria for Discontinuation** | A subject must discontinue the treatment when any of the following conditions occurs:<br>1. Subject requests for discontinuation or withdraws the ICF;<br>2. Imaging examinations show PD;<br>As per RECIST v1.1, a confirmation is required 4-6 weeks after the first documentation of PD (except those with rapid progression, with significant clinical progression, or receiving famitinib monotherapy);<br>Subjects with confirmed PD may continue the treatment if clinically stable (as assessed by the investigators) until further radiographic progression;<br>Definition of clinically stable: a. no significant clinical symptoms or changes in laboratory tests; b. no changes in the performance status score (deterioration); c. no rapid tumor progression and no progression involving vital organs/sites (e.g., spinal cord compression);<br>3. Accumulated use of SHR-1210 monotherapy or combination therapy for 2 years (no radiographic progression). Subjects who achieve radiographically confirmed CR may consider discontinuation after 12 cycles of treatment;<br>4. Unacceptable toxicity;<br>5. Poor compliance;<br>6. Loss to follow-up or pregnancy;<br>7. Other reasons for which the investigators consider a withdrawal necessary. |
| **Study Withdrawal Criteria** | Reasons for withdrawal may include:<br>1. Subject withdraws the ICF and refuses further follow-ups;<br>2. Other investigator-assessed reasons requiring withdrawal, such as the inability to provide voluntary consent due to imprisonment or quarantine;<br>3. Loss to follow-up or death;<br>4. Study termination by the sponsor. |
| **Sample Size Determination** | ➢ **Cohorts 1-5**:<br>This study adopts Lin & Shih's two-stage adaptive design for Cohorts 1-5, with an assumed unacceptable objective response rate (ORR) of $p_0$, low target ORR of $p_1$, and high target ORR of $p_2$, two-sided $\alpha = 0.1$, $\beta_1 = 0.3$, $\beta_2 = 0.2$, $p_0 = 0.15$, $p_1 = 0.25$, and $p_2 = 0.35$.<br>Twenty-two subjects will be enrolled in Stage I; if ≥ 7 subjects achieve response, additional subjects should be enrolled in Stage II to make up a sample size of 33, if ≥ 8 subjects achieve response, move on to the next study stage;<br>If response is observed in 3 to 6 out of the 22 enrolled subjects in Stage I; additional subjects should be enrolled in Stage II to make up a sample size of 53, if ≥ 12 subjects achieve response, move on to the next study stage;<br>Otherwise (response observed in < 3 out of 22 enrolled subjects), terminate the study in the corresponding cohort.<br>When the 33-53 subjects in a certain cohort complete the efficacy observations in Stage II, whether to expand the sample size for this cohort (tumor type) will be determined on the basis of efficacy results.<br>➢ **Cohorts 6-7:**<br>The Simon's (minimax) two-stage design is adopted to calculate the sample size for each tumor type of Cohorts 6 and 7 based on one-sided alpha = 0.1 and power = 0.7: |

**Cohorts 6-7: Calculation of sample size by Simon's (minimax) two-stage method (one-sided alpha = 0.1, power = 0.7)**

| Cohort and Tumor Type | $P_0$ | $P_1$ | Stage I ($r_1/n_1$) | Stage II (r/n) |
|---|---|---|---|---|
| **Cohort 6** Urothelial Carcinoma - SHR-1210 Monotherapy Group | 0.10 | 0.25 | 1/14 | 4/23 |
| **Cohort 7** Urothelial Carcinoma - Famitinib Monotherapy Group | 0.10 | 0.25 | 1/14 | 4/23 |
| **Cohort 7** Cervical Cancer - Famitinib Monotherapy Group | 0.10 | 0.25 | 1/14 | 4/23 |

Note: $P_0$ is the maximum futility boundary, and $P_1$ is the minimum efficacy boundary. $n_1$ is the sample size of Stage I, n is the total sample size of the two stages, $r_1$ is the critical value of CR or PR that needs to be observed in Stage I (not inclusive), and r is the critical value of CR or PR that needs to be observed in the two stages (not inclusive). If, in Stage I, less than or equal to $r_1$ subjects among the $n_1$ subjects achieve CR or PR, the cohort will be terminated; otherwise n - $n_1$ subjects will be additionally enrolled in Stage II.

- **Cohort 8:**

The Simon's (minimax) two-stage design is adopted to calculate the sample size of Cohort 8 based on one-sided alpha = 0.025 and power = 0.8:

**Cohort 8: Calculation of sample size by Simon's (minimax) two-stage method (one-sided alpha = 0.025, power = 0.8)**

| Cohort and Tumor Type | $P_0$ | $P_1$ | Stage I ($r_1/n_1$) | Stage II (r/n) |
|---|---|---|---|---|
| **Cohort 8** Renal Cell Carcinoma | 0.30 | 0.50 | 6/21 | 20/47 |

Note: $P_0$ is the maximum futility boundary, and $P_1$ is the minimum efficacy boundary. $n_1$ is the sample size of Stage I, n is the total sample size of the two stages, $r_1$ is the critical value of CR or PR that needs to be observed in Stage I (not inclusive), and r is the critical value of CR or PR that needs to be observed in the two stages (not inclusive). If, in Stage I, less than or equal to $r_1$ subjects among the $n_1$ subjects achieve CR or PR, the cohort will be terminated; otherwise n - $n_1$ subjects will be additionally enrolled in Stage II.

**Data Analysis/Statistical Methods**

**General analysis:**

Unless otherwise stated, all study data will be summarized with descriptive statistics using the following methods.

Continuous variables will be summarized by mean, standard deviation, median, maximum, and minimum; categorical variables will be summarized by frequency and percentage;

For time-to-event data, the survival rate and median survival will be estimated using the Kaplan-Meier method. A corresponding 95% confidence interval will be provided for the aforementioned, when necessary.

**Efficacy endpoint analysis:**

The primary efficacy endpoint is investigator-assessed objective response rate (ORR) of each tumor type in each cohort as per RECIST v1.1. The number of subjects with objective response, ORR, and the corresponding Clopper-Pearson 95% confidence interval will be provided for each cohort. The analysis of DCR is the same as that of ORR. For efficacy endpoints (DOR, PFS, and OS), the medians will be estimated using the Kaplan-Meier (KM) product-limit method. The 95% CIs for median will be estimated by Brookmeyer-Crowley method, and survival

| | |
|---|---|
| | curves will be plotted; TTR will be summarized by mean, median, standard deviation, minimum, maximum, and quartiles (Q1 and Q3).<br>**Safety analysis:**<br>All adverse events (AEs) will be coded according to MedDRA and graded using NCI-CTCAE v4.03. AE analysis will mainly focus on treatment-emergent adverse events (TEAEs), i.e., AEs observed on the day of the first study dose or thereafter. Non-treatment-emergent AEs (observed prior to first study dose) will only be listed.<br>Incidence, type, and severity of AEs in each cohort will be summarized by system organ class (SOC) and/or preferred term (PT) according to MedDRA.<br>AEs leading to treatment interruption, AEs leading to treatment discontinuation, SAEs, CTCAE Grade ≥ 3 AEs, and treatment-related AEs and SAEs in each cohort will be summarized by system organ class (SOC) and preferred term (PT). Special interest events (SIEs) will be summarized. AEs leading to death will be summarized and listed.<br>Laboratory tests, hematology, blood biochemistry, and other indicators will be summarized by shift table to describe normal changes, abnormal changes without clinical significance, and abnormal changes with clinical significance after treatment, and will be classified using the most severe grades after treatment. Where applicable, laboratory measurements will be summarized by cross-tabulation according to CTCAE grade (baseline level and highest severity post-baseline).<br>Vital signs (blood pressure, temperature, pulse, and respiratory rate), ECG (heart rate, PR interval, QRS interval, QT interval, and QTc interval), and other indicators will be summarized by mean, median, and range (minimum and maximum values) by visit. Changes from baseline in ECG indicators will be summarized descriptively, and cross-tabulated for changes to normal and abnormal after treatment. Concomitant medications will be summarized by frequency and percentages.<br>**Pharmacokinetic analysis:**<br>The PK parameters of famitinib in humans will be calculated using the non- compartmental model (if applicable) and the plasma concentrations will be summarized. The serum concentrations/PK parameters of SHR-1210 in humans will be summarized (if applicable).<br>From the blood concentration-time data, the PK parameters ($C_{max}$, $T_{max}$, $AUC_{0-t}$, $t_{1/2\beta}$, CLs, Vd, and MRT) will be descriptively summarized (by n, mean, standard deviation, median, minimum, maximum, geometric mean and geometric standard deviation, and coefficient of variation (%CV)) and tabulated based on different investigational products and scheduled blood sampling time points. Mean and/or median blood concentration-time curves (linear and semi-logarithmic) of different investigational products will be plotted based on scheduled blood sampling time points. Blood concentration-time curves of individual subjects and/or overlays of individual subjects in each group (linear and semi-logarithmic) will be plotted.<br>**Exploratory analysis:**<br>The proportion of PD-L1-positive cells in tumor tissue (for subjects receiving SHR-1210 combination therapy or monotherapy only), proportion of subjects with dMMR or MSI-H (for endometrial cancer and ovarian cancer only), and proportion of abnormal FGFR2/3 (for urothelial carcinoma treated with combination therapy only) will be evaluated to explore the relationship of PD-L1 expression and/or other biomarkers with efficacy (such as ORR/PFS). |
| **Study Dates** | Anticipated enrollment of the first subject: Oct. 2018<br>Anticipated enrollment of the last subject: Jun. 2021 |

| | |
|---|---|
| | Anticipated study completion: Dec. 2021 |

# SCHEDULE OF ACTIVITIES

| Item | Screening Period[29] | | | Treatment Period (21 days/cycle) | | | Post-Treatment | |
|---|---|---|---|---|---|---|---|---|
| | Within 3 week before administration | Within 1 week before administration | Cycle 1 | | Cycle 2 | Cycles 3-35 | | |
| | | | Day 1 | Day 7 (± 1 d) | Day 1 (± 3 d) | Day 1 (± 3 d) | End of treatment/ withdrawal (-14 d/+3 d) | End-of-treatment visit (± 3 d) |
| Baseline Data | | | | | | | | |
| Signing of Informed Consent | × | | | | | | | |
| Demographics | × | | | | | | | |
| Tumor History | × | | | | | | | |
| Medical History[1] | | | | | | | | |
| Concomitant Medication[2] | × | × | × | | | | | |
| Laboratory Test | | | | | | | | |
| Hematology[3] | | × | | × | × | × | × | × |
| Blood Biochemistry[4] | | × | | × | × | × | × | × |
| Urinalysis[5] | | × | | × | × | × | × | × |
| Routine Stool Test[6] | | × | | | × | | × | |
| Coagulation Function Test[7] | | × | | × | × | × | × | |
| Thyroid Function Test[8] | × | | | | × | Once every 3 cycles | × | |
| Hepatitis B and Hepatitis C Tests[9] | × | | | | | | | |
| Pregnancy Test[10] | | × | | | | | × | |
| Myocardial Zymography[11] | | × | | | | | × | |
| Pituitary Adrenal Axis Test[12] | × | | | | | | | |
| HIV Test | × | | | | | | | |

| Item | Screening Period[29] | | | Treatment Period (21 days/cycle) | | | Post-Treatment | |
|---|---|---|---|---|---|---|---|---|
| | Within 3 week before administration | Within 1 week before administration | Cycle 1 | | Cycle 2 | Cycles 3-35 | | |
| | | | Day 1 | Day 7 (± 1 d) | Day 1 (± 3 d) | Day 1 (± 3 d) | End of treatment/ withdrawal (-14 d/+3 d) | End-of-treatment visit (± 3 d) |
| **Clinical Evaluation and Examination** | | | | | | | | |
| Adverse Events[13] | **From informed consent to 30 days after the last dose** | | | | | | | |
| Vital Signs[14] | | × | × | × | × | × | × | × |
| Physical Examination and Weight Measurement[15] | | × | × | × | × | × | × | × |
| ECOG PS Score | | × | | | × | × | × | × |
| ECG[16] | | × | | × | × | × | × | |
| Echocardiography[17] | | × | | | | | × | |
| Blood Pressure Monitoring[18] | | × | | × | × | × | × | |
| B-Mode Ultrasonography of Lower Extremity Veins[19] | | × | | | | | × | |
| **Investigational Products** | | | | | | | | |
| SHR-1210 Administration[20] | | | × | | × | × | | |
| Famitinib Administration[21] | | | **Oral administration once a day before or after a meal** | | | | | |
| Dispensation/Return of Famitinib[22] | | | × | | × | × | × | |
| **Imaging Evaluation** | | | | | | | | |
| Imaging Examination[23] | × | | **Once every 3 cycles** | | | | × | |
| **Follow-Up After End of Treatment** | | | | | | | | |
| Time to Progression[24] | | | | | | | Imaging evaluations are performed once every 3 months (± 7 d) until PD or start of new anti-tumor treatments (for subjects with non-radiographic PD) | |

| **Item** | **Screening Period[29]** | | | **Treatment Period (21 days/cycle)** | | | **Post-Treatment** | |
|---|---|---|---|---|---|---|---|---|
| | Within 3 week before administration | Within 1 week before administration | **Cycle 1** | | **Cycle 2** | **Cycles 3-35** | | |
| | | | Day 1 | Day 7 (± 1 d) | Day 1 (± 3 d) | Day 1 (± 3 d) | End of treatment/ withdrawal (-14 d/+3 d) | End-of-treatment visit (± 3 d) |
| Death[25] | | | | | | | | × |
| Blood Sampling and Tumor Sample Acquisition/Collection | | | | | | | | |
| Sampling for Biomarkers[26] | × | | | | | | | |
| Blood Sampling for SHR-1210[27] | | | × | | × | × **(From Cycle 4 onwards, collect blood samples before administration once every 12 weeks)** | | |
| Blood Sampling for Famitinib[28] | | | × | | × | × | | |

**Notes: All examinations and trial procedures are carried out according to the time specified in the Schedule of Activities and are not affected by the length of treatment interruption. However, occasional changes outside the allowable window are permitted due to holidays or other management reasons.**

**The safety and imaging visits for subjects who receive continuing treatment after PD in Cohorts 6 and 7 are the same as those before PD.**

[1] Tumor history/medical history: tumor diagnosis, surgical history, history of local treatment, systemic treatment, radiotherapy, and other treatments for past diseases, as well as history of other tumors.

[2] Concomitant medications and treatments received within 30 days prior to the first dose and during the study period should be recorded. Once a subject discontinues the study treatment, only concomitant medications or treatments for new or unresolved treatment-related AEs are recorded, until 30 days after the last dose.

[3] Hematology: white blood cells, neutrophils, lymphocytes, red blood cells, hemoglobin, and platelets. The test should be performed within 7 days prior to enrollment, on Day 7 of Cycle 1, on Day 1 of subsequent cycles, at the end of treatment or upon withdrawal from study, and at 30 days after the end of treatment.

[4] Blood biochemistry: total bilirubin (TBIL), direct bilirubin, ALT, AST, AKP, γ-GT, LDH, total protein, albumin, urea/blood urea nitrogen, creatinine, uric acid, fasting blood glucose, triglyceride, cholesterol, potassium, sodium, chlorine, calcium, phosphorus, blood lipase (only during the screening period and in case of subsequent abdominal pain, abdominal distension, and other symptoms of suspected pancreatitis), and blood amylase (only during the screening period and in case of subsequent abdominal pain, abdominal distension, and other symptoms of suspected pancreatitis). The test should be performed within 7 days prior to enrollment, on Day 7 of Cycle 1, on Day 1 of subsequent cycles, at the end of treatment or upon withdrawal from study, and at 30 days after the end of treatment.

[5] Urinalysis: urine protein, urine glucose, urine occult blood, urine red blood cells, and urine white blood cells. During the screening period, if semi- quantitative test shows urine protein of ≥ 2+, a quantitative 24-h urine protein test is required. Subsequently, if semi-quantitative tests from 2 consecutive follow-ups show urine protein of 2+, a quantitative 24-h urine protein test is required, and if semi-quantitative test shows protein of > 2+, a quantitative 24-h urine protein test should be performed. The test should be performed within 7 days prior to enrollment, on Day 7 of Cycle 1, on Day 1 of subsequent cycles, at the end of treatment or upon withdrawal from study, and at 30 days after the end of treatment.

[6] Routine stool test: Patients with positive fecal occult blood must be retested. If fecal occult blood is confirmed, based on the clinical judgment, a gastroscopy should be performed when necessary. The test should be performed within 7 days prior to enrollment, on Day 1 of Cycle 2, at the end of treatment or upon withdrawal from study, and as judged by the investigators during treatment. If fecal occult blood occurs during treatment, the subject should undergo a gastroscopy if deemed necessary by the investigators.

[7] Coagulation function: INR, APTT, PT, and FIB. The test should be performed within 7 days before enrollment, on Day 7 of Cycle 1 and Day 1 of subsequent cycles, and at the end of treatment or upon withdrawal from study.

[8] Thyroid function test: serum FT3, FT4, and TSH. The test should be performed within 21 days before administration, on Day 1 of Cycle 2, once every 3 cycles thereafter, and at the end of treatment or upon withdrawal from study, with a window period of ± 7 d.

[9] Hepatitis B and hepatitis C tests: Subjects with abnormal HBsAg results should undergo quantitative test of HBV DNA. Subjects with positive anti-HCV antibodies must be tested for HCV titer (HCV RNA).

[10] Pregnancy test: A blood pregnancy test should be performed on women of childbearing potential within 72 h prior to the first dose. Additional tests may be performed to rule out pregnancy if indicated. By considering the subjects' condition, the test can be performed as needed and at the end of treatment or upon withdrawal from study.

[11] Myocardial zymography: creatine kinase and lactate dehydrogenase. The test should be performed within 7 days prior to enrollment, when deemed necessary by the investigators, and at the end of treatment/upon withdrawal.

[12] Pituitary adrenal axis test: including adrenocorticotropic hormone (ACTH), cortisol, and follicle stimulating hormone. The test should be performed within 21 days before administration.

[13] Adverse events: AEs should be recorded from the signing of informed consent until at least 30 days after the last dose. All AEs suspected to be related to SHR-1210 should be collected from 30 to 90 days after the last dose. All AEs must be followed up until the event resolves, returns to the baseline level, reaches a stable disease, or returns to Grade ≤ 1, loss to follow-up or death. See Section 8.2.6 for details.

[14] Vital signs: body temperature, pulse, respiratory rate, and blood pressure. The measurement should be performed within 7 days prior to enrollment, on Day 1 and Day 7 of Cycle 1, on Day 1 of subsequent cycles, at the end of treatment or upon withdrawal from study, and at 30 days after treatment completion.

[15] Physical examination: Examination of major body systems (head and face, skin system, lymph nodes, eyes, ears, nose, throat, oral cavity, respiratory system, cardiovascular system, abdomen, genitourinary system, musculoskeletal system, nervous system, and mental state) should be performed within 7 days prior to enrollment, on Day 7 of Cycle 1, Day 1 of subsequent cycles, at the end of treatment or upon withdrawal from study, and at 30 days after treatment completion.

Note: Except for baseline examinations, only abnormal findings are documented in the eCRF rather than the entire physical examination (although a comprehensive physical examination is still required).

Weight measurement: The measurement should be performed on Day 1 of Cycle 1, on Day 1 of subsequent cycles, at the end of treatment or upon withdrawal from study, and at 30 days after the end of treatment.

[16] 12-lead ECG: The examination should be performed within 7 days prior to enrollment, on Day 7 of Cycle 1, on Day 1 of subsequent cycles, and at the end of treatment or upon withdrawal from study. ECG examination within 7 days before the first dose in the screening period will be performed for 3 consecutive times at an interval of about 5 min. The average value of the 3 QTc results is taken. The average value of the 3 QTc results of the enrolled subject must meet the inclusion and exclusion criteria. If the ECG is abnormal (with clinical significance) during the study, another two examinations must be performed.

[17] Echocardiography: Echocardiography should be performed once within 7 days prior to enrollment and at the end of treatment or upon withdrawal from study, and when clinically significant ECG abnormalities are found during the study.

[18] Blood pressure monitoring: The blood pressure of patients will be measured by the investigators or study nurse in the screening period. At each blood pressure measurement, smoking and coffee are prohibited within 30 min before measurement, and patients should rest for at least 10 min. The sitting position will be taken at measurement by placing the elbow at the same level as the heart. Each blood pressure measurement should be taken on the same side of the body. The blood pressure must be under 140/90 mmHg (average of 2 blood pressure measurements taken at least 24 h apart) before the first dose. Blood pressure will be measured by subjects themselves during the study (subjects in Cohort 6 are not required to measure blood pressure at home) and recorded in their diary cards. Blood pressure should be measured at least 3 times a week in the first 2 cycles. If blood pressure is abnormal, the measurement should be carried out every day; if blood pressure is normal, the measurement should be carried out at least twice a week after 2 cycles. In addition, blood pressure will be measured by the investigators or study nurse at each follow-up.

[19] B-mode ultrasonography of lower extremity veins: B-mode ultrasonography of the deep veins of both lower extremities should be performed within 7 days prior to enrollment and at the end of treatment or upon withdrawal from study. The examination may be performed as necessary based on the investigators' clinical judgment.

[20] SHR-1210 administration to subjects in Cohorts 1-6 and 8 and to subjects who continue treatment after PD in Cohort 7: SHR-1210 will be administered via intravenous infusion (premedication not required) at a fixed dose of 200 mg within 30 min (not less than 20 min, not more than 60 min), once every 3 weeks. Each cycle contains 3 weeks and the longest dosing period is 2 years (up to 35 administrations). After 12 cycles, the SHR-1210 administration can be stopped if the imaging examination confirms the subjects as CR.

[21] Famitinib should be administered orally once a day before or after a meal (recommended at a fixed time: within 0.5 h after a meal) at a dose of 20 mg (for subjects in Cohorts 1-5 and 8 and for subjects who continue treatment after PD in Cohort 6) or 25 mg (for Cohort 7). The drug should be administered continuously in cycles of 3 weeks. In Cohort 7, the dose and frequency of famitinib for subjects who continue treatment after PD are the same as those before PD. If the dose of famitinib before PD is 25 mg, the dose of famitinib should be adjusted to 20 mg for continuing treatment. The drug should be administered until PD, unacceptable toxicity, or withdrawal from the treatment by the subjects or the investigators. Subjects in Cohorts 7 and 8 who participate in the blood sampling for famitinib PK analysis should take famitinib after blood sampling before C2D1 and C3D1 administration.

[22] Dispensation/return of famitinib: Famitinib should be dispensed on Day 1 of Cycle 1, and subsequently dispensed and returned on Day 1 of every cycle thereafter. The remaining drugs should be retrieved to verify the actual dose administered before new drugs are dispensed.

[23] Imaging examination: including contrast-enhanced CT or MRI of the chest, abdomen, and pelvis. A contrast-enhanced MRI or CT of the brain must also be performed for those suspected of brain metastasis to rule out brain metastasis. Tumor evaluation performed within 3 weeks prior to treatment can be accepted as the baseline evaluation. CT/MRI results prior to informed consent may be used for tumor evaluation at screening if requirements are met. Patients with evident or suspected bone metastasis must undergo a bone scan and those with bone lesions need to undergo CT/MRI for confirmation. Imaging examinations in the treatment period should be performed using the same evaluation method as that at baseline every 3 cycles under the same conditions as those of the baseline examination (slice thickness, use of contrast agent, etc.) for efficacy evaluation. Brain imaging evaluation is not required at all time if no brain metastasis is confirmed at baseline. Bone scan is performed for suspected bone progression or CR confirmation (for those with bone metastases at baseline); new suspected lesions may be timely examined as appropriate. The first documentation of PR/CR in a subject must be confirmed 4 weeks +7 days later. A confirmation is required 4-6 weeks after the first documentation of PD as per RECIST v1.1 (except those with rapid progression or significant clinical progression).

The window period for imaging examination schedule is ± 7 days. Unscheduled imaging examinations can be performed when PD is suspected (such as worsening of symptoms).

[24] In addition to the PD as evidenced by imaging, subjects who have discontinued the study treatment for other reasons must be evaluated at the end of treatment if the imaging evaluation is not performed within 4 weeks prior to the end of treatment. Also, tumor efficacy should be followed up every 3 months (± 7 d) after the end of treatment until records confirm PD or initiation of new anti-tumor treatment.

[25] Survival follow-up: After the study treatment is discontinued, the survival status and subsequent anti-tumor treatment can be collected through clinical or telephone follow-ups every 2 months (± 7 d) until death.

[26] Existing paraffin-embedded tumor tissue sections will be collected, and 5 tumor sections with a thickness of 4-5 μm will be used for PD-L1 detection (except for Cohort 7). It is recommended that fresh biopsy specimens (core needle biopsy) be collected before the first dose. After fixation and embedding, 3 tumor sections with a thickness of 4-5 μm will be prepared for PD-L1 detection.

For endometrial cancer and ovarian cancer, no less than 5 additional sections with a thickness of 4-5 μm and 5 mL of whole blood should be collected for the detection of MSI. For urothelial carcinoma (except for Cohorts 6 and 7), no less than 5 additional sections with a thickness of 4-5 μm should be collected for the detection of FGFR2/3 gene abnormality. Refer to the laboratory manual for tumor biosample acquisition/collection, transportation, and processing methods.

[27] Blood sampling for SHR-1210 in the first 12 subjects enrolled in Cohorts 1-5: Blood samples will be collected within 30 min before administration and within 5 min after administration (including flushing) of the first SHR-1210 dose; within 30 min before administration and within 5 min after administration (including flushing) of SHR-1210 on administration days of Cycles 2, 3, and 4; within 30 min pre-administration every 4 cycles (12 weeks) thereafter; at the end of SHR-1210 treatment (the documented time of last SHR-1210 administration prior to the end of study); at 30 days after the end of SHR-1210 treatment. (If subject completes the treatment before scheduled blood sampling is completed, the analysis should be based on actual completed sampling). ADA blood samples will be simultaneously collected along with PK samples before each administration, at the end of treatment, and at 30 days after the end of treatment. For SHR-1210, 4 mL should be collected for PK analysis and 4 mL for ADA analysis at each blood sampling point. The serum should be separated.

[28] For famitinib PK analysis, 3 mL of blood sample will be collected from the first 12 subjects enrolled in Cohorts 1-5 on D1 (± 3 d) of Cycle 3 at each of the following time points: within 30 min pre-administration, and at 2 h ± 5 min, 4 h ± 5 min, 6 h ± 5 min, 8 h ± 5 min, 10 h ± 5 min, and 24 h ± 30 min post-administration (before D2 administration), and the plasma will be separated. For famitinib PK analysis, 3 mL of blood sample will be collected (only at sites with conditions for PK blood sampling and processing) from subjects of Cohorts 7 and 8 at each of the following time points: at 6 (± 1) h post- administration on C1D1, within 30 min pre-administration and at 6 (± 1) h post-administration on C2D1 and C3D1, and the plasma will be separated. If famitinib administration is interrupted on the day of sampling, pre-administration sampling should be continued but post-administration sampling should be skipped. On the sampling day, it is also necessary to record the previous administration time.

[29] Additional eligible subjects with urothelial carcinoma (subjects not enrolled in Cohort 2) will be randomized to Cohort 6 or 7 to receive the corresponding investigational products after screening.

## Schedule of sampling from the first 12 subjects enrolled in Cohorts 1-5 for SHR-1210 PK and ADA analysis

| C1D1 | C2D1 | C3D1 | C4D1 | Every 12 Weeks | End of Treatment | 30 Days After End of Treatment |
|---|---|---|---|---|---|---|
| Within 30 min pre- administration | Within 30 min pre-administration | Within 30 min pre- administration | Within 30 min pre-administration | Within 30 min pre-administration | Once | Once |
| Within 5 min post- administration (including flushing) | Within 5 min post- administration (including flushing) | Within 5 min post- administration (including flushing) | Within 5 min post- administration (including flushing) | —— | | |

4 mL of blood will be collected for PK and 4 mL for ADA at each sampling time point, and the serum will be separated.

## Schedule of sampling from the first 12 subjects enrolled in Cohorts 1-5 for famitinib PK analysis

| C3D1 | | | | | | | |
|---|---|---|---|---|---|---|---|
| Pre-Administration | Within 30 min | —— | —— | —— | —— | —— | |
| Post-Administration | 2 h ± 5 min | 4 h ± 5 min | 6 h ± 5 min | 8 h ± 5 min | 10 h ± 5 min | 24 h ± 30 min | |

3 mL of blood will be collected at each sampling time point, and the plasma will be separated.

## Schedule of sampling from subjects in Cohorts 7 and 8 for famitinib PK analysis

| C1D1 | C2D1 | C3D1 |
|---|---|---|
| —— | Within 30 min pre-administration | Within 30 min pre-administration |
| 6 (± 1) h post-administration | 6 (± 1) h post-administration | 6 (± 1) h post-administration |

3 mL of blood will be collected at each sampling time point, and the plasma will be separated. If famitinib administration is interrupted on the day of sampling, pre-administration sampling should be continued but post-administration sampling should be skipped. On the sampling day, it is also necessary to record the previous administration time.

# ABBREVIATIONS

| Abbreviation | Full Name |
|---|---|
| 12-Lead ECG | 12-Lead electrocardiogram |
| ADA | Anti-drug antibody |
| AE | Adverse event |
| ALP | Alkaline phosphatase |
| ALT | Alanine aminotransferase |
| ANOVA | Analysis of variance |
| AST | Aspartate aminotransferase |
| BUN | Blood urea nitrogen |
| CK | Creatine kinase |
| CK-MB | Creatine kinase-MB |
| $Cl^-$ | Blood chlorine |
| Cr | Creatinine |
| CRF | Case report form |
| CTC | Circulating tumor cells |
| D | Day |
| DLT | Dose-limiting toxicity |
| DoR | Duration of response |
| EC | Ethics committee |
| GCP | Good Clinical Practice |
| GLU | Blood glucose |
| GLU-U | Uglu urine glucose |
| h | Hour |
| Hb | Hemoglobin |
| HDL-C | High-density lipoproteincholesterol |
| IB | Investigator’s brochure |
| $IC_{50}$ | Half maximal inhibitory concentration |
| irAE | Immune related adverse event |
| LEU | Leukocytes in urine |
| IU | International unit |
| $K^+$ | Serum potassium |
| KET | Urine acetone bodies |
| LDH | Lactate dehydrogenase |
| LDL-C | Low-density lipoprotein cholesterol |
| LYMPH | Lymphocyte |
| MMR | Mismatch repair |
| MTD | Maximum tolerated dose |
| $Na^+$ | Plasma sodium |
| NCT-CTCAE | National Cancer Institute-Common Terminology Criteria for Adverse Events |
| NEUT | Neutrophil |
| ORR | Objective Response Rate |
| PD-1 | Programmed death receptor 1 |

| Abbreviation | Full Name |
|---|---|
| PD-L1 | Programmed death ligand 1 |
| PFS | Progression-Free Survival |
| PLT | Blood platelet |
| PRO | Protein in urine |
| RBC | Red blood cell count |
| SAE | Serious adverse event |
| SAP | Statistical analysis plan |
| SIE | Special interest event |
| sUA | Serum uric acid |
| T-BIL | Total bilirubin |
| TC | Total cholesterol |
| TG | Triglyceride |
| TG | Triglyceride |
| TMB | Tumor Mutation Burden |
| TTR | Time to response |
| UA | Uric acid |
| UBIL | Urine bilirubin |
| URBC | Urine red blood cell |
| WBC | White blood cell count |

# 1. INTRODUCTION: BACKGROUND AND SCIENTIFIC RATIONALE

## 1.1. Background

In recent years, significant progress has been made in cancer treatment, and small molecular targeted therapies inhibiting internal driving factors of tumor angiogenesis and cancer cell growth, as well as immunotherapies enhancing anti-cancer immunity of the patients, have been approved by regulatory authorities in many countries. With the deepening of the understanding of the body's immune system and the rapid development of biotechnology, the immunotherapy has become an important means of cancer treatment, and it is occupying an increasingly important position in the comprehensive treatment system of tumors.

Targeted therapy may result in significant clinical response for numerous types of tumors. However, the duration of response is short, and the initial responses are usually accompanied by tumor immune escape and clinical recurrence within a few months. In comparison, immunotherapy is also applicable to many types of cancers, and can provide a longer duration of response in some patients. Contrary to standard chemotherapy or targeted therapy which directly acts on the cancer cells, immunotherapy not only kills cancer cells directly, but more importantly, also enhances the immune response of the body by acting on the immune system and ultimately prolonging the survival of patient.

Programmed cell death protein 1, or PD-1, is a negative costimulatory molecule discovered in recent years. PD-L1 and PD-L2 are the ligands of PD-1 and specifically bind to PD-1. Through high expression of PD-L1, tumor cells bind to PD-1 on T lymphocytes and transmit negative regulatory signals, leading to apoptosis and immune incompetence of tumor antigen-specific T cells and allowing tumor cells to escape the monitoring and killing of the body's immune system. PD-1 inhibitors are new immunotherapeutic drugs of interest. By blocking the PD-1/ PD-L1 signaling pathway, the anti-tumor activity of T lymphocytes is up-regulated, resulting in apoptosis of the cancer cells.

Since 2014, based on breakthrough efficacy, the U.S. Food and Drug Administration (FDA) has successively approved anti-PD-1 monoclonal antibodies, nivolumab from BMS and pembrolizumab from Merck for the treatment of patients with advanced melanoma, non-small cell lung cancer, renal cell carcinoma, head and neck cancer, Hodgkin's lymphoma, hepatocellular carcinoma previously treated with sorafenib, as well as PD-L1-positive cervical cancer, including those with advanced disease, refractory to standard treatment, or without effective treatment options. The above drugs are also used as first- or second-line treatment currently. In addition, due to its long-lasting efficacy and relatively mild adverse reactions, anti- PD-1 monoclonal antibodies have been studied in hundreds of clinical trials internationally for advanced solid tumors and malignant hematologic diseases, including monotherapy and combination therapy. Results have all shown better efficacy and longer survival compared to existing treatments.

Renal cell carcinoma (RCC) is one of the most malignant and most common tumors of the urinary system. The incidence of renal cancer in China ranks second among the cancers of the genitourinary system, second only to bladder cancer. Prior to the approval of nivolumab, targeted therapy was the most commonly used treatment for patients with advanced RCC, and the mTOR inhibitor temsirolimus, approved in 2007, was the only marketed drug that has showed an advantage in OS in randomized, controlled trials. On 23 Sep., 2015, the FDA approved nivolumab for the treatment of previously treated advanced renal cell carcinoma (RCC). This new indication was approved based on results from a randomized, controlled clinical trial (Checkmate-025) enrolling 821 patients with advanced renal cell carcinoma that had progressed after prior anti-angiogenic therapy. Survival was significantly improved in the nivolumab group compared with the everolimus control group (median OS: 25 months vs. 19.6 months; HR, 0.73; $P = 0.002$). The OS benefit was independent of PD-L1 expression condition. In addition, the nivolumab group showed a significant advantage in duration of response compared with the everolimus control group (ORR: 21.5% vs. 3.9%; median DOR: 23.0 months vs. 13.7 months).

Bladder cancer is the most common malignant tumor of the urinary system and the ninth most common cancer worldwide. The incidence of bladder cancer ranks first among the cancers of the genitourinary system in China and ranks second in western countries, second only to prostatic cancer. Urothelial carcinoma is the most common type of bladder cancer, accounting for about 90% of all bladder cancers. Most bladder cancers can be diagnosed at their early stage, but the recurrence and progression rates are high, relapsing in about 78% of patients within 5 years, with a 5-year survival rate of only 15% for stage IV bladder cancer. Previously, platinum-based chemotherapy was the preferred first-line treatment for metastatic or unresectable bladder cancer, with a response rate of 40%-65%. There is currently no clear standard second-line treatment for patients with progression after first-line treatment. The response rate of existing second-line chemotherapy is only about 10%, and most patients will develop PD again within a short period of time. With the advent of immune checkpoint inhibitors, immunotherapy has become a new breakthrough in the treatment of advanced bladder cancer. At present, 5 marketed PD-1/PD-L1 antibody products have been approved by the FDA for the treatment of locally advanced/metastatic bladder cancer (Table 1).

Cervical cancer is one of the common malignant tumors in females, ranking second in incidence among female tumors. In China, cervical cancer ranks first among female reproductive system tumors with the seventh mortality among malignant tumor deaths. Although screening and vaccination have led to a decrease in the incidence of cervical cancer, the disease continues to affect females around the world. The survival rate is only 15%-16% for patients with stage IV cervical cancer. At present, there is no effective treatment for patients with advanced or recurrent/metastatic cervical cancer. Especially in the case of progression after first-line

chemotherapy, there are few effective second-line treatment options. The development of immune checkpoint inhibitors provides a new second-line treatment option for patients with advanced cervical cancer. On 12 Jun., 2018, the FDA approved pembrolizumab for the treatment of recurrent or metastatic cervical cancer as well as cervical cancer that has progressed after chemotherapy. Patients must be tested for PD-L1 expression (CPS ≥ 1) using FDA-approved reagents. The approval was based on study KEYNOTE-158, which enrolled 98 patients with recurrent or metastatic cervical squamous cell carcinoma. The patients received pembrolizumab by intravenous injection at a dose of 200 mg once every 3 weeks until unacceptable toxicity or documented PD. The median duration of follow-up was 11.7 months. The ORR of 77 patients was 14.3%, with a complete response rate of 2.6% and a partial response rate of 11.7%. The duration of response was ≥ 6 months in 91% of patients. No response was observed in patients with negative PD-L1 expression (CPS < 1).

**Table 1. Clinical studies of marketed PD-1/PD-L1 inhibitor monotherapies in advanced urothelial carcinoma.**

| Immune Checkpoint Inhibitor | Indication | Study Design | Dosing Regimen | Study Endpoint |
|---|---|---|---|---|
| Atezolizumab | Second-line treatment of locally advanced or metastatic urothelial carcinoma that has progressed during or after platinum-based chemotherapy or within 12 months after platinum-based neoadjuvant or adjuvant chemotherapy | IMvigor210 (Cohort 2): a single- arm phase II clinical study (n = 310) | 1200 mg IV q3w | ORR: 14.8%; mDOR: 27.7 months |
| | First-line treatment of locally advanced/metastatic urothelial carcinoma | IMvigor210 (Cohort 1): a single- arm phase II clinical study (n = 119) | 1200 mg IV q3w | ORR: 23.5%; mOS: 15.9 months; mPFS: 2.7 months |
| Nivolumab | Second-line treatment of locally advanced or metastatic urothelial carcinoma that has progressed during or after platinum-based chemotherapy or within 12 months after platinum-based neoadjuvant or adjuvant chemotherapy | CheckMate275: a single-arm phase II clinical study (n = 270) | 3 mg/kg IV q2w | ORR: 19.6%; mOS: 8.7 months; mPFS: 2.0 months |
| Pembrolizumab | Second-line treatment of locally advanced or metastatic urothelial carcinoma that has progressed during or after platinum-based chemotherapy or within 12 months after platinum-based neoadjuvant or adjuvant chemotherapy | KEYNOTE-045: a randomized, open-label, controlled phase III clinical study (n = 542) | Pembrolizumab: 200 mg IV q3w Control group: paclitaxel (175 mg/m2), docetaxel (75 mg/m2) or vinflunine (320 mg/m2) IV q3w | mOS: 10.3 months vs 7.4 months; ORR: 21.1% vs. 11.4% |
| | First-line treatment of locally advanced or metastatic urothelial carcinoma | KEYNOTE-052: a single-arm phase II clinical study (n = 370) | 200 mg IV q3w | ORR: 29% |

| Immune Checkpoint Inhibitor | Indication | Study Design | Dosing Regimen | Study Endpoint |
|---|---|---|---|---|
| Durvalumab | Second-line treatment of locally advanced or metastatic urothelial carcinoma that has progressed during or after platinum-based chemotherapy or within 12 months after platinum-based neoadjuvant or adjuvant chemotherapy | Study 1108: a single-arm phase I/II clinical study (n = 182) | 10 mg/kg IV q2w | ORR: 17% (all); ORR: 26.3% (PD-L1 > 5%) |
| Avelumab | Second-line treatment of locally advanced or metastatic urothelial carcinoma that has progressed during or after platinum-based chemotherapy or within 12 months after platinum-based neoadjuvant or adjuvant chemotherapy | JAVELIN: a single-arm phase III clinical study (n = 242) | 10 mg/kg IV q2w | ORR: 16.1% |

Endometrial cancer is a group of epithelial malignancies that occur commonly in the endometrium of perimenopausal and postmenopausal females. Endometrial cancer is one of the most common female reproductive neoplasms, with nearly 200,000 new cases each year, and is the third most common gynecological malignancy (next to ovarian cancer and cervical cancer) that causes death. In China, the incidence of endometrial cancer is increasing year by year and currently ranks second in female reproductive malignancies, second only to cervical cancer. On 23 May, 2017, the FDA granted accelerated approval to pembrolizumab for the treatment of adult or pediatric patients with solid tumors with high microsatellite instability (MSI-H) or defective mismatch repair (dMMR). This is the first time that the FDA approves an anti-tumor therapy based on biomarkers instead of origins of tumor tissues. The approval of pembrolizumab was based on 5 uncontrolled, multi- cohort, multi-center, single-arm clinical trials involving 149 patients with MSI-H or dMMR cancers. Among them, 14 patients with endometrial cancer achieved an ORR of 36%, and the DOR has not been reached (range: 4.2 months to 17.3+ months).

Ovarian cancer consists of several different histopathological types, and treatment depends on the specific tumor type. Epithelial ovarian cancer accounts for the majority (approximately 90%) of ovarian malignancies. Epithelial ovarian cancer is the leading cause of death in patients with gynecological cancers and the seventh most common cause of death from malignancies in Chinese females. Due to the lack of effective disease screening methods, most ovarian cancers are already at an advanced stage when diagnosed. The initial treatment for ovarian cancer includes reasonable surgical staging, cytoreductive surgery, and postoperative systemic chemotherapy, i.e., postoperative adjuvant chemotherapy, for most but not all patients. The generally accepted regimen of initial chemotherapy for ovarian cancer is intravenous administration of platinum (cisplatin or carboplatin) combined with taxane (paclitaxel or docetaxel) for 6 cycles. Patients should undergo clinical evaluation again after initial treatment (e.g., 6 cycles of chemotherapy). Follow-up observation may be conducted if there is no evidence of neoplasm progression (i.e., clinical complete response) after initial treatment. Patients who achieve partial response or develop PD during initial treatment should receive second-line treatment. For recurrent platinum-sensitive tumors (time to recurrence/progression ≥ 6 months after completion of platinum-based chemotherapy), platinum- based combination chemotherapy is the first choice, including: carboplatin/paclitaxel, carboplatin/liposomal doxorubicin, weekly carboplatin/paclitaxel, carboplatin/docetaxel, and carboplatin/gemcitabine. For platinum-resistant tumors (time to recurrence/progression < 6 months after completion of platinum-based chemotherapy), non-platinum monotherapies (i.e., docetaxel, oral etoposide, gemcitabine, liposomal doxorubicin, weekly paclitaxel, and topotecan) are the first choice. The response rates of these drugs are usually about 20% in clinical practice. There is an urgent need to develop new low-toxicity and high- efficiency drugs for recurrent ovarian cancer, especially recurrent platinum-resistant ovarian cancer.

Although therapies with anti-PD-1/PD-L1 antibodies have achieved unprecedented success in a variety of cancers, it should not be ignored that a large part of the patient population still does not respond to such therapies. From the current point of view, the combination therapy will be one of the inevitable trends in the future development of immunotherapy of tumors. How to carry out the combination therapy is a problem to be solved by the medical community.

There are currently a large number of phase I/II clinical trials that combine targeted therapy with immunotherapy. The rationale supporting these combination therapies is that the combination of the two therapies combines different immunological and tumor biological mechanisms that enhance the anti-tumor activity. In addition, some evidence suggests that targeted therapies can enhance certain aspects of the "cancer-immune cycle" (such as tumor antigenicity and T cell initiation/transport/infiltration) to synergistically enhance the efficacy of the immunotherapy. In particular, targeted therapies targeting the vascular endothelial growth factor (VEGF) pathways, including drugs such as sunitinib, can have a direct impact on tumor cell growth and tumor angiogenesis, as well as on tumor antigenicity and intratumoral T cell infiltration. Their impact on the patient's immune response is beyond their role in tumor biology, providing a strong basis for the combination therapy.

Current combination therapies have largely focused on advanced melanoma, lung cancer, and renal cell carcinoma. Good clinical prospect is also seen in the treatment of liver cancer, ovarian cancer, colorectal cancer, head and neck cancer, and Hodgkin's lymphoma. Some key factors should be considered in the clinical development of combination therapies, such as optimizing dosing regimens, minimizing treatment-related toxicity, and selecting appropriate endpoints to assess the efficacy.

This clinical study involves recombinant humanized anti-PD-1 monoclonal antibody injection (SHR-1210), a new class 1 therapeutic biological product developed by Jiangsu Hengrui Pharmaceuticals Co., Ltd. that has been officially approved by the National Medical Products Administration on 29 May, 2019 for the treatment of recurrent or refractory classical Hodgkin lymphoma (cHL) that has been treated with at least two lines of systemic chemotherapy. Preclinical trial data show that SHR-1210 has comparable *in vivo* efficacy and safety with those of similar drugs abroad. Since 2015, Hengrui has conducted phase I/II clinical trials on several types of tumors in both Australia and China, and preliminarily validated the safety, tolerability, and efficacy of SHR-1210 monotherapy in the treatment of advanced solid tumors. For details regarding SHR-1210, refer to the SHR-1210 Investigator's Brochure provided by the sponsor.

This clinical study also involves famitinib malate capsules developed by Jiangsu Hengrui Pharmaceuticals Co., Ltd. Famitinib is a small-molecule targeted drug with significant inhibitory effects on c-Kit, VEGFR2, PDGFRβ, VEGFR3, Flt1, Ret, Flt3, and c- Src kinases. SHR116637, the primary metabolite of famitinib, also has inhibitory effect on these kinases, but the inhibitory activity is weaker than that of famitinib. The positive control compound, sunitinib, also has strong inhibitory effect on these kinases, but the inhibitory effect is about 2-15 times weaker than that of famitinib. The results showed that famitinib and its metabolite SHR116637 are multi- target receptor tyrosine kinase inhibitors; famitinib has obvious selectivity for c-Kit; the inhibitory activity of famitinib against tyrosine kinases is significantly stronger than that of sunitinib (Table 2).

**Table 2. Comparison of the inhibitory activity of sunitinib, famitinib, and famitinib metabolite against receptor tyrosine kinases.**

| Kinase | IC50 (nM, mean±SD) | | |
|---|---|---|---|
| | Sunitinib | Famitinib | The Primary Metabolite of Famitinib, SHR116637 |
| KDR（VEGFR2） | 14.5±7.6 | 4.7±2.9 | 5.3±1.1 |
| c-Kit | 34.8±22.1 | 2.3±2.6 | 22.3±14 |
| PDGFRβ | 8.7±4.4 | 6.6±1.1 | 14±8 |
| Flt1 | 184.6±79.5 | 27.9±15.1 | 42±6.4 |
| VEGFR3 | 137.4±82.6 | 56.3±56.6 | 55.1±2.6 |
| Ret | 187.4±16.7 | 83.9±11.6 | 94.8±39.7 |
| c-Src | 3843±2718 | 757.5±339.5 | 2709±821 |
| Flt3 | 184.2±40.1 | 56.2±11.1 | 198.2±143.6 |
| HER2 | >10,000 | >10,000 | >10,000 |
| EGFR | >10,000 | >10,000 | >10,000 |

The completed phase I clinical study of famitinib malate in advanced solid tumors showed that a daily dose of 4-27 mg of famitinib malate is a safe and tolerable dose range. Dose escalation studies showed a DLT dose of 30 mg and an MTD dose of 27 mg. The DLTs of oral monotherapy were Grade 3 hypertension and Grade 3 hand-and-foot syndrome. The famitinib 20 mg, 25 mg, and 27 mg groups were further expanded to include 9, 14, and 12 subjects, respectively, in total. The DLT percentage of ≥ 33.3% specified in the protocol was not observed, indicating favorable tolerability of continuous administration of famitinib.

Adverse reactions occurred in 51 of 54 subjects in the phase I clinical study. The incidence of adverse reactions of all grades was 94.44%. The incidence of Grade 3-4 adverse reactions was 31.48%. Most adverse reactions were Grade 1 (6 subjects) or 2 (28 subjects). Fifteen subjects had Grade 3 and 2 subjects had Grade 4 adverse reactions.

During the tolerability observation period in the 2 cycles, common hematological adverse reactions (number of subjects, % all grades, % Grade 3/4) with an incidence of ≥ 5% included white blood cell count decreased (33 subjects, 61.11%, 5.56%), neutrophil count decreased (29 subjects, 53.7%, 5.56%), platelet count decreased (19 subjects, 35.19%, 5.56%), and anemia (10 subjects, 18.52%, 3.70%).

During the tolerability observation period in the 2 cycles, common non-hematologic adverse reactions (number of subjects, %all grades, %Grade 3/4) with an incidence of ≥ 5% included hypertension (23 subjects, 42.59%, 7.41%), hand-and-foot syndrome (20 subjects, 37.04%, 3.7%), urine protein increased (20 subjects, 37.04%, 0.00%), asthenia (19 subjects, 35.19%, 0.00 %), triglycerides increased (19 subjects, 35.19%, 3.70%), oral mucositis (18 subjects, 33.33%, 0.00%), blood cholesterol increased (15 subjects, 27.78%, 0.00%), AST increased (14 subjects, 25.93%, 0.00%), TBIL increased (13 subjects, 24.07%, 0.00%), CK/CK-MB increased (10 subjects, 18.52%, 0.00%), hypothyroidism (10 subjects, 18.52%, 0.00%), ALT increased (9 subjects, 16.67%, 1.85%), diarrhea (9 subjects, 16.67%, 0.00%), decreased appetite (9 subjects, 16.67%, 0.00%), abnormal tooth function (8 subjects, 14.81%, 0.00%), GGT increased (8 subjects, 14.81%, 3.7%), adrenocorticotropic hormone increased (7 subjects, 12.96%, 0.00%), constipation (6 subjects, 11.11 %, 0.00%), sensory neurotoxicity (5 subjects, 9.26%, 0.00%), nausea (4 subjects, 7.41%, 0.00%), skin hypopigmentation (4 subjects, 7.41%, 0.00%), and dizziness (3 subjects , 5.56%, 0.00%).

In the PK study, a total of 47 patients with advanced solid tumors were enrolled, including 27 males and 20 females. These patients participated in the single-dose and repeated-dose PK studies of different doses, respectively.

After a single oral administration of different doses of famitinib malate, the times to maximum concentrations of parent drug and metabolite in plasma were 3.3-5.3 h and 4.0-6.2 h, respectively; the plasma exposure of the metabolite was comparable to about 3.6% of that of the parent drug. The elimination of both compounds from the plasma was slow, and the elimination half-life cannot be accurately calculated within the sampling time set in the trial. Meals had no obvious effect on the exposure of parent drug and metabolite in the body. In the dose range of 4-27 mg, the $C_{max}$ and $AUC_{0-24}$ values of parent drug and metabolite increased proportionally with dose.

After Day 1 of continuous oral administration of different doses of famitinib malate, the times to maximum concentrations of parent drug and metabolite in the plasma were 3.0-7.0 h and 3.7-8.1 h, respectively; the plasma exposure of the metabolite was approximately 5.1% of that of the parent drug. After 28 days of continuous administration, the times to maximum concentrations were 3.5-7.1 h and 6.1-12.8 h, respectively, and the plasma exposure of the metabolite was approximately 8.0% of that of the parent drug.

The food-effect study in healthy subjects suggested that, after the administration of the same dose of famitinib malate after meals, the mean times to maximum concentrations of parent drug and metabolite in the plasma were prolonged to 12 h and 18 h, respectively; the maximum concentration $C_{max}$ of the parent drug was decreased by about 16%, and the exposure $AUC_{0-t}$ was 105.1% times the value obtained after administration under fasting condition, indicating no significant change; the $C_{max}$ and $AUC_{0-t}$ of the metabolite were 95.7% and 96.3% of the values obtained after administration under fasting condition, respectively, indicating no significant change. The results suggested that the exposure level of famitinib in humans was basically not affected by meals.

This study explores a new combination therapy, which is urgently needed in clinical practice, not only for laying a foundation for future studies, but also for exploring biomarkers related to cancer immunity. Therefore, it has important scientific significance and clinical value.

## 1.2. Scientific Rationale

Increasing the percentage of patients benefiting from anti-PD-1/PD-L1 antibodies through combination with anti-angiogenic drugs is the focus of current research in the field of immunotherapy. Multiple studies on combination therapy with anti-angiogenic drugs are ongoing. The obtained study results proved that the combination with anti-angiogenics can significantly improve the efficacy of anti-PD-1/PD-L1 in a variety of tumors, with controllable safety[1].

**Table 3. Combination of anti-PD-1/PD-L1 inhibitors and VEGF/VEGFR inhibitors for the treatment of tumors.**

| Drug Combination | Indication (Efficacy) |
|---|---|
| Nivolumab+ Sunitinib/Pazopanib | Advanced renal cell carcinoma (ORR: 42%/45%<br>24-week PFS: 78%/55%) |
| Pembrolizumab+Pazopanib | Advanced renal cell carcinoma |
| Avelumab+Axitinib | Advanced renal cell carcinoma (ORR: 58.2%) |
| Nivolumab+Bevacizumab | Stage III NSCLC;<br>Metastatic renal cell carcinoma |
| Pembrolizumab+Bevacizumab | Metastatic non-squamous NSCLC |
| Atezolizumab+ Bevacizumab+ Paclitaxel +platinum | Non-squamous NSCLC (mPFS: 8.3 months)<br>recurrent ovarian cancer |
| Pembrolizumab+Lenvatinib | Advanced renal cell carcinoma (ORR: 83%);<br>Head and neck squamous cell carcinoma (ORR: 40.9%)<br>Endometrial cancer (ORR: 47.2%)<br>Unresectable hepatocellular carcinoma (ORR: 42.3%)<br>urothelial carcinoma |
| Atezolizumab+ Bevacizumab | Advanced or metastatic hepatocellular carcinoma<br>(ORR: 65%) |

### 1.2.1. Study rationale

The anti-PD-1 antibody camrelizumab (SHR-1210) has been formally approved by the National Medical Products Administration on 29 May, 2019 for the treatment of recurrent or refractory classical Hodgkin lymphoma (cHL) that has been treated with at least two lines of systemic chemotherapy. Subject enrollment has been completed in multiple pivotal phase III studies. The published study results showed that the efficacy of SHR-1210 for a variety of tumors is comparable to that of similar products, and the toxicity information is comprehensive.

In addition, the results of multiple clinical studies of SHR-1210 combined with another anti- angiogenic agent of Hengrui, apatinib mesylate tablets, showed that the combination of SHR-1210 and anti- angiogenic agent effectively reduced the incidence of cutaneous capillary proliferation and significantly improved the efficacy of SHR-1210[2,3].

Famitinib malate capsules have been proved to be effective and safe through multiple phase I/II clinical studies. The preclinical activity of famitinib compared with the similar drug sunitinib: Famitinib showed more significant inhibitory effect on VEGFR2, c-Kit, PDGFR, and Flt1 kinases, as well as more significant anti-tumor effect on renal cancer Caki-1, hepatic cancer Bel- 7402, and other xenografts in nude mice. The clinical characteristics of famitinib compared with sunitinib: Famitinib by oral administration showed higher exposure and less accumulation in the human body. In a phase II clinical study of famitinib vs. sunitinib for the treatment of advanced renal cell carcinoma, the objective response rate (ORR) of famitinib (25 mg/d, continuous oral administration) was 36.0%, while the ORR of the sunitinib control group (50 mg/d, 4 weeks on and 2 weeks off in each 6-week cycle) was 28.0%. In terms of safety, compared with sunitinib, the incidences of Grade 1/2 hypertension and proteinuria in the famitinib group were slightly higher than those in the sunitinib group, and the incidences of overall and Grade 3 platelet count decreased were lower than those in the sunitinib group (9% vs. 24.0%), indicating a good safety profile. In the study, the rates of treatment interruption and dose reduction observed in the famitinib group were comparable to those in the sunitinib group, indicating that famitinib was well tolerated.

Famitinib is safe and well tolerated, supporting the combination with SHR-1210, to explore the efficacy and safety of SHR-1210 combined with famitinib against various solid tumors.

Anti-PD-1 monoclonal antibody monotherapy is evidently effective as the first-line treatment for cisplatin-intolerant, PD-L1-positive (CPS ≥ 10), or platinum-intolerant urothelial carcinoma, and as the second-line treatment for urothelial carcinoma that has progressed during or after prior platinum-based therapy, but no study has been conducted to explore the efficacy of SHR-1210 monotherapy in the treatment of urothelial carcinoma. Multiple studies have shown that targeted

anti-angiogenic agents, whether in monotherapy such as cabozantinib or in combination therapy such as ramucirumab combined with paclitaxel, have certain efficacy in the treatment of urothelial carcinoma[4-6], but the efficacy of famitinib in urothelial carcinoma has not been explored in any study. The OS and ORR of bevacizumab combined with chemotherapy as the first-line treatment for recurrent or metastatic cervical cancer were significantly improved compared with chemotherapy alone[7]. Accordingly, the NCCN guidelines recommend this therapy as the first-line treatment for recurrent or metastatic cervical cancer. In addition, bevacizumab monotherapy is also recommended by the NCCN guidelines as the second-line treatment for recurrent or metastatic cervical cancer. Therefore, in order to investigate the contribution of each drug in the combination therapy regimen of SHR-1210 and famitinib, an SHR-1210 monotherapy group and a famitinib monotherapy group will be added for exploration in urothelial carcinoma and cervical cancer.

In recent years, remarkable results of studies on anti-PD-1 or PD-L1 antibodies combined with targeted anti-angiogenic drugs as the first-line treatment for advanced renal cancer have been achieved. The study results of pembrolizumab combined with axitinib as the first-line treatment for advanced renal cancer (Keynote-426)[8] showed that, compared with the sunitinib control group, objective response rate (ORR), progression-free survival (PFS), and overall survival (OS) were significantly improved in the combination therapy group. For the first time, the ORR of first-line treatment for advanced renal cancer was increased from 25%-35% in the era of targeted anti-angiogenic therapy to 50%-60%, mPFS was improved from 8-10 months to 13-15 months, and the risk of death was reduced by approximately 30%. Meanwhile, similar results have been achieved in the first-line treatment of advanced renal cancer with avelumab combined with axitinib (Javelin Renal 101)[9]. Based on results from the two studies, in 2019, the FDA approved the marketing applications of pembrolizumab combined with axitinib and avelumab combined with axitinib, ushering in the era of anti-PD-1 or PD-L1 antibodies combined with targeted anti- angiogenic therapy for advanced renal cancer. Encouraged by this, an increasing number of clinical trials of anti-PD-1 or PD-L1 antibodies combined with targeted anti-angiogenic therapy as the first-line treatment for advanced renal cancer are being actively carried out, and some of them have achieved good preliminary results, making it a clinical problem that needs to be solved urgently how to treat advanced renal cancer that has progressed after anti-PD-1 or PD-L1 antibody therapy.

In order to address this problem, Merck and Eisai have taken the lead in exploring the preliminary efficacy of pembrolizumab combined with lenvatinib for the treatment of advanced renal cancer that had progressed after previous anti-PD-1 or PD-L1 antibody therapy (Keynote146)[10]. The study results showed that the ORR was as high as 59% for patients with advanced renal cancer that had progressed after previous treatment with anti-PD-1 or PD-L1 and

targeted anti-angiogenic therapy. This suggested that advanced renal cancer that has progressed after previous treatment with anti-PD-1 or PD-L1 antibodies and targeted anti-angiogenic therapy can still benefit significantly from anti-PD-1 or PD-L1 antibodies combined with targeted anti-angiogenic therapy for the second time.

### 1.2.2. Rationale for dosing regimen design

The results of a previous study of sunitinib, a similar drug to famitinib, in combination with nivolumab, a similar anti-PD-1 antibody to SHR-1210, confirmed that the standard dose of sunitinib (50 mg qd, 4 weeks on and 2 weeks off in each 6-week cycle) was well tolerated in combination with nivolumab 2 mg/kg or 5 mg/kg q3w. In addition, there was no DLT observed during the tolerability observation period. The main Grade 3/4 adverse reactions were hypertension, ALT increased, hemoglobin decreased, lymphocyte count decreased, diarrhea, AST increased, and asthenia. These adverse reactions were reversible and relieved by symptomatic treatment, suggesting that labeled dose of sunitinib with nivolumab was well tolerated and safe.

The results of several studies of SHR-1210 combined with apatinib suggest that SHR-1210 200 mg q2w and apatinib 250 mg qd are well tolerated and safe. It is suggested that SHR-1210 can be administered at a fixed dose of 200 mg q2w when used in combination with anti-angiogenic drugs. In a phase II study of SHR-1210 monotherapy as second-line treatment for advanced hepatocellular carcinoma, SHR-1210 200 mg q2w and SHR-1210 200 mg q3w had comparable efficacy. Also, PD-1 receptor occupancy data suggested that, with SHR-1210 administered once, after 4 weeks of observation, the PD-1 receptor occupancy rate remained above 90%. These findings suggested that the efficacy of the SHR-1210 200 mg q3w administration may not be inferior to that of the SHR-1210 q2w administration. The SHR-1210 q3w administration could reduce the frequency of in-patient injections of subjects while ensuring the efficacy. Therefore, SHR-1210 200 mg q3w combined with famitinib will be considered in this study.

Previous studies showed that continuous oral administration of famitinib 25 mg qd produced similar safety and tolerability results to sunitinib 50 mg qd (4 weeks on and 2 weeks off in each 6-week cycle). Combined with the results of the sunitinib plus nivolumab study, it was suggested that continuous oral administration of famitinib 25 mg qd combined with SHR-1210 200 mg q3w would be tolerable. However, given the fact that the dose reduction rate when famitinib 25 mg qd is administered continuously orally is relatively high (36% of dose reduction rate was observed in a phase II study of advanced renal cell carcinoma), the study will select famitinib 20 mg qd continuous dosing as the dose of famitinib to be administered in combination, considering that the duration of dosing in co-administered subjects will likely be longer than the duration of dosing alone, and the tolerability of long-term dosing in subjects will be an important concern.

Therefore, in this study, a fixed dose of 200 mg of SHR-1210 (q3w) combined with 20 mg of famitinib (qd, continuous oral administration) is selected as the starting dose level of combination therapy. The following patients will be enrolled:

1. Patients with advanced renal cell carcinoma who have previously been treated with anti- angiogenic agents but have failed the treatment or are unwilling to accept/unable to afford anti-angiogenic therapy;
2. Patients with advanced urothelial carcinoma who have previously been treated with platinum- based regimens but have failed the treatment or have relapsed or progressed after the treatment;
3. Patients with advanced cervical cancer who have previously been treated with platinum-based regimens but have failed the treatment or have relapsed or progressed after the treatment;
4. Patients with endometrial cancer who have relapsed or developed metastases after prior systemic treatment;
5. Patients with platinum-resistant or platinum-refractory recurrent ovarian cancer.

The efficacy and safety of the combination therapy will be observed to evaluate the efficacy and safety of SHR-1210 combined with famitinib in these solid tumors and thus to provide effective and safe treatment options for patients with these tumors.

In order to investigate the contribution of each drug in the combination therapy regimen, an SHR-1210 monotherapy group and a famitinib monotherapy group will be added. The 200 mg q3w dose is selected for the SHR-1210 monotherapy group based on the results of the phase II study of SHR-1210 monotherapy in advanced HCC. Patients with metastatic urothelial carcinoma will be enrolled to investigate the efficacy and safety of SHR-1210 monotherapy in urothelial carcinoma. The recommended phase II dose of famitinib monotherapy, 25 mg qd, is selected for the famitinib monotherapy group. Patients with metastatic urothelial carcinoma or recurrent/metastatic cervical cancer will be enrolled to investigate the efficacy and safety of famitinib monotherapy in corresponding tumor types.

## 1.3. Potential Risks and Benefits

### 1.3.1. Known potential risks

Any investigational product or treatment may have unpredictable or even serious side effects.

As of 25 Aug., 2017, among all subjects who have received SHR-1210, 214 (34.3%) reported skin toxicity event hemangioma (which has not been reported for other anti-PD-1 IgG4 antibodies). Among subjects who experienced hemangioma, 6 underwent surgery and/or were hospitalized for long-term clinical observation. Therefore, hemangioma in these subjects were considered as SAEs. All hemangiomas were reported as Grade ≤ 2. The hemangiomas resolved after discontinuation of SHR-1210.

In SHR-1210 studies conducted in Australia and China, 7 (1.1%) subjects reported pneumonitis SAEs and 3 (0.5%) reported interstitial lung disease SAEs.

Other immune-related adverse events (irAEs) frequently reported for other anti-PD-1 antibodies were considered to be class effects and were also observed in SHR-1210 clinical studies. These included AST increased (73 subjects, 11.7%), ALT increased (58 subjects, 9.3%), rash (57 subjects, 9.1%; preferred terms include rash, rash macular, rash maculo-papular, rash erythematous, rash pustular, and rash pruritic), diarrhea (44 subjects, 7.1%), hypothyroidism (37 subjects, 5.9%), and hyperthyroidism (8 subjects, 1.3%). These events are thought to be related to the investigational product due to their high incidence and consistency with safety outcomes of other anti-PD-1 antibodies. Note that only 1 case of diarrhea was reported as an SAE. There was no colitis reported as an SAE. Therefore, many diarrhea events may not be immune-related, but may be caused by underlying conditions. In addition, many patients with AST and ALT increased were with liver cancer. Among the 659 subjects enrolled in SHR-1210 studies in China, nearly a quarter were patients with primary liver cancer.

Analyses showed that the investigator-assessed immune-mediated AEs assessed where SHR- 1210 could not be ruled out as a potential cause were prominently skin toxicities (such as rash or hemangioma of skin). Most immune-mediated AEs were Grade 1-2 in severity.

The above data were compared with adverse reactions reported for other approved anti-PD-1 antibodies - nivolumab from BMS and pembrolizumab from Merck. The incidence and severity were both low. Overall, the adverse reactions of this product are expected to be similar to those of nivolumab and pembrolizumab.

Recommended management of common side effects and protocol-specified dose modifications have been established for this study, so that subjects may continue to receive SHR-1210 treatment, given that subjects will benefit clinically.

Adverse drug reactions observed in phase I clinical studies of famitinib included:

Hematologic toxicity (with an incidence rate of ≥ 5%, sorted in descending order of incidence): white blood cell count decreased, neutrophil count decreased, platelet count decreased, and anemia;

Non-hematologic toxicity (with an incidence rate of ≥ 5%, sorted in descending order of incidence): hypertension, hand-and-foot syndrome, urine protein increased, asthenia, triglycerides increased, oral mucositis, blood cholesterol increased, AST increased, TBIL increased, CK/CK-MB increased, hypothyroidism, ALT increased, diarrhea, decreased appetite, dentofacial functional disorder, GGT increased, adrenocorticotropic hormone increased, constipation, sensory neurotoxicity, nausea, skin hypopigmentation, dizziness, etc.

Compared with the adverse reactions of monotherapy of anti-angiogenic drugs, the overall adverse reactions occurring after combined administration of famitinib with anti-angiogenic drugs were mainly manifested as an increased incidence of adverse reactions such as hypertension, proteinuria, hand-and-foot syndrome, asthenia, decreased appetite, rash, AST increased, ALT increased, white blood cell count decreased, hemoglobin decreased, and platelet count decreased, but the drugs were well tolerated. Immune-related adverse reactions still require special interest, including immune-related interstitial pneumonia, rash, thyroiditis, as well as those with lower incidence (≤ 1%) such as vitiligo, colitis, nephritis, hepatitis, uveitis, adrenal insufficiency, and nerve palsy. These immune-related adverse reactions are mostly mild and controllable. Very few are SAEs or potentially life-threatening. Thanks to established procedures for toxicity management, the majority of immune-mediated AEs can be adequately controlled.

Subjects receiving macromolecular protein monoclonal antibody drugs may also encounter other risks including infusion reactions, which prominently manifest as chills, shivers, facial and peripheral cyanosis, followed by fever and probably accompanied by nausea, vomiting, headache, dizziness, dysphoria, delirium, etc. In severe cases, there may be coma, fall in blood pressure, and symptoms such as shock and respiratory failure. These risks may arise due to various factors during intravenous infusion, such as pyrogens, drugs, impurities, low temperature of drug formulation, high concentration of drug, and high rate of infusion. SHR-1210 is a fully humanized monoclonal antibody. Its reported infusion reactions were low in incidence and mild in severity.

A tumor biopsy may be required for subjects in this study in order to obtain tumor tissues (after obtaining informed consent). This is required for determining PD-L1 expression level and other biomarkers for predicting treatment response, and can generally be completed successfully. However, due to health status of subjects, individual differences, and certain unpredictable factors, anaphylactic shock due to anesthesia, local pain, local infection, local bleeding, and local nerve injury may occur, or severe occult diseases may be induced. Also, pathological examinations for malignant tumors may not be completed with the tissue obtained from one biopsy. Thus biopsies may be repeated when necessary.

Medical examinations during the study may also pose risks to subjects. Frequent tumor imaging assessments may expose subjects to low-dose radiation more frequently. However, since advanced or metastatic non-small cell lung cancer usually progresses rapidly, frequent tumor imaging assessments are necessary to determine whether the subject has PD.

### 1.3.2. Known potential benefits

For the above-mentioned various tumors treated with standard treatment, existing treatments pose limited efficacy, and only less than 20% of all patients achieve tumor response from existing treatments. The study results of similar PD-1/PD-L1 drugs in the treatment of these tumors suggested that anti-PD-1/PD-L1 antibodies play a role in the treatment of these tumors, but the response rate of monotherapy is low or close to 20%, superior to that of existing treatments, but the efficacy awaits further improvement. Combining the study data of the combination of similar immune and anti-angiogenic therapies, participating in the study and receiving the study medication may bring clinical benefits superior to existing treatments and anti-PD-1/PD-L1 antibody monotherapy to patients with advanced renal cell carcinoma, urothelial carcinoma, cervical cancer, endometrial cancer, and refractory/drug-resistant recurrent ovarian cancer, providing these patients with a beneficial treatment option.

## 1.4. Study Objectives

### 1.4.1. Primary objectives

- To evaluate the efficacy of SHR-1210 combined with famitinib in subjects with advanced renal cell carcinoma, urothelial carcinoma, advanced cervical cancer, recurrent ovarian cancer, and endometrial cancer.
- To evaluate the efficacy of SHR-1210 monotherapy in subjects with urothelial carcinoma.
- To evaluate the efficacy of famitinib monotherapy in subjects with urothelial carcinoma and cervical cancer.

### 1.4.2. Secondary objectives

- To evaluate the safety and tolerability of SHR-1210 combined with famitinib in the treatment of various tumors.
- To evaluate the safety and tolerability of SHR-1210 monotherapy in subjects with urothelial carcinoma.
- To evaluate the safety and tolerability of famitinib monotherapy in subjects with urothelial carcinoma and cervical cancer.
- To evaluate the PK of SHR-1210 combined with famitinib in subjects with advanced solid tumors and famitinib monotherapy in subjects with urothelial carcinoma and cervical cancer.

- To investigate the anti-SHR-1210 antibodies (ADAs) in subjects receiving combination therapy.

### 1.4.3. Exploratory objective

- To explore biomarkers for response prediction.

## 1.5. Study Endpoints

### 1.5.1. Primary endpoint

- Objective response rate (ORR) assessed as per RECIST 1.1

### 1.5.2. Secondary endpoints

**Efficacy:**

- Duration of response (DOR);
- Disease control rate (DCR);
- Time to response (TTR);
- Progression-free survival (PFS) as per RECIST 1.1;
- Overall survival (OS) and 12-month overall survival rate (12-month OS%)

**Safety and tolerability:**

- Adverse events (AEs): including type, incidence, grade (according to NCI-CTCAE v4.03 criteria), severity, duration, and causality with the investigational products;
- Laboratory abnormalities: including type, incidence, and grade (according to NCI-CTCAE v4.03 criteria);
- Vital signs: including blood pressure, pulse, respiratory rate, body temperature, ECG, and ECOG PS score;
- Incidence of dose interruption, reduction, and discontinuation due to treatment-related AEs.

**Pharmacokinetics:**

- Plasma concentrations and PK parameters (if applicable) of famitinib and its main metabolites for combination therapy and monotherapy, including $C_{max}$, $T_{max}$, $AUC_{0\text{-}24\,h}$, CL/F, and V/F.
- Concentrations of anti-PD-1 antibody SHR-1210 during combination therapy.

**Others:**

- Proportion of subjects with anti-SHR-1210 antibodies (ADAs) during combination therapy.

### 1.5.3. Exploratory endpoints

The proportion of PD-L1-positive cells in tumor tissue (for subjects receiving SHR-1210 combination therapy or monotherapy only), proportion of subjects with dMMR or MSI-H (for endometrial cancer and ovarian cancer only), and proportion of abnormal FGFR2/3 (for urothelial carcinoma treated with combination therapy only).

The relationship of PD-L1 expression and/or other biomarkers with efficacy (efficacy parameters such as ORR/PFS) will be explored.

# 2. STUDY DESIGN

## 2.1. Overview of Study Design

This study is a single-arm, open-label, multicenter phase II clinical study to observe and evaluate the efficacy and safety of anti-PD-1 antibody SHR-1210 combined with famitinib malate in subjects with advanced urinary system tumors and gynecological tumors, and the efficacy and safety of SHR-1210 monotherapy in treatment of urothelial carcinoma, and famitinib monotherapy in treatment of urothelial carcinoma and cervical cancer.

The subjects will be divided into 8 cohorts according to tumor type or investigational products:

Cohort 1: renal cell carcinoma (no prior anti-PD-1/PD-L1/CTLA-4 antibody treatment)

Cohort 2: urothelial carcinoma

Cohort 3: recurrent ovarian cancer

Cohort 4: endometrial cancer

Cohort 5: cervical cancer

Cohort 6: SHR-1210 monotherapy (urothelial carcinoma)

Cohort 7: famitinib monotherapy (urothelial carcinoma and cervical cancer)

Cohort 8: renal cell carcinoma (prior anti-PD-1/PD-L1/CTLA-4 antibody treatment)

- **Cohorts 1-5**

A combination therapy of oral famitinib 20 mg, qd and SHR-1210 200 mg fixed dose, intravenous infusion (iv) once every 3 weeks (q3w) will be selected in this study, to observe its efficacy and safety in 5 types of tumors (cohorts), i.e., renal cell carcinoma, urothelial carcinoma, recurrent ovarian cancer, endometrial cancer, and cervical cancer. Cohorts 1-5 will adopt a two- stage adaptive design, in which 22 subjects will be enrolled in Stage I for a specific tumor, and the enrollment in Stage II depends on the number of subjects with tumor response among the 22 subjects. Whether to conduct a further expansion study for a certain tumor will be determined based on the efficacy of enrolled 22-53 subjects. See Section 0 for details.

Safety data from observation of the first 12 subjects who are enrolled in Cohorts 1-5 after completion of the first two cycles of combination therapy will be summarized and analyzed. If clinically significant toxicity is observed in ≥ 4 out of these 12 subjects, or if > 30% subjects (for Cohorts 1-5) requires a dose reduction of famitinib, the combination of SHR-1210 with famitinib 20 mg, qd will be deemed to be poorly tolerated. Subjects enrolled subsequently should be given a combination of famitinib 15 mg, qd and SHR-1210 200 mg, q3w for observation.

The first 12 subjects enrolled in Cohorts 1-5 will undergo blood sampling for PK analysis to explore the blood concentrations and PK parameters of famitinib and SHR-1210 in combination therapy.

➢ **Cohorts 6-7**

In addition, subjects with urothelial carcinoma and cervical cancer will be screened for monotherapy study of Cohorts 6 and 7. Among them, subjects with urothelial carcinoma will be randomly assigned to Cohort 6 (SHR-1210 monotherapy group, a fixed dose of 200 mg, intravenous infusion (iv), q3w) or Cohort 7 (famitinib monotherapy group, 25 mg, qd, continuous oral administration) to be observed for efficacy and safety. Subjects with cervical cancer will be directly enrolled in Cohort 7 to receive famitinib monotherapy (25 mg, qd, continuous oral administration), and the efficacy and safety will be observed. 14-23 subjects with urothelial carcinoma and cervical cancer will be enrolled in each of Cohorts 6 and 7. After Stage I enrollment and efficacy observation, whether to proceed to Stage II enrollment will be decided according to the number of subjects with response in Stage I.

Subjects in Cohort 7 will also undergo blood sampling for PK analysis (only at sites with conditions for PK blood sampling and processing) to analyze the PK of famitinib monotherapy in subjects with urothelial carcinoma and cervical cancer.

➢ **Cohort 8**

Subjects with advanced renal cell carcinoma will be screened to Cohort 8 to receive SHR-1210 (200 mg, q3w, iv) combined with famitinib (20 mg, qd, continuous oral administration), and the efficacy and safety will be observed. 21 subjects will be enrolled in Stage I. After Stage I enrollment and efficacy observation, whether to proceed to Stage II enrollment will be decided according to the number of subjects with response in Stage I.

Subjects in Cohort 8 will undergo blood sampling for PK analysis (only at sites with conditions for PK blood sampling and processing) to explore the PK of famitinib in combination therapy of renal cancer.

This study will consist of screening period, treatment period, and follow-up period. After signing the informed consent, subjects will proceed to the screening period. Eligible subjects in Cohorts 1-5 and 8 as well as eligible subjects with cervical cancer in Cohort 7 will proceed to the treatment period. Additional eligible subjects with urothelial carcinoma will be randomized to Cohort 6 or 7.

All subjects will receive treatment with the investigational products until the criteria for treatment discontinuation as specified in the protocol are met. After the end of treatment, subjects will continue safety follow-ups and survival follow-ups. Subjects who discontinue the treatment due to reasons other than PD/death will also be followed for PD after discontinuation.

For Cohorts 6 and 7, if subjects discontinue treatment (SHR-1210 monotherapy or famitinib monotherapy) due to PD, with the informed consent of the subjects, SHR-1210 combined with famitinib can be used to continue treatment until the study treatment discontinuation criteria specified in the protocol are met.

After enrollment in the study, subjects will undergo safety follow-ups prior to administration on D1 and D7 (± 1 d) of Cycle 1, as well as D1 (± 3 d) of each subsequent cycle. Imaging assessments should be conducted once every 3 cycles (9 weeks) to evaluate efficacy from the start of treatment until radiographic progression, commencement of new anti-tumor treatment, withdrawal of informed consent, loss to follow-up, or death. The visit cycle and efficacy evaluation of subjects who continue treatment after PD in Cohorts 6 and 7 are the same as those before PD.

Existing paraffin-embedded tumor tissue sections will be acquired/collected during the screening period. Prior to the first dose, it is also recommended to collect fresh biopsy specimens (core needle biopsy), prepare 3 tumor sections with a thickness of 4-5 µm after fixation and embedding, and collect 5 mL of whole blood for biomarker detection, including but not limited to: proportion of PD-L1-positive cells (for subjects receiving SHR-1210 combination therapy or monotherapy only), MMR (for endometrial cancer and ovarian cancer only), and FGFR2/3 mutation (for urothelial carcinoma treated with combination therapy only).

### 2.1.1. Clinically significant toxicity

Limited to events observed in the first 12 subjects enrolled in Cohorts 1-5 within the first 2 cycles that are deemed related to the investigational products by the investigators and meet the following:

1. Grade 4 hematologic toxicity that lasts ≥ 3 days, Grade ≥ 3 thrombocytopenia with hemorrhage, or Grade ≥ 3 neutropenia with fever and infection;

2. Grade ≥ 3 non-hematologic toxicity (except for laboratory abnormalities), Grade 3 hypertension, rash, diarrhea, nausea, and vomiting that cannot be effectively controlled after symptomatic treatment;
3. Grade ≥ 3 laboratory abnormalities that lead to hospitalization or last ≥ 7 days;
4. Related toxicity resulting in failure to complete 2 administrations of SHR-1210 within the first two cycles or normal SHR-1210 administration in Cycle 3 (treatment delay > 7 days);
5. Related toxicity resulting in famitinib interruption for > 14 days.

If any of the first 12 subjects fails to complete treatment observation for two cycles due to non- drug-related toxicity, the subject must be replaced.

### 2.1.2. Follow-up management of subjects with clinically significant toxicity

Subjects with clinically significant toxicity may receive subsequent SHR-1210 and famitinib combination therapy as assessed by the investigators after they have recovered from toxicity. The investigators may interrupt famitinib, reduce the dose of famitinib, modify the method of famitinib administration, discontinue famitinib, or discontinue the study treatment according to the dose modification principle. Subjects will also be followed up and their data will be collected according to the Schedule of Activities.

### 2.1.3. Randomization

This is an open-label, multicenter phase II clinical study. Only subjects with urothelial carcinoma for Cohorts 6 and 7 will be randomized. After the completion of enrollment of Cohort 2, eligible subjects with urothelial carcinoma will be assigned at a 1:1 ratio to Cohort 6 or 7 to receive SHR-1210 or famitinib monotherapy, with no stratification factor.

For Cohorts 6 and 7, randomization will be performed using an interactive web response system (IWRS). Subjects participating in this study will be assigned subject ID as the unique identification code. Eligible subjects will be assigned a randomization number and a cohort by the IWRS to receive treatment corresponding to the assigned cohort.

## 2.2. Blood Sampling for PK and ADA Analysis

### 2.2.1. Study procedure and arrangement of blood sampling points

➢ Cohorts 1-5

The first 12 subjects enrolled in Cohorts 1-5 will undergo blood sampling for PK and ADA analysis.

For SHR-1210 PK analysis, blood samples will be collected within 30 min before administration and within 5 min after administration (including flushing) of the first SHR-1210 dose; within 30 min before administration and within 5 min after administration (including flushing) of SHR- 1210 on administration days of Cycles 2, 3, and 4; within 30 min pre-administration every 4 cycles thereafter; at the end of SHR-1210 treatment or upon withdrawal from study (the documented time of last SHR- 1210 administration prior to the end of treatment or withdrawal from study); at 30 days after the end of SHR-1210 treatment. (If subject completes the treatment before scheduled blood sampling is completed, the analysis should be based on actual completed sampling).

ADA blood samples will be simultaneously collected along with PK samples before each administration, at the end of treatment or upon withdrawal from study, and at 30 days after the end of treatment.

For SHR-1210, 4 mL should be collected for PK analysis and 4 mL for ADA analysis at each blood sampling point. The serum should be separated.

For famitinib PK analysis, 3 mL of blood sample will be collected on D1 (± 3 d) of Cycle 3 at each of the following time points: within 30 min pre-administration, and at 2 h ± 5 min, 4 h ± 5 min, 6 h ± 5 min, 8 h ± 5 min, 10 h ± 5 min, and 24 h ± 30 min post-administration (before D2 administration), and the plasma will be separated.

If famitinib administration is interrupted for a subject due to AEs or other reasons within 7 days before the scheduled blood sampling time of famitinib, then PK blood sampling for famitinib will not be performed on C3D1. After resuming famitinib administration for at least 14 consecutive days, drugs will be given with the protocol-specified method of administration of famitinib on C3D1 (fasting administration) and blood samples will be collected at the protocol- specified sampling time points.

In the case of dose reduction of famitinib due to toxicity before the PK blood sampling for famitinib, drugs will be given with the protocol-specified method of administration of famitinib on C3D1 (fasting administration) and blood samples will be collected at the protocol-specified sampling time points after at least 14 days of continuous administration of famitinib following the dose reduction.

If famitinib administration is discontinued due to toxicity before blood sampling for famitinib PK analysis, blood sampling for famitinib will no longer be performed for the subject.

- Cohorts 7 and 8

For famitinib PK analysis, 3 mL of blood sample will be collected (only at sites with conditions for PK blood sampling and processing) at each of the following time points: at 6 (± 1) h post- administration on C1D1, within 30 min pre-administration and at 6 (± 1) h post- administration on C2D1 and C3D1, and the plasma will be separated.

If famitinib administration is interrupted on the day of sampling, pre-administration sampling should be continued but post-administration sampling should be skipped. On the sampling day, it is also necessary to record the previous administration time.

Famitinib will be administered under fasting condition on the days of blood sampling. That is, food but not water is prohibited after dinner the day before blood sampling. Subjects will take famitinib capsules orally before breakfast on the day of blood sampling and will be fasted for food but not water within 2 h after administration.

### 2.2.2. Blood sample processing and testing

Refer to the laboratory manual for details on blood sampling, processing, and transportation methods.

## 2.3. Acquisition, Collection, and Processing of Biomarkers

1) Existing paraffin-embedded tumor tissue sections will be collected, and 5 tumor sections with a thickness of 4-5 μm will be used for PD-L1 detection (except for Cohort 7).
2) It is recommended that biopsy samples (core needle biopsy) be collected before the first dose. After fixation and embedding, 3 tumor sections with a thickness of 4-5 μm will be prepared for PD-L1 detection (except for Cohort 7);
3) For endometrial cancer and ovarian cancer, no less than 5 additional sections with a thickness of 4-5 μm and 5 mL of whole blood should be collected for the detection of MSI;
4) For urothelial carcinoma treated with combination therapy, no less than 5 additional sections with a thickness of 4-5 μm should be collected for the detection of FGFR2/3 gene abnormality.

**Table 4. Acquisition and collection of biomarker samples.**

| Sample Type | Collection Requirement |
|---|---|
| Screening Period | |
| Previously archived samples | 5 unstained sections with a thickness of 4-5 μm |
| Tumor biopsy samples recommended (before the first dose) | After fixation and embedding, 3 unstained sections with a thickness of 4-5 μm |
| Collection of Additional Tumor Tissue Samples for Specific Tumor Types | |
| For ovarian cancer, endometrial cancer, or urothelial carcinoma treated with combination therapy | No less than 5 additional unstained sections with a thickness of 4-5 μm |
| Collection of Additional Tumor Blood Samples for Specific Tumor Types | |
| For ovarian cancer and endometrial cancer | 5 mL of whole blood |

Refer to the laboratory manual for details on sample acquisition, collection, processing, and transportation methods.

# 3. SELECTION AND WITHDRAWAL OF SUBJECTS

The enrollment of eligible subjects is critical to ensure the outcome of the study. Patients must meet the following criteria to be allowed to participate in this study. Any medical or non-medical conditions of a patient are considered for his/her eligibility.

Before the patient's enrollment in the study, the investigators should review, confirm, and document whether the patient is suitable for participating in the study.

## 3.1. Inclusion Criteria

Patients must meet all of the following inclusion criteria to be eligible for this study.

1. Voluntarily participate in this study and sign the informed consent form (ICF);
2. Male or female aged 18-75 years old;
3. Advanced renal cancer, urothelial carcinoma, cervical cancer, recurrent ovarian cancer, and endometrial cancer:
   - Renal cell carcinoma: Histologically or cytologically confirmed advanced clear cell renal cell carcinoma (in the case of mixed tumors, predominant clear cell renal cell carcinoma is required);

1) Cohort 1: Have been previously treated with at most one targeted anti-angiogenic drug and failed the treatment (if any);
2) Cohort 8: Progression after prior anti-PD-1/PD-L1/CTLA-4 antibody monotherapy or combination therapy (at least 2 doses of anti-PD-1/PD-L1/CTLA-4 antibody);

- Urothelial carcinoma: Histologically or cytologically confirmed unresectable urothelial carcinoma, including renal pelvis cancer, ureteric cancer, bladder cancer, and urethral cancer (in the case of mixed tumors, transitional cell carcinoma as the predominant histological subtype is required) that progressed or recurred after platinum-based therapies and have been treated with no more than 2 previous systemic treatments;

Cohorts 6 and 7: Have received at least one platinum-based therapy in the recurrence/metastasis stage, and the disease progressed or recurred.

Note: Neoadjuvant or adjuvant therapies, with recurrence and progression within 12 months after the end of treatment, are counted as one systemic treatment.

- Cervical cancer: Histologically or cytologically confirmed squamous cell carcinoma of the cervix; for recurrent/metastatic cervical cancer that has been treated with 1 or 2 lines of previous systemic treatment (except radiation-enhanced chemotherapy), recurrence or progression during or after previous treatment is required;

Note: Neoadjuvant or adjuvant therapies (except radiation-enhanced chemotherapy), with recurrence and progression within 1 year after first-line standard surgery, or within 6 months after radiotherapy, are included in the first-line systemic treatment.

- Recurrent ovarian cancer: Histopathologically confirmed recurrent epithelial ovarian cancer, fallopian tube cancer, or primary peritoneal cancer that has been treated with platinum-based therapy and recurred/progressed during or within 6 months after the last platinum-based therapy (completing 4 or more treatment cycles);

Note: No more than 1 non-platinum-based therapy between the last 2 platinum-based therapies; no other anti-tumor treatments except endocrine therapy, PARP inhibitor maintenance therapy, or traditional Chinese medicine and modern Chinese medicinal preparations after the last platinum-based therapy.

- Endometrial cancer: Histopathologically confirmed endometrial cancer (excluding carcinosarcoma) that recurred/metastasized after previous treatment; for recurrent/metastatic cancer that has been treated with 1 or 2 lines of previous systemic treatment (except radiation-enhanced chemotherapy), recurrence or progression during or after previous treatment is required.

4. At least one measurable lesion that meets RECIST v1.1 (must be ≥ 10 mm in long-axis diameter by spiral CT or ≥ 15 mm in short-axis diameter for enlarged lymph nodes, as per RECIST v1.1);
5. Able to swallow tablets;
6. ECOG PS score: 0-1 (refer to Appendix I for ECOG scoring criteria);
7. Life expectancy ≥ 12 weeks;
8. Major organ functions must meet the following requirements (No blood components or growth factor corrective therapy is allowed within 14 days prior to the start of study treatment):

- Absolute neutrophil count ≥ 1.5 × $10^9$/L;
- Platelets ≥ 90 × $10^9$/L;
- Hemoglobin ≥ 90 g/L;
- Serum albumin ≥ 30 g/L;
- Thyroid stimulating hormone (TSH) ≤ 1 × ULN (In case of abnormalities, FT3 and FT4 levels should be measured at the same time. If FT3 and FT4 levels are normal, the subject can be enrolled);
- Bilirubin ≤ 1 × ULN (within 7 days prior to the first dose);
- ALT and AST ≤ 3 × ULN (within 7 days prior to the first dose);
- AKP ≤ 2.5 × ULN (< 5 × ULN if accompanied by bone metastasis);
- Serum creatinine ≤ 1.25 × ULN;

9. Female patients of childbearing potential or female patients who are not sterilized by surgical operations are required to take two medically approved contraceptive measures (such as intrauterine device, oral contraceptive, or condom) during the study treatment period and within 3 months after the end of the study treatment; female patients of childbearing potential who are not surgically sterilized must have a negative serum HCG test result within 72 h prior to enrollment, and must not be on breast-feeding; male patients with partners of childbearing potential should take two effective contraceptive measures during the study and within 3 months after the end of study treatment.

### 3.2. Exclusion Criteria

Patients meeting any of the following criteria will be excluded:

1. Any active autoimmune diseases or history of autoimmune diseases (including but not limited to the following: autoimmune hepatitis, interstitial pneumonia, uveitis, enteritis, hepatitis, hypophysitis, vasculitis, nephritis, and hyperthyroidism; vitiligo; adult patients with completely relieved childhood asthma can be enrolled if no intervention is required; patients with asthma requiring medical intervention with bronchodilators cannot be enrolled);
2. Currently using immunosuppressants, or systemic hormonal therapy for immunosuppression (> 10 mg/day of prednisone or an equivalent dose of other therapeutic hormones) within 2 weeks prior to the first dose;
3. Severe allergic reactions to other monoclonal antibodies;
4. Untreated metastases to central nervous system,
   - Asymptomatic patients who have received prior systemic and radical treatment for metastases to the brain or meninges (radiotherapy or surgery) may be enrolled if they are stable for at least 1 month as confirmed by imaging and have stopped systemic hormonal therapy (> 10 mg/day of prednisone or equivalent) for more than 2 weeks;
5. Hypertension not adequately controlled with antihypertensive therapy (systolic blood pressure ≥ 140 mmHg or diastolic blood pressure ≥ 90 mmHg);
6. Uncontrolled cardiac diseases or symptoms, such as: (1) NYHA Class II or above heart failure, (2) unstable angina, (3) myocardial infarction within the past year, (4) clinically significant supraventricular or ventricular arrhythmia requiring treatment or intervention, or (5) QTc > 450 ms (males) or QTc > 470 ms (females);
7. Abnormal coagulation function (INR > 2.0, PT > 16 s), bleeding tendency or receiving thrombolytics or anticoagulant therapy. Prophylactic use of low-dose aspirin or low molecular weight heparin is allowed;
8. Any Grade ≥ 2 bleeding as per CTCAE v4.03 within 4 weeks before the first dose;
9. Radiographically confirmed significant vascular invasions or a high possibility of significant vascular invasions that may cause fatal bleeding as determined by the investigators during treatment;
10. Events of arterial/venous thrombosis within 6 months prior to the first dose, such as cerebrovascular accidents (including transient ischemic attacks, cerebral hemorrhage, and brain infarction), deep vein thrombosis, and pulmonary embolism;

11. Known hereditary or acquired hemorrhage and thrombophilia (such as hemophilia, coagulopathy, and thrombocytopenia);
12. The routine urinalysis indicates that urine protein is ≥ ++ and confirms that 24 h urine protein is > 1.0 g;
13. Prior chemotherapy which ended (last dose) within 4 weeks prior to the start of this study treatment; prior surgery or palliative radiotherapy within 2 weeks prior to study treatment; molecular targeted therapy (including oral targeted drugs in other clinical trials) within < 5 drug half-lives from the first study dose; or AEs caused by previous treatment (except for alopecia) that have not returned to CTCAE Grade ≤ 1;
14. Radiation-induced enteritis after receiving pelvic radiotherapy within 12 months prior to the study treatment;
15. Active infection, unexplained fever ≥ 38.5 °C within 7 days prior to the study treatment, or baseline white blood cell count > 15 × $10^9$/L;
16. Known history or evidence of interstitial lung disease or non-infectious pneumonitis that has been treated with corticosteroids; or conditions that may interfere with the testing or management of suspected drug-related pulmonary toxicity;
17. Congenital or acquired immunodeficiency (such as HIV positive);
18. Active hepatitis (hepatitis B: positive HBsAg and HBV DNA ≥ 500 IU/ mL; hepatitis C: positive HCV antibody and HCV virus copy number > upper limit of normal);
19. Other malignancies currently or within the past 5 years (except for cured basal cell carcinoma and cervical cancer *in situ*; for recurrent ovarian cancer previously accompanied by breast cancer, patients with no breast cancer recurrence for > 3 years after radical mastectomy can be included);
20. Prior treatment with anti-PD-1/PD-L1 antibodies (except for cervical cancer in Cohort 7 and Cohort 8) or famitinib;
21. Have received live vaccines within 4 weeks before the first dose or may possibly receive live vaccines during the study;
22. Other potential factors that may affect the study results or result in premature discontinuation as determined by the investigators, such as alcoholism, drug abuse, other serious diseases (including mental illness) requiring concomitant treatment, serious laboratory abnormalities, or family or social factors that could affect the safety of the patients.

## 3.3. Lifestyle Requirements

### 3.3.1. Contraception

In this study, camrelizumab (SHR-1210) and famitinib have been suspected of teratogenicity/fetal toxicity, but whether they have transient adverse impact on components of sperm is still unknown. Therefore, for all female subjects of childbearing potential and male subjects with female partners of childbearing potential who receive the study treatment, if the investigators determine that they or their partners are at risk of pregnancy, the subjects and their partners must adopt at least two effective contraceptive measures during the entire treatment period from the signing of the ICF until at least 3 months after the end of the treatment period.

An effective contraception method refers to a method with an annual failure rate of < 1% when correctly used independently or with other methods, including:

1. Commonly used hormonal contraceptive methods associated with the suppression of ovulation (e.g., oral, inserted, injectable, implants, subcutaneous) should meet the requirement that the female subject or partner of male subject has been using this method for a period of time with proven effectiveness, and plans to continue using it correctly throughout the study.
2. Correctly inserted intrauterine devices.
3. Male/female condom combined with topical spermicides (i.e., foams, gels, films, creams, and suppositories).
4. Male sterilization by vasectomy.
5. Bilateral tubal ligation/bilateral salpingectomy or bilateral tubal occlusion (the occlusion should be proven effective by relevant instruments).

### 3.3.2. Exposure to sunlight

Subjects who have undergone treatment should avoid sunbathing or prolonged unprotected exposure to sun.

## 3.4. Discontinuation/Withdrawal of Subjects

### 3.4.1. Criteria for treatment discontinuation

The study treatment must be discontinued when any of the following occurs:

1. Subject requests for discontinuation or withdraws the ICF;
2. Imaging examinations show PD;

As per RECIST v1.1, a confirmation is required 4-6 weeks after the first documentation of PD (except those with rapid progression, with significant clinical progression, or receiving famitinib monotherapy);

Subjects with confirmed PD may continue the treatment if clinically stable (as assessed by the investigators) until further radiographic progression;

Definition of clinically stable: a. no significant clinical symptoms or changes in laboratory tests; b. no changes in the performance status score (deterioration); c. no rapid tumor progression and no progression involving vital organs/sites (e.g., spinal cord compression);

3. Accumulated use of SHR-1210 monotherapy or combination therapy for 2 years (no radiographic progression). Subjects who achieve radiographically confirmed CR may consider discontinuation after 12 cycles of treatment;
4. Unacceptable toxicity;
5. Poor compliance;
6. Loss to follow-up or pregnancy;
7. Other reasons for which the investigators consider a withdrawal necessary.

### 3.4.2. Study withdrawal criteria

Reasons for withdrawal may include:

- Subject withdraws the ICF and refuses further follow-ups;
- Other investigator-assessed reasons requiring withdrawal, such as the inability to provide voluntary consent due to imprisonment or quarantine;
- Loss to follow-up;
- Death;
- Study termination by the sponsor.

### 3.4.3. Procedures for withdrawal from study or treatment discontinuation

The efficacy and safety examinations to be completed upon study withdrawal as specified in the protocol must be completed as much as possible. In addition, the safety follow-up should be completed along with fully documented AEs and their outcomes. The investigators can recommend or provide new or alternative treatments to a subject based on the condition of the subject. Subjects showing no PD need to be continuously followed up for imaging evaluation until the subjects begin new anti-tumor treatment or show PD.

Subject's survival status should still be followed up even when the subject refuses to visit the study site, unless the subject withdraws consent to provide further information or consent to be further contacted. In such case, no study assessment is performed, nor any data are collected. The sponsor can retain and continue to use all data collected before withdrawal of informed consent, unless the subject requests a retraction of collected data.

## 3.5. Premature Termination or Suspension of Study

This study can be terminated prematurely or suspended if there are sufficient reasons. This may result from the decision of regulatory authorities, changes in comments by the ethics committee, efficacy or safety issues of the investigational products, or the judgment of the sponsor. In addition, Hengrui reserves the right to terminate research and development at any time. The party who decides to suspend/terminate the study should notify the investigators, sponsor, and regulatory authorities in writing, documenting the reasons for suspension/termination. The investigators must immediately notify the ethics committee and sponsor, and provide relevant reasons.

The reasons for premature termination or suspension of the study may include:

- Confirmed unexpected, major, or unacceptable risk to the subjects.
- Existing efficacy data supporting premature study termination.
- Poor protocol compliance.
- Incomplete or unmeasurable data.
- Meaningless study results.

The study may continue once those issues related to drug safety, protocol compliance, and data quality have been resolved and approved by the sponsor, ethics committee, or CFDA (now NMPA).

## 3.6. Definition of End of Study

At 6 months after the first dose of the last subject, the primary and secondary endpoints of the study will be statistically analyzed.

All subjects will be followed up until 12 to 24 months after the last dose of the last subject, and supplemental analysis of the primary and secondary endpoints will be performed thereafter.

After the end of study, if the subjects continue to benefit from the investigational products, the treatment can be continued until the criteria for discontinuation or study withdrawal are met. The occurrence of SAEs will be collected and recorded during treatment and after the last dose according to the protocol.

# 4. STUDY MEDICATION

## 4.1. Drug Allocation

After signing the ICF and completing the necessary baseline assessments, subjects will automatically be assigned a subject number, which will be used for all CRFs and study documents. After completing the screening process, subjects will be formally enrolled. The study site staff will send a completed Eligibility Form via email to the study team member designated by the sponsor.

No investigational products will be administered to any subject until the investigators or designee has received the following written information.

- Confirmation of subject enrollment;
- Permission to administer drugs to subjects.

The sponsor or designee will notify other study sites of the enrollment of new subjects and next possible enrollment time.

## 4.2. Dosage Form, Appearance, Packaging, and Label

**Investigational product 1: SHR-1210 injection**

| | |
|---|---|
| Manufacturer: | Suzhou Suncadia Biopharmaceuticals Co., Ltd. |
| Dosage form: | lyophilized powder |
| Strength: | 200 mg (tentative)/20 mL vial. |
| Batch No.: | see label |
| Method of administration: | intravenous infusion |
| Shelf life: | 2 years (tentative) from the date of manufacture. |
| Storage conditions: | sealed, away from light, stored at 2-8 °C in medical refrigerator. Do not freeze. |

**Investigational product 2:** famitinib malate capsules

| | |
|---|---|
| Manufacturer: | Jiangsu Hengrui Pharmaceuticals Co., Ltd. |
| Dosage form: | capsule |
| Strength: | 15 mg/capsule; 20 mg/capsule; 25 mg/capsule |
| Batch No.: | see label |
| Method of administration: | oral |
| Shelf life: | 2 years (tentative) from the date of manufacture. |
| Storage conditions: | sealed, away from light, stored at room temperature |

### 4.2.1. Packaging and labeling

**Packaging:**

Packaging strength and quantity for SHR-1210: 200 mg vial, 1 vial per box, and 20 boxes per carton.

Packaging strength and quantity for famitinib: Famitinib is available in 3 strengths, 25 mg/capsule, 20 mg/capsule, and 15 mg/capsule. Drugs with the same strength are packaged in the same vial. Do not package different strengths in the same vial. Each vial should contain 24 famitinib capsules, which equals to the quantity for one treatment cycle.

**Labeling:**

The labeling of the investigational products follows relevant guidelines in Good Clinical Practice (GCP). The contents of the label include but are not limited to: clinical approval number, name of study, drug name, drug number, packaging strength, manufacturing batch number, shelf life, route of administration and dose, and storage conditions. "For Clinical Use Only" should also be noted on the label.

Label of SHR-1210 (the actual label shall prevail), the drug number is increased incrementally from 0001.

The label content includes:

| | |
|---|---|
| Label name: | SHR-1210 injection - investigational product for clinical study |
| Study no.: | SHR-1210-II-213 |
| Strength: | lyophilized powder for injection, 200 mg/vial |
| Indications: | advanced urinary system tumors or gynecological tumors |
| Method of administration: | 200 mg, q3w, intravenous infusion |

Drug number, packaging quantity, storage conditions, batch number, shelf life, sponsor (Jiangsu Hengrui Pharmaceuticals Co., Ltd.), and manufacturer (Suzhou Suncadia Biopharmaceuticals Co., Ltd.)

Precautions: For clinical study only. Prepare according to the pharmacy manual

Label of famitinib (the actual label shall prevail)

The label content includes:

| | |
|---|---|
| Label name: | famitinib malate capsules - investigational product for clinical study |
| Study no.: | SHR-1210-II-213 |
| Strength: | 25 mg/capsule, 20 mg/capsule, or 15 mg/capsule; quantity: 24 capsules/bottle |
| Indications: | advanced urinary system tumors or gynecological tumors |
| Method of administration: | 1 capsule per dose, qd, orally administered after meals |

Drug number, storage conditions, batch number, shelf life, study site (Jiangsu Hengrui Pharmaceuticals Co., Ltd.)

Precautions: For clinical study only; please return the remaining drug to the physician.

## 4.3. Storage of Investigational Products

The investigators or the authorized representative thereof (e.g., pharmacist) will ensure that all investigational products are stored in a secure and access-controlled area conforming to storage conditions and regulatory requirements.

SHR-1210 should be stored in its original container and match with the labels. Once prepared or diluted, the product should be stored according to the storage conditions specified in the pharmacy manual.

The study site personnel should provide subjects with the proper storage instructions (for famitinib) based on the pharmacy manual.

## 4.4. Drug Preparation, Dispensation, and Return

SHR-1210 should be prepared by qualified or experienced study personnel, such as physicians, pharmacists, and medical assistants (approved by national authorities or study site operating guidelines) according to the pharmacy manual.

Refer to the SHR-1210 pharmacy manual for blending, concentration (preparation), and administration of the injection. Since this product does not contain any antimicrobial preservatives or bacteriostatic agents, care must be taken to ensure that the preparations are sterile.

Please refer to the pharmacy manual for details on storage of prepared medication at room temperature/under light and in the refrigerator.

Expired or remaining drug solutions must be disposed.

Famitinib should be dispensed and returned by cycle. When the subject returns to the hospital for a visit on D1 (± 3 d) of each cycle, the remaining drugs should be retrieved to verify the actual dose administered before new drugs are dispensed.

### 4.4.1. Disposal of investigational products

The sponsor or its authorized personnel will provide guidance on the destruction of unused investigational products. If the study site is authorized to destroy the investigational products, the investigators must ensure that the destruction of the investigational products complies with applicable environmental regulations and company policies, and provide relevant procedures for destruction. The investigators should record all destruction activities.

## 4.5. Administration of investigational products

SHR-1210 is an intravenous injection. It must be used in the outpatient or ward of the study site by qualified or experienced study personnel and must not be used outside of a study site.

SHR-1210 is intravenously infused over 30 min (not less than 20 min and not more than 60 min, including flushing). Do not administer through intravenous bolus or rapid bolus injection.

The intravenous infusion should be performed through a medical infusion bag using an infusion set with an in-line filter (0.2 μm).

Do not administer other medications with this venous access before or after the infusion.

Famitinib is an oral capsule. The investigators will prescribe the medicine and the subjects should take the medicine at home. Refer to the dosing regimen and pharmacy manual for details.

### 4.5.1. Precautions for special drug delivery devices

Intravenous infusion bags, diluent, and infusion tubing with micron filter (i.e., 0.2/1.2 μm; please refer to the pharmacy manual for details of all required filters) should be prepared at the study site.

## 4.6. Dosing Regimen

For subjects in Cohorts 1-6 and 8 and for subjects who continue treatment after PD in Cohorts 6 and 7, SHR-1210 will be administered via intravenous infusion at a fixed dose of 200 mg within 30 min (not less than 20 min, not more than 60 min), once every 3 weeks. Each cycle contains 3 weeks and the longest dosing period is 2 years.

During the study, SHR-1210 will be administered once every 3 weeks. The time window is ± 7 days from the scheduled administration time. If an SHR-1210 dose is delayed beyond the scheduled administration time, then the dose should be skipped, and the administration should be resumed at the original dose at the next scheduled time point.

For subjects in Cohorts 1-5 and 8 and for subjects who continue treatment after PD in Cohort 6, famitinib malate capsules should be administered once a day before or after a meal (recommended at a fixed time: within 0.5 h after a meal) at a dose of 20 mg. The drug should be administered continuously in cycles of 3 weeks.

For subjects in Cohort 7, famitinib malate capsules should be administered once a day before or after a meal (recommended at a fixed time: within 0.5 h after a meal) at a dose of 25 mg. The drug should be administered continuously in cycles of 3 weeks. In Cohort 7, the dose and frequency of famitinib for subjects who continue treatment after PD are the same as those before PD. If the dose of famitinib before PD is 25 mg, the dose of famitinib should be adjusted to 20 mg (continuous administration) for continuing treatment.

Subjects in Cohorts 7 and 8 who participate in the blood sampling for famitinib PK analysis should take famitinib after blood sampling before C2D1 and C3D1 administration. Subjects in the PK study should take famitinib orally before breakfast on the day of blood sampling for PK study.

Subjects continue to use the investigational products until the criteria for discontinuation specified in the protocol are met, or until withdrawal from study.

## 4.7. Dose Modification

The study treatment may be modified according to the toxic side effects that appeared. The options for treatment modification include: interruption, dose reduction, change in method of administration, and discontinuation;

In this study, SHR-1210 treatment may be interrupted for up to 12 consecutive weeks;

Dose modifications caused by famitinib-related toxicity include: dose interruption, dose reduction (e.g., reduction to 15 mg/d from the starting dose of 20 mg/d; reduction to 20 mg/d and again to 15 mg/d from the starting dose of 25 mg/d), change in method of administration (14 days on and 7 days off in each cycle), and discontinuation. A reduced dose or changed method of administration for famitinib should never be resumed for the subject.

If a famitinib-related AE occurs during the study, such as white blood cell count decreased, neutrophil count decreased, platelet count decreased, hypertension, proteinuria, or hand-and-foot syndrome, famitinib may be interrupted, and can be resumed, administered in a modified dose or method, or discontinued accordingly when the toxicity resolves. Subjects may continue SHR- 1210 as monotherapy if famitinib treatment is discontinued.

The dose modification of famitinib should be carried out according to the following procedure as much as possible: treatment interruption → dose reduction → change in method of administration (15 mg/d, 14 days on and 7 days off in each cycle). If famitinib-related SAEs still occur after the above change in method of administration has been made, famitinib is not well- tolerated. At the same time, if determining the subject can benefit from continuing use of the investigational products, the investigators should discuss further modifications or famitinib discontinuation with the sponsor.

For immune-related toxicity occurring during the study, such as immune-related pneumonia, hepatitis, and colitis, the dosing of SHR-1210 and famitinib should be interrupted as appropriate. The dosing can be resumed when the toxicity returns to Grade ≤ 1 or baseline levels (for subjects with elevated ALT/AST, TBIL, and other laboratory measurements). The dosing of SHR-1210 should be resumed first. The dosing of famitinib can be resumed when no significant abnormality is observed within 1-2 weeks after SHR-1210 administration. The dose and method of subsequent administration of famitinib can be modified.

During the study, in case of Grade ≥ 3 immune-related pneumonia (first onset), Grade ≥ 3 TBIL increased (recurrent), Grade 4 ALT/AST increased (recurrent), and other Grade 4 immune- related toxicities, Grade 4 infusion reactions, or the interruption of SHR-1210 administration for more than 12 weeks due to immune-related toxicity which can still not return to Grade ≤ 1 or baseline levels, permanent discontinuation of SHR-1210 may be considered.

After discontinuation of SHR-1210, the subject may continue oral administration of famitinib as monotherapy, if the investigators determine that the subject can benefit from such treatment.

If Grade 3 or greater capillary endothelial proliferation occurs during the study, no dose modifications are required for famitinib, but SHR-1210 should be interrupted until the toxicity returns to Grade ≤ 2.

If signs/symptoms or laboratory abnormalities are observed, symptomatic treatment should be provided immediately. Refer to the following recommendations for dose modifications:

## Table 5. Dose modifications.

| Treatment-Related Toxicity | | Grade | Whether to Interrupt Treatment | | Criteria for Resuming | Dose Modification for Famitinib | Criteria for Discontinuation |
|---|---|---|---|---|---|---|---|
| | | | SHR-1210 | Famitinib | | | |
| SHR-1210- and Famitinib-Related Toxicity | Hematologic toxicity | Grade 1-2 | No | No | — | — | — |
| | | Grade 3 | No | Yes | Until the toxicity returns to Grade ≤ 2 | Resume at original dose | Discontinue famitinib if Grade 3 or greater hematologic toxicities recur after two modifications |
| | | Grade 4 | Yes | Yes | Until the toxicity returns to Grade ≤ 2 | Reduce the dose to 15 mg/d, or treat for 14 days and hold for 7 days | |
| | pneumonitis | Grade 2 | Yes | Yes | Until the toxicity returns to Grade ≤ 1 | First onset: Resume at original dose<br>Reduce the dose to 15 mg/d, or treat for 14 days and hold for 7 days | Interruption > 12 weeks without return to Grade ≤ 1 |
| | Elevation in ALT/AST and TBIL (after hepatoprotective treatment) | Grade 2 ALT or AST increased<br>Grade 2 bilirubin increased | Yes | Yes | Until the toxicity returns to baseline level | Reduce the dose to 15 mg/d, or treat for 14 days and hold for 7 days | 1. Discontinue SHR- 1210 treatment after > 12 weeks of interruption without return to baseline level; |
| | | Grade 3/4 ALT/AST increased (first onset)<br>Grade 3 TBIL increased (first onset) | Yes | Yes | Until the toxicity returns to baseline level | Reduce the dose to 15 mg/d, or treat for 14 days and hold for 7 days | 1. Discontinue SHR- 1210 treatment after > 12 weeks of interruption without return to baseline level;<br>2. Discontinue SHR- 1210 treatment if Grade 3 ALT/AST increased recurs |

| **Treatment-Related Toxicity** | | **Grade** | **Whether to Interrupt Treatment** | | **Criteria for Resuming** | **Dose Modification for Famitinib** | **Criteria for Discontinuation** |
|---|---|---|---|---|---|---|---|
| | | | **SHR-1210** | **Famitinib** | | | |
| | Other non- hematologic toxicities (immune-related) | Grade 1 | No | No | — | — | — |
| | | Grade 2 (last ≥ 7 days) | Yes | Yes | Until the toxicity returns to Grade ≤ 1 | Resume at original dose | Discontinue SHR- 1210 treatment after > 12 weeks of interruption without return to Grade ≤ 1 |
| | | Grade 3 | Yes | Yes | Until the toxicity returns to Grade ≤ 1 | Reduce the dose to 15 mg/d, or treat for 14 days and hold for 7 days | |
| SHR- 1210-Related Toxicity | Reactive cutaneous capillary endothelial proliferation | Grade 3 | Yes | No | Until the toxicity returns to Grade ≤ 2 | Resume at original dose | SHR-1210 interruption for more than 12 weeks |
| Famitinib-Related Toxicity | Hypertension | Grade 3 (after corrective treatment) | No | Yes | Until the toxicity returns to Grade ≤ 1 | First onset: Resume at original dose Second onset of Grade 3 hypertension: Reduce the dose to 15 mg/d, or treat for 14 days and hold for 7 days | Discontinue famitinib treatment if Grade 3 hypertension recurs after re-modification |
| | | Hypertensive crisis | Yes | Yes | Until the toxicity returns to Grade ≤ 1 | Permanently discontinue famitinib treatment | Discontinue famitinib treatment |
| | Proteinuria (without significant increase in blood creatinine) | Grade 3 (24-h urine protein quantitation) | No | Yes | Until the toxicity returns to Grade ≤ 2 | Reduce the dose to 15 mg/d, or treat for 14 days and hold for 7 days | Discontinue famitinib treatment if Grade 3 proteinuria recurs after re-modification |
| | Hand-and-foot syndrome | Grade 3 | No | Yes | Until the toxicity returns to Grade ≤ 1 | Reduce the dose to 15 mg/d, or treat for 14 days and hold for 7 days | Discontinue famitinib treatment if Grade 3 hand-and-foot syndrome recurs after 2 modifications |
| | Headache | Grade 2 headache lasting ≥ 7 days despite symptomatic treatment, or Grade 3 headache | No | Yes | Until the toxicity returns to Grade ≤ 1 | Reduce the dose to 15 mg/d, or treat for 14 days and hold for 7 days Second onset: treat with famitinib for 7 days and hold for 7 days | Discontinue famitinib treatment if the event recurs after re-modification |

The investigators may consider interrupting the treatment for subjects who experience persistent and significant toxicities despite symptomatic treatment, including persistent Grade 2 non-hematologic toxicities (except for asymptomatic Grade 2 hypertension) lasting 2 weeks or longer, and abnormal laboratory findings (except < 2 g/24 h proteinuria). After the toxicity resolves, the dose or method of subsequent famitinib administration may be modified accordingly.

In the course of the study and based on the above regulations for dose modification, the investigators may modify the dose appropriately by comprehensively considering the drug-related toxicity in the subjects (if a subject experiences multiple Grade 2 drug-related toxicity and shows poor tolerance to the drugs, the dose and method of administration of famitinib can be modified after treatment interruption and toxicity recovery).

During the study, if a subject has fever (> 38 °C) and needs to use medications for corrective treatment, or in case of obvious wheezing, dyspnea, or symptoms of suffocation, the administration of SHR-1210 should be skipped for the current or next scheduled time of SHR-1210 administration before the symptoms are recovered. After the symptoms are relieved and become stable for more than 7 days, the subjects are given SHR-1210 according to the subsequent dosing schedule. The possibility of pneumonia should be eliminated by imaging examinations before drug administration if necessary.

Once hypertensive crisis, cerebral hemorrhage, other Grade ≥ 3 hemorrhage, arterial thrombosis, Grade 4 venous thrombosis, leukoencephalopathy syndrome, or gastrointestinal perforation occurs during the study, the administration of famitinib should be discontinued, the administration of SHR-1210 should be interrupted, and active symptomatic treatment should be given. The resumption of SHR-1210 treatment will depend on the toxicity recovery.

## 4.8. Concomitant Treatment

Concomitant treatment refers to other treatment that is given for the benefit of subjects as determined by the investigators.

All concomitant medications and treatments within 30 days prior to the start of study treatment and during the study must be documented in the eCRF in strict accordance with the GCP.

Once a subject discontinues the study treatment, only concomitant medications or treatments for new or unresolved treatment-related AEs are recorded, until at least 30 days after the last dose.

### 4.8.1. Other anti-tumor/cancer or investigational products

Other anti-tumor treatments are not permitted when the subject is receiving treatment with the investigational products.

Subjects are not permitted to take anti-tumor traditional Chinese medicine concurrently: Huatan Huisheng tablets, Brucea javanica oil soft capsules, Mandarin melon berry syrup, cantharidin, cinobufotalin, bufotoxin, Kang'ai Injection, Kanglaite, Sarcandra glabra injection, Aidi injection, Awei Huapi cream, Kangaiping pills, Fukang capsules, Xiaoaiping, Pingxiao capsules, Pingxiao tablets, Shendan Sanjie capsules, Ankangxin capsules, Bosheng'aining, Zedoary turmeric oil and glucose injection, Kanglixin capsules, Cidan capsules, GFL tablets, Huai'er granules, Delisheng injection, and other TCM preparations with "anti-tumor" effect described in the package insert.

Palliative radiation is permitted for the treatment of painful bone lesions, provided that these lesions existed prior to enrollment and the investigators must clearly specify that the palliative radiation does not indicate PD. Subjects can receive bisphosphonate for the treatment of bone metastases. If systemic or local pain-relieving medicines are not effective in controlling painful lesions of bone metastasis, palliative radiotherapy is allowed.

Palliative treatment for lesions outside the lungs and liver is allowed during the study (when the treatment of the subjects is needed to improve symptoms upon the onset of PD), and the treatments include the treatments for hydrothorax and ascites. During treatment, the subjects should suspend the administration of the investigational products until the end of the recovery period of palliative treatment.

### 4.8.2. Supportive care

Palliative and supportive care for disease-related symptoms will be based on the investigators' judgment and relevant guidelines. The best oral anti-viral drug for the treatment of hepatitis B is permitted during the study, but interferon is prohibited.

Subjects should be given optimal supportive care during treatment. Comorbidities and various adverse reactions, especially immune-related adverse reactions, should be actively treated. Oral administration of megestrol acetate for promoting appetite during treatment is permitted for subjects in the renal cancer, urothelial carcinoma, and cervical cancer cohorts. Oral administration of megestrol acetate for promoting appetite is not allowed for subjects in the endometrial cancer and ovarian cancer cohorts.

### 4.8.3. Immunological agents

The concurrent use of thymalfasin, interferon, interleukin-2, and other immunological agents is not allowed.

### 4.8.4. Drugs that may have drug-drug interactions with famitinib

*In vitro* studies have shown that famitinib is metabolized by various liver P450 enzymes and may be affected by strong CYP3A4 inducers or inhibitors. Strong CYP3A4 inhibitors (ketoconazole, itraconazole, erythromycin, and klacid) should be prohibited during treatment.

### 4.8.5. Drugs that prolong the QT interval of the heart

As tinib drugs may cause toxicities of prolonged QT interval in clinical applications, drugs that may prolong the QT interval should be used with caution during the study. These mainly include but are not limited to the following categories of drugs:

- Antibiotics: fluoroquinolones: sparfloxacin, gatifloxacin, levofloxacin, moxifloxacin, ofloxacin, ciprofloxacin; macrolides: erythromycin, clarithromycin, telithromycin, azithromycin, roxithromycin, metronidazole
- Antiarrhythmics: quinidine, procainamide, disopyramide, flecainide, propafenone, amiodarone, dronedarone, sotalol, dofetilide, ibutilide
- Drugs used to relieve angina pectoris: ranolazine, ivabradine
- Antipsychotics: risperidone, fluphenazine, droperidol, haloperidol, thioridazine, pimozide, olanzapine, clozapine
- Antifungal drugs: voriconazole, posaconazole
- Antimalarial drugs: mefloquine, chloroquine
- Antihistamines: terfenadine, astemizole, hydroxyzine
- Gastrointestinal drugs: antiemetics: ondansetron, granisetron, dolasetron, droperidol (0.625 to 1.25 mg may be a safe dose), hydroxyzine; prokinetics: cisapride, domperidone, metoclopramide
- Antidepressants: amitriptyline, imipramine, clomipramine, dosulepin, doxepin

#### 4.8.6. Surgery or palliative radiotherapy

Any surgery or palliative radiotherapy conducted during the study period must be reasonable and necessary. The interval between the treatment and drug must not affect the recovery of the wound as much as possible and the investigation on hemorrhage of unknown cause. It is recommended that the investigational products be suspended 7 days before surgery or palliative radiotherapy, during premedication, and at least 7 days after surgery/radiotherapy. Resumption in subjects who undergo surgery depends on the clinical assessment of wound healing and postoperative recovery.

# 5. STUDY PROCEDURES

Before the study commences, the subjects must read and sign the current ICF approved by the ethics committee (EC). All examinations and study procedures will be carried out according to the Schedule of Activities, and will not be affected by the duration of drug interruption. However, changes outside the allowable window are permitted due to holidays or other management reasons.

## 5.1. Screening

Unless otherwise stated, the following screening procedures must be completed within 21 days prior to the first dose:

**[Signing of informed consent]** A written ICF must be signed by the subject before any procedures of the clinical study are carried out;

**[Demographics]** Gender, date of birth, ethnicity, height, and weight;

**[Tumor history]**

(1) Tumor diagnosis: clinical diagnosis prior to enrollment, date of initial pathological diagnosis, initial pathological diagnosis, pathological grade, presence of distant metastasis, and clinical staging;

(2) History of surgery: the surgical history of primary lesions (name, date), and the surgical history of metastatic lesions (name, date);

(3) History of systemic treatment: treatment mode - neoadjuvant therapy, adjuvant therapy, or palliative therapy; drug name in treatment regimen, start date, end date, best response, and treatment outcome; treatment outcome: relapse after treatment; progression and recurrence during treatment; intolerable toxicity. Time of recurrence and progression or specific name and grade of intolerable AE should be recorded;

(4) History of radiotherapy: name of radiotherapy, date, dose, site (total body/local); seed implantation (name, date);

**[Medical history]** Name of disease, date of diagnosis, name of medication, persistence, history of cancers other than the cancer types specified in the protocol;

**[Concomitant medication]** Concomitant medication and treatment received within 30 days prior to the first dose and during the study period should be recorded. Once a subject discontinues the study treatment, only concomitant medication and treatment for new or unresolved treatment-related AEs should be recorded, until at least 30 days after the last dose. If new anti-tumor treatment starts within 30 days, only concomitant medication for treatment-related AEs will be documented;

**[Thyroid function]** FT3, FT4, and TSH;

**[Pituitary adrenal axis test]** Including ACTH, cortisol, and follicle stimulating hormone;

**[HIV test]** HIV antibody test;

**[Hepatitis B and hepatitis C tests]** Subjects with abnormal HBsAg results should undergo quantitative test of HBV DNA. Subjects with positive anti-HCV antibodies must be tested for HCV RNA;

**[Adverse events]** AEs should be recorded from the signing of informed consent until at least 30 days after the last dose and followed up until the AEs are resolved or stabilized. All AEs suspected to be related to SHR-1210 should be collected from 30 to 90 days after the last dose. If no new anti-tumor treatment starts during the period, all SAEs should be collected; if new anti- tumor treatment starts, only SAEs related to the investigational products will be collected after the start of the new anti-tumor treatment;

**[Imaging examination]** Including contrast-enhanced CT or MRI of the chest, abdomen, and pelvis. A contrast-enhanced MRI or CT of the brain must also be performed for those suspected of brain metastasis to rule out brain metastasis. Tumor evaluation performed within 3 weeks prior to treatment can be accepted as the baseline evaluation. CT/MRI results prior to informed consent may be used for tumor evaluation at screening if requirements are met. Patients with suspected bone metastasis must undergo a bone scan and those with bone lesions need to undergo CT/MRI for confirmation;

**[Acquisition/collection of biomarker samples]** For subjects receiving SHR-1210 combination therapy or monotherapy, existing paraffin-embedded tumor tissue sections should be collected and 5 tumor sections with a thickness of 4-5 µm should be used for PD-L1 detection. It is recommended to collect biopsy specimens (core needle biopsy) prior to the first dose and prepare 3 tumor sections with a thickness of 4-5 µm after fixation and embedding for PD-L1 detection. For endometrial cancer and ovarian cancer, no less than 5 additional sections with a thickness of

4-5 μm will be collected and 5 mL of whole blood will be collected for the detection of MSI. If failure to collect 5 mL of whole blood at screening, then collect it at a subsequent or unscheduled visit. For urothelial carcinoma treated with combination therapy, no less than 5 additional sections with a thickness of 4-5 μm will be collected for the detection of FGFR2/3 gene abnormality.

Refer to the laboratory manual for tumor sample acquisition/collection, transportation, and processing methods.

Unless otherwise stated, the following screening procedures must be completed within 7 days prior to the first dose:

**[Hematology]** White blood cell count (WBC), absolute neutrophil count (ANC), lymphocyte count (LYM), red blood cell count (RBC), hemoglobin (Hb), and platelet count (PLT);

**[Urinalysis]** Urine protein, urine occult blood, urine red blood cells, and urine white blood cells. During the screening period, if semi-quantitative test shows urine protein of ≥ 2+, a quantitative 24-h urine protein test is required. Subsequently, if semi-quantitative tests from 2 consecutive follow-ups show protein of 2+, a quantitative 24-h urine protein test is required, and if semi- quantitative test shows protein of > 2+, a quantitative 24-h urine protein test should be performed;

**[Routine stool test]** Subjects with positive fecal occult blood must be retested. If fecal occult blood is confirmed after retest, by combing medical history, a gastroscopy should be performed when necessary;

**[Blood biochemistry]** TBIL, direct bilirubin, ALT, AST, AKP, γ-GT, LDH, total protein, albumin, urea/blood urea nitrogen, creatinine, uric acid, fasting blood glucose, triglyceride, cholesterol, potassium, sodium, chlorine, calcium, phosphorus, blood lipase (only during the screening period and in case of subsequent abdominal pain, abdominal distension, and other symptoms of suspected pancreatitis), and blood amylase (only during the screening period and in case of subsequent abdominal pain, abdominal distension, and other symptoms of suspected pancreatitis);

**[Coagulation function]** Including INR, APTT, PT, and FIB;

**[Myocardial zymography]** Including creatine kinase and lactate dehydrogenase;

**[Pregnancy test]** A serum pregnancy test should be performed on women of childbearing potential within 72 h prior to the first dose. Additional tests may be performed to rule out pregnancy if indicated;

**[Vital signs]** Body temperature, pulse, respiratory rate, and blood pressure;

**[Blood pressure monitoring]** The blood pressure of subjects will be measured by the investigators or study nurse in the screening period. At each blood pressure measurement, smoking and coffee are prohibited within 30 min before measurement, and subjects should rest for at least 10 min. The sitting position will be taken at measurement by placing the elbow at the same level as the heart. Each blood pressure measurement should be taken on the same side of the body;

**[Physical examination]** General conditions, head and face, skin, lymph nodes, eyes (scleras, pupils), ears, nose, throat, mouth, respiratory system, cardiovascular system, abdomen (including liver and spleen), reproductive and urinary systems, musculoskeletal system, nervous system, and mental state. Note: Comprehensive physical examinations must be performed during the study, but only abnormal findings need to be documented in the eCRF. Repeated documentation is not required if there is no change from baseline;

**[ECOG PS score]** See Appendix I;

**[12-lead ECG]** ECG examination within 7 days before the first dose in the screening period will be performed for 3 consecutive times at an interval of about 5 min. The average value of the 3 QTc results is taken. The average value of the 3 QTc results of the enrolled subject must meet the inclusion and exclusion criteria. If the ECG is abnormal (with clinical significance) during the study, another two examinations must be performed, and additional tests must be performed when necessary as determined by the investigators;

**[Echocardiography]**

**[Ultrasonography of lower extremity veins]** B-mode ultrasonography of the deep veins of both lower extremities should be performed within 7 days prior to the first dose during the screening period.

The inclusion and exclusion criteria will be verified again. Subjects must meet all inclusion criteria and must not meet any of the exclusion criteria before they can be enrolled in the study.

## 5.2. Enrollment

After completing all medical history and laboratory tests during the screening period, the inclusion and exclusion criteria will be verified again. Patients must meet all inclusion criteria and must not meet any of the exclusion criteria before they can be enrolled in the study.

Additional eligible subjects with urothelial carcinoma (subjects not enrolled in Cohort 2) will be randomized to Cohort 6 or 7 to receive the corresponding investigational products after screening.

## 5.3. Treatment Period

C1D1 [Vital signs] [Physical examination and weight measurement] [SHR-1210 intravenous infusion (if applicable)] [Famitinib dispensation (if applicable)] [Famitinib administration (if applicable)]

Within 24 h after the first dose of SHR-1210, the subjects should be closely monitored for acute allergic reactions. If an acute allergic reaction occurs, it should be treated according to the medical practice of the hospital and relevant guidelines.

In order to improve the patient compliance, only SHR-1210 is administered on C1D1, and famitinib is orally administered starting from D2 before or after a breakfast (if applicable). Daily oral administration at a fixed time is recommended.

C1D7 (± 1 d): [Hematology] [Blood biochemistry] [Urinalysis] [Coagulation function] [Vital signs] [Physical examination] [ECG] [Adverse events] [Concomitant medication]

C2D1 (± 3 d): [Hematology] [Blood biochemistry] [Urinalysis] [Coagulation function] [Routine stool test] [Thyroid function] [Vital signs] [Physical examination and weight measurement] [ECOG PS score] [ECG] [SHR-1210 intravenous infusion (if applicable)] [Dispensation/return of famitinib] [Adverse events] [Concomitant medication]

D1 of C3-C35 (± 3 d): [Hematology] [Blood biochemistry] [Urinalysis] [Coagulation function] [Vital signs] [Physical examination and weight measurement] [ECOG PS score] [ECG] [SHR-1210 intravenous infusion (if applicable)] [Adverse events] [Concomitant medication] [Dispensation/return of famitinib]; **[Thyroid function test]** will be performed on D1 ± 7 d of every 3 cycles;

During each cycle, SHR-1210 should be administered via intravenous infusion after the evaluation of examinations and tests specified in the Schedule of Activities is completed.

**[Imaging evaluation]** in the treatment period: Imaging examinations in the treatment period should be performed every 3 cycles under the same conditions as those of the baseline examination (slice thickness, use of contrast agent, etc.). Bone scan will be performed for suspected bone progression or CR confirmation. Contrast-enhanced MRI or CT of the brain must also be performed for suspected brain metastasis. Tumor evaluation may also be performed if new lesions are suspected. The first documentation of PR/CR in a subject must be confirmed 4 weeks + 7 days later. An imaging examination for confirmation is required 4-6 weeks after the first documentation of PD as per RECIST v1.1 (except those with rapid progression or significant clinical progression). The window period for imaging examination schedule is ± 7 days. Unscheduled imaging examinations can be performed when PD is suspected (such as worsening of symptoms).

**[Blood pressure monitoring]** in the treatment period: During treatment, blood pressure will be measured by subjects themselves (subjects in Cohort 6 are not required to measure blood pressure at home) and recorded in their diary cards. Blood pressure should be measured at least 3 times per week during the first 2 cycles. For subjects with abnormal blood pressure, blood pressure should be measured daily; if normal, blood pressure should be measured at least twice per week after Cycle 2. The investigators or study nurse will also measure blood pressure at each visit.

The first 12 subjects enrolled in Cohorts 1-5 will undergo **[Blood sampling for PK and ADA analysis]** in the treatment period. Subjects in Cohort 7 will undergo **[Blood sampling for PK analysis]** of famitinib (only at sites with conditions for PK blood sampling and processing).

- The first 12 subjects enrolled in Cohorts 1-5:

For SHR-1210 PK analysis, blood samples will be collected within 30 min before administration and within 5 min after administration (including flushing) of the first SHR-1210 dose; within 30 min before administration and within 5 min after administration (including flushing) of SHR-1210 on administration days of Cycles 2, 3, and 4; within 30 min pre-administration every 4 cycles thereafter; at the end of SHR-1210 treatment (the documented time of last SHR-1210 administration prior to the end of treatment); at 30 days after the end of SHR-1210 treatment. (If subject completes the treatment before scheduled blood sampling is completed, the analysis should be based on actual completed sampling).

ADA blood samples will be simultaneously collected along with PK samples before each administration, at the end of treatment, and at 30 days after the end of treatment.

For SHR-1210, 4 mL should be collected for PK analysis and 4 mL for ADA analysis at each blood sampling point. The serum should be separated.

For famitinib PK analysis, 3 mL of blood sample will be collected on D1 (± 3 d) of Cycle 3 at each of the following time points: within 30 min pre-administration, and at 2 h ± 5 min, 4 h ± 5 min, 6 h ± 5 min, 8 h ± 5 min, 10 h ± 5 min, and 24 h ± 30 min post-administration (before D2 administration), and the plasma will be separated.

- Cohorts 7 and 8:

For famitinib PK analysis, 3 mL of blood sample will be collected (only at sites with conditions for PK blood sampling and processing) at each of the following time points: at 6 (± 1) h post-administration on C1D1, within 30 min pre-administration and at 6 (± 1) h post-administration on C2D1 and C3D1, and the plasma will be separated. If famitinib administration is interrupted on the day of sampling, pre-administration sampling should be continued but post-administration sampling should be skipped. On the sampling day, it is also necessary to record the previous administration time.

Famitinib will be administered under fasting condition on the days of blood sampling. That is, food but not water is prohibited after dinner the day before blood sampling. Subjects will take famitinib capsules orally before breakfast on the day of blood sampling and will be fasted for food but not water within 2 h after administration.

## 5.4. End of Treatment/Withdrawal

The treatment should be discontinued if events specified in "Section 3.4.3. Criteria for Discontinuation" occur. At the end of the study treatment or upon withdrawal from the study, if a subject has not undergone examinations within 14 days prior to the end of the study, the subject should undergo the following procedures:

[Hematology] [Blood biochemistry] [Urinalysis] [Routine stool test] [Coagulation function] [Pregnancy test] [Thyroid function test] [Myocardial zymography] [Vital signs] [Physical examination] [ECOG PS score] [ECG] [Echocardiography] [B-mode ultrasonography of lower extremity veins] [Blood pressure monitoring] [Adverse events] [Concomitant medication] [Famitinib return]

If a subject has not undergone imaging examinations within 4 weeks prior to the end of the study, the subject should undergo an imaging examination for response evaluation at the end of the study treatment or upon withdrawal from the study. For subjects with non-radiographic progression (intolerability, other conditions), a tumor evaluation should be performed once every 3 months (± 7 d) after the end of the study treatment until PD, death, or initiation of other anti- tumor treatments.

In addition, the first 12 subjects enrolled in Cohorts 1-5 will undergo blood sampling for SHR- 1210 PK and ADA analysis at the end of SHR-1210 treatment, 4 mL each.

## 5.5. Follow-Up Period

30 ± 3 d after the last dose

[Vital signs] [Physical examination] [ECOG PS score] [Hematology] [Urinalysis] [Blood biochemistry] [Adverse events] [Concomitant medication]

The first 12 subjects enrolled in Cohorts 1-5 will undergo blood sampling for SHR-1210 PK and ADA analysis at 30 days after the end of SHR-1210 treatment, 4 mL each.

All AEs suspected to be related to SHR-1210 should be collected from 30 to 90 days after the last dose. If no new anti-tumor treatment starts during the period, all SAEs should be collected; if new anti-tumor treatment starts, only SAEs related to the investigational products will be collected after the start of the new anti-tumor treatment.

[Survival follow-up] After the end of treatment, the survival status and subsequent anti-tumor treatment can be collected through clinical or telephone follow-up every 2 months (± 7 d) until death.

## 5.6. Continuing Treatment After Progressive Disease

### 5.6.1. Criteria for continuing treatment

Subjects in Cohort 6 (SHR-1210 monotherapy group) and Cohort 7 (famitinib monotherapy group) who can benefit from continuing treatment with the investigational products after PD (radiographic) as per the investigators' judgment may continue the combination therapy with the investigational products (SHR-1210 combined with famitinib) after discussion between the investigators and the sponsor and on the premise of fully informed consent of the subjects, until protocol-specified criteria for discontinuation are met. Subjects whose treatment is discontinued for non-PD reasons will directly proceed to the end-of-treatment visit and subsequent follow-ups.

The visit cycle and efficacy evaluation of subjects who continue treatment after PD are the same as those before PD. AEs/SAEs and concomitant medications/treatments that occur during the period of continuing treatment after PD and within 30 days after the last dose should be recorded and reported in accordance with the time limit (dosing time limit) specified in the protocol.

Subjects in Cohorts 6 and 7 who choose to continue treatment after PD need to meet the following criteria:

- With radiographically confirmed PD as per RECIST v1.1;
- The investigators deem that it is in the best interest of the subject to receive SHR-1210 combined with famitinib, and the subject is not required to start other anti-tumor treatment immediately;
- Eligible for all inclusion criteria (except the requirement for the number of prior treatment lines) and none of the exclusion criteria after completing all examinations and assessments specified within 2 weeks before the first dose;
- The subject is able to tolerate continued study treatment;
- No significant clinical symptoms/signs of tumor progression;
- No rapid progression and no tumor progression involving vital organs/sites (e.g., spinal cord compression);
- Have completed imaging evaluation within 4 weeks before the first dose of SHR-1210 combined with famitinib;
- The subject must re-sign the ICF before continuing treatment.

#### 5.6.2. Other considerations for continuing treatment

The assessment of clinical benefit must consider whether the subject has clinical exacerbations and whether the subject can benefit from continuing treatment. It is recommended that the investigators should discuss with the sponsor whether the subject should continue treatment after PD.

The time window for continuing treatment is within 4 weeks after confirmed PD.

If it is decided that the subject will continue the study treatment after PD, the subject should continue to be treated, evaluated, and followed up according to the protocol requirements.

Subjects who continue treatment after PD must be fully informed and sign the ICF for continuing treatment after PD.

Continuing treatment will be given until confirmed PD and re-signing of the ICF for continuation is not allowed.

# 6. EVALUATIONS

## 6.1. Efficacy Evaluation

### 6.1.1. Efficacy endpoints

The primary efficacy endpoint for this study is objective response rate (ORR) assessed as per RECIST 1.1 (Appendix II).

Objective response rate (ORR): The proportion of subjects with a best overall response (BOR) of complete response (CR) or partial response (PR) as per RECIST 1.1 in all cohorts.

CR or PR must be confirmed at least 4 weeks + 7 days after the initial assessment.

The ORR refers to the result obtained by dividing the number of subjects whose best overall response (BOR) is complete response (CR) or partial response (PR) by the number of subjects. For subjects without documented PD or new anti-tumor treatment, the best overall response will be determined on the basis of all response evaluations.

For subjects who continue SHR-1210 treatment after progression, the best overall response should be based on the efficacy assessment at the time of first documented progression as per RECIST 1.1.

**Secondary efficacy endpoints include:**

- Duration of response (DOR): defined as the period of time from the first documented tumor response (as per RECIST 1.1) to the first documented objective progression (as per RECIST 1.1) or death of any cause, whichever occurs first. Subjects without PD or death will be censored on the date of their last tumor evaluation. Subjects who start new anti-tumor treatment (not including treatment for non-target bone lesions or palliative radiotherapy) without previously reported progression will be censored on the date of their last tumor evaluation prior to the start of new anti-tumor treatment.
- Disease control rate (DCR): the proportion of subjects with a best overall response (BOR) of complete response (CR), partial response (PR), or stable disease (SD) as per RECIST 1.1. CR or PR must be confirmed at least 4 weeks (28 days) after the initial assessment. Best overall response refers to the best response assessed by the investigators, or the best response from the date of first dose to the first documentation of progression as per RECIST 1.1 or to the start of new anti-tumor treatment (whichever comes first). For subjects without documented PD or new anti-tumor treatment, the best overall response will be determined on the basis of all response evaluations. For subjects who continue SHR-1210 treatment after progression, the best overall response should be based on the efficacy assessment at the time of first documented progression as per RECIST 1.1.
- Time to objective response (TTR): defined as the period of time from the date of first dose to the first documented tumor response (as per RECIST 1.1). Subjects without PD or death will be censored on the date of their last tumor evaluation. Subjects who start new anti-tumor treatment (not including palliative radiotherapy for non-target bone lesions) without previously reported progression will be censored on the date of their last tumor evaluation prior to the start of new anti-tumor treatment.
- Progression-free survival (PFS): defined as the period of time from the date of first dose to the date of the first documented tumor progression (as per RECIST 1.1) or death of any cause, whichever occurs first.
- When determining PFS, clinical exacerbations without definite evidence of PD (as per RECIST 1.1) is not considered progression. For subjects who die without any prior reports of progression, the date of death is considered the date of progression. Subjects without PD or death will be censored on the date of their last evaluable tumor assessment. Subjects without tumor evaluation during the study or death will be censored on their date of first dose. Subjects who have no previously reported PD but have started new anti-tumor treatment will be censored on the date of their last evaluable tumor assessment prior to the start of new anti-tumor treatment.

- 12-month overall survival rate: defined as the survival rate at 12 months from the date of first dose. The 12-month overall survival rate will be estimated using the Kaplan-Meier method;
- Overall survival (OS): defined as the time from the start of the first dose until the death of the subject caused by any reasons. For subjects who are still alive at the last follow-up, their OS will be censored at the date of last follow-up. For subjects who are lost to follow-up, their OS will be censored at the last confirmed survival time before loss to follow-up. The censored OS is defined as the time from the first dose to censoring.

### 6.1.2. Criteria for efficacy evaluation

The primary and secondary efficacy endpoints of SHR-1210 combined with famitinib in subject with advanced urinary system tumors and gynecological tumors will be evaluated as per RECIST v1.1 (Appendix II).

Lesions found at baseline will be assessed every 3 cycles (9 weeks) during treatment and the assessments are not affected by treatment interruption or delay. Bone scan will be performed for suspected bone progression or CR confirmation. Tumor evaluation may also be performed if new lesions are suspected. The first documentation of PR/CR in a subject must be confirmed 4 weeks + 7 days later. If there is PD as per RECIST v1.1, an imaging examination is required for confirmation 4-6 weeks later (except those with rapid progression, with significant clinical progression, or receiving famitinib monotherapy); after the first PD, the following table can be referred to for the criteria of PD confirmation (the criteria below are stipulated in reference to the judgment criteria of iCPD in the iRECIST):

| Cause of First PD | Criteria for Confirming PD |
|---|---|
| Progression of Target Lesions | 1. The absolute value of SoD increases by ≥ 5 mm compared with the minimum SoD measured in the first PD and after the first PD<br>2. Progression of non-target lesions<br>3. Appearance of new lesions |
| Progression of Non-Target Lesions | 1. SoD increases by ≥ 20% and the absolute value of SoD increases by ≥ 5 mm compared with the minimum SoD measured during the screening period and the treatment period<br>2. Further progression of non-target lesions<br>3. Appearance of new lesions |
| Appearance of New Lesions | 1. SoD increases by ≥ 20% and the absolute value of SoD increases by ≥ 5 mm compared with the minimum SoD measured during the screening period and the treatment period<br>2. Progression of non-target lesions<br>3. Re-appearance of new lesions<br>4. The absolute value of sum of diameters (short axis for lymph nodes) of new lesions increases by ≥ 5 mm (for measurable new lesions)<br>5. Further progression of new lesions (for non-measurable lesions). |

SoD: the sum of diameters of target lesions

For the record and assessment of survival, the subjects will be followed up by telephone for survival once every 2 months after the end of treatment until death, loss to follow-up, withdrawal of informed consent, or termination of the study by Hengrui.

Assessments of tumor response include all known or suspected lesions.

Radiographic examinations include computed tomography (CT) or magnetic resonance radiographic (MRI) scans of the chest, abdomen, or pelvis. Brain CT or MRI is performed for subjects with known or suspected brain metastasis, while bone scan is for subjects with known or suspected bone metastasis.

If discontinuation is indicated by deterioration of global health status in absence of objective evidence of PD, such event should be reported as symptomatic deterioration.

If a subject has not undergone imaging examinations within 4 weeks prior to the end of the study, the subject should undergo an imaging examination for response evaluation at the end of the study treatment or upon withdrawal from the study. For subjects with non-radiographic progression (intolerability, other conditions), a tumor evaluation should be performed once every 3 months after the end of the study treatment until PD, death, or initiation of other anti-tumor treatments.

## 6.2. Safety Evaluation

### 6.2.1. Pregnancy test

A blood pregnancy test will be performed on female subjects of childbearing potential before the signing of the ICF to enter the screening period and within 72 h prior to the first dose. Additional tests may be performed to rule out pregnancy if indicated. Appropriate contraceptive measures should be taken after a negative result is obtained from the screening pregnancy test. A pregnancy test will be performed again during the study when necessary, such as at the end of study treatment.

Subjects of childbearing potential are required to take 2 medically approved contraceptive measures (such as intrauterine device, oral contraceptive, or condom) during the study treatment period and within 3 months after the end of study treatment. Male subjects with a partner of childbearing potential should take 2 effective contraceptive measures during the study period and within 3 months after the end of study treatment.

During the study, if a female subject becomes pregnant, she must withdraw from the study immediately. The investigators must report to the sponsor within 24 h and fill out the "Pregnancy Report/Follow-up Form for Hengrui's Clinical Studies".

During the study, if the partner of a male subject becomes pregnant, the subject can continue in the study. The investigators must report to the sponsor within 24 h and fill out the "Pregnancy Report/Follow-up Form for Hengrui's Clinical Studies".

### 6.2.2. Adverse event

The assessment of AEs include type, incidence, severity (according to NCI-CTCAE v4.03), start and end date, whether it is an SAE, whether it is an irAE, whether it is an SIE, causality, and outcome.

AEs that occur during the study, including signs and symptoms at screening, will be recorded on the AE page of the CRF.

### 6.2.3. Laboratory safety evaluation

The blood samples for hematology and blood biochemistry tests will be collected according to the Schedule of Activities, and will be analyzed at the local laboratory. Laboratory safety evaluation mainly includes the following:

**[Hematology]** White blood cell count (WBC), absolute neutrophil count (ANC), lymphocyte count (LYM), red blood cell count (RBC), hemoglobin (Hb), and platelet count (PLT);

**[Urinalysis]** Urine protein, urine occult blood, urine red blood cells, and urine white blood cells. During the screening period, if semi-quantitative test shows urine protein of ≥ 2+, a quantitative 24-h urine protein test is required. Subsequently, if semi-quantitative tests from 2 consecutive follow-ups show protein of 2+, a quantitative 24-h urine protein test is required, and if semi-quantitative test shows protein of > 2+, a quantitative 24-h urine protein test should be performed;

**[Routine stool test]** Subjects with positive fecal occult blood must be retested. If fecal occult blood is confirmed after retest, by combing medical history, a gastroscopy should be performed when necessary;

**[Blood biochemistry]** TBIL, direct bilirubin, ALT, AST, AKP, γ-GT, LDH, total protein, albumin, urea/blood urea nitrogen, creatinine, uric acid, fasting blood glucose, triglyceride, cholesterol, potassium, sodium, chlorine, calcium, phosphorus, blood lipase (only during the screening period and in case of subsequent abdominal pain, abdominal distension, and other symptoms of suspected pancreatitis), and blood amylase (only during the screening period and in case of subsequent abdominal pain, abdominal distension, and other symptoms of suspected pancreatitis);

**[Coagulation function]** Including INR, APTT, PT, and FIB;

**[Myocardial zymography]** Including creatine kinase and lactate dehydrogenase;

**[Thyroid function]** FT3, FT4, and TSH;

**[Pituitary adrenal axis test]** Including ACTH, cortisol, and follicle stimulating hormone;

**[HIV test]** HIV antibody test;

**[Hepatitis B and hepatitis C tests]** Subjects with abnormal hepatitis B markers should undergo quantitative tests of HBV DNA and HBsAg. Subjects with positive anti-HCV antibodies must be tested for HCV RNA;

Refer to the Schedule of Activities for details.

### 6.2.4. Vital signs and physical examination

**[Vital signs]** Body temperature, pulse, respiratory rate, and blood pressure;

**[Blood pressure monitoring]** The blood pressure of subjects will be measured by the investigators or study nurse in the screening period. At each blood pressure measurement, smoking and coffee are prohibited within 30 min before measurement, and subjects should rest for at least 10 min. The sitting position will be taken at measurement by placing the elbow at the same level as the heart. Each blood pressure measurement should be taken on the same side of the body. During treatment, blood pressure will be measured by subjects themselves (subjects in Cohort 6 are not required to measure blood pressure at home) and recorded in their diary cards. Blood pressure should be measured at least 3 times per week during the first 2 cycles. For subjects with abnormal blood pressure, blood pressure should be measured daily; if normal, blood pressure should be measured at least twice per week after Cycle 2. The investigators or study nurse will also measure blood pressure at each visit;

**[Physical examination]** General conditions, head and face, skin, lymph nodes, eyes (scleras, pupils), ears, nose, throat, mouth, respiratory system, cardiovascular system, abdomen (including liver and spleen), reproductive and urinary systems, musculoskeletal system, nervous system, and mental state. Note: Comprehensive physical examinations must be performed during the study, but only abnormal findings need to be documented in the eCRF. Repeated documentation is not required if there is no change from baseline.

### 6.2.5. 12-lead ECG

**[12-lead ECG]**: At least QT, QTc, and P-R intervals need to be indicated. ECG examination within 7 days before the first dose in the screening period will be performed for 3 consecutive times at an interval of about 5 min. The average value of the 3 QTc results is taken. The average value of the 3 QTc results of the enrolled subject must meet the inclusion and exclusion criteria. If the ECG is abnormal (with clinical significance) during the study, another two examinations must be performed, and the results of the additional examinations will be recorded in the "Unscheduled Examinations" section of eCRF.

## 6.3. Pharmacokinetic and SHR-1210 ADA Evaluation

PK evaluation:

- Plasma concentrations and PK parameters (if applicable) of famitinib and its main metabolites for combination therapy and monotherapy, including $C_{max}$, $T_{max}$, $AUC_{0\text{-}24\ h}$, CL/F, and V/F.
- Concentrations of anti-PD-1 antibody SHR-1210 during combination therapy.

Others:

- Proportion of subjects with anti-SHR-1210 antibodies (ADAs) during combination therapy.

## 6.4. Biomarker Evaluation

If a subject agrees to participate in the exploratory biomarker part of the study, biosamples (e.g., plasma, serum, as well as archived tumor specimens and those obtained in the study) should be collected for the exploratory analysis of biomarkers to evaluate the correlation among disease activity, effects of investigational products, and clinical outcomes.

The expression level of PD-L1 in tumor tissue will be assessed by immunohistochemistry (for subjects receiving SHR-1210 combination therapy or monotherapy only); the MSI of tumor tissue will be assessed by next-generation sequencing (for endometrial cancer and ovarian cancer only); the FGFR2/3 mutation will be assessed by immunohistochemistry/next-generation sequencing (for urothelial carcinoma treated with combination therapy only);

Subsequent tests and analyses will also be performed on tumor or blood samples and test results obtained during the study. These tests and analyses include re-analysis of existing data, as well as further testing and analysis of DNA, RNA, signal pathway-related proteins, and tumor markers of obtained samples.

The results of the exploratory biomarker study will be reported separately and will not be included in the clinical study report.

The results from the exploratory biomarker study may be combined and analyzed with biomarker data from other studies of the investigational products to formulate a hypothesis which will be further validated in future studies.

# 7. ADVERSE EVENT REPORTING

## 7.1. Adverse Event (AE)

All AEs that occur from the signing of the ICF until at least 30 days after the last study dose should be collected and followed up. All AEs suspected to be related to SHR-1210 should be collected from 30 to 90 days after the last dose. If no new anti-tumor treatment starts during the period, all SAEs should be collected; if new anti-tumor treatment starts, only SAEs related to the investigational products will be collected after the start of the new anti-tumor treatment.

Definition of adverse event

An AE refers to any untoward medical occurrence that may present during treatment with a pharmaceutical product but which does not necessarily have a causal relationship with this treatment. AEs can include any unfavorable and unintended symptoms, signs, laboratory abnormalities, or diseases, including the following:

1) Worsening of pre-existing (prior to entering clinical study) medical conditions/diseases (including worsening symptoms, signs, or laboratory abnormalities);

2) Any new AE: Any new adverse medical conditions (including symptoms, signs, and newly diagnosed diseases);

3) Clinically significant abnormal laboratory findings.

All AEs should be collected in detail by study personnel, including: the name of the AE and description of all relevant symptoms, onset time, severity, causality with the investigational products, duration, measures taken, as well as final results and outcomes.

### 7.1.1. AE severity grading criteria

Refer to NCI-CTCAE v4.03 for grading criteria. Refer to the following criteria for AEs not listed in NCI-CTCAE v4.03:

| Grade | Clinical Description of Severity |
|---|---|
| 1 | Mild; asymptomatic or mild symptoms; clinical or laboratory test abnormality only; intervention not indicated |
| 2 | Moderate; minimal, local, or non-invasive intervention indicated; limiting age-appropriate instrumental activities of daily living (ADL), e.g., preparing meals, shopping for groceries or clothes, using the telephone, managing money, etc. |
| 3 | Severe or medically significant but not immediately life-threatening; hospitalization or prolongation of hospitalization indicated; disabling; limiting self-care ADL. Self-care ADL: refer to bathing, dressing and undressing, feeding self, using the toilet, taking medications, and not bedridden |
| 4 | Life-threatening consequences; urgent intervention indicated |
| 5 | Leading to death |

#### 7.1.2. Causality assessment

AEs include all unexpected clinical manifestations. All the AEs occurring after the signing of the ICF must be reported and recorded, regardless of whether the AEs are related to the investigational products and whether the subject has been administered with the products. All subject complaints and abnormal changes in laboratory tests during the treatment period should be documented truthfully. The severity, duration, measures taken, and outcome of the AE should be noted. The investigators should assess the relationship between the AE and the investigational products, such as whether there is a plausible temporal relationship with the investigational products, the characteristics of the investigational products, the toxicological and pharmacological effects of the investigational products, whether there are concomitant medications, the subject's pre-existing diseases, medical history, family history, as well as challenge and rechallenge. The causality assessment will be provided using the following five categories: "related, possibly related, unlikely related, not related, and unassessable".

### 7.2. Serious Adverse Event (SAE)

#### 7.2.1. Definition of SAE

An SAE refers to a medical occurrence during the study that results in hospitalization, prolonged hospitalization, disability, incapacity, life-threatening or death, or congenital malformation. The following medical events are included:

- Events resulting in death;
- Life-threatening events (defined as when the subject is at immediate risk of death at the time of the event);
- Events resulting in hospitalization or prolonged hospitalization;
- Events resulting in permanent or serious disability/incapacity/impairment of work ability;
- Congenital anomalies or birth defects;
- Other important medical events (defined as events that may jeopardize the subject or require interventions to prevent any of the above).

#### 7.2.2. Hospitalization

AEs resulting in hospitalization (even if for less than 24 h) or prolonged hospitalization during the clinical study should be considered as SAEs.

Hospitalization does not include the following:

- Hospitalization at a rehabilitation institution
- Hospitalization at a sanatorium
- General emergency admission
- Day surgery (e.g., outpatient/same-day/ambulatory surgery)
- Social reasons (medical insurance reimbursement, etc.)

Hospitalization or prolonged hospitalization unrelated to the worsening of an AE is not an SAE. For example:

- Hospitalization due to the pre-existing disease without new AEs and aggravation of the pre-existing disease (e.g., hospitalization to examine laboratory abnormalities that have persisted before the study until now);
- Hospitalization for management reasons (e.g., annual physical examination);
- Hospitalization during the study as specified in the study protocol (e.g., as required by the protocol);
- Elective hospitalization unrelated to worsening of AEs (e.g., elective surgery);
- Scheduled treatment or surgery that should be documented throughout the entire study protocol and/or in the subjects' individual baseline information;
- Hospitalization merely for use of blood products.

Diagnostic or therapeutic invasive (e.g., surgery) and non-invasive procedures should not be reported as AEs. However, when a condition resulting in such procedures meet the definition of AE, it should be reported as such. For example, acute appendicitis during the AE reporting period should be reported as an AE, and the resulting appendicectomy should be recorded as the treatment of the AE.

### 7.2.3. Progressive disease

Progressive disease is defined as the worsening of the subject's conditions caused by the indications of the study, including radiographic progressions and progressions in clinical symptoms and signs. New metastases relative to the primary tumor or progressions of the previous metastases are recognized as PD. Life-threatening events, hospitalization or prolonged hospitalization, or events resulting in permanent or severe disability/incapacity/impairment of work ability, congenital anomalies or birth defects arising from the symptoms and signs of PD are not reported as SAEs. If there is any uncertainty over the causality between the SAE and PD, the event should be reported as an SAE.

In the clinical study, if a subject dies during the safety follow-up period (30 days after the last dose of famitinib or 90 days after the last dose of SHR-1210, whichever is later), it should be reported as an SAE, regardless of whether the investigators evaluate that the death may be related to PD or whether the subject has received other anti-tumor treatments. The term "death" should not be used as a term of the SAE, but should be the outcome of an event. Events that result in death should be recorded as SAEs. The medical condition/disease (including deterioration of symptoms and signs) that causes or leads to death should be recorded in the eCRF as an SAE term, and reported as an SAE. If the cause of death cannot be determined at the time of reporting, the SAE term "death of unknown cause" should be used for documentation.

If the subject dies from PD as assessed by the investigators, the Grade 5 event caused by PD should be recorded in the eCRF and reported as an SAE; if the death caused by PD cannot be attributed to a specific medical event, then the Grade 5 "tumor progression" should be recorded in the eCRF and reported as an SAE, and the investigators should provide evidence at the same time indicating that the death is caused by PD (for example, radiographic changes that suggest tumor development or progression, and clinical deterioration related to the disease process).

### 7.2.4. SAE reporting

In the event of an SAE, whether it is in the initial report or a follow-up report, the investigators must complete the "Serious Adverse Event/Special Interest Event Report Form for Hengrui's Clinical Studies" immediately, with a signature and date, and notify the sponsor within 24 h of knowing of the event. Relevant authorities must be informed of the SAE in a timely manner according to regulatory requirements.

SAEs that occur after the safety follow-up period which are suspected to be related to the investigational products should be collected. Symptoms, severity, causality with the investigational products, time of occurrence, duration of treatment, measures taken, follow-up time and methods, and outcomes of SAEs should be documented. If the investigators believe that an SAE is not related to the investigational products but potentially related to the study conditions (such as the discontinuation of past treatment, or comorbidities during the study), the causality should be explained in the description section of the SAE report form.

The email address for the sponsor to receive the report of SAEs (and SIEs and pregnancy) is hengrui_drug_safety@hrglobe.cn.

## 7.3. Pregnancy

If a female subject becomes pregnant during the clinical study, the subject must discontinue study treatment immediately; if the partner of a male subject becomes pregnant during the clinical study, the subject can continue the study. The investigators should report pregnancy to the sponsor within 24 h of knowing the event by filling out the "Pregnancy Report/Follow-up Form for Hengrui's Clinical Studies".

The investigators should track a pregnancy event until its final outcome (including any premature termination of pregnancy or childbirth), and in the case of childbirth, it should be followed up for 1 month after delivery. The pregnancy outcome should be reported to the sponsor. If pregnancy outcome meets the SAE criteria (such as ectopic pregnancy, spontaneous abortion, intrauterine death, neonatal death, or congenital anomalies), it should be reported according to SAE procedures.

If a subject experiences any SAE during pregnancy, the SAE should be reported according to the SAE reporting procedure.

## 7.4. Special Interest Event

When an SIE specified in the study protocol occurs, the investigators must fill out the "Special Interest Event Report Form for Hengrui's Clinical Studies" and report to the sponsor within 24 h of being notified. If the SIE is an SAE, it should be reported to the relevant institution following the SAE reporting procedures.

In this study, the sponsor will pay special attention to irAEs. If the subject develops an AE of the following type and severity which is determined as possibly related or related to the investigational products, the investigators should promptly inform the sponsor:

- Grade ≥ 3 infusion reaction;
- Other Grade ≥ 3 irAEs;
- Abnormal liver function tests (potential drug-induced liver injury)

Abnormal liver function tests:

For abnormal AST and/or ALT levels with abnormal TBIL elevation, if all of the following conditions (1), (2), and (3) are met without other possible causes, the abnormality should be reported as an SIE; if an SAE criterion is also met, the event should also be reported in accordance with SAE procedures.

| Condition | Criterion |
|---|---|
| (1) Abnormal ALT or AST | Normal at baseline: ALT or AST > 3 × ULN during treatment period;<br>Abnormal at baseline: ALT or AST > 2 × baseline level and value > 3 × ULN during treatment period; or value > 8 × ULN. |
| (2) Abnormal TBIL | Normal at baseline: TBIL > 2 × ULN during treatment period;<br>Abnormal at baseline: TBIL increase > 1 × ULN **or** value > 3 × ULN during treatment period. |
| (3) No hemolysis, and alkaline phosphatase < 2 × ULN (or not available) | |

Abbreviations: ALT = alanine aminotransferase, AST = aspartate aminotransferase, TBIL = total bilirubin, and ULN = upper limit of normal.

If abnormal AST and/or ALT with abnormal TBIL increase are observed during the treatment or follow-up period, the subject must return to the study site promptly after being notified (ideally within 48 h) for examinations and assessments. The examinations and assessments should include hepatic function laboratory tests, detailed interview on medical history, and physical examinations, and should consider the possibility of liver tumors (primary or secondary).

Except for reexaminations of AST and ALT, hepatic function laboratory tests should also include albumin, creatine kinase, TBIL, direct and indirect bilirubin, γ-glutamyltransferase, PT/INR, and alkaline phosphatase. It is recommended to collect the following medical histories: history of alcohol, acetaminophen, soft drugs, various supplements, family diseases, occupational exposure, sexual behavior, travel, contact with patients with jaundice, surgery, blood transfusion, hepatic diseases, or allergies. Further tests may include the testing for acute infection of hepatitis A, B, C, D, and E, and hepatic imaging (such as biliary tract).

## 7.5. Collection and Follow-Up of AEs/SAEs

All AEs/SAEs should be followed up until they are resolved, return to baseline levels or Grade ≤ 1, reach a stable state, or are reasonably explained (e.g., loss to follow-up or death).

During each visit, the investigators should ask about the situation of AEs/SAEs that occur after the last visit and provide follow-up information in a timely manner based on the sponsor's query request. Refer to Table 6 below for the time limits of AE/SAE collection:

**Table 6. Time limits of AE/SAE collection.**

| Time Limit | Collection/Documentation Requirement |
|---|---|
| Up to 30 Days (inclusive) After the Last Dose | Collect all AEs/SAEs |
| 30 to 90 Days After the Last Dose | Collect all AEs related to SHR-1210 |
| | Collect all SAEs if no new anti-tumor treatment starts;<br>If new anti-tumor treatment starts, only SAEs related to the investigational products will be collected after the start of the new anti-tumor treatment. |
| After 90 Days Post the Last Dose | Collect SAEs related to the investigational products |

# 8. MANAGEMENT OF ADVERSE EVENTS

## 8.1. Immune-Related Adverse Event (irAE)

Immune-related adverse events (irAEs) are clinically significant side effects that are consistent with the immunological mechanisms of the investigational products. irAEs require further serological, immunological, and pathological (biopsy) data to support its diagnosis. Also, tumors, infections, metabolism, toxins, or other pathogenic factors must be ruled out.

**Management principles for irAEs (see Appendix IV for details):**

The treatment of immune-related adverse reactions should be based on the medical practice and guidelines of the study site. The treatment recommendations for immune-related adverse reactions are as follows for reference:

Subjects using hormones should pay attention to calcium and vitamin D3 supplement, acid suppression, and protection of gastric mucosa.

- **Immune-related skin reaction**

If skin reactions are suspicious of immune-related instead of infection-related or being caused by other drugs, photosensitivity is often present. The following hematological tests are required: antibody (ANA) test, SS-A/Anti-Ro, and SS-B/Anti-La, anti-smooth muscle antibodies, and antineutrophil cytoplasmic antibodies, etc. In principle, the skin reactions will be treated according to the NCI-CTCAE (v4.03: 14 Jun., 2010) severity grade (Grade 1-4):

Grade 1: Avoid skin irritants and sun exposure (wear UV-proof clothes and glasses), and take oral antipruritics;

Grade 2: Consider starting prednisone 0.5-1 mg/kg (gradually reduce within 4 weeks) (The oral dose of Medrol® is 0.4-0.8 mg/kg/d for one week, then reduced by half for one week, then to 1/4 for one week, and then stopped if appropriate). In addition, give an antihistamine, and topical application of Pevisone® cream and skin lubricant. Assess efficacy every 3 days;

Grade 3: Consider interrupting the investigational products in subjects who fail symptomatic treatment. Give oral antihistamines, topical corticosteroid cream, and skin lubricant. Start methylprednisolone tablets (Medrol®) 1-2 mg/kg/d, and gradually reduce for at least 4 weeks (immunosuppressive agents may be added in the case of exfoliative dermatitis);

Grade 4: All serious toxicities, uncontrolled with previous treatment methods. Stop immunotherapy until systemic dose of prednisone is less than or equal to 10 mg per day (less than or equal to 8 mg of Medrol®). Antihistamines can be used instead of hormone therapy.

Hospital admission: Methylprednisolone IV 1-2 mg/kg/d, add ciclosporin if necessary (under the guidance of rheumatology and immunology department). Gradually reduce when the toxicity is resolved. Monitor toxicities closely. Resume immunotherapy when the toxicity returns to Grade ≤ 1. If the toxicity cannot return to Grade 1, then consider switching the medication.

If symptoms persist, use Mabthera® (375 $mg/m^2$ once per week for a total of 4 weeks, or 500-1000 mg/dose, repeated 2 weeks later) instead of long-term hormone therapy.

Use antibiotics concomitantly if skin infection is suspected due to skin breakdown.

If accompanied by extensive exfoliative dermatitis, provide symptomatic supportive treatment, hydration, and electrolytes.

- **Immune-related pneumonitis**

In the clinical study of SHR-1210, the monitoring of signs and symptoms of immune-related pneumonitis, such as cough and chest discomfort, in the subjects will be strengthened.
A high-dose hormone therapy will be given to subjects with a Grade 2 or greater event confirmed by chest CT. Subjects with Grade 2 immune-related pneumonitis can interrupt SHR-1210 and be treated, but subjects with a Grade 3 or 4 immune-related pneumonitis should permanently discontinue SHR-1210. Consultation with the department of respiration is recommended.

For specific operations, refer to the followings:

Grade 2 event: 1 mg/kg/day of methylprednisolone or equivalent given via intravenous or oral administration. The changes in CT should be closely monitored. After the event recovers to Grade 1, oral administration of 0.5 mg/kg/day of prednisone can be continued for 2 weeks, then the dose of prednisone should be reduced by 5 mg/week until drug discontinuation.

Grade 3 event: 2-4 mg/kg/day of methylprednisolone or equivalent given via intravenous injection. The changes in CT should be closely monitored. After the event recovers to Grade 1, the dose of methylprednisolone should be reduced by 50% every 3 days. Oral administration of 0.5 mg/kg/day of prednisone can be continued for 2 weeks, then the dose of prednisone should be reduced by 5 mg/week until drug discontinuation.

If the hormone therapy does not improve or deteriorate the condition after 3-5 days, a combination therapy with immunosuppressants may be used for the treatment after discussion with the sponsor.

- **Immune-related hepatitis**

In the clinical study of SHR-1210, the monitoring of signs and symptoms of immune-related hepatitis, such as liver discomfort and abnormally increased transaminase, in the subjects will be strengthened. A high-dose hormone therapy will be given to subjects with a Grade 2 or greater event. For specific operations, refer to the followings:

Grade 2 hepatitis: 0.5-1 mg/kg/day of methylprednisolone or equivalent given via intravenous or oral administration. The changes in liver function parameters should be closely monitored. After the event recovers to Grade 1, the dose of hormone should be slowly reduced in a period no less than 1 month.

Grade 3 event: 1-2 mg/kg/day of methylprednisolone or equivalent given via intravenous injection. The changes in liver function parameters should be closely monitored. After the hepatitis recovers to Grade 1, the dose of hormone should be slowly reduced in a period no less than 1 month.

If the hormone therapy does not improve or deteriorate the condition after 3-5 days, a combination therapy with immunosuppressants may be used for the treatment after discussion with the sponsor.

- **Treatment-related diarrhea**

Grade 1: Continue treatment with the original regimen. Avoid spicy and irritating foods, take probiotics orally, use pinaverium bromide or raceanisodamine if necessary to relieve pain, add diosmectite to stop diarrhea;

Grade 2: Continue treatment with the original regimen. In addition to principles as for Grade 1, repeat routine stool test, and if no red or white blood cell detected:

1) Less than 3 bowel movements per day with no abdominal pain, adjust diet, with no special treatment required;

2) More than 3 bowel movements per day with loose stool but not watery, recommend oral Smecta®;

3) Add belladonna tablet to relieve abdominal pain;

4) Under the guidance of the physician, recommend an initial dose of Imodium® 4 mg, followed by 2 mg every 2 h until the diarrhea stops for 12 h (up to 48 h), 4 mg every 4 h at night, along with oral diosmectite and intestinal antibiotics. If red and white blood cells present in routine stool test, then berberine is preferred. If there are a lot of red and white blood cells, the antibiotics and probiotics are recommended.

Grade 3: Treat as for Grade 2 except for reduction of one treatment dose;

Grade 4: Treatment interruption, hospital admission, fasting, gastrointestinal decompression, parenteral nutrition, hydration and electrolytes, continuous administration of acid suppressant and octreotide/somatostatin by intravenous pump.

Continue treatment with the investigational products when diarrhea returns to Grade 1-2. Consider modifying the dose of the investigational products if appropriate.

- **Immune-related enteritis**

In the clinical study of SHR-1210, the monitoring of signs and symptoms of immune-related enteritis, such as abdominal pain, diarrhea, and hematochezia, in the subjects will be strengthened. A high-dose hormone therapy will be given to subjects with a Grade 2 or greater event. Subjects with Grade 2 or 3 immune-related enteritis can interrupt SHR-1210 and be treated, but subjects with Grade 4 immune-related enteritis should permanently discontinue SHR-1210.

- **Immune-related thyroid dysfunction**

Thyroid dysfunction can occur at any time during the study. Therefore, in the clinical study of SHR-1210, the thyroid functions of the subjects will be regularly examined to closely monitor the clinical symptoms of thyroid dysfunction. After the occurrence of immune-related hyperthyroidism, the subject should be given a high dose of cortisone/prednisone. Hormone replacement therapy is used for the treatment of hypothyroidism, but glucocorticoids are not applicable.

In the clinical study of SHR-1210, the monitoring of signs and symptoms of immune-related thyroid dysfunction in the subjects will be strengthened. A high-dose hormone therapy will be given to subjects with a Grade 3 or greater event, but subjects with a Grade 4 event should permanently discontinue SHR-1210.

- **Immune-related nephritis and renal failure**

In the clinical study of SHR-1210, the monitoring of signs and symptoms of immune-related nephritis in the subjects will be strengthened. A high-dose hormone therapy will be given to subjects with a Grade 2 or greater event. Subjects with a Grade 2 event can interrupt SHR-1210 and be treated, but subjects with a Grade 3 or 4 event should permanently discontinue SHR-1210.

- **Immune-related hypophysitis**

In the clinical study of SHR-1210, the monitoring of signs and symptoms of immune-related hypophysitis in the subjects will be strengthened. A high-dose hormone therapy will be given to subjects with a Grade 2 or greater event. Subjects with a Grade 2 or 3 event can interrupt SHR-1210 and be treated, but subjects with a Grade 4 event should permanently discontinue SHR-1210.

- **Other immune-related adverse reactions**

In principle, interruption of SHR-1210 is preferred based on the severity of the adverse reaction. The study treatment can be considered to resume when AE returns to Grade ≤ 1. The study treatment should be permanently discontinued if severe (Grade 3) or life-threatening (Grade 4) adverse reactions occur.

## 8.2. Infusion Reaction

As a fully humanized monoclonal antibody, SHR-1210 poses relatively lower risks of infusion reactions and premedication is therefore not required. Once an infusion reaction occurs, the infusion should be slowed or interrupted accordingly, and supportive treatment should be given. Also, premedication should be given before further administrations. For acute infusion reactions (including cytokine release syndrome, angioedema, anaphylactic shock, and allergic reactions, please refer to terms and criteria of NCI-CTCAE v4.03), their relevant symptoms and signs usually occur during drug infusion or shortly after infusion, and usually disappear within 24 h after the infusion is completed. The signs and symptoms include: allergic reaction/hypersensitivity reaction (including drug-induced fever), cough, chills, shiver, dizziness, headache, fatigue (weakness, somnolence), rash/peeling skin, pruritus/itching, arthralgia, myalgia, hypotension/hypertension, nausea, vomiting, diaphoresis, tachycardia, cancer pain, urticaria (rubella), dyspnea (shortness of breath), or bronchospasms. Any Grade 3 or 4 infusion reactions must be reported to the sponsor within 24 h, and should be reported as SAEs if the criteria for SAE are met.

Management of allergic reactions should be based on the medical practice and guidelines of the study site. The treatment recommendations for infusion reactions are shown below for reference.

**Table 7. Recommendations for treatment of infusion reactions.**

| CTCAE Grade | Clinical Symptom | Clinical Management | SHR-1210 Treatment |
|---|---|---|---|
| Grade 1 (mild) | Mild and transient reactions; | Bedside observation and close monitoring until recovery. (Premedication is recommended for subsequent infusion: diphenhydramine 50 mg or equivalent, and/or acetaminophen 325-1000 mg, at least 30 min prior to SHR-1210 administration) | Continue |
| Grade 2 (moderate) | Moderate reactions requiring treatment or interruption; rapidly resolve after symptomatic treatment (such as antihistamines, nonsteroidal anti-inflammatory drugs, anesthetics, bronchodilators, and intravenous fluids) | Intravenous infusion of normal saline, IV of diphenhydramine 50 mg or equivalent and/or acetaminophen 325-1000 mg;<br>Bedside observation and close monitoring until recovery.<br>Corticosteroids or bronchodilators can be considered based on clinical needs;<br>The amount of investigational products infused should be recorded in the original medical record;<br>Premedication is recommended for subsequent infusion: diphenhydramine 50 mg or equivalent, and/or acetaminophen 325-1000 mg, at least 30 min prior to SHR-1210 administration. Use corticosteroids (equivalent to 25 mg of hydrocortisone) when necessary. | Interrupt. Re-administer at 50% of the initial rate after symptoms resolve. If no reaction occurs within 30 min, restore the original infusion rate (100%). Closely monitor. If the symptoms recur, the administration of the current SHR-1210 dose will be discontinued. |
| Grade ≥ 3 (severe) | Grade 3: Severe reactions with no rapid resolution after intervention and/or treatment interruption; symptom recurrence after resolution; sequelae requiring hospitalization.<br>Grade 4: life-threatening | Immediately discontinue SHR-1210;<br>Administer normal saline by intravenous infusion.<br>• Bronchodilators are recommended: 0.2-1 mg of subcutaneous 1:1000 adrenaline injection or slow intravenous injection of 1:10,000 adrenaline solution 0.1-0.25 mg, and/or intravenous diphenhydramine 50 mg plus methylprednisolone 100 mg or equivalent if necessary;<br>• Based on the guidelines for anaphylaxis of the study sites;<br>Bedside observation and close monitoring until recovery. | Permanently discontinue. |

## 8.3. Symptomatic Treatment for Famitinib-Related Adverse Reactions

### 1) Hand-and-foot syndrome (HFS)

Hand-and-foot syndrome is skin toxicity with palmar-plantar dysesthesia or acral erythema and manifests especially in areas under pressure or force. It may occur in patients with tumor during chemotherapy or molecular targeted therapy. HFSR is characterized by numbness, dysesthesia, paresthesia, tingling, no pain or ache, skin swelling, or erythema, desquamation, chapping, scleroma-like blisters, and severe pain.

HFS grading:

Grade 1: numbness/dysesthesia/paresthesia, painless swelling or erythema of the hands and/or feet and/or discomforts that do not affect normal activities.

Grade 2: painful erythema and swelling of the hands and/or feet and/or discomforts affecting patients' activities of daily living.

Grade 3: wet desquamation, ulcers, blisters, or severe pain of the hands and/or feet and/or severe discomforts that cause the patients to be unable to work or

perform activities of daily living. Intense pain and loss of skin function, relatively rare.

Symptomatic treatment and management of HFS:

Some necessary symptomatic and supportive treatments must be taken, including: strengthen skin care, keep skin clean, and avoid secondary infections; avoid pressure or friction; use moisturizers or lubricants, topically use lotions or lubricants containing urea and corticosteroids; topically use antifungal or antibiotic treatment if necessary.

Note: If Grade 3 or greater HFS occurs for more than 3 times with an aggravating trend, the subject should discontinue the study treatment and withdraw from the study.

**2) Hypertension**

Patients should be strictly screened according to blood pressure requirements in the inclusion and exclusion criteria prior to enrollment. Patients with hypertension can control the blood pressure by adjusting the dose of or adding new antihypertensive drugs before administering the investigational products. The blood pressure must be under 140/90 mmHg (average of 2 blood pressure measurements taken at least 24 h apart) before the first dose.

Monitoring and handling of such hypertension: Blood pressure should be monitored at least 3 times a week during the first 2 cycles of the study treatment.

Since anti-VEGF/VEGFR targeted drugs can decrease the synthesis of NO and ultimately activate the renin-angiotensin-aldosterone system to cause hypertension, angiotensin converting enzyme (ACE) inhibitors (such as captopril, enalapril, benazepril, and cilazapril) are preferred for antihypertensive therapy. For some patients who are allergic or intolerant to ACE inhibitors, angiotensin II receptor blockers (ARB, such as losartan, valsartan, irbesartan, and telmisartan) can be used for treatment. In addition to lowering the blood pressure, ARB is also beneficial for alleviating proteinuria. ACE inhibitors can be used in patients with chronic kidney diseases, proteinuria, and metabolic syndrome; dihydropyridine calcium ion antagonists are suitable for elderly patients.

When subjects develop hypertension or aggravated hypertension during drug administration, the following measures should be used: 1) adjust the investigational products according to the protocol (see table below); 2) start the administration of antihypertensive drugs or adjust the dose of antihypertensive drugs.

Diuretic antihypertensive drugs are not recommended. Antihypertensive drugs with an inhibitory effect on CYP3A4, such as nicardipine, diltiazem, and verapamil, are prohibited during the administration period of the investigational products. For those with hypertensive crisis, the application of famitinib should be terminated.

### 3) Hemorrhage

Symptomatic treatment should be actively given for hemorrhage of digestive tract, including fecal occult blood (++) or above, hematemesis, or bloody stool. Patients with upper gastrointestinal hemorrhage should be put under fasting and given acid suppression, gastric mucosal protection, hemostasis (transamin, reptilase, etc.), as well as octreotide if necessary; patients with lower gastrointestinal hemorrhage should be given hemostasis, blood transfusion and supportive care, etc.; for those whose bleeding cannot be controlled, assistance from the surgery department should be requested immediately.

Patients with hemoptysis should be given hemostasis, blood transfusion and supportive care, etc.; for those whose bleeding cannot be controlled, assistance from the surgery department should be requested.

Note: Patients with cerebral hemorrhage, Grade 2 or greater pulmonary hemorrhage, and Grade 3 or greater hemorrhage should stop the study treatment immediately, undergo symptomatic treatment, discontinue famitinib, and interrupt SHR-1210. Consider whether to resume SHR-1210 as appropriate after symptoms resolve or disappear.

### 4) Proteinuria

All subjects should be closely monitored for proteinuria throughout the entire treatment period, especially for those with a history of hypertension. For those with a urine protein result of 2+ in 2 consecutive tests, a 24-h urine protein assay is required. For those with urine protein > 2+, a 24-h urine protein assay is required.

Note: In the case of nephrotic syndrome, the subject should discontinue the treatment permanently and withdraw from this clinical study.

**5) Thrombosis**

If any arterial thrombosis (such as cerebral ischemia, stroke, angina pectoris, and myocardial infarction) occurs, the subject should discontinue the treatment immediately and withdraw from the study. In the case of any symptomatic IV venous thrombosis, the subject should discontinue the treatment and withdraw from the study.

Once the symptoms of thrombosis are observed, symptomatic treatment, surgery, or anticoagulants should be immediately given.

**6) Fatigue and weakness**

Fatigue and weakness are common tumor-related clinical symptoms, the cause of which might be electrolyte disturbance, abnormal liver function, abnormal cardiac function, etc. Also, fatigue and weakness are common clinical adverse reactions of targeted anti-angiogenic drugs, such as sunitinib, pazopanib, and sorafenib. Clinical reports show that targeted anti-angiogenic drugs may increase the incidence of fatigue and weakness through hypothyroidism.

In previously completed clinical trial of famitinib, subjects in the treatment group showed a higher incidence of fatigue and weakness than those in the control group, and the mechanism behind the increased incidence of fatigue and weakness caused by famitinib is yet unidentified.

Therefore, close attention should be paid when a patient shows and reports Grade 2 or greater fatigue and weakness. In the case of Grade 3 or greater fatigue and weakness, the patient should be admitted to the hospital immediately for detailed examinations to exclude possible reasons such as electrolyte disturbance, abnormal liver function, cardiac dysfunction (ECG, echocardiography), and abnormal hormone levels (adrenal hormones, thyroid hormones). Symptomatic treatment should be given and the dose should be interrupted or modified according to the principle of dose modification.

**7) Abdominal pain**

Abdominal pain is not uncommon in the treatment with famitinib and is often a concomitant symptom of tumor. Also, gastrointestinal perforation occasionally occurs in clinical trials of famitinib and other anti-angiogenic drugs. For subjects with abdominal pain, the investigators should be cautious of potential gastrointestinal perforation. Upon the observation of gastrointestinal perforation, the drugs should be discontinued immediately, and the subject should withdraw from the study and be given active symptomatic treatment.

# 9. CLINICAL MONITORING

The CRA must follow the GCP and SOP, make visits to the study site for clinical monitoring on a regular basis or according to the actual conditions, supervise the implementation and progress of the clinical study, check and confirm that all data recorded and entered into CRF are correct and intact and are consistent with source data, and ensure that the clinical study is implemented following the study protocol. The investigators should cooperate with the CRA actively. Specifically, the CRA is responsible for:

1) Confirming that the study site is qualified prior to starting the study, including personnel and training, a well-equipped and functional laboratory with various study-related test conditions, sufficient number of subjects, and study personnel's familiarity with the protocol requirements;

2) Monitoring how the investigators are implementing the study protocol during the course of the study, confirming that ICFs are obtained from all subjects before the study, the enrollment rate and progress of the study, as well as the eligibility of enrolled subjects;

3) Confirming the accuracy and integrity of documentations and reports, and ensuring accurate data entry of all case report forms and consistency with source data. All errors or omissions have been corrected or noted, signed and dated by the investigators. Dose modifications, treatment changes, concomitant medications, intercurrent diseases, loss to follow-up, and missing investigations should be confirmed and documented for each subject. Verifying that withdrawal and loss to follow-up of enrolled subjects are explained in the case report forms;

4) Confirming that all AEs have been recorded, and that SAEs have been recorded and reported within the specified time frame. Verifying that the investigational products are supplied, stored, dispensed, and returned in accordance with relevant regulations, and corresponding documentation should be made;

5) Recording visits, tests, and examinations that the investigators have failed to perform, and whether errors or omissions have been corrected;

6) Completing a written monitoring report, which should state the date and time of the monitoring visit, the name of the CRA, and the findings of the visit.

# 10. DATA ANALYSIS/STATISTICAL METHODS

## 10.1. Statistical Hypothesis and Discriminatory Rules

This study adopts Lin & Shih's two-stage adaptive design[11] for Cohorts 1-5.

The discriminatory rules for Cohorts 1-5 are as follows: $n_1$ is the sample size of Stage I, m is the total sample size of the two stages corresponding to the low ORR, n is the total sample size of the two stages corresponding to the high ORR, $s_1$ is the critical value of CR or PR (not inclusive) that needs to be observed in Stage I corresponding to the low ORR (not inclusive), $r_1$ is the (not inclusive) of CR or PR (not inclusive) that needs to be observed in Stage I corresponding to the high ORR (not inclusive), s is the critical value of CR or PR (not inclusive) that needs to be observed in the two stages corresponding to the low ORR (not inclusive), and r is the critical value of CR or PR (not inclusive) that needs to be observed in the two stages corresponding to the high ORR (not inclusive). If, in Stage I, less than or equal to $s_1$ subjects among the $n_1$ subjects achieve CR or PR, the cohort will be terminated. If, in Stage I, more than $s_1$ but less than or equal to $r_1$ subjects among the $n_1$ subjects achieve CR or PR, then proceed to Stage II and additionally enroll m - $n_1$ subjects. If, in Stage I, more than $r_1$ subjects among the $n_1$ subjects achieve CR or PR, then proceed to Stage II and additionally enroll n - $n_1$ subjects. Refer to Tables 8 and 9 for details.

For Cohorts 1-5, when enrollment of Stage I is completed and the minimum number of subjects achieving CR and PR (confirmed or unconfirmed responses ≥ 3) is observed in a cohort, the Stage II enrollment of that cohort can be started. If the minimum number of subjects achieving CR or PR (confirmed or unconfirmed responses ≥ 3) has been observed before the enrollment of Stage I is completed, the enrollment of Stage II can be started immediately after the completion of the Stage I enrollment without being interrupted. If the minimum number of subjects achieving CR or PR (confirmed or unconfirmed responses ≤ 2) has not observed after the enrollment of Stage I is completed, enrollment must be interrupted until the conditions for continuing the enrollment are met. After completion of Stage II, if more than or equal to 12 subjects achieve CR or PR among a total of 53 subjects in the two stages corresponding to the low ORR, or if more than or equal to 8 subjects achieve CR or PR among a total of 33 subjects in the two stages corresponding to the high ORR, further clinical study is recommended.

Simon's two-stage minimax design will be adopted for each tumor type of Cohorts 6, 7, and 8 in this study.

The discriminatory rules for each tumor type of Cohorts 6, 7, and 8 are as follows: $n_1$ is the sample size of Stage I, n is the total sample size of the two stages, $r_1$ is the critical value of CR or PR that needs to be observed in Stage I (not inclusive), and r is the critical value of CR or PR that needs to be observed in the two stages (not inclusive). If, in Stage I, less than or equal to $r_1$ subjects among the $n_1$ subjects achieve CR or PR, the cohort will be terminated; otherwise $n - n_1$ subjects will be additionally enrolled in Stage II. If the number of subjects enrolled in Stage II does not equal to the planned number of enrolled subjects, the new critical value can be calculated using the Koyama and Chen's method[12], if necessary.

For each tumor type of Cohorts 6, 7, and 8, when enrollment of Stage I is completed and the minimum number of subjects achieving CR or PR (Cohorts 6 and 7: confirmed or unconfirmed responses ≥ 2; Cohort 8: confirmed or unconfirmed responses ≥ 7) is observed in a cohort, the Stage II enrollment of that cohort can be started. If the minimum number of subjects achieving CR or PR (Cohorts 6 and 7: confirmed or unconfirmed responses ≥ 2; Cohort 8: confirmed or unconfirmed responses ≥ 7) has been observed before the enrollment of Stage I is completed, the enrollment of Stage II can be started immediately after the completion of the Stage I enrollment without being interrupted. If the minimum number of subjects achieving CR or PR has not observed (Cohorts 6 and 7: confirmed or unconfirmed responses ≤ 1; Cohort 8: confirmed or unconfirmed responses ≤ 6) after the enrollment of Stage I is completed, enrollment must be interrupted until the conditions for continuing the enrollment are met. After completion of Stage II, if, for Cohort 6 or 7, more than or equal to 5 subjects achieve CR or PR among a total of 23 subjects in the two stages, and if, for Cohort 8, more than or equal to 21 subjects achieve CR or PR among a total of 47 subjects in the two stages, further clinical study is recommended.

## 10.2. Sample Size

The sample size for Cohorts 1-5 in this study is estimated based on Lin and Shih's literature and corresponding programs (applications/SAS).

In this study, $p_0$ is set at 15%, low ORR $p_1$ is set at 25%, and high ORR $p_2$ is set at 35%. The power set based on the high ORR $p_2$ is 80%, the power set based on the low ORR $p_1$ is 70%, and two-sided type I error α is set at 0.1. Each cohort requires the following numbers of subjects as shown in Tables 8 and 9.

In each cohort, 22 subjects will be enrolled in Stage I, and 11 or 31 subjects will be enrolled in Stage II depending on the results from Stage I (see Table 9). The maximum number of subjects for each cohort is 33 or 53.

If at least 7 of the 22 subjects in Stage I achieve objective response, 11 subjects will be enrolled in Stage II as estimated based on the sample size of "high ORR at the end of Stage I".
Thus a total of 33 subjects will be enrolled. If 8 or more subjects achieve objective response, the study will proceed to the next period.

However, if only 3, 4, 5, or 6 of the 22 subjects in Stage I achieve objective response, it shows that the investigational products may be effective for that cohort, and 31 subjects will be enrolled in Stage II as estimated based on the sample size of "low ORR at the end of Stage I". Thus a total of 53 subjects will be enrolled. If 12 or more subjects achieve objective response, the study will proceed to the next period.

If less than 3 subjects in a cohort achieve objective response in Stage I, this cohort will be terminated.

**Table 8. Sample size by cohort - Stage I/II.**

| **Type of Tumor** | **Sample Size Based on Period I** | |
|---|---|---|
| | **Low ORR (m)** | **High ORR (n)** |
| Advanced Renal Cell Carcinoma | 53 | 33 |
| Urothelial Carcinoma | 53 | 33 |
| Recurrent Ovarian Cancer | 53 | 33 |
| Endometrial Cancer | 53 | 33 |
| Cervical Cancer | 53 | 33 |

**Table 9. Sample size by cohort and in Stage I/II.**

| | **Stage (two-stage design)** | | **Total Number of Subjects in Each Cohort** | **Two-Sided Alpha Level/Power** |
|---|---|---|---|---|
| | Stage I $(s_1 < \& \le r_1)$ | Stage II[b] $(> r_1)$ | (m\|n) | |
| All Cohorts | | | | |
| Low ORR at End of Stage I | | | | |
| Number of Subjects | 22 | 53 | 53 | 10%/70% |
| Number of Subjects Achieving Response[a] | $\ge 3 \& \le 6$ | $\ge 12$ | | |
| High ORR at End of Stage I | | | | |
| Number of Subjects | 22 | 33 | 33 | 10%/80% |
| Number of Subjects Achieving Response[a] | $\ge 7$ | $\ge 8$ | | |

The sample size is estimated using the method in Lin and Shih's literature (Biometrics. 2004; 60: 482-490) and corresponding programs

a. Number of subjects who achieve response required for Stage II or to achieve a positive result at the end of study.

b. Showing the maximum number of subjects required for each cohort and the maximum number of subjects who achieve objective response at the end of Stage II in order to declare that the treatment is effective.

The Simon's (minimax) two-stage design is adopted to calculate the sample size for each tumor type of Cohorts 6 and 7 based on one-sided alpha = 0.1 and power = 0.7:

**Table 10. Cohorts 6-7: Calculation of sample size by Simon's (minimax) two-stage method.**

| Cohort and Tumor Type | $P_0$ | $P_1$ | Stage I ($r_1/n_1$) | Stage II (r/n) |
|---|---|---|---|---|
| **Cohort 6** Urothelial Carcinoma - SHR-1210 Monotherapy Group | 0.10 | 0.25 | 1/14 | 4/23 |
| **Cohort 7** Urothelial Carcinoma - Famitinib Monotherapy Group | 0.10 | 0.25 | 1/14 | 4/23 |
| **Cohort 7** Cervical Cancer - Famitinib Monotherapy Group | 0.10 | 0.25 | 1/14 | 4/23 |

Note: $P_0$ is the maximum futility boundary, and $P_1$ is the minimum efficacy boundary. $n_1$ is the sample size of Stage I, n is the total sample size of the two stages, $r_1$ is the critical value of CR or PR that needs to be observed in Stage I (not inclusive), and r is the critical value of CR or PR that needs to be observed in the two stages (not inclusive). If, in Stage I, equal to $r_1$ subjects among the $n_1$ subjects achieve CR or PR, the cohort will be terminated; otherwise n - $n_1$ subjects will be additionally enrolled in Stage II.

The Simon's (minimax) two-stage design is adopted to calculate the sample size of Cohort 8 based on one-sided alpha = 0.025 and power = 0.8:

**Table 11. Cohorts 8: Calculation of sample size by Simon's (minimax) two-stage method.**

| Cohort and Tumor Type | $P_0$ | $P_1$ | Stage I ($r_1/n_1$) | Stage II (r/n) |
|---|---|---|---|---|
| **Cohort 8** Renal Cell Carcinoma | 0.30 | 0.50 | 6/21 | 20/47 |

Note: $P_0$ is the maximum futility boundary, and $P_1$ is the minimum efficacy boundary. $n_1$ is the sample size of Stage I, n is the total sample size of the two stages, $r_1$ is the critical value of CR or PR that needs to be observed in Stage I (not inclusive), and r is the critical value of CR or PR that needs to be observed in the two stages (not inclusive). If, in Stage I, equal to $r_1$ subjects among the $n_1$ subjects achieve CR or PR, the cohort will be terminated; otherwise n - $n_1$ subjects will be additionally enrolled in Stage II.

If the ORR of a given cohort in Stage II has been confirmed, the project team will decide whether to enter the expansion study based on the stopping rules specified by the protocol.

## 10.3. Statistical Analysis Plan

The primary objective of this study is to explore the efficacy, safety, tolerability, PK, ADA, biomarkers, etc. of SHR-1210 combined with famitinib in subjects with advanced renal cell carcinoma, urothelial carcinoma, advanced cervical cancer, recurrent ovarian cancer, and endometrial cancer. The study is also aimed at exploring the efficacy, safety, etc. of SHR-1210 monotherapy in subjects with urothelial carcinoma and famitinib monotherapy in subjects with urothelial carcinoma and cervical cancer.

Detailed summaries and methods of statistical analyses for the data collected from the study will be included in the statistical analysis plan (SAP), which will be finalized and filed by the sponsor. The SAP should be revised accordingly if there are any changes to the study protocol that may have a major impact on the SAP, as determined by the sponsor or the principal investigators. Relevant content in the SAP that is relevant to this protocol may be revised. However, if revised content involves the main and/or key factors of the protocol (such as the definition of endpoints or their analysis), such content in the protocol will be revised.

## 10.4. Analysis Population

This study will involve the following analysis sets:

- Full analysis set (FAS): All enrolled subjects who have received at least one dose of the investigational products. This analysis set will be used for efficacy analysis, with FAS1 to FAS8 (FAS7 includes FAS7-1, FAS7-2, and FAS7) corresponding to various cohorts (Cohorts 1-8).
- Evaluable set (ES): All enrolled subjects who have received at least one dose of the investigational products and have undergone at least one valid post-baseline imaging assessment. This analysis set will be used for the analysis of the primary efficacy endpoint, with ES1 to ES8 (ES7 includes ES7-1, ES7-2, and ES7) corresponding to various cohorts (Cohorts 1-8).
- Safety set (SS): All enrolled subjects who have received at least one dose of the investigational products and have post-administration safety data. This analysis set will be used for safety analysis, with SS1 to SS8 corresponding to various cohorts.
- PK analysis set (PKS): All subjects who have received the investigational products and have evaluable post-administration PK data.

## 10.5. Statistical Methods

The following sections include the description of the planned statistical methods.

With regard to the efficacy analysis of this study, Cohorts 1-5 are analyzed and summarized on the combination therapy by tumor type, Cohort 6 is analyzed and summarized on SHR-1210 monotherapy for urothelial carcinoma, Cohort 7 is analyzed and summarized on famitinib monotherapy for urothelial carcinoma, famitinib monotherapy for cervical cancer, and the both as a whole, and Cohort 8 is analyzed and summarized on SHR-1210 combined with famitinib for renal cell carcinoma, respectively.

With regard to the safety analysis of this study, Cohorts 1-5 are analyzed and summarized on the combination therapy by tumor type and as a whole, Cohort 6 is analyzed and summarized on SHR-1210 monotherapy for urothelial carcinoma, Cohort 7 is analyzed and summarized on famitinib monotherapy for urothelial carcinoma, famitinib monotherapy for cervical cancer, and the both as a whole, and Cohort 8 is analyzed and summarized on SHR-1210 combined with famitinib for renal cell carcinoma, respectively.

### 10.5.1. Basic methods

In this study, unless otherwise stated, all data will be summarized using the following general principles/rules.

Basic principles of data analysis:

The continuous variables will be summarized using mean, standard deviation, median, maximum, and minimum; the categorical variables will be summarized using frequency and percentage; for time to event variables , the survival rate and median survival time will be estimated using the Kaplan-Meier method and the survival curves will be plotted; blood concentration data and PK parameters will be summarized using geometric mean, geometric standard deviation, geometric coefficient of variation, mean, standard deviation, coefficient of variation, median, maximum, and minimum. All statistical analyses will be conducted using SAS 9.4 (or later version).

### 10.5.2. Analysis of primary efficacy endpoint

- Objective response rate (ORR)

The primary efficacy endpoint is investigator-assessed objective response rate (ORR) of each cohort or tumor type treated with monotherapy as per RECIST v1.1. The evaluation data will be based on the following indicators: complete response (CR), partial response (PR), stable disease (SD), progressive disease (PD), and not evaluable (NE). All efficacy analyses will be conducted based on the FAS. The number of subjects with objective response, ORR, and the corresponding Clopper-Pearson 95% confidence interval will be provided for each cohort or tumor type treated with monotherapy. The best overall response will be summarized.

### 10.5.3. Analysis of secondary efficacy endpoints

- Duration of response (DOR)

DOR will be analyzed based on subjects with confirmed CR or PR. DOR is defined as the time from the date of first response to PD or death.

The median DOR (mDOR) and the corresponding 95% confidence interval will be estimated using the Kaplan-Meier (KM) method. The survival curve will be plotted.

- Disease control rate (DCR)

The number of subjects with disease control, DCR, and the corresponding Clopper-Pearson 95% confidence interval will be provided for each cohort or tumor type treated with monotherapy. Disease control rate (DCR) will be analyzed with method similar to that of ORR.

$$\mathrm{DCR}（\%） = \frac{\text{subjects with CR/PR/SD}}{\text{all subjects in FAS}} * 100\%$$

- Time to response (TTR)

TTR is defined as the time from the first dose of the investigational products to the first response. The median and quartile of TTR will be estimated.

- Progression-free survival (PFS)

PFS is defined as the time from the first dose of the investigational products to PD (radiographic), or the time from the first dose to death if the subject dies due to any cause before PD. For subjects without PD or death, the date of the last valid imaging evaluation will be taken as the date of censoring. For PFS analysis, the median PFS (mPFS) will be estimated using the Kaplan-Meier (KM) method and the corresponding 95% confidence interval will be estimated using the Brookmeyer Crowley method.

- Overall survival (OS)

OS is defined as the time from the first dose of the investigational products to death. The median OS (mOS) will be estimated using the Kaplan-Meier (KM) method and the 95% confidence interval will be estimated using the Brookmeyer Crowley method.

- 12-month overall survival rate (12-month OS%)

The 12-month survival rate will be estimated using the KM method and the corresponding 95% confidence interval will also be calculated.

### 10.5.4. Handling of missing data

Apart from exceptional circumstances, the following imputing rules apply to the missing dates of safety data events.

If the date of an event is completely missing, it is not imputed. If the day is missing but the year and month of the event onset are the same as those of study treatment, then the missing day is imputed with the day of first dose of study treatment, otherwise it is imputed with the first day of that month; if the day of the end date is missing but the year and month of the event onset are the same as those of end date of study treatment, then it is imputed with the day when the study treatment is ended, otherwise it is imputed with the last day of that month.

If both month and day of an event onset date are missing but its year is the same as that of study treatment, then they are imputed with the month and day of starting study treatment, otherwise, they are imputed with 1 Jan.

All imputed dates must be before the date of withdrawal of ICF, loss to follow-up, and death.

Missing data of laboratory tests, ECG, and vital signs are not imputed.

Refer to the statistical analysis plan for the detailed rules of imputation of other missing data.

### 10.5.5. Safety analysis

All adverse events (AEs) will be coded according to MedDRA and graded using NCI-CTCAE v4.03. AE analysis will mainly focus on treatment-emergent adverse events (TEAEs), i.e., AEs that occur on the day of the first study dose (SHR-1210 combined with famitinib or monotherapy) or thereafter. Non-treatment-emergent AEs (observed prior to first study dose) will only be listed.

Incidence, type, and severity of AEs in each cohort will be summarized by system organ class (SOC) and/or preferred term (PT) according to MedDRA.

AEs leading to treatment interruption, AEs leading to treatment discontinuation, SAEs, CTCAE Grade ≥ 3 AEs, and treatment-related AEs and SAEs in each cohort will be summarized by system organ class (SOC) and preferred term (PT). AEs leading to death will be summarized and listed.

Laboratory tests, hematology, blood biochemistry, and other indicators will be summarized by shift table to describe normal changes, abnormal changes without clinical significance, and abnormal changes with clinical significance after treatment, and will be classified using the most severe grades after treatment. Where applicable, laboratory measurements will be summarized by cross-tabulation according to CTCAE grade (baseline level and highest severity post-baseline).

Vital signs (blood pressure, temperature, pulse, and respiratory rate), ECG (heart rate, PR interval, QRS interval, QT interval, and QTc interval), and other indicators will be summarized by mean, median, and range (minimum and maximum values) by visit. Changes from baseline in ECG indicators will be summarized descriptively, and cross-tabulated for changes to normal and abnormal after treatment. Concomitant medications will be summarized by frequency and percentages.

### 10.5.6. Pharmacokinetic analysis

The PK parameters of famitinib in humans will be calculated using the non-compartmental model (if applicable) and the plasma concentrations will be summarized. The serum concentrations and PK parameters of SHR-1210 in humans will be summarized (if applicable).

From the blood concentration-time data, the PK parameters ($C_{max}$, $T_{max}$, $AUC_{0-t}$, $t_{1/2\beta}$, CLs, Vd, and MRT) will be descriptively summarized (by n, mean, standard deviation, median, minimum, maximum, geometric mean and standard deviation, and %CV) and tabulated based on different investigational products and scheduled blood sampling time points. Mean and/or median blood concentration-time curves (linear and semi-logarithmic) of different investigational products will be plotted based on scheduled blood sampling time points. Blood concentration-time curves of individual subjects and/or overlays of individual subjects in each group (linear and semi-logarithmic) will be plotted.

### 10.5.7. Immunogenicity analysis

The positive rates of ADA will be calculated. The antibody levels of positive subjects will be listed.

### 10.5.8. Multiple comparison/multiplicity

Since this is an early phase II trial and the cohorts are independent of each other, there is no issue of multiplicity adjustment. Thus, the issue of multiplicity adjustment in this section is "not applicable".

### 10.5.9. Exploratory analysis

The proportion of PD-L1-positive cells in tumor tissue and circulating tumor cells (for subjects receiving SHR-1210 combination therapy or monotherapy only), proportion of subjects with dMMR or MSI-H (for endometrial cancer and ovarian cancer only), and proportion of abnormal FGFR2/3 (for urothelial carcinoma treated with combination therapy only) will be evaluated to explore the relationship of PD-L1 expression and/or other biomarkers with efficacy (such as ORR/PFS).

The results of the exploratory biomarker study will be reported separately and will not be included in the clinical study report.

The results from the exploratory biomarker study may be combined and analyzed with biomarker data from other studies of the investigational products to formulate a hypothesis which will be further validated in future studies.

## 10.6. Interim Analysis

According to the study design, multiple interim analyses will be conducted by the study team during the study, based on which the following will be determined:

1) The dose of famitinib for subjects enrolled subsequently
2) The number of enrolled subjects for a certain tumor type or cohort

The first interim analysis will be conducted after the 2-cycle (6-weeks) observation (start after the first dose) for the first 12 subjects enrolled in Cohorts 1-5 is completed. The dose reduction of famitinib in enrolled subjects and the clinically significant toxicities in subjects of Cohorts 1-5 who have underwent 2-cycle observation during the first 2 treatment cycles of the study will be analyzed:

If 4 or more subjects experience clinically significant toxicity (incidence $\geq 0.33$) in the first 2 cycles or if the rate of famitinib dose reduction in subjects enrolled in Cohorts 1-5 is > 30%, it will be considered that continuous famitinib administration at 20 mg/d is not well tolerated. The dose of famitinib for subjects subsequently enrolled in the study will be reduced to 15 mg/d.

In addition, for Cohorts 1-5, other interim analyses will be performed after enrollment of 22 subjects for a certain tumor type is completed and at least one efficacy evaluation is completed after administration. The efficacy analysis will be based on response rates (including unconfirmed CR/PR). If tumor response (confirmed or unconfirmed) is observed in $\geq 3$ of 22 subjects, enrollment of this tumor type will be continued until 33-53 subjects are enrolled.

For a certain tumor type where 33-53 subjects are enrolled and the last subject completes at least 2 efficacy evaluations after treatment observation and enrollment (or at least 18 weeks after the last subject's enrollment), the previously accumulated efficacy data will be analyzed. The efficacy analysis will be based on objective response rate (ORR). Further sample size expansion for this tumor type will be determined based on efficacy results.

For each tumor type in Cohorts 6 and 7, other interim analyses will be performed after enrollment of 14 subjects for a certain cohort is completed and at least one efficacy evaluation is completed after administration. The efficacy analysis will be based on response rates (including unconfirmed CR/PR). If tumor response (confirmed or unconfirmed) is observed in $\geq 2$ of 14 subjects, enrollment of this cohort will be continued until 23 subjects are enrolled.

For the tumor type of a certain cohort where 23 subjects are enrolled and the last subject completes at least 2 efficacy evaluations after treatment observation and enrollment (or at least 18 weeks after the last subject's enrollment), the previously accumulated efficacy data will be analyzed. The efficacy analysis will be based on objective response rate (ORR). Further studies of this cohort will be determined based on efficacy results.

For Cohort 8, other interim analyses will be performed after enrollment of 21 subjects for a certain cohort is completed and at least one efficacy evaluation is completed after administration. The efficacy analysis will be based on response rates (including unconfirmed CR/PR). If tumor response (confirmed or unconfirmed) is observed in ≥ 7 of 21 subjects, enrollment of this cohort will be continued until 47 subjects are enrolled.

For the tumor type of this cohort where 47 subjects are enrolled and the last subject completes at least 2 efficacy evaluations after treatment observation and enrollment (or at least 18 weeks after the last subject's enrollment), the previously accumulated efficacy data will be analyzed. The efficacy analysis will be based on objective response rate (ORR). Further studies of this cohort will be determined based on efficacy results.

# 11. DATA MANAGEMENT METHOD

## 11.1. Data Recording

In this study, electronic case report forms (eCRFs) will be used to collect and manage clinical data.

### 11.1.1. Filing of study medical records

As source documents, the medical records should be completely retained. The investigators are responsible for completing and keeping medical records. Before filling in, the subject's information on the cover of medical records should be checked. The handwriting should be neat and legible in order that the sponsor's CRA can verify the data against those in the eCRF.

### 11.1.2. eCRF entry

Clinical study data will be collected using the HRTAU EDC system.

Data entry: Data in the eCRF are derived from source documents, e.g., medical records and laboratory test reports, and should be consistent with these source documents. Any observations or test results in the study should be entered in the eCRF in a timely, accurate, complete, clear, normative, and verifiable manner. Data should not be changed arbitrarily. All items in the eCRF should be filled out, with no blank or omission.

Modifications: The system instructions must be followed when correcting the eCRF data as needed, and the reason for data correction must be recorded. The logic verification program in the system will verify the integrity and logic of the clinical data entered into the EDC system and generate a prompt message for problematic data. The principal investigators (PIs) or clinical research coordinator (CRC) is permitted to modify or explain the problematic data. If necessary, multiple inquiries can be raised until the problematic data are resolved.

#### 11.1.3. eCRF review

The investigators or designated personnel should complete, review, and submit the eCRF soon after the end of visit for each subject, and promptly respond to queries raised by the CRA, data manager, and medical reviewer. After data cleaning is completed, the investigators will sign the completed eCRF for verification.

### 11.2. Data Monitoring

The clinical study associate (CRA) is responsible for monitoring whether the study is carried out in accordance with relevant regulations, the GCP, and study protocol; whether the eCRF is entered accurately and completely, and consistent with source documents such as medical records and laboratory test reports, and free from errors and omissions. According to the monitoring plan, the CRA will verify the completeness, consistency, and accuracy of study data in the database. The CRA will discuss problematic data with study personnel and direct them to add or correct the data whenever necessary. Ensure that the data in the eCRF are consistent with source data. This process is also known as source data verification (SDV).

### 11.3. Data Management

#### 11.3.1. EDC database establishment

The data manager will establish a study data collection system and database according to the study protocol, which will be available for online usage before the first subject is enrolled. All the EDC users must have completed relevant trainings and finished training records and account application forms before they can obtain the corresponding account number to log in the system. Note that the investigators are required to provide the laboratory reference ranges and the signature page of the investigators' electronic signature statement.

#### 11.3.2. Data entry and verification

The investigators or CRC should input data into the EDC system in accordance with the requirements of the visit procedures and the eCRF completion guideline. After submitting the eCRF, the CRA, data manager, and medical reviewer should review the data. Questions during the review will be submitted to the investigators or CRC in the form of queries. After data cleaning is completed, the investigators should sign the completed eCRF for verification.

### 11.3.3. Database lock

After SDV is completed by the CRA, the data manager and medical reviewer will conduct a final quality control of all data in the database, summarize all protocol deviations and violations during the study, and hold the data review meeting. The database will be locked after quality requirements are met. The data manager will export the data to the statistics department for data analysis.

### 11.3.4. Data archiving

After the study is completed, subject's eCRF in PDF format must be generated from the EDC system and kept in CD-ROMs. These CD-ROMs will be archived by the sponsor and various institutions for auditing.

The keeping and management of the study data must be carried out according to the GCP requirements. Before the destruction of any study-related documents or data, the investigators must inform the sponsor in advance. The sponsor must keep the clinical data until at least 5 years after the investigational product is approved for marketing.

### 11.3.5. Protocol deviation

Protocol deviation refers to any practice that does not comply with study protocol or GCP. This non-compliance may occur in the subject and may also occur in the investigators or other study personnel. Study sites should prepare corresponding corrective measures and implement them immediately if a protocol deviation occurs.

Study sites have the responsibility to maintain constant vigilance, complete the identification of protocol deviations in a timely manner, and complete the actions required by the protocol, to identify and report protocol deviations in a timely manner. All protocol deviations must be documented in the source documents. Protocol deviations must be submitted to local IRB in accordance with local ethical regulations. The principal investigators or study personnel of study sites are responsible for understanding and complying with local ethical standards.

## 12. SOURCE DATA AND DOCUMENTS

According to ICH E6, relevant regulations, and requirements for subjects' personal information protection of the study sites, each study site must properly keep all the treatment and scientific research records related to this study. As a part of the study that Hengrui sponsors or participates in, each study site must allow the authorized representative of Hengrui and regulatory authorities to inspect the clinical records (which may be copied if permissible by law) for quality review, audit, and evaluations of safety, study progress, and data validity.

Source data are information required to reconstruct and evaluate the clinical study, and are the original documentation of clinical findings, observations, and other activities. These source documents and data records include but are not limited to: hospital record, laboratory records, memos, subject diary cards, pharmacy dispensing records, recordings of advisory meetings, recorded data from automated devices, copies or transcripts that are verified to be accurate and intact, microfiche, photographic negatives, microfilms or magnetic disks, X-ray films, and subject's documents and records that are kept in the pharmacies, laboratories, and medical technology departments that are involved in this study.

# 13. QUALITY ASSURANCE AND QUALITY CONTROL

To ensure study quality, the sponsor and the investigators will jointly discuss and formulate a clinical study plan before the formal study initiation. All study personnel participating in the study will receive GCP training.

All the study sites must comply with the SOPs for the management of the investigational products, including receipt, storage, dispensation, return, and destruction (if applicable).

According to the GCP guidelines, necessary measures must be taken at the design and implementation phases of the study to ensure that all collected data are accurate, consistent, intact, and reliable. All observed results and abnormal findings in the clinical study must be verified and recorded in a timely manner to ensure data reliability. All devices, equipment, reagents, and standards used in various tests in the clinical study must have stringent specifications and be operated under normal conditions.

The investigators will input data required by the protocol into the eCRF. The CRA will check whether the eCRF is completely and accurately filled and guide the study site personnel for necessary correction and addition.

The drug regulatory authorities, Institutional Review Board (IRB)/Independent Ethics Committee (IEC), sponsor's CRA and/or auditor may carry out systemic inspection of study-related activities or documents to assess whether the study is implemented based on the study protocol, SOPs, and relevant regulations (such as Good Laboratory Practices [GLP] and Good Manufacturing Practices [GMP]) and whether the study data are recorded in a prompt, truthful, accurate, and complete manner. The audit should be performed by personnel not directly involved in this clinical study.

# 14. REGULATORY ETHICS, INFORMED CONSENT, AND SUBJECT PROTECTION

## 14.1. Regulatory Considerations

According to the corresponding regulatory requirements in China, an application should be submitted to the CFDA (now NMPA) before starting a new drug study and the study can only be carried out after approval is obtained. The clinical approval number for SHR-1210 is 2016L01455. The clinical approval numbers for famitinib malate are 2012L02340, 2012L2341, and 2013L1867.

The legal basis for the design of this protocol is as follows:

1) "Provisions for Drug Registration"

2) "Good Clinical Practice"

3) "Technical Guidelines for Clinical Pharmacokinetic Study of Chemical Drugs"

4) Consensus on ethical principles based on international ethics guidelines, including the Declaration of Helsinki and the Council for International Organizations of Medical Sciences (CIOMS) International Ethics Guidelines

5) ICH Guidelines

6) Other applicable laws and regulations

## 14.2. Ethical Standards

This study protocol must first be reviewed and approved in writing by the ethics committee of the hospital before being implemented. The study protocol, protocol amendments, ICF, and other relevant documents such as recruitment advertisements should be submitted to the ethics committee. This clinical study must comply with the "Declaration of Helsinki", CFDA's (now NMPA) "Good Clinical Practice" (GCP), and other relevant regulations. Before the study is initiated, approval must be obtained from the ethics committee of the hospital.

The study protocol must not be unilaterally modified without approvals from both the sponsor and the investigators. The investigators can modify or deviate from the study protocol before obtaining an approval from the ethics committee/institutional review board only when in purpose of eliminating direct and immediate harm to the subject. Besides, the deviation or change and the corresponding reason, and the recommended protocol amendment should be submitted to the EC/IRB for review. The investigators must provide explanations and document any protocol deviation.

During the study, any changes to this study protocol must be submitted to the ethics committee. If necessary, corresponding changes should be simultaneously made to other study documents and be submitted and/or approved according to the pertinent requirements of the ethics committee. The investigators are responsible for submitting the interim reports regularly according to the pertinent requirements of the ethics committee. After the end of study, the completion should be informed to the ethics committee.

## 14.3. Independent Ethics Committee

The protocol, ICF, recruitment material, and all subject materials must be reviewed and approved by the ethics committee. Subjects may be enrolled only after the protocol and ICF have been approved. Any revisions to the protocol must be reviewed and approved by the ethics committee prior to being implemented. All revisions to the ICF must be approved by the ethics committee, who will decide whether the subjects who have signed the previous version of the ICF are required to sign the new one.

## 14.4. Informed Consent

### 14.4.1. Informed consent form and other written information for subjects

Hengrui will provide a suitable ICF sample to the investigators, including all the key elements required by ICH, GCP, and regulatory authorities. The ICF sample will adhere to the ethical principles mentioned in the "Declaration of Helsinki".

The ICF sample will describe the study medication and study process in detail and fully explain the risks of the study to the subjects. Written ICFs must be obtained before the start of study treatment.

All revisions to the ICF must be approved by the ethics committee, who will decide whether the subjects who have signed the previous version of the ICF are required to sign the new one.

The ICF should state that the identity of the subjects must be remained confidential, but the medical representative of Hengrui and regulatory authorities are allowed to access the information.

### 14.4.2. Informed consent process and records

Informed consent will begin before an individual decides to participate in the clinical study and continues during the entire clinical study. The risks and potential benefits of participating in the study should be discussed fully and in detail with subjects or their legally acceptable representatives. Subjects will be asked to read and review the ICF that has been approved by the ethics committee. The investigators will explain the clinical study to subjects and answer any questions posed by subjects. Subjects can only participate in the study after they have signed the ICF. During the clinical study, subjects can withdraw the informed consents at any time. One copy of the signed ICF will be kept by subjects. Even if a patient refuses to participate in this study, his or her rights will be fully protected, and the nursing quality will not be affected.

## 14.5. Confidentiality of Subject Information

The confidentiality of subject information will be strictly enforced by the investigators, participated study personnel, and sponsor and its representative. In addition to the clinical information, confidentiality also simultaneously covers biosamples and genetic tests of the subjects. Therefore, the study protocol, documentation, data, and other information generated from these materials will be kept strictly confidential. All relevant study or data information should not be disclosed to any unauthorized third-party without prior written approval from the sponsor.

Other authorized representatives of the sponsor, IRB or regulatory authorities, and the representatives of the pharmaceutical company that provides the investigational products can examine all the documents and records that are maintained by the investigators, including but not limited to the medical records and subject's administration records. Study sites should allow access to these records.

The contact information of the subjects will be safely kept in each study site and only used internally during the study. When the study is ended, all the records will be kept in a secure place based on the time limit specified by local IRB and regulations.

Subject's study data that are collected for statistical analysis and scientific reports will be uploaded and kept in the HRTAU EDC system. This should not include the contact information or identification information of subjects. Instead, individual subjects and their study data will be given a unique study identification number. The study data entry and study management system used by the study personnel at study sites should be confidential and password-protected. At the end of study, all identification information in the study database will be erased and archived in the study site.

# 15. PUBLICATION OF STUDY RESULTS

The study results belong to Jiangsu Hengrui Pharmaceuticals Co., Ltd. If the investigators plan to publish any study-related data and information, Hengrui should be provided with the manuscript, abstract, or full text of all planned publications (poster, invited lectures, or guest lectures) at least 30 days prior to the submission of documents for publication or other forms of release.

# 16. CLINICAL STUDY PROGRESS

Anticipated enrollment of the first subject: Oct. 2018

Anticipated enrollment of the last subject: Jun. 2021

Anticipated study completion: Dec. 2021

# 17. REFERENCES

1. Khan KA, Kerbel RS: Improving immunotherapy outcomes with anti-angiogenic treatments and vice versa. Nat Rev Clin Oncol 15:310-324, 2018
2. Zhou C, Gao G, Wu F, et al: A phase Ib study of SHR-1210 plus apatinib for heavily previously treated advanced non-squamous non-small cell lung cancer (NSCLC) patients, American Society of Clinical Oncology, 2018
3. Xu J-M, Zhang Y, Jia R, et al: Anti-programmed death-1 antibody SHR-1210 (S) combined with apatinib (A) for advanced hepatocellular carcinoma (HCC), gastric cancer (GC) or esophagogastric junction (EGJ) cancer refractory to standard therapy: A phase 1 trial, American Society of Clinical Oncology, 2018
4. Petrylak DP, de Wit R, Chi KN, et al: Ramucirumab plus docetaxel versus placebo plus docetaxel in patients with locally advanced or metastatic urothelial carcinoma after platinum-based therapy (RANGE): overall survival and updated results of a randomised, double-blind, phase 3 trial. Lancet Oncol 21:105-120, 2020
5. Apolo AB, Nadal R, Tomita Y, et al: Cabozantinib in patients with platinum-refractory metastatic urothelial carcinoma: an open-label, single-centre, phase 2 trial. Lancet Oncol 21:1099-1109, 2020
6. Bellmunt J, Gonzalez-Larriba JL, Prior C, et al: Phase II study of sunitinib as first-line treatment of urothelial cancer patients ineligible to receive cisplatin-based chemotherapy: baseline interleukin-8 and tumor contrast enhancement as potential predictive factors of activity. Ann Oncol 22:2646-2653, 2011
7. Tewari KS, Sill MW, Long HJ, 3rd, et al: Improved survival with bevacizumab in advanced cervical cancer. N Engl J Med 370:734-43, 2014
8. Rini BI, Plimack ER, Stus V, et al: Pembrolizumab plus Axitinib versus Sunitinib for Advanced Renal-Cell Carcinoma. N Engl J Med 380:1116-1127, 2019
9. Motzer RJ, Penkov K, Haanen J, et al: Avelumab plus Axitinib versus Sunitinib for Advanced Renal-Cell Carcinoma. N Engl J Med 380:1103-1115, 2019
10. Lee C-H, Shah AY, Hsieh JJ, et al: Phase II trial of lenvatinib (LEN) plus pembrolizumab (PEMBRO) for disease progression after PD-1/PD-L1 immune checkpoint inhibitor (ICI) in metastatic clear cell renal cell carcinoma (mccRCC). 38:5008-5008, 2020
11. Lin Y, Shih WJ: Adaptive two-stage designs for single-arm phase IIA cancer clinical trials. Biometrics 60:482-90, 2004
12. Koyama T, Chen H: Proper inference from Simon's two-stage designs. Stat Med 27:3145-54, 2008

# Appendix I Performance Status (ECOG)

(Eastern Cooperative Oncology Group)

| Score | Description |
|---|---|
| 0 | Asymptomatic, fully active, able to carry on all performance without restriction. |
| 1 | Symptomatic, restricted in physically strenuous activity but ambulatory and able to carry out work of a light or sedentary nature, e.g., light house work, office work. |
| 2 | Symptomatic, ambulatory and capable of all self-care but unable to carry out any physical activities; up and about more than 50% of waking hours (confined to bed < 50% of waking hours). |
| 3 | Symptomatic, capable of only limited self-care; confined to bed or chair more than 50% of waking hours, but not totally confined to bed. |
| 4 | Completely disabled; cannot carry on any self-care; totally confined to bed or chair. |
| 5 | Dead. |

Oken, M.M., Creech, R.H., Tormey, D.C., Horton, J., Davis, T.E., McFadden, E.T., Carbone, P.P.: Toxicity And Response Criteria Of The Eastern Cooperative Oncology Group. Am J Clin Oncol 5: 649 - 655, 1982

# Appendix II Response Evaluation Criteria in Solid Tumors (RECIST v1.1)

Response Evaluation Criteria in Solid Tumors Version **1.1** (Excerpt)

(New Response Evaluation Criteria in Solid Tumors: Revised RECIST Version 1.1)

Note: This appendix is translated internally and is for reference only. Please refer to the English version during practice.

## 1 BACKGROUND

Omitted

## 2 PURPOSE

Omitted

## 3 MEASURABILITY OF TUMOR AT BASELINE

### 3.1 Definitions

At baseline, tumor lesions/lymph nodes will be categorized measurable or non-measurable as follows:

#### 3.1.1 Measurable

Tumor lesions: Must be accurately measured in at least one dimension (longest diameter is to be recorded) with a minimum size of:

- 10 mm by CT scan (CT scan slice thickness no greater than 5 mm)
- 10 mm caliper measurement by clinical exam (lesions which cannot be accurately measured with calipers should be recorded as non-measurable)
- 20 mm by chest X-ray
- Malignant lymph nodes: pathologically enlarged and measurable, single lymph node must be ≥ 15 mm in short axis when assessed by CT scan (CT scan slice thickness recommended to be no greater than 5 mm). At baseline and during follow-up, only the short axis will be measured and followed.

### 3.1.2 Non-measurable

All other lesions, including small lesions (longest diameter < 10 mm or pathological lymph nodule with ≥ 10 mm to < 15 mm short axis) as well as truly non-measurable lesions. Non-measurable lesions include: meningeal disease, ascites, pleural or pericardial effusion, inflammatory breast cancer, lymphangitis carcinomatosa of the skin or lung, abdominal masses unable to be diagnosed or followed by imaging techniques, and cystic lesions.

### 3.1.3 Special considerations regarding lesion measurability

Bone lesions, cystic lesions, and lesions previously treated with local therapy require particular comment:

Bone lesions:

- Bone scan, PET scan or plain films are not considered adequate to measure bone lesions. However, these techniques can be used to confirm the presence or disappearance of bone lesions;
- Lytic lesions or mixed lytic-blastic lesions, with identifiable soft tissue components, that can be evaluated by tomography techniques such as CT or MRI can be considered as measurable lesions if the soft tissue component meets the definition of measurability described above;
- Blastic lesions are non-measurable.

Cystic lesions:

- Lesions that meet the criteria for radiographically defined simple cysts should not be considered as malignant lesions (neither measurable nor non-measurable) since they are, by definition, simple cysts;
- Cystic lesions thought to represent cystic metastases can be considered as measurable lesions, if they meet the definition of measurability described above. However, if noncystic lesions are present in the same patient, these are preferred for selection as target lesions.

Lesions with prior local treatment:

- Tumor lesions situated in a previously irradiated area, or in an area subjected to other locoregional therapy, are usually considered non-measurable unless there has been demonstrated progression in the lesion. Study protocols should detail the conditions under which such lesions would be considered measurable.

### 3.2 Specifications by Methods of Measurements

#### 3.2.1 Measurement of lesions

All measurements should be recorded in metric notation if clinically assessed. All baseline evaluations should be performed as close as possible to the treatment start and never more than 28 days (4 weeks) before the beginning of the treatment.

#### 3.2.2 Method of assessment

The same method and technique should be used to assess lesions at baseline and during follow-up. Imaging based evaluation should always be done rather than clinical examination unless the lesion(s) being followed cannot be imaged but are assessable by clinical exam.

Clinical lesions: Clinical lesions will only be considered measurable when they are superficial and ≥ 10 mm diameter as assessed using calipers (e.g. skin nodules). For the case of cutaneous lesions, documentation by color photography including a ruler to estimate the size of the lesion is suggested. When lesions can be evaluated by both imaging and clinical examination, imaging evaluation should be undertaken since it is more objective and may also be reviewed at the end of study.

Chest X-ray: Chest CT is preferred over chest X-ray, especially when tumor progression is an important clinical endpoint, since CT is more sensitive, particularly in identifying new lesions. Chest X-ray is only applicable when the measured lesion boundary is clear and the lungs are well ventilated.

CT and MRI: CT is currently the best available and reproducible method for response evaluation. This guideline has defined measurability of lesions on CT scan based on the assumption that CT slice thickness is ≤ 5 mm. When CT scans have slice thickness greater than 5 mm, the minimum size for a measurable lesion should be twice the slice thickness. MRI is also acceptable in certain situations (e.g. for whole body scans).

Ultrasound: Ultrasound should not be used as a method to measure lesion size. Ultrasound examinations are operation-dependent, and cannot be reproduced at a later date. It cannot be guaranteed that the same technique and measurements will be taken from one assessment to the next. If new lesions are identified by ultrasound in the course of the study, confirmation by CT or MRI is advised. If there is concern about radiation exposure at CT, MRI may be used instead.

Endoscopy and laparoscopy: The utilization of these techniques for objective tumor evaluation is not advised. However, they can be useful to confirm CR when biopsies are obtained, or to determine relapse in trials where recurrence following CR or surgical excision is an endpoint.

Tumor biomarkers: Tumor biomarkers alone cannot be used to assess objective tumor response. However, if the marker levels exceed the upper normal limit at baseline, they must return to the normal levels for evaluation of complete response. Because tumor biomarkers are disease specific, instructions for their measurement should be incorporated into protocols on a disease specific basis. Specific guidelines for both CA-125 response (in recurrent ovarian cancer) and PSA response (in recurrent prostate cancer) have been published. In addition, the Gynecologic Cancer Intergroup has developed CA-125 progression criteria which are to be integrated with objective tumor evaluation for use in first-line studies in ovarian cancer.

Cytology/Histology: These techniques can be used to differentiate between PR and CR in certain cases specified in the protocol (e.g., residual benign tumor tissue is often present in the lesions of germ cell tumors). When effusions are known to be a potential adverse effect of treatment (e.g., with certain taxane compounds or angiogenesis inhibitors), the cytological confirmation of the neoplastic origin of any effusion that appears or worsens during treatment can be considered if the measurable tumor has met the criteria for response or stable disease in order to differentiate between response (or stable disease) and PD.

# 4 TUMOR RESPONSE EVALUATION

## 4.1 Assessment of Overall Tumor Burden and Measurable Disease

To assess objective response or future progression, it is necessary to estimate the overall tumor burden at baseline and use this as a comparator for subsequent measurements. Only patients with measurable lesions at baseline should be included in protocols where objective response is the primary endpoint. Measurable lesion is defined by the presence of at least one measurable lesion. In trials where the primary endpoint is tumor progression (either time to progression or proportion with progression at a fixed date), the protocol must specify if enrollment is restricted to those with measurable lesions or whether patients with non-measurable lesions are also eligible.

## 4.2 Baseline Documentation of "Target" and "Non-Target" Lesions

When more than one measurable lesion is present at baseline, all lesions up to a maximum of five lesions total (and a maximum of two lesions per organ), representative of all involved organs should be identified as target lesions and will be recorded and measured at baseline (this means in instances where subjects have only one or two organ sites involved, a maximum of two and four lesions respectively will be recorded).

Target lesions should be selected on the basis of their size (lesions with the longest diameter), be representative of all involved organs, but in addition should be those that lend themselves to reproducible repeated measurements. It may be the case that, on occasion, the largest lesion does not lend itself to reproducible measurement in which circumstance the next largest lesion which can be measured reproducibly should be selected.

Lymph nodes merit special mention since they are normal tissues which may be visible by imaging even if not involved by tumor metastasis. Pathological nodes which are defined as measurable and may be identified as target lesions must meet the criterion of a short axis of ≥ 15 mm by CT scan. Only the short axis of these nodes needs to be measured at baseline. The short axis of a node is the diameter normally used by radiologists to judge if the node is involved by tumor metastasis. Nodule size is normally reported as two dimensions in the plane in which the image is obtained (for CT scan this is almost always the axial plane; for MRI the plane of acquisition may be axial, sagittal or coronal). The smallest of these measures is the short axis. For example, an abdominal node which is reported as being 20 mm × 30 mm has a short axis of 20 mm and qualifies as a malignant, measurable node. In this example, 20 mm should be recorded as the node measurement. Nodes with short axis ≥ 10 mm but < 15 mm should be considered non-target lesions. Nodes that have a short axis < 10 mm are considered non-pathological and are thus not to be recorded or followed up.

A sum of the diameters (longest for non-nodal lesions, short axis for nodal lesions) for all target lesions will be calculated and reported as the baseline sum diameters. If lymph nodes are to be included in the sum, then as noted above, only the short axis is added into the sum. The baseline sum diameters will be used as reference.

All other lesions including pathological lymph nodes should be identified as non-target lesions, and while measurements are not required, they should be recorded at baseline. These lesions should be recorded as "present", "absent", or in rare cases "unequivocal progression". It is possible to record multiple non-target lesions involving the same organ as a single item on the case record form (e.g., "multiple enlarged pelvic lymph nodes" or "multiple liver metastases").

## 4.3 Response Criteria

### 4.3.1 Evaluation of target lesions

Complete response (CR): Disappearance of all target lesions. Any pathological lymph nodules (whether target or non-target) must have reduction in short axis to < 10 mm.

Partial response (PR): At least a 30% decrease in the sum of diameters of target lesions, compared with baseline.

Progressive disease (PD): At least a 20% increase in the sum of diameters of target lesions, taking as reference the smallest sum on study (this includes the baseline sum if that is the smallest on study). In addition, the sum must also demonstrate an absolute increase of at least 5 mm (the appearance of one or more new lesions is also considered PD).

Stable disease (SD): Neither sufficient shrinkage to qualify for PR nor sufficient increase to qualify for PD, taking as reference the smallest sum diameters while on study.

#### 4.3.2 Special notes on the assessment of target lesions

Lymph nodes: Lymph nodes identified as target lesions should always have the actual short axis measurement recorded (measured in the same anatomical plane as the baseline examination), even if the nodes regress to below 10 mm on study. This means that when lymph nodes are included as target lesions, the sum of lesions may not be zero even if CR criteria are met, since a normal lymph node is defined as having a short axis < 10 mm. CRFs or other data collection methods may therefore be designed to have target nodal lesions recorded in a separate section where, in order to qualify for CR, each node must achieve a short axis < 10 mm. For PR, SD and PD, the actual short axis measurement of the nodes is to be included in the sum of target lesions.

Target lesions that become too small to measure: While on study, all lesions (nodal and non-nodal) recorded at baseline should have their actual measurements recorded at each subsequent evaluation, even when very small (e.g., 2 mm). However, sometimes lesions or lymph nodes which are recorded as target lesions at baseline become so faint on CT scan that the radiologist may not feel comfortable assigning an exact measure and may report them as being "too small to measure". When this occurs it is important that a value be recorded on the CRF. If it is the opinion of the radiologist that the lesion has likely disappeared, the measurement should be recorded as 0 mm. If the lesion is believed to be present and is faintly seen but too small to measure, a default value of 5 mm could be assigned. (Note: It is less likely that this rule will be used for lymph nodules since they usually have a definable size when normal and are frequently surrounded by adipose tissues as in the retroperitoneum; however, if a lymph nodule is believed to be present and is faintly seen but too small to measure, a default value of 5 mm could be assigned in this circumstance as well). This default value is derived from the 5 mm CT slice thickness (but should not be changed with varying CT slice thickness). The measurement of these lesions is potentially non-reproducible, and therefore providing this default value will prevent false evaluation based upon measurement error. To reiterate, however, if the radiologist is able to provide an actual measure, that should be recorded, even if it is below 5 mm.

Lesions that split or coalesce: When non-nodal lesions fragment, the longest diameters of the fragmented portions should be added together to calculate the target lesion sum. Similarly, as lesions coalesce, a plane between them may be maintained that would aid in obtaining maximal diameter measurements of each individual lesion. If the lesions have truly coalesced such that they are no longer separable, the vector of the longest diameter in this instance should be the maximal longest diameter for the coalesced lesion.

### 4.3.3 Evaluation of non-target lesions

This section provides the definitions of the criteria used to determine the tumor response for the group of non-target lesions. While some non-target lesions may actually be measurable, they need not be measured and instead should be assessed only qualitatively at the time points specified in the protocol.

Complete response (CR): Disappearance of all non-target lesions and normalization of tumor biomarker level. All lymph nodules must be non-pathological in size (< 10 mm short axis).

Non-CR/Non-PD: Persistence of one or more non-target lesion(s) and/or maintenance of tumor biomarker level above the normal limits.

Progressive disease (PD): Unequivocal progression of existing non-target lesions. Note: The appearance of one or more new lesions is also considered PD.

### 4.3.4 Special notes on assessment of progression of non-target disease

The concept of progression of non-target disease requires additional explanation as follows: When the patient also has measurable disease, to achieve unequivocal progression on the basis of the non-target disease, there must be an overall level of substantial worsening in non-target disease such that the overall tumor load has increased sufficiently to the point where treatment must be discontinued. A modest increase in the size of one or more non-target lesions is usually not sufficient to qualify for unequivocal progression status. The designation of overall progression solely on the basis of change in non-target disease in the face of SD or PR of target disease will therefore be extremely rare.

When the patient has only non-measurable disease: This circumstance arises in some phase III trials when it is not a criterion of study inclusion to have measurable disease. The same general concepts apply here as noted above; however, in this instance there is no measurable disease assessment. Because worsening in non-target disease cannot be easily quantified (by definition: if all lesions are truly non-measurable), a useful test that can be applied when assessing patients for unequivocal progression is to consider if the increase in overall disease load based on the change in non-measurable disease is comparable in magnitude to the increase that would be

required to declare PD for measurable disease. For example, an increase in tumor burden representing an additional 73% increase in volume (which is equivalent to a 20% increase diameter in a measurable lesion). Examples include an increase in a pleural effusion from "trace" to "large", an increase in lymphangitic disease from "localized" to "widespread", or may be described in protocols as "sufficient to require a change in treatment". Examples include an increase in a pleural effusion from trace to large, an increase in lymphangitic disease from localized to widespread, or may be described in protocols as "sufficient to require a change in therapy". If unequivocal progression is seen, the patient should be considered to have had overall PD at that point. While it would be ideal to have objective criteria to apply to non-measurable disease, the very nature of that disease makes it impossible to do so, and therefore the increase must be substantial.

### 4.3.5 New lesions

The appearance of new malignant lesions denotes PD; therefore, some comments on detection of new lesions are important. There are no specific criteria for the identification of radiographically detected lesions; however, the finding of a new lesion should be unequivocal. For example, it should not be attributable to differences in scanning technique, change in imaging modality, or findings thought to represent something other than tumor (for example, some new bone lesions that may be simply healing, or re-occurrence of pre-existing lesions). This is particularly important when the patient's baseline lesions show partial or complete response. For example, necrosis of a liver lesion may be reported on a CT scan report as a new cystic lesion, which it is not.

A lesion identified on a follow-up study that is not scanned at baseline will be considered a new lesion and will indicate PD. An example of this is the patient who has visceral disease at baseline and while on study has a CT or MRI brain ordered which reveals metastases. The patient's brain metastases are considered to be evidence of PD even if he/she did not have brain imaging at baseline.

If a new lesion is equivocal, for example, because of its small size, continued treatment and follow-up evaluation are required to clarify if it represents a truly new disease. If repeated scans confirm there is definitely a new lesion, then progression should be declared using the date of the initial identification.

While FDG-PET response assessments generally need additional study, it is sometimes reasonable to incorporate the use of FDG-PET scanning to complement CT scanning in assessment of progression (particularly possible new disease). New lesions on the basis of FDG-PET imaging can be identified according to the following process:

Negative FDG-PET at baseline, with a positive FDG-PET at follow-up is a sign of PD based on a new lesion.

No FDG-PET at baseline and a positive FDG-PET at follow-up:

If the positive FDG-PET at follow-up corresponds to a new site of disease confirmed by CT, PD is confirmed.

If the positive FDG-PET at follow-up is not confirmed as a new site of disease on CT, additional follow-up CT scans are needed to determine if there is truly progression occurring at that site (if so, the date of PD will be the date of the initial abnormal FDG-PET scan).

If the positive FDG-PET at follow-up corresponds to a pre-existing site of disease on CT that is not progressing on the basis of the imaging examination, this is not PD.

### 4.4 Evaluation of Best Overall Response

The best overall response is the best response recorded from the start of the trial until the end of trial taking into account any necessary requirement for confirmation. On occasion a response may not be documented until after the end of treatment, so protocols should be clear if post-treatment assessments are to be considered in the evaluation of best overall response. Protocols must specify how any new treatment introduced before progression will affect best response evaluation. The patient's best overall response evaluation will depend on the findings of both target and non-target diseases and will also take into consideration the characteristics of new lesions. Furthermore, depending on the nature of the study and the protocol requirements, it may also require confirmatory measurement. Specifically, in non-randomized studies where response is the primary endpoint, confirmation of PR or CR is needed to determine either one is the best overall response.

#### 4.4.1 Time point response

It is assumed that at each time point specified in protocol, an efficacy response occurs. Table 1 provides a summary of the overall response status calculation at each time point for patients who have measurable disease at baseline.

**Table 1. Time point response: patients with target (+/– non-target) disease.**

| Target Lesions | Non-Target Lesions | New Lesions | Overall Response |
|---|---|---|---|
| CR | CR | No | CR |
| CR | Non-CR/non-PD | No | PR |
| CR | NE | No | PR |
| PR | Non-PD or not all evaluated | No | PR |

| Target Lesions | Non-Target Lesions | New Lesions | Overall Response |
|---|---|---|---|
| SD | Non-PD or not all evaluated | No | SD |
| Not All Evaluated | Non-PD | No | NE |
| PD | Any | Yes or No | PD |
| Any | PD | Yes or No | PD |
| Any | Any | Yes | PD |

CR = complete response, PR = partial response, SD = stable disease, PD = progressive disease, and NE = not evaluable

When patients have non-measurable (therefore non-target) disease only, Table 2 is to be used.

**Table 2. Time point response: patients with non-target disease only.**

| Non-Target Lesions | New Lesions | Overall Response |
|---|---|---|
| CR | No | CR |
| Non-CR/Non-PD | No | Non-CR/non-PD[a] |
| Not All Evaluated | No | NE |
| Equivocal PD | Yes or No | PD |
| Any | Yes | PD |

a: "Non-CR/non-PD" is preferred over SD for non-target disease since SD is increasingly used as endpoint for assessment of efficacy in some trials so to assign this category when no lesions can be measured is not advised.

### 4.4.2 Missing assessments and inevaluable designation

When no imaging/measurement is done at all at a particular time point, the patient is not evaluable (NE) at that time point. If only a subset of lesion measurements are made at an assessment, usually the case is also considered NE at that time point, unless a convincing argument can be made that the contribution of the individual missing lesion(s) would not change the assigned time point response. This would be most likely to happen in the case of PD. For example, if a patient had a baseline sum of 50 mm with three measured lesions and at follow-up only two lesions were assessed, but those gave a sum of 80 mm, the patient will have achieved PD status, regardless of the contribution of the missing lesion.

### 4.4.3 Best overall response: all time points

The best overall response is determined once all the data for the patient are known.

Best response determination in trials where confirmation of complete or partial response is not required: Best response in these trials is defined as the best response across all time points (for example, a patient who has SD in evaluation at Cycle 1, PR at Cycle 2, and PD at the last cycle has a best overall response of PR). When SD is believed to be best response, it must also meet the protocol specified minimum time calculated from baseline. If the minimum time is not met when SD is otherwise the best overall response, the patient's best overall response depends on

the subsequent assessments. For example, a patient who has SD at Cycle 1, PD at Cycle 2 and does not meet minimum duration for SD, will have a best overall response of PD. The same patient lost to follow-up after the first SD assessment would be considered inevaluable.

Best response determination in trials where confirmation of complete or partial response is required: Complete or partial responses may be claimed only if the criteria for each are met at a subsequent time point as specified in the protocol (generally 4 weeks later). In this circumstance, the BOR can be interpreted as in Table 3.

**Table 3. Best overall response when confirmation of CR and PR required.**

| Overall Response at First Time Point | Overall Response at Subsequent Time Point | Best Overall Response |
|---|---|---|
| CR | CR | CR |
| CR | PR | SD, PD, or PR[a] |
| CR | SD | SD (provided minimum criteria for SD duration met, otherwise, PD) |
| CR | PD | SD (provided minimum criteria for SD duration met, otherwise, PD) |
| CR | NE | SD (provided minimum criteria for SD duration met, otherwise, NE) |
| PR | CR | PR |
| PR | PR | PR |
| PR | SD | SD |
| PR | PD | SD (provided minimum criteria for SD duration met, otherwise, PD) |
| PR | NE | SD (provided minimum criteria for SD duration met, otherwise, NE) |
| NE | NE | NE |

CR = complete response, PR = partial response, SD = stable disease, PD = progressive disease, and NE = not evaluable.

a: If a CR is truly met at first time point, then any disease seen at a subsequent time point, even disease meeting PR criteria relative to baseline, makes the disease PD at that point (since disease must have reappeared after CR). Best response would depend on whether minimum duration for SD was met. However, sometimes CR may be claimed when subsequent scans suggest small lesions were likely still present and in fact the patient had PR, not CR at the first time point. Under these circumstances, the original CR should be changed to PR and the best response is PR.

### 4.4.4 Special notes on response assessment

When nodal disease is included in the sum of target lesions and the nodules decrease to a normal size < 10 mm, they may still have a measurement reported on scans. This measurement should be recorded even though the nodules are normal in order not to overstate progression should it be based on increase in size of the nodules. As noted earlier, this means that patients with CR may not have a total sum of zero on the CRF.

In trials where confirmation of response is required, repeated "NE" time point assessments may complicate best response determination. The analysis plan for the trial must address how missing data/assessments will be addressed in determination of response and progression. For example, in most trials it is reasonable to consider a patient with time point responses of PR-NE-PR as a confirmed response.

Patients with an overall deterioration of health status requiring discontinuation of treatment without objective evidence of disease progression at that time should be reported as symptomatic progression. Efforts should be made to evaluate objective progression even after discontinuation of treatment. Symptomatic deterioration is not a description of an objective response: it is a reason for discontinuation of treatment. The objective response status of such patients is to be determined by evaluation of target and non-target disease as shown in Tables 1-3.

Conditions that are defined as early progression, early death, and not evaluable are study specific and should be clearly described in each protocol (depending on treatment duration and treatment cycle).

In some circumstances it may be difficult to distinguish residual lesions from normal tissues. When the evaluation of complete response depends upon this definition, it is recommended to perform a biopsy before evaluating the efficacy of complete remission of local lesions. FDG-PET may be used to confirm a response to a CR in a manner similar to a biopsy in cases where a residual radiographic abnormality is thought to represent fibrosis or scarring. The use of FDG-PET in this circumstance should be prospectively described in the protocol and supported by disease specific medical literature for the indication. However, it must be acknowledged that both approaches may lead to false positive CR due to limitations of FDG-PET and biopsy resolution/sensitivity.

For equivocal findings of progression (e.g., very small and uncertain new lesions; cystic changes or necrosis in existing lesions), treatment may continue until the next scheduled evaluation. If at the next scheduled evaluation, progression is confirmed, the date of progression should be the earlier date when progression was suspected.

### 4.5 Frequency of Tumor Re-Evaluation

Frequency of tumor re-evaluation during treatment should be protocol-specific and consistent with the type and schedule of treatment. However, in the phase II studies where the beneficial effect of treatment is not known, follow-ups for every 6-8 weeks (timed to coincide with the end of a cycle) is reasonable. Interval adjustments can be justified in specific regimens or circumstances. The protocol should specify which organ sites are to be evaluated at baseline (usually those most likely to be involved with metastatic disease for the tumor type under study) and how often evaluations are repeated. Normally, all target and non-target sites are evaluated at each assessment. In selected circumstances, certain non-target organs may be evaluated less frequently. For example, bone scans may need to be repeated only when CR is identified in target disease or when progression in bone is suspected.

After the treatment, the need for tumor re-evaluations depends on whether the study has made the response rate or the time to an event (progression/death) an endpoint. If time to an event (e.g. TTP/DFS[1]/PFS) is the main endpoint of the study, then routine scheduled re-evaluation of protocol specified sites of disease is warranted. In randomized comparative studies in particular, the scheduled assessments should be performed as identified on a calendar schedule (for example: every 6-8 weeks on treatment or every 3-4 months after treatment) and should not be affected by delays in therapy, drug holidays or any other events that might lead to imbalance in a treatment group in the timing of disease assessment.

### 4.6 Confirmatory Measurement/Duration of Response

#### 4.6.1 Confirmation

In non-randomized trials where response is the primary endpoint, confirmation of PR and CR is required to ensure responses identified are not the result of measurement error. This will also permit appropriate interpretation of results in the context of historical data where response has traditionally required confirmation in such trials. However, in all other circumstances, i.e., in randomized trials (phase II or III) or studies where stable disease or progression are the primary endpoints, confirmation of response is not required since it will not add value to the interpretation of trial results. However, elimination of the requirement for response confirmation may increase the importance of central review to protect against bias, in particular in studies which are not blinded.

In the case of SD, measurements must have met the SD criteria at least once after study entry at a minimum interval (in general not less than 6-8 weeks) that is defined in the study protocol.

### 4.6.2 Duration of overall response

The duration of overall response will be measured from the time measurement criteria are first met for CR/PR (whichever is first recorded) until the first date that recurrent or PD is objectively documented (taking as reference for PD the smallest measurements recorded on study). The duration of overall complete response will be measured from the time criteria are first met for CR until the first date that recurrent or PD is truly documented.

### 4.6.3 Duration of stable disease

Stable disease is measured from the start of the treatment (in randomized trials, from date of randomization) until the criteria for progression are met, taking as reference the smallest sum on study (if the baseline sum is the smallest, this is the reference for calculation of PD). The clinical relevance of the duration of stable disease varies in different studies and diseases. If the proportion of patients achieving SD for a minimum period of time is an endpoint in a particular study, the protocol should specify the minimal time interval required between two measurements for determination of SD.

Note: The duration of response and stable disease as well as the progression-free survival are influenced by the frequency of follow-up after baseline evaluation. It is not in the scope of this guideline to define a standard follow-up frequency. The frequency should take into account many parameters including disease types and stages, treatment periodicity and standard practice. However, these limitations of the precision of the measured endpoint should be taken into account if comparisons between trials are to be made.

## 4.7 PFS/TTP

### 4.7.1 Phase II trials

This guideline is focused primarily on the use of objective response as study endpoints for phase II trials. In some circumstances, response rate may not be the optimal method to assess the potential anti-cancer activity of new agents/regimens. In such cases PFS/PPF at landmark time points, might be considered appropriate alternatives to provide an initial signal of biologic effect of new agents. It is clear, however, that in an uncontrolled trial, these measures are subject to criticism since an apparently promising observation may be related to biological factors such as patient selection and not the impact of the intervention. Thus, phase II screening studies utilizing these endpoints are best designed with a randomized control. Exceptions may exist where the behavior patterns of certain cancers are so consistent (and usually consistently poor) that a non-randomized trial is justifiable. However, in these cases it will be essential to document with care the basis for estimating the expected PFS or PPF[2] in the absence of a treatment effect.

2Proportion of progress-free

## Appendix III Percent Bone Marrow Content in Human Skeleton

**Percent Bone Marrow in the Adult Skeleton**

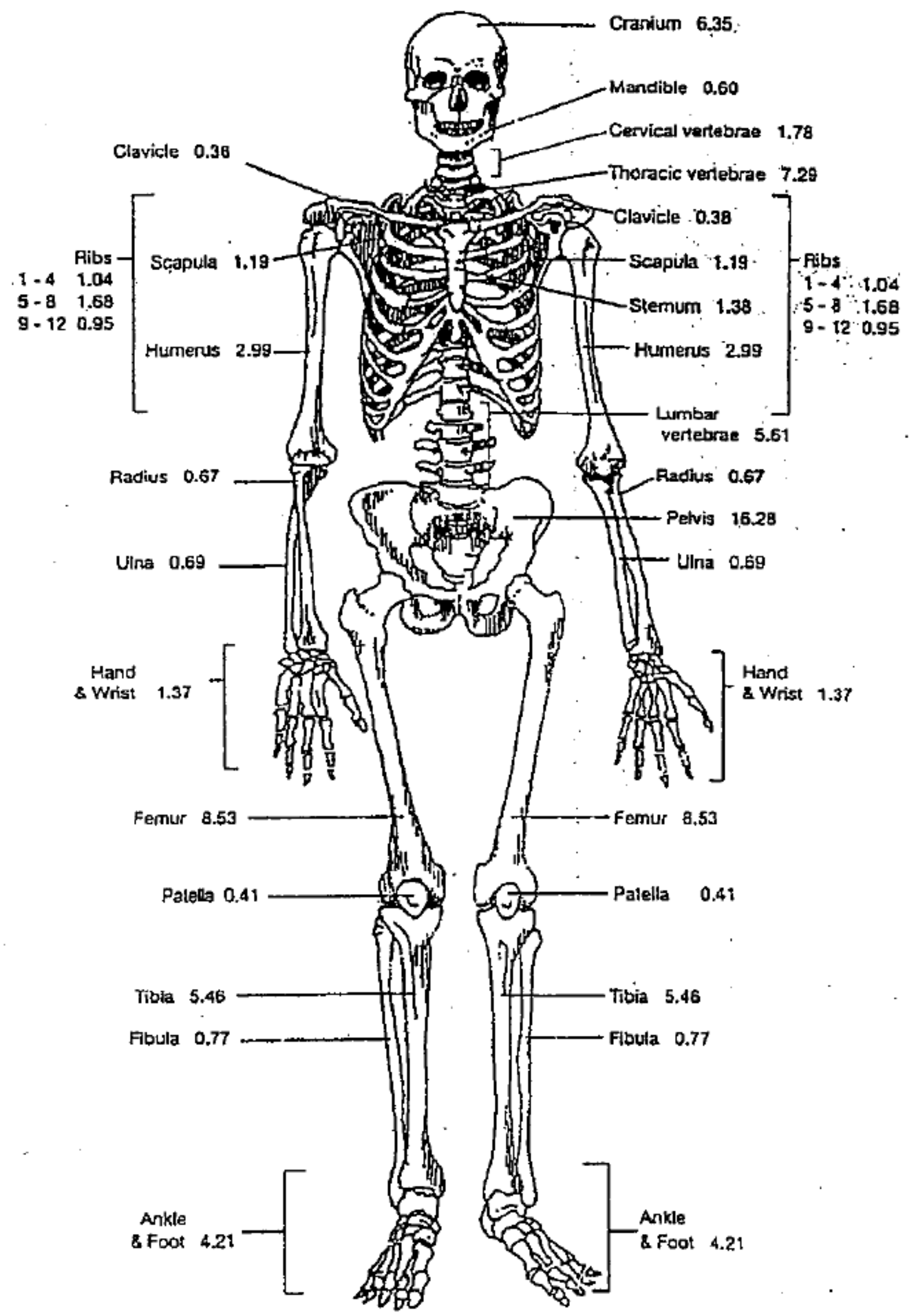


Woodward Holodny E. A summary of the data of Mechanik on the distribution of human bone marrow. Phys Med Biol. 1960;5:57-59

# Appendix IV Management Principles for Immune-Related Adverse Events

## 1. Management Principles for Gastrointestinal Adverse Events

**Non-inflammatory causes of disease should be excluded. Opioids/anesthetics may mask the symptoms of perforation. Do not use infliximab in the case of perforation/sepsis.**

| Diarrhea/Colitis Grade (NCI CTCAE v4) | Management | Follow-Up |
|---|---|---|
| **Grade 1**<br>Diarrhea: Increase of < 4 stools per day over baseline<br>Colitis: No symptom | • Continue I-O treatment as per study protocol<br>• Symptomatic treatment | • Closely monitor aggravated symptoms.<br>• Educate subjects to report aggravated symptoms immediately<br>If it is aggravated:<br>• Provide treatment according to the method for Grade 2 or Grade 3/4 situations |
| **Grade 2**<br>Diarrhea: Increase of 4-6 stools per day over baseline; intravenous infusion of < 24 h is required; daily living is not affected<br>Colitis: Abdominal pain; hematochezia | • Delay I-O treatment as per study protocol<br>• Symptomatic treatment | If it is improved to Grade 1:<br>• Resume I-O treatment as per study protocol<br>If it lasts for > 5-7 days or relapses:<br>• 0.5-1.0 mg/kg/day of methylprednisolone IV or equivalent PO<br>• When the symptoms improve to Grade 1, reduce the dose of steroids for at least 1 month and consider prophylactic antibiotics to prevent opportunistic infections, then resume I-O treatment as per study protocol<br>If the symptoms are aggravated or persisted after >3-5 days of oral administration of steroids:<br>• Provide treatment according to the method for Grade 3/4 situations |
| **Grade 3-4**<br>Diarrhea (Grade 3): Increase of ≥ 7 stools per day over baseline; fecal incontinence; intravenous infusion of ≥ 24 h is required; daily living is affected<br>Colitis (Grade 3): Severe abdominal pain, indications for medical interventions, peritoneal signs<br>Grade 4: Life-threatening, perforation | • Discontinue I-O treatment as per study protocol<br>• 1.0-2.0 mg/kg/day of methylprednisolone IV<br>• or equivalent IV<br>• Add prophylactic antibiotics to prevent<br>• opportunistic infections<br>• Consider lower GI endoscopy | If symptoms improve:<br>• Continue steroid treatment until the symptoms return to Grade 1, then gradually reduce the dose of steroids for at least 1 month<br>If it lasts for > 3-5 days or relapses after improvement:<br>• Add 5 mg/kg infliximab (if there are no contraindications). Note: Do not use infliximab in the case of perforation/sepsis |

If subjects receiving intravenous injection of steroids show continuous clinical improvements, it is allowed, at the start of dose tapering or earlier, to switch to oral administration of corticosteroids (such as prednisone) at equivalent dose. When switching to oral corticosteroids with an equivalent dose, it should be considered that the bioavailability of oral corticosteroids is relatively low

## 2. Management Principles for Pulmonary Adverse Events

**Non-inflammatory causes of disease should be excluded. If it is due to a non-inflammatory cause, a symptomatic treatment should be given while the I-O therapy should be continued. Imaging evaluation and consultations with the respiratory department should be performed.**

| **Grade of Pneumonitis (NCI CTCAE v4)** | **Management** | **Follow-Up** |
|---|---|---|
| **Grade 1**<br>Only radiographic changes | • Consider delaying the I-O treatment<br>• Monitor symptoms every 2-3 days<br>• Consider consulting with respirologist and infectious diseases specialist | • Repeat imaging examinations at least every 3 weeks<br>If it is aggravated:<br>• Provide treatment according to the method for Grade 2 or Grade 3-4 situations |
| **Grade 2**<br>Mild to moderate new symptoms | • Delay I-O treatment as per study protocol<br>• Consult with respirologist and infectious diseases specialist<br>• Monitor symptoms daily and consider hospitalization<br>• 1.0 mg/kg/day of methylprednisolone IV or equivalent PO<br>• Consider bronchoscopy and lung biopsy | • Repeat imaging examinations every 1-3 days<br>If symptoms improve:<br>• When symptoms return to near baseline levels, reduce the dose of steroids for at least 1 month and then resume the I-O treatment as per study protocol while considering prophylactic antibiotics<br>If the symptoms are not improved or aggravated after 2 weeks:<br>• Provide treatment according to the method for Grade 3 or Grade 4 situations |
| **Grade 3-4**<br>Severe new symptoms; new onset/aggravation of hypoxia; life-threatening | • Terminate I-O treatment as per study protocol<br>• Hospitalization<br>• Consult with respirologist and infectious diseases specialist<br>• 2-4 mg/kg/day of methylprednisolone IV or equivalent IV<br>• Add prophylactic antibiotics to prevent opportunistic infections<br>• Consider bronchoscopy and lung biopsy | If symptoms improve to the baseline level:<br>• Gradually reduce the dose of steroids for at least 6 weeks<br>If the symptoms are not improved or aggravated after 48 h:<br>• Add other immunosuppressants (e.g., infliximab, cyclophosphamide, intravenous immunoglobulin or mycophenolate mofetil) |

If subjects receiving intravenous injection of steroids show continuous clinical improvements, it is allowed, at the start of dose tapering or earlier, to switch to oral administration of corticosteroids (such as prednisone) at equivalent dose. When switching to oral corticosteroids with an equivalent dose, it should be considered that the bioavailability of oral corticosteroids is relatively low.

## 3. Management Principles for Hepatic Adverse Events

**Non-inflammatory causes of disease should be excluded. If it is due to a non-inflammatory cause, a symptomatic treatment should be given while the I-O therapy should be continued. Consider imaging examinations to rule out obstruction/tumor progression.**

| Elevation Grade in the Hepatic Function Test | Management | Follow-Up |
|---|---|---|
| **Grade 1**<br>AST or ALT > ULN-3.0 to ≤ 5 × ULN and/or T.bili > ULN-1.5 × ULN | • Continue I-O treatment as per study protocol | • Continue hepatic function monitoring as per study protocol<br>If it is aggravated:<br>• Provide treatment according to the method for Grade 2 or Grade 3/4 situations |
| **Grade 2**<br>AST or ALT > 3.0 to ≤ 5 × ULN and/or T.bili > 1.5 to ≤ 3 × ULN | • Delay I-O treatment as per study protocol<br>• Increase the monitoring frequency to once every 3 days | If it returns to the baseline level:<br>• Resume routine monitoring and resume I-O treatment as per study protocol<br>If elevation persists for > 5-7 days or aggravates:<br>• Intravenous administration of 0.5-1 mg/kg/day methylprednisolone or an equivalent orally administrated dose. If LFT returns to Grade 1 or baseline, reduce the dose of steroids for at least 1 month and consider prophylactic antibiotics to prevent opportunistic infections, and then resume I-O treatment as per study protocol |
| **Grade 3-4**<br>AST or ALT > 5 × ULN and/or T.bili > 3 × ULN | • Terminate I-O treatment*<br>• Increase the monitoring frequency to once every 1-2 days<br>• 1.0-2.0 mg/kg/day of methylprednisolone IV or equivalent IV **<br>• Add prophylactic antibiotics to prevent opportunistic infections<br>• Consult with the gastroenterology department | If it returns to Grade 2:<br>• Reduce the dose of steroids for at least 1 month<br>If there is no improvement within > 3-5 days, or if the symptoms are aggravated or deteriorated:<br>• Add mycophenolate mofetil 1 g bid<br>• If the symptoms are not relieved within 3-5 days, consider other immunosuppressants based on local guidelines |

If subjects receiving intravenous injection of steroids show continuous clinical improvements, it is allowed, at the start of dose tapering or earlier, to switch to oral administration of corticosteroids (such as prednisone) at equivalent dose. When switching to oral corticosteroids with an equivalent dose, it should be considered that the bioavailability of oral corticosteroids is relatively low.

*** If AST/ALT ≤ 8 × ULN and T.bili ≤ 5 × ULN, the I-O treatment can be delayed rather than discontinued.**

** For Grade 4 hepatitis, the recommended starting dose of methylprednisolone intravenous injection is 2 mg/kg/day.

## 4. Management Principles for Endocrine Adverse Events

**Non-inflammatory causes of disease should be excluded. If it is due to a non-inflammatory cause, a symptomatic treatment should be given while the I-O therapy should be continued. Visual field tests, endocrinology consultation and imaging examinations are considered**

**Asymptomatic TSH elevation** →

- Continue I-O treatment as per study protocol
- If TSH < 0.5 × LLN or TSH > 2 × ULN, or TSH results are out of range in 2 consecutive subsequent measurements: According to clinical indications, detect the level of free T4 in subsequent cycles; consider endocrinology consultation

**Symptomatic endocrinopathy** →

- Assess endocrine functions
- Consider pituitary scans

Observation of symptoms and abnormalities in laboratory test results/pituitary scans:
- Delay I-O treatment as per study protocol
- 1-2 mg/kg/day of methylprednisolone IV or equivalent PO
- Start a corresponding hormone therapy

No abnormalities in laboratory test results/pituitary MRI but the symptoms persist:
- Repeat the laboratory test within 1-3 weeks and repeat the pituitary MRI scan 1 month later

→

If there is improvement (with or without hormone replacement):
- Reduce the dose of steroids for at least 1 month and consider prophylactic antibiotics to prevent opportunistic infections
- Resume I-O treatment as per study protocol
- Subjects with adrenal insufficiency may need to continue the use of mineralocorticoid-containing steroids

**Suspected adrenal crisis (e.g., severe dehydration, hypotension, and shock that does not match the severity of the disease)** →

- Delay or terminate I-O treatment as per study protocol
- Exclude sepsis
- Intravenous injection of a stress dose of mineralocorticoid-containing steroids
- Intravenous infusion
- Consult an endocrinologist
- If the adrenal crisis is ruled out, treat the symptomatic endocrinopathy using the methods described above

If subjects receiving intravenous injection of steroids show continuous clinical improvements, it is allowed, at the start of dose tapering or earlier, to switch to oral administration of corticosteroids (such as prednisone) at equivalent dose. When switching to oral corticosteroids with an equivalent dose in the lungs and liver, it should be considered that the bioavailability of oral corticosteroids is relatively low

## 5. Management Principles for Skin Adverse Events

| Rash Grade (NCI CTCAE v4) | Management | Follow-Up |
|---|---|---|
| **Grade 1-2 Coverage ≤ 30% body surface area (BSA)** | • Symptomatic treatment (e.g., antihistamines, topical steroids)<br>• Continue I-O treatment as per study protocol | If symptoms persist for > 1-2 weeks or recur:<br>• Consider skin biopsy<br>• Delay I-O treatment as per study protocol<br>• Consider 0.5-1.0 mg/kg/day of methylprednisolone IV or equivalent PO. Once improved, reduce the dose of steroids for at least 1 month and consider prophylactic antibiotics to prevent opportunistic infections, and resume I-O treatment as per study protocol<br>If it is aggravated:<br>• Provide treatment according to the method for Grade 3-4 situations |
| **Grade 3-4 Coverage > 30% body surface area (BSA); or life-threatening results** | • Delay or terminate I-O treatment as per study protocol<br>• Consider skin biopsy<br>• Consult with the department of dermatology<br>• 1.0-2.0 mg/kg/day of methylprednisolone IV or equivalent IV | If it is improved to Grade 1:<br>• Reduce the dose of steroids for at least 1 month and add prophylactic antibiotics to prevent opportunistic infections<br>• Resume I-O treatment as per study protocol |

If subjects receiving intravenous injection of steroids show continuous clinical improvements, it is allowed, at the start of dose tapering or earlier, to switch to oral administration of corticosteroids (such as prednisone) at equivalent dose. When switching to oral corticosteroids with an equivalent dose in the lungs and liver, it should be considered that the bioavailability of oral corticosteroids is relatively low

（**Weber JS**, Postow M, Lao CD, Schadendorf D. Management of Adverse Events Following Treatment With Anti-Programmed Death-1 Agents. Oncologist. 2016 Jul 8: 2016-0055.

| |
|---|
| Protocol Title: An Open-Label, Multicenter Phase II Clinical Study of Anti-PD-1 Antibody SHR-1210 Combined with Famitinib Malate in Patients with Advanced Urinary System Tumors and Gynecological Tumors |
| Protocol No., Version, and Date: SHR-1210-II-213, V1.1, 30 Sep., 2018 |
| Previous Version: V1.0, 25 Jul., 2018 |
| Revised Version: V1.1, 30 Sep., 2018 |

| Page | Content | Before | After | Reason for Revision |
|---|---|---|---|---|
| Cover Page and Full Text | Cover and header | 1. Version no.: Ethics V1.0;<br>2. Version date: 25 Jul., 2018; | 1. Version no.: Review V1.1;<br>2. Version date: 30 Sep., 2018; | Version upgrade. |
| Version History/ Revision History | Version history/revision history | None | Version 1.1, 30 Sep., 2018, revised wording based on the review comments of the ethics committee | Revised wording based on the review comments of the ethics committee. |
| Sponsor's Signature Page | Sponsor's signature page | 1. Version no.: Ethics V1.0;<br>2. Version date: 25 Jul., 2018; | 1. Version no.: Review V1.1;<br>2. Version date: 30 Sep., 2018; | Version upgrade. |
| P1 | PROTOCOL SYNOPSIS | 1. Version no.: Ethics V1.0;<br>2. Version date: 25 Jul., 2018; | 1. Version no.: Review V1.1;<br>2. Version date: 30 Sep., 2018; | Version upgrade. |
| P29 | 1.2.1. Study rationale | The efficacy for a variety of tumors is evident, and the toxicity information is comprehensive. | The published study results showed that the efficacy of SHR-1210 for a variety of tumors is comparable to that of similar products, and the toxicity information is comprehensive. | Revised wording based on the review comments of the ethics committee. |
| P30 | 1.2.1. Study rationale | The preclinical activity of famitinib is superior to that of the similar drug sunitinib | The preclinical activity of famitinib compared with the similar drug sunitinib: Famitinib showed more significant inhibitory effect on VEGFR2, c-Kit, PDGFR, and Flt1 kinases, as well as more significant anti-tumor effect on renal cancer Caki-1, hepatic cancer Bel-7402, and other xenografts in nude mice. | Revised wording and detailed description based on the review comments of the ethics committee. |

| |
|---|
| Protocol Title: An Open-Label, Multicenter Phase II Clinical Study of Anti-PD-1 Antibody SHR-1210 Combined with Famitinib Malate in Patients with Advanced Urinary System Tumors and Gynecological Tumors |
| Protocol No., Version, and Date: SHR-1210-II-213, V1.1, 30 Sep., 2018 |
| Previous Version: V1.0, 25 Jul., 2018 |
| Revised Version: V1.1, 30 Sep., 2018 |

| Page | Content | Before | After | Reason for Revision |
|---|---|---|---|---|
| P30 | 1.2.1. Study rationale | The clinical characteristics are superior to those of sunitinib: Famitinib showed higher exposure and less accumulation than sunitinib in the human body. In a phase II clinical study of famitinib vs. sunitinib for the treatment of renal cell carcinoma, the efficacy of famitinib at a dose of 25 mg/d was superior to that of sunitinib at a dose of 50 mg/d with 4 weeks on and 2 weeks off in each 6-week cycle (ORR: 36.0% vs. 28.0%); | The clinical characteristics of famitinib compared with sunitinib: Famitinib by oral administration showed higher exposure and less accumulation in the human body. In a phase II clinical study of famitinib vs. sunitinib for the treatment of advanced renal cell carcinoma, the objective response rate (ORR) of famitinib (25 mg/d, continuous oral administration) was 36.0%, while the ORR of the sunitinib control group (50 mg/d, 4 weeks on and 2 weeks off in each 6-week cycle) was 28.0%; | Revised wording based on the review comments of the ethics committee. |

# Revision Record of Protocol SHR-1210-II-213

Jiangsu Hengrui Pharmaceuticals Co., Ltd

| |
|---|
| Protocol Title: An Open-Label, Multicenter Phase II Clinical Study of Anti-PD-1 Antibody SHR-1210 Combined with Famitinib Malate in Patients with Advanced Urinary System Tumors and Gynecological Tumors |
| Protocol No., Version, and Date: SHR-1210-II-213, V2.0, 6 Aug., 2019 |
| Previous Version: V1.1, 30 Sep., 2018 |
| Revised Version: V2.0, 6 Aug., 2019 |

<table>
<tr><th>Page</th><th>Content</th><th>Before</th><th>After</th><th>Reason for Revision</th></tr>
<tr><td>Cover Page and Full Text</td><td>Cover and header (all pages)</td><td>1. Version no.: 1.1;<br>2. Version date: 30 Sep., 2018;</td><td>1. Version no.: 2.0;<br>2. Version date: 6 Aug., 2019;</td><td>Updated the version number and version date.</td></tr>
<tr><td>Version History/ Revision History</td><td>Version history/revision history</td><td>Version History/Revision History
<table>
<tr><th>Document</th><th>Version Date</th><th>Amendment Rationale and Summary of Changes</th></tr>
<tr><td>Version 1.0</td><td>25 Jul., 2018</td><td>Not applicable</td></tr>
<tr><td>Version 1.1</td><td>30 Sep., 2018</td><td>Revised wording and detailed description based on the review comments of the ethics committee</td></tr>
</table></td><td>Version History/Revision History
<table>
<tr><th>Document</th><th>Version Date</th><th>Amendment Rationale and Summary of Changes</th></tr>
<tr><td>Version 1.0</td><td>25 Jul., 2018</td><td>Not applicable</td></tr>
<tr><td>Version 1.1</td><td>30 Sep., 2018</td><td>Revised wording and detailed description based on the review comments of the ethics committee</td></tr>
<tr><td>Version 2.0</td><td>6 Aug., 2019</td><td>1. Revised the requirements for previous systemic treatment in the inclusion criteria for renal cancer, urothelial carcinoma, cervical cancer, endometrial cancer, and ovarian cancer, such that the inclusion criteria are more precise, to further meet the needs of efficacy exploration;<br>2. Modified and added exclusion criteria: patients with CTCAE Grade ≥ 2 hemorrhage within 4 weeks prior to the study treatment; patients with radiation-induced enteritis after receiving pelvic radiotherapy within 12 months prior to the study treatment;<br>3. Based on the results of previous studies and the preliminary safety results of this study, after full discussion with the investigators, it was decided to add the dose level of famitinib at 20 mg combined with SHR-1210 at a fixed dose of 200 mg q2w. Observation of clinically significant toxicity and PK blood</td></tr>
</table></td><td>Updated the version number and version date.</td></tr>
</table>

| |
|---|
| Protocol Title: An Open-Label, Multicenter Phase II Clinical Study of Anti-PD-1 Antibody SHR-1210 Combined with Famitinib Malate in Patients with Advanced Urinary System Tumors and Gynecological Tumors |
| Protocol No., Version, and Date: SHR-1210-II-213, V2.0, 6 Aug., 2019 |
| Previous Version: V1.1, 30 Sep., 2018 |
| Revised Version: V2.0, 6 Aug., 2019 |

| Page | Content | Before | After | Reason for Revision |
|---|---|---|---|---|
| | | | sampling will be first performed in 12 subjects at this dose level. Also, the corresponding safety and efficacy visit points in the Schedule of Activities were revised;<br>4. Added some biomarker detection parameters in the exploratory endpoints: detection of MMR or MSI-H for ovarian cancer and detection of proportion of abnormal FGFR2/3 for urothelial carcinoma;<br>5. Added "B-mode ultrasonography of lower extremity veins" in the examinations of screening period and the end of treatment;<br>6. Revised the wording of the statistics part, and changed the description of exploratory analysis according to the revisions made;<br>7. Revised wording and logic. | |
| Sponsor's Signature Page | Sponsor's signature page | 1. Version no.: 1.1;<br>2. Version date: 30 Sep., 2018; | 1. Version no.: 2.0;<br>2. Version date: 6 Aug., 2019; | Updated the version number and version date. |
| P1 | Protocol synopsis | 1. Version no.: 1.1;<br>2. Version date: 30 Sep., 2018; | 1. Version no.: 2.0;<br>2. Version date: 6 Aug., 2019; | Updated the version number and version date. |
| P2-P3<br>P44 | Study endpoints | Exploratory endpoints<br>The proportion of PD-L1-positive cells in tumor tissue and ~~circulating tumor cells~~, ~~tumor mutation burden (TMB), and mismatch repair (MMR) status in tumor tissue and/or peripheral blood~~ (for endometrial cancer only) will be | Exploratory endpoints<br>The proportion of PD-L1-positive cells in tumor tissue, **proportion of subjects with dMMR or MSI-H** (for endometrial cancer and **ovarian cancer** only), and **proportion of abnormal FGFR2/3 gene (for urothelial carcinoma only)** will be evaluated | Selected more appropriate biomarkers for exploration to provide scientific rationale for further studies. |

| |
|---|
| Protocol Title: An Open-Label, Multicenter Phase II Clinical Study of Anti-PD-1 Antibody SHR-1210 Combined with Famitinib Malate in Patients with Advanced Urinary System Tumors and Gynecological Tumors |
| Protocol No., Version, and Date: SHR-1210-II-213, V2.0, 6 Aug., 2019 |
| Previous Version: V1.1, 30 Sep., 2018 |
| Revised Version: V2.0, 6 Aug., 2019 |

| Page | Content | Before | After | Reason for Revision |
|---|---|---|---|---|
| | | evaluated to explore the relationship of PD-L1 expression and/or other biomarkers (~~such as TMB at baseline~~) with efficacy. | to explore the relationship of PD-L1 expression and/or other biomarkers with efficacy **(such as ORR/PFS)**. | |
| P4<br>P45 | Study design | The first 12 subjects enrolled in the study will also undergo blood sampling for PK analysis to explore the blood concentrations and PK parameters of famitinib and SHR-1210 in combination therapy | **If famitinib 20 mg, qd and SHR-1210 200 mg, q3w, iv combination therapy is demonstrated well-tolerated, a combination of famitinib 20 mg, qd and SHR-1210 200 mg, q2w, iv will be given with 4-week cycles for observation. Twelve subjects will be enrolled. If clinically significant toxicity is observed in ≥ 4 out of the 12 subjects in Cycle 1, or > 30% subjects requires a reduced famitinib dose, the combination of famitinib 20 mg, qd and SHR-1210 q2w will be deemed to be poorly tolerated.**<br><br>The first 12 subjects enrolled **at each dose level** in the study will also undergo blood sampling for PK analysis to explore the blood concentrations and PK parameters of famitinib and SHR-1210 in combination therapy **(for the first 12 subjects in the SHR-1210 200 mg, q2w group, only blood sampling for the analysis of SHR-1210 blood concentration and PK is required).** | On the premise of tolerance to SHR-1210 q3w combined with famitinib, the tolerability of SHR-1210 q2w combined with famitinib will be further evaluated to provide scientific rationales for the selection of doses in subsequent clinical trials. |
| P4<br>P45 | Study design, study rationale | After enrollment in the study, subjects should undergo safety follow-up prior to SHR-1210 administration on D1 in each treatment cycle. Imaging assessments should be conducted once | After enrollment in the study, subjects who **receive famitinib 20 mg, qd combined with SHR-1210 q3w** should undergo safety follow-up prior to SHR-1210 administration on D1 in each treatment cycle. Imaging assessments should be conducted | Revised based on the added tolerability study of SHR-1210 q2w combined with famitinib. |

| |
|---|
| Protocol Title: An Open-Label, Multicenter Phase II Clinical Study of Anti-PD-1 Antibody SHR-1210 Combined with Famitinib Malate in Patients with Advanced Urinary System Tumors and Gynecological Tumors |
| Protocol No., Version, and Date: SHR-1210-II-213, V2.0, 6 Aug., 2019 |
| Previous Version: V1.1, 30 Sep., 2018 |
| Revised Version: V2.0, 6 Aug., 2019 |

| Page | Content | Before | After | Reason for Revision |
|---|---|---|---|---|
| | | every 3 cycles (9 weeks) to evaluate efficacy from the start of treatment until radiographic progression, commencement of new anti-tumor treatment, withdrawal of informed consent, loss to follow-up, or death. | once every 3 cycles (9 weeks) to evaluate efficacy from the start of treatment until radiographic progression, commencement of new anti-tumor treatment, withdrawal of informed consent, loss to follow-up, or death.<br>**After enrollment in the study, subjects who receive famitinib 20 mg, qd combined with SHR-1210 q2w should undergo safety follow-up prior to SHR-1210 administration on D1 and D15 in each treatment cycle. Imaging assessments should be conducted once every 2 cycles (8 weeks) to evaluate efficacy from the start of treatment until radiographic progression, commencement of new anti-tumor treatment, withdrawal of informed consent, loss to follow-up, or death.** | |
| P4-5 | Study design, study rationale | Tumor tissue samples, in sections, paraffin blocks, ~~or biopsy tissue blocks~~, after the last treatment prior to this study, ~~as well as whole blood~~ for biomarker detection will be acquired/collected from subjects at screening. Tests include but are not limited to the following: PD-L1 expression ~~level and the proportion of positive cells, TMB, and MMR (for endometrial cancer only).~~ | Tumor tissue samples, in sections or paraffin blocks, after the last treatment prior to this study for biomarker detection will be acquired/collected from subjects at screening. Tests include but are not limited to the following: **proportion of** PD-L1-**positive cells in tumor tissue, MMR (for endometrial cancer and ovarian cancer only), and FGFR2/3 mutation (for urothelial carcinoma only). The proportion of PD-L1-positive cells in tumor tissue, proportion of subjects with dMMR or MSI-H (for endometrial cancer and ovarian cancer only), and proportion of abnormal FGFR2/3 (for urothelial carcinoma only) will be** | Revised based on adjusted biomarker assays. |

| Protocol Title: An Open-Label, Multicenter Phase II Clinical Study of Anti-PD-1 Antibody SHR-1210 Combined with Famitinib Malate in Patients with Advanced Urinary System Tumors and Gynecological Tumors |
|---|
| Protocol No., Version, and Date: SHR-1210-II-213, V2.0, 6 Aug., 2019 |
| Previous Version: V1.1, 30 Sep., 2018 |
| Revised Version: V2.0, 6 Aug., 2019 |

| Page | Content | Before | After | Reason for Revision |
|---|---|---|---|---|
| | | | **evaluated to explore the relationship of PD-L1 expression and/or other biomarkers with efficacy (such as ORR/PFS).** | |
| P5 | Dosing Regimen | SHR-1210: administered via intravenous infusion (premedication not required) at a fixed dose of 200 mg within 30 min (not less than 20 min, not more than 60 min), once every 3 weeks; each cycle contains 3 weeks and the longest dosing period is 2 years.<br>Famitinib malate capsules: administered orally once a day before or after a meal (recommended at a fixed time: within 0.5 h after a meal) at a dose of 20 mg or 15 mg. The drug should be administered continuously in cycles of 3 weeks.<br>Subjects in the PK study should take famitinib orally before breakfast on the day of blood sampling for PK study. | SHR-1210: administered via intravenous infusion (premedication not required) at a fixed dose of 200 mg within 30 min (not less than 20 min, not more than 60 min), once every 3 weeks; each cycle contains 3 weeks; **for subjects at the "dose level of SHR-1210 q2w", the drug will be administered once every 2 weeks in cycles of 4 weeks;** the longest dosing period is 2 years.<br>Famitinib malate capsules: administered orally once a day before or after a meal (recommended at a fixed time: within 0.5 h after a meal) at a dose of 20 mg or 15 mg. The drug should be administered continuously in cycles of 3 weeks. **For subjects at the "dose level of SHR-1210 q2w", famitinib capsules will be administered in cycles of 4 weeks.**<br>Subjects in the PK study should take famitinib orally before breakfast on the day of blood sampling for PK study. | Revised based on the added tolerability study of SHR-1210 q2w combined with famitinib. |
| P5<br>P46 | Definition of clinically significant toxicity | The definition is limited to events observed in the first 12 enrolled subjects within the first 2 cycles that are deemed related to the investigational products by the investigators and meet the following:<br>2. Grade ≥ 3 non-hematologic toxicity (except | This definition is limited to events observed in the first 12 enrolled subjects within the first 2 cycles **(within the first cycle for the first 12 subjects enrolled at the "dose level of SHR-1210 q2w")** that are deemed related to the investigational products by the investigators and meet the following: | Revised based on the added tolerability study of SHR-1210 q2w combined with famitinib. |

| |
|---|
| Protocol Title: An Open-Label, Multicenter Phase II Clinical Study of Anti-PD-1 Antibody SHR-1210 Combined with Famitinib Malate in Patients with Advanced Urinary System Tumors and Gynecological Tumors |
| Protocol No., Version, and Date: SHR-1210-II-213, V2.0, 6 Aug., 2019 |
| Previous Version: V1.1, 30 Sep., 2018 |
| Revised Version: V2.0, 6 Aug., 2019 |

| Page | Content | Before | After | Reason for Revision |
|---|---|---|---|---|
| | | for laboratory abnormalities), Grade 3 hypertension, rash, diarrhea, nausea, and vomiting that cannot be effectively controlled after symptomatic treatment; | 2. Grade ≥ 3 non-**hematologic** toxicity (except for laboratory abnormalities), Grade 3 hypertension, rash, diarrhea, nausea, and vomiting that cannot be effectively controlled after symptomatic treatment; | |
| P6<br>P46 | Definition of clinically significant toxicity | 4. Related toxicity resulting in failure to complete 2 administrations of SHR-1210 within the first two cycles or normal SHR-1210 administration in Cycle 3 (treatment delay > ~~10 days~~);<br><br>5. Related toxicity resulting in famitinib interruption for > 14 days.<br>If any of the first 12 subjects fails to complete treatment observation for two cycles due to non-drug-related toxicity, the subject must be replaced. | 4. Related toxicity resulting in failure to complete 2 administrations of SHR-1210 within the first two cycles or normal SHR-1210 administration in Cycle 3 (treatment delay **> 7 days**). **Note: Related toxicity resulting in failure to complete 2 administrations of SHR-1210 in Cycle 1 or normal SHR-1210 administration in Cycle 2 (treatment delay > 3 days) for subjects at the "dose level of SHR-1210 q2w"**<br>5. Related toxicity resulting in famitinib interruption for > 14 days.<br>If any of the first 12 subjects fails to complete treatment observation for two cycles due to non-drug-related toxicity, the subject must be replaced. **(If any of the first 12 subjects at the "dose level of SHR-1210 q2w" fails to complete treatment observation for the first cycle due to non-drug-related toxicity, the subject must be replaced.)** | Revised based on the added tolerability study of SHR-1210 q2w combined with famitinib. |

| |
|---|
| Protocol Title: An Open-Label, Multicenter Phase II Clinical Study of Anti-PD-1 Antibody SHR-1210 Combined with Famitinib Malate in Patients with Advanced Urinary System Tumors and Gynecological Tumors |
| Protocol No., Version, and Date: SHR-1210-II-213, V2.0, 6 Aug., 2019 |
| Previous Version: V1.1, 30 Sep., 2018 |
| Revised Version: V2.0, 6 Aug., 2019 |

| Page | Content | Before | After | Reason for Revision |
|---|---|---|---|---|
| P6 | Blood Sampling for PK and ADA Analysis | For SHR-1210 PK analysis, blood samples will be collected within 30 ~~min~~ before administration and within 5 min after administration (including flushing) of the first SHR-1210 dose; within 30 min before administration and within 5 min after administration (including flushing) of SHR-1210 on administration days of Cycles 2, 3, and 4; within 30 min pre-administration every 4 cycles (12 weeks) thereafter; at the ~~end of study~~ (the documented time of last SHR-1210 administration prior to the end of study); at 30 days after the end of treatment. (If subject completes the treatment before scheduled blood sampling is completed, the analysis should be based on actual completed sampling).<br>ADA blood samples will be simultaneously collected along with PK samples before each administration, at the end of treatment, and at 30 days after the end of treatment. | For SHR-1210 PK analysis, blood samples will be collected within 30 **min** before administration and within 5 min after administration (including flushing) of the first SHR-1210 dose; within 30 min before administration and within 5 min after administration (including flushing) of SHR-1210 on administration days of Cycles 2, 3, and 4; within 30 min pre-administration every 4 cycles (12 weeks) thereafter; **at the end of treatment or upon withdrawal from study** (the documented time of last SHR-1210 administration prior to the end of study); at 30 days after the end of **SHR-1210** treatment. (If subject completes the treatment before scheduled blood sampling is completed, the analysis should be based on actual completed sampling).<br>ADA blood samples will be simultaneously collected along with PK samples before each administration, at the end of **SHR-1210** treatment, and at 30 days after the end of **SHR-1210** treatment. | Revised wording to improve expressions. |
| P7<br>P50 | Inclusion criteria | 2. Male or female aged ≥ 18 years; | 2. Male or female aged between 18**-70 years (for endometrial cancer cohort: 18-75 years old)**; | Revised based on the consensus reached by the investigators at the investigators' meeting. |
| P7<br>P50 | Inclusion criteria | 3.<br>- Renal cell carcinoma: Histologically or cytologically confirmed advanced clear cell renal cell carcinoma (in the case of mixed | - Renal cell carcinoma: Histologically or cytologically confirmed advanced clear cell renal cell carcinoma (in the case of mixed tumors, predominant clear cell renal cell carcinoma is | Clarified the inclusion criteria to further meet the needs of efficacy exploration. |

| |
|---|
| Protocol Title: An Open-Label, Multicenter Phase II Clinical Study of Anti-PD-1 Antibody SHR-1210 Combined with Famitinib Malate in Patients with Advanced Urinary System Tumors and Gynecological Tumors |
| Protocol No., Version, and Date: SHR-1210-II-213, V2.0, 6 Aug., 2019 |
| Previous Version: V1.1, 30 Sep., 2018 |
| Revised Version: V2.0, 6 Aug., 2019 |

| Page | Content | Before | After | Reason for Revision |
|---|---|---|---|---|
| | | tumors, predominant clear cell renal cell carcinoma is required); ~~those with the primary tumor being surgically removed who have previously received interleukin-2 and/or targeted anti-angiogenic therapy and have failed the treatment; those who are not willing to accept or are unable to afford targeted anti-angiogenic therapy can also be considered for enrollment;~~ | required) **that has been previously treated with at most one targeted anti-angiogenic drug and failed the treatment (if any);** | |
| P7-P8<br>P50-51 | Inclusion criteria | - Cervical cancer: Histologically or cytologically ~~confirmed advanced~~ squamous cell cervical cancer; have ~~failed~~ the first-line or above systemic treatment;<br>- Recurrent ovarian cancer: Histopathologically confirmed recurrent epithelial ovarian cancer, fallopian tube cancer, or primary peritoneal cancer that has been treated with platinum-based therapy and recurred/progressed during or within 6 months after the last platinum-based therapy (completing 4 or more treatment cycles);<br>- Endometrial cancer: Histopathologically confirmed endometrial cancer ~~that has previously been treated with at least a platinum-based regimen and has relapsed or progressed after or during treatment.~~ | - Cervical cancer: Histologically or cytologically confirmed squamous cell carcinoma of the cervix; for recurrent/metastatic cervical cancer that has been treated with 1 or 2 lines of previous systemic treatment **(except radiation-enhanced chemotherapy), recurrence or progression during or after previous treatment** is required;<br>**Note: Neoadjuvant or adjuvant therapies (except radiation-enhanced chemotherapy), with recurrence and progression within 1 year after first-line standard surgery, or within 6 months after radiotherapy, are included in the first-line systemic treatment.**<br>- Recurrent ovarian cancer: Histopathologically confirmed recurrent epithelial ovarian cancer, fallopian tube cancer, or primary peritoneal cancer that has been treated with platinum-based therapy and recurred/progressed during or within 6 months after the last platinum-based therapy (completing 4 or more treatment cycles); | Clarified the inclusion criteria to further meet the needs of efficacy exploration. |

| |
|---|
| Protocol Title: An Open-Label, Multicenter Phase II Clinical Study of Anti-PD-1 Antibody SHR-1210 Combined with Famitinib Malate in Patients with Advanced Urinary System Tumors and Gynecological Tumors |
| Protocol No., Version, and Date: SHR-1210-II-213, V2.0, 6 Aug., 2019 |
| Previous Version: V1.1, 30 Sep., 2018 |
| Revised Version: V2.0, 6 Aug., 2019 |

| Page | Content | Before | After | Reason for Revision |
|---|---|---|---|---|
| | | | **Note: No more than 1 non-platinum-based therapy between the last 2 platinum-based therapies; no other anti-tumor treatments except endocrine therapy, PARP inhibitor maintenance therapy, or traditional Chinese medicine and modern Chinese medicinal preparations after the last platinum-based therapy.**<br>- Endometrial cancer: Histopathologically confirmed endometrial cancer **(excluding carcinosarcoma) that recurred/metastasized after previous treatment; for recurrent/metastatic cancer that has been treated with 1 or 2 lines of previous systemic treatment (except radiation-enhanced chemotherapy), recurrence or progression during or after previous treatment is required.** | |
| P8<br>P51 | Inclusion criteria | 8. Major organ functions must meet the following requirements (No blood components or growth factor corrective therapy is allowed within 14 days prior to the start of study treatment):<br><br>• Serum creatinine ≤ ~~1.5~~ × ULN; | 8. Major organ functions must meet the following requirements (No blood components or growth factor corrective therapy is allowed within 14 days prior to the start of study treatment):<br><br>• Serum creatinine ≤ **1.25** × ULN; | To ensure the medication safety of subjects. |
| P9<br>P51 | Inclusion criteria | 9. Female patients of childbearing potential or female patients who are not sterilized by surgical operations are required to take two medically approved contraceptive measures (such as | 9. Female patients of childbearing potential or female patients who are not sterilized by surgical operations are required to take two medically approved contraceptive measures (such as | Revised wording and logic. |

| |
|---|
| Protocol Title: An Open-Label, Multicenter Phase II Clinical Study of Anti-PD-1 Antibody SHR-1210 Combined with Famitinib Malate in Patients with Advanced Urinary System Tumors and Gynecological Tumors |
| Protocol No., Version, and Date: SHR-1210-II-213, V2.0, 6 Aug., 2019 |
| Previous Version: V1.1, 30 Sep., 2018 |
| Revised Version: V2.0, 6 Aug., 2019 |

| Page | Content | Before | After | Reason for Revision |
|---|---|---|---|---|
| | | intrauterine device, oral contraceptive, or condom) during the study treatment period and within 3 months after the end of the study treatment; female patients of childbearing potential who are not surgically sterilized must have a negative serum ~~or urine HCG~~ test result within ~~7 days prior to enrollment~~, and must not be on breast-feeding; male patients with partners of childbearing potential should take effective contraceptive measures during the study and within 3 months after ~~the last dose of SHR-1210~~. | intrauterine device, oral contraceptive, or condom) during the study treatment period and within 3 months after the end of the study treatment; female patients of childbearing potential who are not surgically sterilized must have a negative serum HCG test result within **72 h prior to the first dose**, and must not be on breast-feeding; male patients with partners of childbearing potential should take **two** effective contraceptive measures during the study and within 3 months after **the end of study treatment**. | |
| P9<br>P52 | Exclusion criteria | 2. Currently using immunosuppressants, or systemic hormonal therapy for immunosuppression (> 10 mg/day of prednisone or an equivalent dose of other therapeutic hormones) within 2 weeks prior to ~~enrollment~~; | 2. Currently using immunosuppressants, or systemic hormonal therapy for immunosuppression (> 10 mg/day of prednisone or an equivalent dose of other therapeutic hormones) within 2 weeks prior to **the first dose**; | Revised wording to improve expressions. |
| P10<br>P52 | Exclusion criteria | 8. ~~History of bleeding,~~ and any Grade ≥ ~~3~~ bleeding as per CTCAE ~~4.0~~ within 4 weeks before screening; | 8. Any Grade ≥ **2** bleeding as per CTCAE **4.03** within 4 weeks before the **first dose**; | Revised wording and logic to ensure the medication safety of subjects. |
| P10<br>P52 | Exclusion criteria | 10. Events of arterial/venous thrombosis within 6 months ~~prior to randomization~~, such as cerebrovascular accidents (including transient ischemic attacks, cerebral hemorrhage, and brain infarction), deep vein thrombosis, and pulmonary embolism; | 10. Events of arterial/venous thrombosis within 6 months **prior to the first dose**, such as cerebrovascular accidents (including transient ischemic attacks, cerebral hemorrhage, and brain infarction), deep vein thrombosis, and pulmonary embolism; | Revised logic. |

| |
|---|
| Protocol Title: An Open-Label, Multicenter Phase II Clinical Study of Anti-PD-1 Antibody SHR-1210 Combined with Famitinib Malate in Patients with Advanced Urinary System Tumors and Gynecological Tumors |
| Protocol No., Version, and Date: SHR-1210-II-213, V2.0, 6 Aug., 2019 |
| Previous Version: V1.1, 30 Sep., 2018 |
| Revised Version: V2.0, 6 Aug., 2019 |

| Page | Content | Before | After | Reason for Revision |
|---|---|---|---|---|
| P10-11<br>P53 | Exclusion criteria | ~~14~~. Active infection, unexplained fever ≥ 38.5 °C within 7 days prior to the study treatment, or baseline white blood cell count > 15 × $10^9$/L;<br>~~15~~. Known history or evidence of interstitial lung disease or non-infectious pneumonitis that has been treated with corticosteroids; or conditions that may interfere with the testing or management of suspected treatment-related pulmonary toxicity;<br>~~16.~~ Congenital or acquired immunodeficiency (such as HIV positive);<br>~~17. HBV DNA > 500 IU/mL, HCV RNA > $10^3$-copies/mL, and HBsAg positive and anti-HCV antibody positive;~~<br>~~18.~~ Other malignancies currently or within the past 5 years (except for cured basal cell carcinoma and cervical cancer *in situ*; for recurrent ovarian cancer previously accompanied by breast cancer, patients with no breast cancer recurrence for > 3 after radical mastectomy can be included);<br>~~19~~. Prior treatment with anti-PD-1/PD-L1 antibodies or famitinib, ~~or known allergies to antibodies~~;<br>~~20.~~ Have received live vaccines in ~~less than~~ 4 weeks before ~~the study treatment~~ or may possibly receive live vaccines during the study; | **14. Radiation-induced enteritis after receiving pelvic radiotherapy within 12 months prior to the study treatment;**<br>**15.** Active infection, unexplained fever ≥ 38.5 °C within 7 days prior to the **first** study dose, or baseline white blood cell count > 15 × $10^9$/L;<br>**16.** Known history or evidence of interstitial lung disease or non-infectious pneumonitis that has been treated with corticosteroids; or conditions that may interfere with the testing or management of suspected treatment-related pulmonary toxicity;<br>**17.** Congenital or acquired immunodeficiency (such as HIV positive);<br>**18. Active hepatitis (hepatitis B: positive HBsAg and HBV DNA ≥ 500 IU/ mL; hepatitis C: positive HCV antibody and HCV virus copy number > upper limit of normal);**<br>**19.** Other malignancies currently or within the past 5 years (except for cured basal cell carcinoma and cervical cancer *in situ*; for recurrent ovarian cancer previously accompanied by breast cancer, patients with no breast cancer recurrence for > 3 **years** after radical mastectomy can be included);<br>**20**. Prior treatment with anti-PD-1/PD-L1 antibodies or famitinib;<br>**21.** Have received live vaccines within 4 weeks | Revised wording and logic to ensure the medication safety of subjects. |

| |
|---|
| Protocol Title: An Open-Label, Multicenter Phase II Clinical Study of Anti-PD-1 Antibody SHR-1210 Combined with Famitinib Malate in Patients with Advanced Urinary System Tumors and Gynecological Tumors |
| Protocol No., Version, and Date: SHR-1210-II-213, V2.0, 6 Aug., 2019 |
| Previous Version: V1.1, 30 Sep., 2018 |
| Revised Version: V2.0, 6 Aug., 2019 |

| Page | Content | Before | After | Reason for Revision |
|---|---|---|---|---|
| | | ~~21.~~ Other potential factors that may affect the study results or result in premature discontinuation as determined by the investigators, such as alcoholism, drug abuse, other serious diseases (including mental illness) requiring concomitant treatment, serious laboratory abnormalities, or family or social factors that could affect the safety of the patients. | before the **first** dose or may possibly receive live vaccines during the study;<br>**22.** Other potential factors that may affect the study results or result in premature discontinuation as determined by the investigators, such as alcoholism, drug abuse, other serious diseases (including mental illness) requiring concomitant treatment, serious laboratory abnormalities, or family or social factors that could affect the safety of the patients. | |
| P11<br>P54 | Criteria for discontinuation | A subject must ~~withdraw from~~/discontinue the treatment when any of the following conditions occurs:<br>1. Subject requests for discontinuation or withdraws informed consent; | A subject must discontinue the treatment when any of the following conditions occurs:<br>1. Subject requests for discontinuation or withdraws the **ICF**; | Revised wording to improve expressions. |
| P12<br>P55 | Study withdrawal criteria | Reasons for withdrawal may include:<br>1. Subject withdraws informed consent; | Reasons for withdrawal may include:<br>1. Subject withdraws the **ICF and refuses further follow-ups**; | Revised wording to improve expressions. |
| P14 | Exploratory analysis | Exploratory analysis<br>The proportion of PD-L1-positive cells in tumor tissue and ~~circulating tumor cells~~, ~~tumor mutation burden (TMB), and mismatch repair (MMR) status in tumor tissue and/or peripheral blood~~ (for endometrial cancer only) will be evaluated to explore the relationship of PD-L1 expression and/or other biomarkers (~~such as TMB at baseline~~) with efficacy. | Exploratory analysis<br>The proportion of PD-L1-positive cells in tumor tissue, **proportion of subjects with dMMR or MSI-H** (for endometrial cancer and **ovarian cancer** only), and **proportion of abnormal FGFR2/3 gene (for urothelial carcinoma only)** will be evaluated to explore the relationship of PD-L1 expression and/or other biomarkers with efficacy **(such as ORR/PFS)**. | Selected more appropriate biomarkers for exploration to provide scientific rationale for further studies. |

| |
|---|
| Protocol Title: An Open-Label, Multicenter Phase II Clinical Study of Anti-PD-1 Antibody SHR-1210 Combined with Famitinib Malate in Patients with Advanced Urinary System Tumors and Gynecological Tumors |
| Protocol No., Version, and Date: SHR-1210-II-213, V2.0, 6 Aug., 2019 |
| Previous Version: V1.1, 30 Sep., 2018 |
| Revised Version: V2.0, 6 Aug., 2019 |

| Page | Content | Before | After | Reason for Revision |
|---|---|---|---|---|
| P18<br>P67 | Notes | Notes: All examinations and trial procedures are carried out according to the time specified in the Schedule of Activities and are not affected by the length of treatment interruption. However, occasional changes ~~within~~ the allowable window are permitted due to holidays or other management reasons. | Notes: All examinations and trial procedures are carried out according to the time specified in the Schedule of Activities and are not affected by the length of treatment interruption. However, occasional changes **outside** the allowable window are permitted due to holidays or other management reasons. | Revised logic. |
| P18-19 | Notes<br>[3] [4] [5] [6] [7] [8] [10] [14] [15] [16] [17] | At the ~~end of study~~ and 30 days after treatment. | **At the end of treatment or upon withdrawal from study,** and at 30 days after treatment completion. | Revised wording to improve expressions. |
| P18<br>P69<br>P74<br>P77 | Notes<br>6.2.3 - [Urinalysis] | [5] Urinalysis: urine protein, urine glucose, urine occult blood, urine red blood cells, and urine white blood cells. ~~If semi-quantitative tests from 2 consecutive follow-ups show protein of 2+, a quantitative 24-h urine protein test is required~~, and if semi-quantitative test shows protein of ~~≥ 2+~~, a quantitative 24-h urine protein test should be performed. The test should be performed within 7 days prior to enrollment, on Day 7 of Cycle 1, on Day 1 of subsequent cycles, at ~~the end of treatment~~, and at 30 days after the end of treatment. | [5] Urinalysis: urine protein, urine glucose, urine occult blood, urine red blood cells, and urine white blood cells. **During the screening period**, if semi-quantitative test shows **urine** protein of **≥ 2+**, a quantitative 24-h urine protein test is required. **Subsequently, if semi-quantitative tests from 2 consecutive follow-ups show urine protein of 2+, a quantitative 24-h urine protein test is required, and if semi-quantitative test shows protein of > 2+, a quantitative 24-h urine protein test should be performed.** The test should be performed within 7 days prior to enrollment, on Day 7 of Cycle 1, on Day 1 of subsequent cycles, **at the end of treatment or upon withdrawal from study**, and at 30 days after the end of treatment. | Revised wording and logic. |

| |
|---|
| Protocol Title: An Open-Label, Multicenter Phase II Clinical Study of Anti-PD-1 Antibody SHR-1210 Combined with Famitinib Malate in Patients with Advanced Urinary System Tumors and Gynecological Tumors |
| Protocol No., Version, and Date: SHR-1210-II-213, V2.0, 6 Aug., 2019 |
| Previous Version: V1.1, 30 Sep., 2018 |
| Revised Version: V2.0, 6 Aug., 2019 |

| Page | Content | Before | After | Reason for Revision |
|---|---|---|---|---|
| P18 | Notes | [9] Hepatitis B and hepatitis C tests: Subjects with abnormal HBsAg results should undergo quantitative test of HBV DNA. Subjects with positive anti-~~HCV~~ antibodies must be tested for HCV titer (HCV RNA). | [9] Hepatitis B and hepatitis C tests: Subjects with abnormal HBsAg results should undergo quantitative test of HBV DNA. Subjects with positive anti-HCV antibodies must be tested for HCV titer (HCV RNA). | Revised wording. |
| P18 | Notes | [10] Pregnancy test: A blood pregnancy test should be performed on women of ~~childbearing potential~~ within 72 h prior to the first dose. Additional tests may be performed to rule out pregnancy if indicated. By considering the subjects' condition, the test can be performed as needed and ~~at the end of study~~. | [10] Pregnancy test: A blood pregnancy test should be performed on women **of childbearing potential** within 72 h prior to the first dose. Additional tests may be performed to rule out pregnancy if indicated. By considering the subjects' condition, the test can be performed as needed and **at the end of treatment or upon withdrawal from study**. | Revised wording. |
| P18 | Notes | [12] Pituitary adrenal axis test: including adrenocorticotropic hormone (ACTH), cortisol, and follicle stimulating hormone. The test should be performed within 21 days before administration ~~in the screening period~~. | [12] Pituitary adrenal axis test: including adrenocorticotropic hormone (ACTH), cortisol, and follicle stimulating hormone. The test should be performed within 21 days before administration. | Revised logic. |
| P18 | Notes | [13] Adverse events: AEs should be recorded from the signing of the ICF until at least 30 days after the last dose and should be ~~followed up until the AEs are resolved or stabilized~~. SAEs and irAEs observed within 90 days after the last dose of SHR-1210 should be followed up. ~~If subjects start new anti-tumor treatment, they should be followed up until they start the tumor treatment.~~ | [13] Adverse events: AEs should be recorded from the signing of informed consent until at least 30 days after the last dose. SAEs and irAEs within 90 days after the last dose of SHR-1210 must be **collected** and followed up. **All AEs must be followed up until the event resolves, returns to the baseline level, reaches a stable disease, or returns to Grade ≤ 1, loss to follow-up or death. See Section 8.2.6 for details.** | Revised wording to improve expressions. |

| Protocol Title: An Open-Label, Multicenter Phase II Clinical Study of Anti-PD-1 Antibody SHR-1210 Combined with Famitinib Malate in Patients with Advanced Urinary System Tumors and Gynecological Tumors |
|---|
| Protocol No., Version, and Date: SHR-1210-II-213, V2.0, 6 Aug., 2019 |
| Previous Version: V1.1, 30 Sep., 2018 |
| Revised Version: V2.0, 6 Aug., 2019 |

| Page | Content | Before | After | Reason for Revision |
|---|---|---|---|---|
| P19<br>P69 | Notes | [16] 12-lead ECG: The examination should be performed within 7 days prior to enrollment, on Day 7 of Cycle 1, on Day 1 of subsequent cycles, and at ~~the end of study~~. ~~If the ECG is abnormal (with clinical significance), another two examinations must be performed for confirmation.~~ | [16] 12-lead ECG: The examination should be performed within 7 days prior to enrollment, on Day 7 of Cycle 1, on Day 1 of subsequent cycles, and **at the end of treatment or upon withdrawal from study**. **ECG examination within 7 days before the first dose in the screening period will be performed for 3 consecutive times at an interval of about 5 min. The average value of the 3 QTc results is taken. The average value of the 3 QTc results of the enrolled subject must meet the inclusion and exclusion criteria. If the ECG is abnormal (with clinical significance) during the study, another two examinations must be performed.** | Revised logic to make it consistent with the main text. |
| P19 | Notes | [18] Blood pressure monitoring: The blood pressure of **patients** will be measured by the investigators or study nurse in the screening period. At each blood pressure measurement, smoking and coffee are prohibited within 30 min before measurement, and patients should rest for at least 10 min. The sitting position will be taken at measurement by placing the elbow at the same level as the heart. Each blood pressure measurement should be taken on the same side of the body. Blood pressure will be measured by **patients** themselves during the study and recorded in their diary cards. Blood pressure should be measured at least 3 times a week in the first 2 cycles. If blood pressure is abnormal, | [18] Blood pressure monitoring: The blood pressure of patients will be measured by the investigators or study nurse in the screening period. At each blood pressure measurement, smoking and coffee are prohibited within 30 min before measurement, and patients should rest for at least 10 min. The sitting position will be taken at measurement by placing the elbow at the same level as the heart. Each blood pressure measurement should be taken on the same side of the body. Blood pressure will be measured by **subjects** themselves during the study and recorded in their diary cards. Blood pressure should be measured at least 3 times a week in the first 2 cycles. If blood pressure is abnormal, the measurement should be carried out every day; if | Revised wording |

| |
|---|
| Protocol Title: An Open-Label, Multicenter Phase II Clinical Study of Anti-PD-1 Antibody SHR-1210 Combined with Famitinib Malate in Patients with Advanced Urinary System Tumors and Gynecological Tumors |
| Protocol No., Version, and Date: SHR-1210-II-213, V2.0, 6 Aug., 2019 |
| Previous Version: V1.1, 30 Sep., 2018 |
| Revised Version: V2.0, 6 Aug., 2019 |

| Page | Content | Before | After | Reason for Revision |
|---|---|---|---|---|
| | | the measurement should be carried out every day; if blood pressure is normal, the measurement should be carried out at least twice a week after 2 cycles. In addition, blood pressure will be measured by the investigators or study nurse at each follow-up. | blood pressure is normal, the **measurement** should be carried out at least twice a week after 2 cycles. In addition, blood pressure will be measured by the investigators or study nurse at each follow-up. | |
| P16<br>P19<br>P70<br>P72 | Schedule of activities<br>Notes<br>Main text of protocol<br>Main text of protocol | None | [19] B-mode ultrasonography of lower extremity veins: B-mode ultrasonography of the deep veins of both lower extremities should be performed within 7 days prior to enrollment and at the end of treatment or upon withdrawal from study. The examination may be performed as necessary based on the investigators' clinical judgment. | Made revisions corresponding to the added exclusion criteria. |
| P17<br>P19-20 | Schedule of activities<br>Notes (numbering adjustment) | [19] [20] [21] [22] [23] [24] [25] [26] [27] | [20] [21] [22] [23] [24] [25] [26] [27] [28] | Revised logic. |
| P19 | Notes | ~~[22]~~ Imaging examination: including CT or MRI of the chest, abdomen, and pelvis.<br>A contrast-enhanced MRI or CT of the brain must also be performed for those suspected of brain metastasis to rule out brain metastasis. Tumor evaluation performed within 3 weeks prior to treatment can be accepted as the baseline evaluation. CT/MRI results prior to informed consent may be used for tumor evaluation at | **[23]** Imaging examination: including **contrast-enhanced** CT or MRI of the chest, abdomen, and pelvis. A contrast-enhanced MRI or CT of the brain must also be performed for those suspected of brain metastasis to rule out brain metastasis. Tumor evaluation performed within 3 weeks prior to treatment can be accepted as the baseline evaluation. CT/MRI results prior to informed consent may be used for tumor evaluation | Revised wording to improve expressions. |

| |
|---|
| Protocol Title: An Open-Label, Multicenter Phase II Clinical Study of Anti-PD-1 Antibody SHR-1210 Combined with Famitinib Malate in Patients with Advanced Urinary System Tumors and Gynecological Tumors |
| Protocol No., Version, and Date: SHR-1210-II-213, V2.0, 6 Aug., 2019 |
| Previous Version: V1.1, 30 Sep., 2018 |
| Revised Version: V2.0, 6 Aug., 2019 |

| Page | Content | Before | After | Reason for Revision |
|---|---|---|---|---|
| | | screening if requirements are met. Patients with evident or suspected bone metastasis must undergo a bone scan. | at screening if requirements are met. Patients with evident or suspected bone metastasis must undergo a bone scan **and those with bone lesions need to undergo CT/MRI for confirmation.** | |
| P20 | Notes | ~~[23]~~ In addition to the PD as evidenced by imaging, ~~patients~~ who have discontinued the study treatment for other reasons must be evaluated at the end of treatment if imaging is not performed within 4 weeks prior to the end of ~~study~~. Also, tumor efficacy should be followed up every 3 months after the ~~end of study~~ until records confirm PD or initiation of new anti-tumor treatment. | **[24]** In addition to the PD as evidenced by imaging, **subjects** who have discontinued the study treatment for other reasons must be evaluated at the end of treatment if the imaging **evaluation** is not performed within 4 weeks prior to the end of **treatment**. Also, tumor efficacy should be followed up every 3 months **(± 7 d) after the end of treatment** until records confirm PD or initiation of new anti-tumor treatment. | Revised wording to improve expressions. |
| P20 | Notes | ~~[24]~~ Survival follow-up: After the study treatment is ~~interrupted~~, the survival status and subsequent anti-tumor treatment can be collected through clinical or telephone follow-ups every 2 months (± 7 d) until death. | **[25]** Survival follow-up: After the study treatment is **discontinued**, the survival status and subsequent anti-tumor treatment can be collected through clinical or telephone follow-ups every 2 months (± 7 d) until death. | Revised wording. |
| P20<br>P68<br>P70 | Notes | [25] ~~In the baseline period, a total of 14 mL of blood samples should be collected into dedicated blood collection tubes from subjects and aliquoted into 2 tubes.~~ Existing paraffin-embedded tumor tissue sections should be collected and 5 tumor sections with a thickness of ~~3-4 μm~~ should be used for PD-L1 detection. It is recommended that fresh biopsy specimens (core needle biopsy) be collected before the first dose. After fixation and | [26] Existing paraffin-embedded tumor tissue sections will be collected, and 5 tumor sections with a thickness of **4-5 μm** will be used for PD-L1 detection. It is recommended that fresh biopsy specimens (core needle biopsy) be collected before the first dose. After fixation and embedding, 3 tumor sections with a thickness of **4-5 μm** will be prepared for PD-L1 detection.<br>**For endometrial cancer and ovarian cancer, no** | Revised based on adjusted biomarker assays. |

| |
|---|
| Protocol Title: An Open-Label, Multicenter Phase II Clinical Study of Anti-PD-1 Antibody SHR-1210 Combined with Famitinib Malate in Patients with Advanced Urinary System Tumors and Gynecological Tumors |
| Protocol No., Version, and Date: SHR-1210-II-213, V2.0, 6 Aug., 2019 |
| Previous Version: V1.1, 30 Sep., 2018 |
| Revised Version: V2.0, 6 Aug., 2019 |

| Page | Content | Before | After | Reason for Revision |
|---|---|---|---|---|
| | | embedding, 3 tumor sections with a thickness of ~~3-4 μm~~ will be prepared for PD-L1 detection; ~~1-2 biopsy samples (without fixation) will be tested for TMB. If it is difficult to perform a biopsy, if the tumor tissue has been collected recently (after the end of the most recent treatment), or if the subject refuses to undergo a biopsy, additional existing paraffin-embedded tumor tissue sections will be collected before administration, with 5-8 paraffin sections (paraffin blocks may be collected directly, without mounting onto slides) with a thickness of 8-10 μm for TMB detection.~~ Refer to the laboratory manual for ~~tumor biomarker blood sampling~~ as well as tumor sample acquisition/collection and processing methods. | **less than 5 additional sections with a thickness of 4-5 μm should be collected for the detection of dMMR. For urothelial carcinoma, no less than 5 additional sections with a thickness of 4-5 μm should be collected for the detection of FGFR2/3 gene abnormality.** Refer to the laboratory manual for tumor **bio**sample acquisition/collection and processing methods. | |
| P20 | Notes | [26] Blood sampling for SHR-1210: Blood samples will be collected within 30 min before administration and within 5 min after administration (including flushing) of the first SHR-1210 dose; within 30 min before administration and within 5 min after administration (including flushing) of SHR-1210 on administration days of Cycles 2, 3, and 4; within 30 min pre-administration every 4 cycles (12 weeks) thereafter; at the ~~end of study~~ (the documented time of last SHR-1210 administration prior to the end of study); at 30 days after the end of treatment. (If subject completes the treatment before scheduled blood sampling is completed, the analysis should be based on actual completed | [27] Blood sampling for SHR-1210: Blood samples will be collected within 30 min before administration and within 5 min after administration (including flushing) of the first SHR-1210 dose; within 30 min before administration and within 5 min after administration (including flushing) of SHR-1210 on administration days of Cycles 2, 3, and 4; within 30 min pre-administration every 4 cycles (12 weeks) thereafter; at the **end of SHR-1210 treatment** (the documented time of last SHR-1210 administration prior to the end of study); at 30 days after the end of **SHR-1210** treatment. (If subject completes the treatment before scheduled blood sampling is completed, the analysis should be based on actual completed sampling). ADA blood | Revised wording to improve expressions. |

| Protocol Title: An Open-Label, Multicenter Phase II Clinical Study of Anti-PD-1 Antibody SHR-1210 Combined with Famitinib Malate in Patients with Advanced Urinary System Tumors and Gynecological Tumors |
|---|
| Protocol No., Version, and Date: SHR-1210-II-213, V2.0, 6 Aug., 2019 |
| Previous Version: V1.1, 30 Sep., 2018 |
| Revised Version: V2.0, 6 Aug., 2019 |

<table>
<tr><th>Page</th><th>Content</th><th>Before</th><th>After</th><th>Reason for Revision</th></tr>
<tr><td></td><td></td><td>sampling). ADA blood samples will be simultaneously collected along with PK samples before each administration, at the end of treatment, and at 30 days after the end of treatment. For SHR-1210, 4 mL should be collected for PK analysis and 4 mL for ADA analysis at each blood sampling point. The serum should be separated.</td><td>samples will be simultaneously collected along with PK samples before each administration, at the end of treatment, and at 30 days after the end of treatment. For SHR-1210, 4 mL should be collected for PK analysis and 4 mL for ADA analysis at each blood sampling point. The serum should be separated.</td><td></td></tr>
<tr><td>P21, P27</td><td>Schedule of sampling for SHR-1210 PK and ADA analysis</td><td>None</td><td>Schedule of sampling for SHR-1210 PK and ADA analysis
<table>
<tr><th>C1D1</th><th>C2D1</th><th>C3D1</th><th>C4D1</th><th>Every 12 weeks</th><th>End of Treatment</th><th>30 Days After End of Treatment</th></tr>
<tr><td>Within 30 min pre-administration</td><td>Within 30 min pre-administration</td><td>Within 30 min pre-administration</td><td>Within 30 min pre-administration</td><td>Within 30 min pre-administration</td><td rowspan="2">Once</td><td rowspan="2">Once</td></tr>
<tr><td>Within 5 min post-administration (including flushing)</td><td>Within 5 min post-administration (including flushing)</td><td>Within 5 min post-administration (including flushing)</td><td>Within 5 min post-administration (including flushing)</td><td>——</td></tr>
</table>
4 mL should be collected for PK analysis and 4 mL for ADA analysis at each blood sampling point. The serum should be separated.</td><td>Added graphical representations to facilitate accurate understanding and correct operation.</td></tr>
<tr><td>P21</td><td>Schedule of sampling for famitinib PK analysis</td><td>None</td><td>Schedule of sampling for famitinib PK analysis
<table>
<tr><th>C3D1</th><th></th><th></th><th></th><th></th><th></th><th></th></tr>
<tr><td>Pre-Administration</td><td>Within 30 min</td><td>——</td><td>——</td><td>——</td><td>——</td><td>——</td></tr>
<tr><td>Post-Administration</td><td>2 h ± 5 min</td><td>4 h ± 5 min</td><td>6 h ± 5 min</td><td>8 h ± 5 min</td><td>10 h ± 5 min</td><td>24 h ± 5 min</td></tr>
</table>
3 mL of blood will be collected at each sampling time point, and the plasma will be separated</td><td>Added graphical representations to facilitate accurate understanding and correct operation.</td></tr>
<tr><td>P22-27<br>P70-71</td><td>Schedule of activities and its notes, and study procedures<br>Dose level of</td><td>None</td><td>All contents were newly added, mainly including:<br>"Dose level of SHR-1210 q2w":<br>C1D15 (± 3 d): [Hematology] [Blood biochemistry] [Urinalysis] [Coagulation function] [Vital signs] [Physical examination] [ECG] [Adverse events]</td><td>Revised based on the added tolerability study of SHR-1210 q2w combined with famitinib.</td></tr>
</table>

| | |
|---|---|
| Protocol Title: An Open-Label, Multicenter Phase II Clinical Study of Anti-PD-1 Antibody SHR-1210 Combined with Famitinib Malate in Patients with Advanced Urinary System Tumors and Gynecological Tumors | |
| Protocol No., Version, and Date: SHR-1210-II-213, V2.0, 6 Aug., 2019 | |
| Previous Version: V1.1, 30 Sep., 2018 | |
| Revised Version: V2.0, 6 Aug., 2019 | |

| Page | Content | Before | After | Reason for Revision |
|---|---|---|---|---|
| | famitinib combined with SHR-1210 q2w | | [Concomitant medication]<br>"Dose level of SHR-1210 q2w":<br>C2D1 (± 3 d): [Hematology] [Blood biochemistry] [Urinalysis] [Coagulation function] [Routine stool test] [Thyroid function] [Vital signs] [Physical examination and weight measurement] [ECOG PS score] [ECG] [SHR-1210 intravenous infusion] [Dispensation/return of famitinib] [Adverse events] [Concomitant medication]; **[Thyroid function test]** will be performed on D1 ± 7 d of every 2 cycles from C2 onwards<br>D15 of C2-C35 (± 3 d): [Hematology] [Blood biochemistry] [Urinalysis] [Vital signs] [Physical examination] [SHR-1210 intravenous infusion] [Adverse events] [Concomitant medication]<br>The examinations should be performed once every 2 cycles for the "dose level of SHR-1210 q2w". Contrast-enhanced MRI or CT of the brain must also be performed for suspected brain metastasis.<br>The first 12 subjects enrolled **at each dose level** in this study will undergo **[Blood sampling for PK and ADA analysis]** during the treatment period | |
| P35 | 1.1. Background | This clinical study involves recombinant humanized anti-PD-1 monoclonal antibody injection (SHR-1210), a new class 1 therapeutic biological product developed by Jiangsu Hengrui Pharmaceuticals Co., Ltd. ~~that has not been~~ | This clinical study involves recombinant humanized anti-PD-1 monoclonal antibody injection (SHR-1210), a new class 1 therapeutic biological product developed by Jiangsu Hengrui Pharmaceuticals Co., Ltd. **that has been officially** | Updated the study progress. |

| |
|---|
| Protocol Title: An Open-Label, Multicenter Phase II Clinical Study of Anti-PD-1 Antibody SHR-1210 Combined with Famitinib Malate in Patients with Advanced Urinary System Tumors and Gynecological Tumors |
| Protocol No., Version, and Date: SHR-1210-II-213, V2.0, 6 Aug., 2019 |
| Previous Version: V1.1, 30 Sep., 2018 |
| Revised Version: V2.0, 6 Aug., 2019 |

| Page | Content | Before | After | Reason for Revision |
|---|---|---|---|---|
| | | ~~marketed both in China and abroad~~. Preclinical trial data show that SHR-1210 has comparable *in vivo* efficacy and safety with those of similar drugs abroad. Since 2015, Hengrui has conducted phase I/II clinical trials on several types of tumors in both Australia and China, and preliminarily validated the safety, tolerability, and efficacy of SHR-1210 monotherapy in the treatment of advanced solid tumors. For details regarding SHR-1210, refer to the SHR-1210 Investigator's Brochure provided by the sponsor. | **approved by the National Medical Products Administration on 29 May, 2019 for the treatment of recurrent or refractory classical Hodgkin lymphoma (cHL) that has been treated with at least two lines of systemic chemotherapy**. Preclinical trial data show that SHR-1210 has comparable *in vivo* efficacy and safety with those of similar drugs abroad. Since 2015, Hengrui has conducted phase I/II clinical trials on several types of tumors in both Australia and China, and preliminarily validated the safety, tolerability, and efficacy of SHR-1210 monotherapy in the treatment of advanced solid tumors. For details regarding SHR-1210, refer to the SHR-1210 Investigator's Brochure provided by the sponsor. | |
| P38-39 | 1.2.1. Study rationale | The anti-PD-1 antibody camrelizumab (SHR-1210) has been formally approved by the National Medical Products Administration on 29 May, 2019 for the treatment of recurrent or refractory classical Hodgkin lymphoma (cHL) that has been treated with at least two lines of systemic chemotherapy. ~~The phase I/II clinical studies have been completed.~~ Subject enrollment has been completed in multiple pivotal phase III studies. The published study results showed that the efficacy of SHR-1210 for a variety of tumors is comparable to that of similar products, and the toxicity information is comprehensive~~. It is~~ | The anti-PD-1 antibody camrelizumab (SHR-1210) has been **formally approved by the National Medical Products Administration on 29 May, 2019 for the treatment of recurrent or refractory classical Hodgkin lymphoma (cHL) that has been treated with at least two lines of systemic chemotherapy**. Subject enrollment has been completed in multiple pivotal phase III studies. The published study results showed that the efficacy of SHR-1210 for a variety of tumors is comparable to that of similar products, and the toxicity information is comprehensive. | Updated the study progress. |

| |
|---|
| Protocol Title: An Open-Label, Multicenter Phase II Clinical Study of Anti-PD-1 Antibody SHR-1210 Combined with Famitinib Malate in Patients with Advanced Urinary System Tumors and Gynecological Tumors |
| Protocol No., Version, and Date: SHR-1210-II-213, V2.0, 6 Aug., 2019 |
| Previous Version: V1.1, 30 Sep., 2018 |
| Revised Version: V2.0, 6 Aug., 2019 |

| Page | Content | Before | After | Reason for Revision |
|---|---|---|---|---|
| | | ~~currently in the stage of new drug application.~~ | | |

| | |
|---|---|
| Protocol Title: An Open-Label, Multicenter Phase II Clinical Study of Anti-PD-1 Antibody SHR-1210 Combined with Famitinib Malate in Patients with Advanced Urinary System Tumors and Gynecological Tumors | |
| Protocol No., Version, and Date: SHR-1210-II-213, V2.0, 6 Aug., 2019 | |
| Previous Version: V1.1, 30 Sep., 2018 | |
| Revised Version: V2.0, 6 Aug., 2019 | |

| Page | Content | Before | After | Reason for Revision |
|---|---|---|---|---|
| P45 | 2.1. Overview of Study Design | ~~Tumor tissue samples, in sections, paraffin blocks, or biopsy tissue blocks, after the last treatment prior to this study, as well as whole blood~~ for biomarker detection will be acquired/collected from subjects at screening. Tests include but are not limited to the following: PD-L1 expression ~~level~~ and the proportion of positive cells, ~~TMB, and~~ MMR (for endometrial cancer only). | **Existing paraffin-embedded tumor tissue sections** will be acquired/collected during the screening period. **Prior to the first dose, it is also recommended to collect fresh biopsy specimens (core needle biopsy) and prepare 3 tumor sections with a thickness of 4-5 μm after fixation and embedding** for biomarker detection, including but not limited to: proportion of PD-L1-positive cells, MMR (for endometrial cancer **and ovarian cancer only), and FGFR2/3 mutation (for urothelial carcinoma only).** | Revised based on adjusted biomarker assays. |
| P46 | 2.1.2. Follow-up management of subjects with clinically significant toxicity: | Subjects with clinically significant toxicity may receive subsequent SHR-1210 and famitinib combination therapy as assessed by the investigators after they have recovered from toxicity. The investigators may interrupt famitinib, reduce the dose of famitinib, discontinue famitinib, or discontinue the study treatment according to the dose modification principle. | Subjects with clinically significant toxicity may receive subsequent SHR-1210 and famitinib combination therapy as assessed by the investigators after they have recovered from toxicity. The investigators may interrupt famitinib, reduce the dose of famitinib, **modify the method of famitinib administration**, discontinue famitinib, or discontinue the study treatment according to the dose modification principle. | Revised logic. |
| P47 | 2.2.1. Study procedure and arrangement of blood sampling points | The first 12 subjects enrolled in the study will undergo blood sampling for PK and ADA analysis.<br>For SHR-1210 PK analysis, blood samples will be collected within 30 min before administration and within 5 min after administration (including flushing) of the first SHR-1210 dose; within 30 | The first 12 subjects **enrolled at each dose level** in the study will undergo blood sampling for PK and ADA analysis **(for the first 12 subjects in the SHR-1210 200 mg, q2w group, only blood sampling for the analysis of SHR-1210 blood concentration, PK, and ADA is required)**.<br>For SHR-1210 PK analysis, blood samples will be | Revised based on the added tolerability study of SHR-1210 q2w combined with famitinib; revised wording. |

| |
|---|
| Protocol Title: An Open-Label, Multicenter Phase II Clinical Study of Anti-PD-1 Antibody SHR-1210 Combined with Famitinib Malate in Patients with Advanced Urinary System Tumors and Gynecological Tumors |
| Protocol No., Version, and Date: SHR-1210-II-213, V2.0, 6 Aug., 2019 |
| Previous Version: V1.1, 30 Sep., 2018 |
| Revised Version: V2.0, 6 Aug., 2019 |

| Page | Content | Before | After | Reason for Revision |
|---|---|---|---|---|
| | | min before administration and within 5 min after administration (including flushing) of SHR-1210 on administration days of Cycles 2, 3, and 4; within 30 min pre-administration every 4 cycles (12 weeks) thereafter; at the end of ~~study~~ (the documented time of last SHR-1210 administration prior to the ~~end of study~~); at 30 days after the end of treatment. (If subject completes the treatment before scheduled blood sampling is completed, the analysis should be based on actual completed sampling).<br>ADA blood samples will be simultaneously collected along with PK samples before each administration, at the end of treatment, and at 30 days after the end of treatment. | collected within 30 min before administration and within 5 min after administration (including flushing) of the first SHR-1210 dose; within 30 min before administration and within 5 min after administration (including flushing) of SHR-1210 on administration days of Cycles 2, 3, and 4; within 30 min pre-administration every 4 cycles (12 weeks) thereafter; at the end of **SHR-1210 treatment** or **upon withdrawal from study** (the documented time of last SHR-1210 administration prior to the end of treatment or withdrawal from study); at 30 days after the end of **SHR-1210** treatment. (If subject completes the treatment before scheduled blood sampling is completed, the analysis should be based on actual completed sampling).<br>ADA blood samples will be simultaneously collected along with PK samples before each administration, at the end of treatment **or upon withdrawal from study**, and at 30 days after the end of treatment. | |
| P48 | 2.2.2. Processing and testing of blood samples | Blood sampling before and after the administration of SHR-1210: At each blood sampling point before administration, 2 tubes of 4 mL of whole blood will be collected into coagulation tubes, and the coagulation tubes should be inverted several times to mix the blood sample evenly. After being let stand at room temperature for 30 min, the blood sample should be centrifuged at 4 °C and 1500 g | Blood sampling before and after the administration of SHR-1210: At each blood sampling point before administration, 2 tubes of 4 mL of whole blood will be collected into coagulation tubes, and the coagulation tubes should be inverted several times to mix the blood sample evenly. After being let stand at room temperature for 30 min **to 1 h until blood agglutination**, the blood sample should be | Revised according to the laboratory manual. |

| |
|---|
| Protocol Title: An Open-Label, Multicenter Phase II Clinical Study of Anti-PD-1 Antibody SHR-1210 Combined with Famitinib Malate in Patients with Advanced Urinary System Tumors and Gynecological Tumors |
| Protocol No., Version, and Date: SHR-1210-II-213, V2.0, 6 Aug., 2019 |
| Previous Version: V1.1, 30 Sep., 2018 |
| Revised Version: V2.0, 6 Aug., 2019 |

| Page | Content | Before | After | Reason for Revision |
|---|---|---|---|---|
| | | (centrifugal force) for 10 min; then, the supernatant in each tube of whole blood will be aliquoted into 2 tubes (not less than 500 µL per tube).<br>At each blood sampling point after administration, 1 tube of 4 mL of whole blood will be collected into a coagulation tube, and the coagulation tube should be inverted several times to mix the blood sample evenly. After being let stand at room temperature for 30 min, the blood sample should be centrifuged at 4 °C and 1500 g (centrifugal force) for 10 min; then, the supernatant will be aliquoted into 2 tubes (not less than 500 µL per tube).<br>Blood sampling before and after the administration of famitinib: At each blood sampling point, 3 mL of whole blood will be collected into a heparin-containing anticoagulation tube, and the tube will be inverted several times to mix the blood with the anticoagulant. ~~Within 30 min~~Within 2 h after sampling, the blood sample should be centrifuged at 4 °C and 1500 g (centrifugal force) for 10 min; then, the supernatant will be aliquoted into 2 tubes (not less than 500 µL per tube). | centrifuged at 4 °C and 1500 g (centrifugal force) for 10 min; then, the supernatant in each tube of whole blood will be aliquoted into 2 tubes (not less than 500 µL per tube).<br>At each blood sampling point after administration, 1 tube of 4 mL of whole blood will be collected into a coagulation tube, and the coagulation tube should be inverted several times to mix the blood sample evenly. After being let stand at room temperature for 30 min **to 1 h until blood agglutination**, the blood sample should be centrifuged at 4 °C and 1500 g (centrifugal force) for 10 min; then, the supernatant will be aliquoted into 2 tubes (not less than 500 µL per tube).<br>Blood sampling before and after the administration of famitinib: At each blood sampling point, 3 mL of whole blood will be collected into a heparin-containing anticoagulation tube, and the tube will be inverted several times to mix the blood with the anticoagulant. **Within 2 h** after sampling, the blood sample should be centrifuged at 4 °C and 1500 g (centrifugal force) for 10 min; then, the supernatant will be aliquoted into 2 tubes (not less than 500 µL per tube).<br>**Refer to the laboratory manual for details on sample acquisition, processing, and transportation methods.** | |

| |
|---|
| Protocol Title: An Open-Label, Multicenter Phase II Clinical Study of Anti-PD-1 Antibody SHR-1210 Combined with Famitinib Malate in Patients with Advanced Urinary System Tumors and Gynecological Tumors |
| Protocol No., Version, and Date: SHR-1210-II-213, V2.0, 6 Aug., 2019 |
| Previous Version: V1.1, 30 Sep., 2018 |
| Revised Version: V2.0, 6 Aug., 2019 |

<table>
<tr><th>Page</th><th>Content</th><th>Before</th><th>After</th><th>Reason for Revision</th></tr>
<tr>
<td>P48-49, P68</td>
<td>2.3. Acquisition, Collection, and Processing of Biomarkers</td>
<td>~~1) Fourteen mL of blood samples will be collected before the first dose and aliquoted into two tubes, with 10 mL in one tube and 4 mL in the other for the detection of ctDNA, circulating tumor cells, and PD-L1 of circulating tumor cells.~~<br>2) Existing paraffin-embedded tumor tissue sections should be collected and 5 tumor sections with a thickness of ~~3-4 μm~~ should be used for PD-L1 detection.<br>3) It is recommended that biopsy samples (core needle biopsy) be collected before the first dose. After fixation and embedding, 3 tumor sections with a thickness of ~~3-4 μm~~ will be prepared for PD-L1 detection;<br>~~4) Another 1-2 biopsy samples (without fixation) will be stored directly in a dedicated tissue collection tube containing RNase protection solution for detections including TMB.~~<br>~~If it is difficult to perform a biopsy, if the tumor tissue has been collected recently (after the end of the most recent treatment), or if the subject refuses to undergo a biopsy, additional existing paraffin-embedded tumor tissue sections will be collected before administration, with at least 8 paraffin sections (paraffin blocks may be collected directly, without mounting onto slides) with a thickness of 8-10 μm for TMB detection.~~<br>**Table 4. Acquisition and collection of biomarker samples.**</td>
<td>1) Existing paraffin-embedded tumor tissue sections should be collected and 5 tumor sections with a thickness of 4-5 μm should be used for PD-L1 detection.<br>2) It is recommended that biopsy samples (core needle biopsy) be collected before the first dose. After fixation and embedding, 3 tumor sections with a thickness of 4-5 μm will be prepared for PD-L1 detection;<br>**3) For endometrial cancer and ovarian cancer, no less than 5 additional sections with a thickness of 4-5 μm should be collected for the detection of dMMR;**<br>**4) For urothelial carcinoma, no less than 5 additional sections with a thickness of 4-5 μm should be collected for the detection of FGFR2/3 gene abnormality.**<br>**Table 4. Acquisition and collection of biomarker samples.**
<table>
<tr><th>Sample Type</th><th>Collection Requirement</th></tr>
<tr><td colspan="2">Screening Period</td></tr>
<tr><td>Previously archived samples</td><td>5 unstained sections with a thickness of 4-5 μm</td></tr>
<tr><td>Tumor biopsy samples recommended (before the first dose)</td><td>After fixation and embedding, 3 unstained sections with a thickness of 4-5 μm</td></tr>
<tr><td colspan="2">Collection of Additional Tumor Tissue Samples for Specific Tumor Types</td></tr>
<tr><td>For ovarian cancer, endometrial cancer, or urothelial carcinoma</td><td>No less than 5 additional unstained sections with a thickness of 4-5 μm</td></tr>
</table>
</td>
<td>Revised based on adjusted biomarker assays.</td>
</tr>
</table>

<table>
<tr><th>Page</th><th>Content</th><th>Before</th><th>After</th><th>Reason for Revision</th></tr>
<tr><td></td><td></td><td><table>
<tr><th>Sample Type</th><th>Collection Requirement</th></tr>
<tr><td colspan="2">Screening Period</td></tr>
<tr><td>Peripheral blood (before first study medication)</td><td>1. 10 mL, 1 streck tube.<br>2. 4 mL, 1 streck tube.</td></tr>
<tr><td>Previously archived samples</td><td>5 unstained sections with a thickness of 3-4 μm;<br>no less than 8 sections with a thickness of 8-10 μm; paraffin blocks directly collected without mounting onto slides (if biopsy not available).</td></tr>
<tr><td>Tumor biopsy samples (before first study medication)</td><td>After fixation and embedding,<br>3 unstained sections with a thickness of 3-4 μm.</td></tr>
<tr><td>Tumor biopsy samples (before first study medication)</td><td>1-2 samples in tissue collection tubes containing RNase protection solution.</td></tr>
</table>
The acquired/collected ~~peripheral blood samples and~~ tumor tissue samples will be transported to the designated central laboratory at room temperature. No processing by the study site is required.</td><td>The acquired/collected tumor tissue samples will be transported to the designated central laboratory at room temperature. No processing by the study site is required.</td><td></td></tr>
<tr><td>P52</td><td>Exclusion criteria</td><td>3. Severe allergic reactions to other monoclonal antibodies, ~~or allergic constitution;~~</td><td>3. Severe allergic reactions to other monoclonal antibodies.</td><td>Revised logic.</td></tr>
<tr><td>P53-54</td><td>3.3.1. Contraception</td><td>In ~~this~~ study, camrelizumab (SHR-1210) and famitinib have been suspected of teratogenicity/fetal toxicity, but whether they have transient adverse impact on components of sperm is still unknown. Therefore, for all female subjects of childbearing potential and male subjects with female partners of childbearing potential who receive the study treatment, if the investigators determine that they or their</td><td>In this study, camrelizumab (SHR-1210) and famitinib have been suspected of teratogenicity/fetal toxicity, but whether they have transient adverse impact on components of sperm is still unknown. Therefore, for all female subjects of childbearing potential and male subjects with female partners of childbearing potential who receive the study treatment, if the investigators determine that they or</td><td>Refined expressions.</td></tr>
</table>

| |
|---|
| Protocol Title: An Open-Label, Multicenter Phase II Clinical Study of Anti-PD-1 Antibody SHR-1210 Combined with Famitinib Malate in Patients with Advanced Urinary System Tumors and Gynecological Tumors |
| Protocol No., Version, and Date: SHR-1210-II-213, V2.0, 6 Aug., 2019 |
| Previous Version: V1.1, 30 Sep., 2018 |
| Revised Version: V2.0, 6 Aug., 2019 |

| Page | Content | Before | After | Reason for Revision |
|---|---|---|---|---|
| | | partners are at risk of pregnancy, the subjects must adopt at least two ~~effective~~ contraceptive measures during the entire treatment period from the signing of the ICF until at least ~~90 days~~ after the ~~last dose~~. | their partners are at risk of pregnancy, the subjects **and their partners** must adopt at least two **effective** contraceptive measures during the entire treatment period from the signing of the ICF until at least **3 months** after **the end of the treatment period**. | |
| P56 | 3.6. Definition of End of Study | After the end of study, if the subjects continue to benefit from the investigational products, the treatment can be continued until the criteria for ~~interruption~~ are met. The occurrence of SAEs will be collected and recorded during treatment and after the last dose according to the protocol. | After the end of study, if the subjects continue to benefit from the investigational products, the treatment can be continued until the criteria for **discontinuation or study withdrawal** are met. The occurrence of SAEs will be collected and recorded during treatment and after the last dose according to the protocol. | Revised wording to improve expressions. |
| P57-58 | 4.2.1. Packaging and labeling | / | Added "q2w" to the method of administration of SHR-1210;<br>Added "31 capsules/bottle" to the packaging strength and quantity for famitinib;<br>Corrected clerical errors: "200 mg/20 mL vial" should be "200 mg vial" in packaging strength and quantity for SHR-1210;<br>famitinib is available in 2 strengths: 20 mg**/capsule** and 15 mg**/capsule** | Revised based on the added tolerability study of SHR-1210 q2w combined with famitinib. |
| P59 | 4.4. Drug Preparation, Dispensation, and Return | **Deleted:**<br>~~The total storage period (overall duration in the refrigerator and room temperature storage) from the preparation of SHR-1210 to administration should not exceed 24 h.~~ | | No need to repeat the content here as it is detailed in the pharmacy manual. |

| |
|---|
| Protocol Title: An Open-Label, Multicenter Phase II Clinical Study of Anti-PD-1 Antibody SHR-1210 Combined with Famitinib Malate in Patients with Advanced Urinary System Tumors and Gynecological Tumors |
| Protocol No., Version, and Date: SHR-1210-II-213, V2.0, 6 Aug., 2019 |
| Previous Version: V1.1, 30 Sep., 2018 |
| Revised Version: V2.0, 6 Aug., 2019 |

| Page | Content | Before | After | Reason for Revision |
|---|---|---|---|---|
| P59 | 4.5. Administration of Investigational Products | **Deleted:** ~~Subjects must complete all clinically required examinations except for imaging examination within 72 h before each dose to determine whether continuing the medication is appropriate.~~ | | Revised logic. |
| P60 | 4.6. Dosing Regimen | / | **Added:** For subjects at the "dose level of q2w", the drug will be administered once every 2 weeks in cycles of 4 weeks.<br>For subjects at the "dose level of SHR-1210 q2w", SHR-1210 will be administered once every 2 weeks with a time window of ± 3 days from the scheduled administration time.<br>For subjects at the "dose level of SHR-1210 q2w", famitinib capsules will be administered continuously in cycles of 4 weeks. | Revised based on the added tolerability study of SHR-1210 q2w combined with famitinib. |
| P60 | 4.6. Dosing Regimen | If an SHR-1210 dose is delayed by more than ~~7 days~~ beyond the scheduled administration time, then the dose should be skipped, and the administration should be resumed at the original dose at the next scheduled time point. | If an SHR-1210 dose is delayed beyond the scheduled administration time, then the dose should be skipped, and the administration should be resumed at the original dose at the next scheduled time point. | Revised logic. |
| P60 | 4.7. Dose Modification | In this study, SHR-1210 treatment may be interrupted for up to 12 weeks; | In this study, SHR-1210 treatment may be interrupted for up to 12 **consecutive** weeks; | Refined expressions. |
| P60 | 4.7. Dose Modification | Grade 4 ~~injection~~ reaction | Grade 4 **infusion** reaction | Revised wording. |

| |
|---|
| Protocol Title: An Open-Label, Multicenter Phase II Clinical Study of Anti-PD-1 Antibody SHR-1210 Combined with Famitinib Malate in Patients with Advanced Urinary System Tumors and Gynecological Tumors |
| Protocol No., Version, and Date: SHR-1210-II-213, V2.0, 6 Aug., 2019 |
| Previous Version: V1.1, 30 Sep., 2018 |
| Revised Version: V2.0, 6 Aug., 2019 |

| Page | Content | Before | After | Reason for Revision |
|---|---|---|---|---|
| P63 | Table 5. Dose modifications. | ~~Capillary hemangioma~~ | **Reactive cutaneous capillary endothelial proliferation** | Revised wording to be consistent with the name of the adverse reaction in the package insert. |
| P65 | 4.8.2. Supportive care | Subjects should be given optimal supportive care during treatment. Comorbidities and various adverse reactions, especially immune-related adverse reactions, should be actively treated. | Subjects should be given optimal supportive care during treatment. Comorbidities and various adverse reactions, especially immune-related adverse reactions, should be actively treated. **Oral administration of megestrol acetate for promoting appetite during treatment is permitted for subjects in the renal cancer, urothelial carcinoma, and cervical cancer cohorts. Oral administration of megestrol acetate for promoting appetite is not allowed for subjects in the endometrial cancer and ovarian cancer cohorts.** | Clarified the scope of use of megestrol acetate. |
| P72 | 5.4. End of Treatment/ Withdrawal | In addition, the first 12 subjects enrolled in the study will undergo blood sampling for SHR-1210 PK and ADA analysis at the end of treatment, 4 mL each. | In addition, the first 12 subjects enrolled in the study will undergo blood sampling for SHR-1210 PK and ADA analysis at the end of **SHR-1210** treatment, 4 mL each. | Refined expressions. |
| P72 | 5.5. Follow-Up Period | The first 12 subjects enrolled in the study will undergo blood sampling for SHR-1210 PK and ADA analysis at 30 days after the end of treatment, 4 mL each. | The first 12 subjects enrolled in the study will undergo blood sampling for SHR-1210 PK and ADA analysis at 30 days after the end of **SHR-1210** treatment, 4 mL each. | Refined expressions. |

| | |
|---|---|
| Protocol Title: An Open-Label, Multicenter Phase II Clinical Study of Anti-PD-1 Antibody SHR-1210 Combined with Famitinib Malate in Patients with Advanced Urinary System Tumors and Gynecological Tumors | |
| Protocol No., Version, and Date: SHR-1210-II-213, V2.0, 6 Aug., 2019 | |
| Previous Version: V1.1, 30 Sep., 2018 | |
| Revised Version: V2.0, 6 Aug., 2019 | |

| Page | Content | Before | After | Reason for Revision |
|---|---|---|---|---|
| P73 | 6.1.1. Efficacy endpoints | • Duration of response (DOR): defined as the period of time from the first documented tumor response (as per RECIST 1.1) to the first documented objective progression (as per RECIST 1.1) or death of any cause. Subjects without PD or death will be censored on the date of their last tumor evaluation. Subjects who start new anti-tumor treatment (not including treatment for non-target bone lesions or palliative radiotherapy) without previously reported progression will be censored on the date of their last tumor evaluation prior to the start of new anti-tumor treatment ~~or on the date of starting the new anti-tumor treatment~~. | • Duration of response (DOR): defined as the period of time from the first documented tumor response (as per RECIST 1.1) to the first documented objective progression (as per RECIST 1.1) or death of any cause, **whichever occurs first**. Subjects without PD or death will be censored on the date of their last tumor evaluation. Subjects who start new anti-tumor treatment (not including treatment for non-target bone lesions or palliative radiotherapy) without previously reported progression will be censored on the date of their last tumor evaluation prior to the start of new anti-tumor treatment. | Refined expressions. |
| P74 | 6.1.1. Efficacy endpoints | • Time to objective response (TTR): defined as the period of time from the date of first dose to the first documented tumor response (as per RECIST 1.1). Subjects without PD or death will be censored on the date of their last tumor evaluation. Subjects who start new anti-tumor treatment (not including palliative radiotherapy for non-target bone lesions) without previously reported progression will be censored on the date of their last tumor evaluation prior to the start of new anti-tumor treatment ~~or on the date of starting the new anti-tumor treatment~~. | • Time to objective response (TTR): defined as the period of time from the date of first dose to the first documented tumor response (as per RECIST 1.1). Subjects without PD or death will be censored on the date of their last tumor evaluation. Subjects who start new anti-tumor treatment (not including palliative radiotherapy for non-target bone lesions) without previously reported progression will be censored on the date of their last tumor evaluation prior to the start of new anti-tumor treatment. | Refined expressions. |

| |
|---|
| Protocol Title: An Open-Label, Multicenter Phase II Clinical Study of Anti-PD-1 Antibody SHR-1210 Combined with Famitinib Malate in Patients with Advanced Urinary System Tumors and Gynecological Tumors |
| Protocol No., Version, and Date: SHR-1210-II-213, V2.0, 6 Aug., 2019 |
| Previous Version: V1.1, 30 Sep., 2018 |
| Revised Version: V2.0, 6 Aug., 2019 |

| Page | Content | Before | After | Reason for Revision |
|---|---|---|---|---|
| P74 | 6.1.1. Efficacy endpoints | When determining PFS, clinical exacerbations without definite evidence of PD (as per RECIST 1.1) is not considered progression. For subjects who die without any prior reports of progression, the date of death is considered the date of progression. Subjects without PD or death will be censored on the date of their last evaluable tumor assessment. Subjects without tumor evaluation during the study or death will be censored on their date of first dose. ~~Subjects who discontinue the study for reasons other than PD (no subsequent imaging examinations) will be censored on their date of study discontinuation.~~ Subjects who have no previously reported PD but have started new anti-tumor treatment will be censored on the date of their last evaluable tumor assessment prior to the start of new anti-tumor treatment ~~or on the date of starting the new anti-tumor treatment~~. ~~When subjects are not censored on the date of study discontinuation or the date of starting new anti-tumor treatment, the scheduled sensitivity analysis will confirm PFS based only on the time of radiographically confirmed progression events. The occurrence of a new tumor will not be considered a progression event and is not censored~~. | When determining PFS, clinical exacerbations without definite evidence of PD (as per RECIST 1.1) is not considered progression. For subjects who die without any prior reports of progression, the date of death is considered the date of progression. Subjects without PD or death will be censored on the date of their last evaluable tumor assessment. Subjects without tumor evaluation during the study or death will be censored on their date of first dose. Subjects who have no previously reported PD but have started new anti-tumor treatment will be censored on the date of their last evaluable tumor assessment prior to the start of new anti-tumor treatment. | Refined expressions. |

| |
|---|
| Protocol Title: An Open-Label, Multicenter Phase II Clinical Study of Anti-PD-1 Antibody SHR-1210 Combined with Famitinib Malate in Patients with Advanced Urinary System Tumors and Gynecological Tumors |
| Protocol No., Version, and Date: SHR-1210-II-213, V2.0, 6 Aug., 2019 |
| Previous Version: V1.1, 30 Sep., 2018 |
| Revised Version: V2.0, 6 Aug., 2019 |

<table>
<tr><th>Page</th><th>Content</th><th>Before</th><th>After</th><th>Reason for Revision</th></tr>
<tr>
<td>P75</td>
<td>6.1.2. Criteria for efficacy evaluation</td>
<td>Lesions found at baseline will be assessed every 3 cycles (9 weeks) during treatment ~~(bone scan will be performed for suspected bone progression or CR confirmation) and the assessments are not affected by treatment interruption or delay~~. Tumor evaluation may also be performed if new lesions are suspected. The first documentation of PR/CR in a subject must be confirmed 4 weeks ± 7 days later~~.~~ An imaging examination for confirmation is required 4-6 weeks after the documentation of PD as per RECIST v1.1 (except those with rapid progression or significant clinical progression).</td>
<td>Lesions found at baseline will be assessed every 3 cycles (9 weeks) during treatment. **Lesions found at baseline will be assessed every 2 cycles (8 weeks) during treatment for subjects enrolled at the SHR-1210 q2w dose level, and the assessments are not affected by treatment interruption or delay. Bone scan will be performed for suspected bone progression or CR confirmation.** Tumor evaluation may also be performed if new lesions are suspected. The first documentation of PR/CR in a subject must be confirmed 4 weeks ± 7 days later**.** If there is PD as per RECIST v1.1, an imaging examination is required for confirmation 4-6 weeks later (except those with rapid progression or significant clinical progression)**; after the first PD, the following table can be referred to for the criteria of PD confirmation (the criteria below are stipulated in reference to the judgment criteria of iCPD in the iRECIST):**
<table>
<tr><td>Cause of First PD</td><td>Criteria for Confirming PD</td></tr>
<tr><td>Progression of Target Lesions</td><td>1. The absolute value of SoD increases by ≥ 25 mm compared with the minimum SoD measured in the first PD and after the first PD<br>2. Progression of target lesions<br>3. Appearance of new lesions</td></tr>
<tr><td>Progression of Non-Target Lesions</td><td>1. SoD increases by ≥ 20% and the absolute value of SoD increases by ≥ 25 mm compared with the minimum SoD measured during the screening period and the treatment period<br>2. Further progression of non-target lesions</td></tr>
</table>
</td>
<td>Revised based on the added tolerability study of SHR-1210 q2w combined with famitinib.<br>Refined expressions to facilitate understanding and operation.</td>
</tr>
</table>

| |
|---|
| Protocol Title: An Open-Label, Multicenter Phase II Clinical Study of Anti-PD-1 Antibody SHR-1210 Combined with Famitinib Malate in Patients with Advanced Urinary System Tumors and Gynecological Tumors |
| Protocol No., Version, and Date: SHR-1210-II-213, V2.0, 6 Aug., 2019 |
| Previous Version: V1.1, 30 Sep., 2018 |
| Revised Version: V2.0, 6 Aug., 2019 |

| Page | Content | Before | After | Reason for Revision |
|---|---|---|---|---|
| | | | (continued) 3. Appearance of new lesions<br>Appearance of New Lesions: 1. SoD increases by ≥ 20% and the absolute value of SoD increases by ≥ 25 mm compared with the minimum SoD measured during the screening period and the treatment period 2. Progression of non-target lesions 3. Re-appearance of new lesions 4. The absolute value of sum of diameters (short axis for lymph nodes) of new lesions increases by ≥ 5 mm (for measurable new lesions) 5. Further progression of new lesions (for non-measurable lesions).<br>**SoD: the sum of diameters of target lesions** | |
| P76 | 6.2.2. Adverse event | The assessment of AEs include type, incidence, severity (according to NCI-CTCAE v4.03), start and end date, whether it is an SAE, causality, and outcome. | The assessment of AEs include type, incidence, severity (according to NCI-CTCAE v4.03), start and end date, whether it is an SAE**, whether it is an irAE, whether it is an SIE**, causality, and outcome. | Revised logic and added the content related to AE assessment. |
| P78 | 6.4. Biomarker Evaluation | The expression level of PD-L1 in tumor tissue will be assessed by immunohistochemistry; ~~The level of circulating tumor cells (CTCs) in peripheral blood will be assessed by magnetic bead capture, and the proportion of PD-L1-positive CTCs will be assessed by PD-L1 staining of the captured CTCs;~~ ~~The level of tumor mutation burden (TMB) in tumor tissue and peripheral blood will be tested using a next-generation sequencing method.~~ | The expression level of PD-L1 in tumor tissue will be assessed by immunohistochemistry; **the MMR of tumor tissue will be assessed by immunohistochemistry (for endometrial cancer and ovarian cancer only); the FGFR2/3 mutation will be assessed by immunohistochemistry/next-generation sequencing (for urothelial carcinoma only);** | Revised based on adjusted biomarker assays. |
| P79-80 | 8.1. Adverse | AEs will be collected from the signing of the ICF until at least 30 days after the last dose. | AEs will be collected from the signing of the ICF until at least 30 days after the last **study** dose. SAEs | Refined expressions. |

| |
|---|
| Protocol Title: An Open-Label, Multicenter Phase II Clinical Study of Anti-PD-1 Antibody SHR-1210 Combined with Famitinib Malate in Patients with Advanced Urinary System Tumors and Gynecological Tumors |
| Protocol No., Version, and Date: SHR-1210-II-213, V2.0, 6 Aug., 2019 |
| Previous Version: V1.1, 30 Sep., 2018 |
| Revised Version: V2.0, 6 Aug., 2019 |

| Page | Content | Before | After | Reason for Revision |
|---|---|---|---|---|
| | Event | SAEs and irAEs observed within 90 days after the last dose of SHR-1210 should be followed up and collected. If subjects start new anti-tumor treatment, they should be ~~followed up until they start the new anti-tumor treatment~~. | and irAEs that occur within 90 days after the last dose of SHR-1210 will be followed up and collected. **Meanwhile**, if a subject starts new anti-tumor treatment, **only SAEs related to the investigational products will be collected thereafter**. | |
| P81 | 8.1.3. Causality assessment | ~~AEs should be collected and documented from the signing of the ICF until the end of the safety follow-up period~~, regardless of whether the event is related to the investigational products, ~~whether the subject is allocated to the investigational product group,~~ or even whether the medication is administered. ~~Events deemed "related", "possibly related", "unlikely related", and "unassessable" should be listed as adverse drug reactions. When calculating the incidence of adverse reactions, the total of these four categories should be used as the numerator and the total number of subjects for safety assessments should be used as the denominator.~~ | **AEs include all unexpected clinical manifestations. All the AEs occurring after the signing of the ICF** must be reported and recorded, regardless of whether the AEs are related to the investigational products and whether the subject has been administered with the products. | Refined expressions and deleted unnecessary description. Updated according to the company's template (the classification of drug-related adverse reactions will be performed according to the requirements of regulatory authorities during analysis and will not be pre-specified in the protocol). |
| P83 | 8.2.3. Progressive disease | / | **Added:**<br>In the study population of this study, "PD" is an expected situation and should not be documented as AE. When PD occurs, events that confirm PD should be reported as AEs. For example, for a subject with epilepsy determined to be related to | Explained the reported term "SAE of death due to PD" in detail. |

| Protocol Title: An Open-Label, Multicenter Phase II Clinical Study of Anti-PD-1 Antibody SHR-1210 Combined with Famitinib Malate in Patients with Advanced Urinary System Tumors and Gynecological Tumors |
|---|
| Protocol No., Version, and Date: SHR-1210-II-213, V2.0, 6 Aug., 2019 |
| Previous Version: V1.1, 30 Sep., 2018 |
| Revised Version: V2.0, 6 Aug., 2019 |

<table>
<tr><th>Page</th><th>Content</th><th>Before</th><th>After</th><th>Reason for Revision</th></tr>
<tr><td></td><td></td><td></td><td>brain metastases, the AE term "epilepsy" rather than "PD" or "brain metastasis" should be documented.<br>Death caused by the symptoms and signs of PD will be reported as an SAE. The term "death" should not be used as an AE or SAE, but an outcome of an event. Events that result in death should be recorded as AEs or SAEs. If the cause of death is unknown and cannot be determined at the time of reporting, the AE or SAE term "death of unknown cause" should be used for documentation.</td><td></td></tr>
<tr><td>P83</td><td>8.2.4. Hepatic enzyme abnormalities</td><td>~~For two consecutive observations of abnormal AST and/or ALT levels with abnormal TBIL elevation, if all of the following conditions are met without other possible causes, the abnormality should be regarded as an important medical event and reported as an SAE.~~
<table>
<tr><th>Baseline Period</th><th>Normal (AST/ALT and TBIL)</th><th>Abnormal (AST/ALT and TBIL)</th></tr>
<tr><td>Treatment Period</td><td>• ALT or AST ≥ 3 × ULN<br>• With TBIL ≥ 2 × ULN<br>• And alkaline phosphatase ≤ 2 × ULN<br>• And no hemolysis</td><td>• AST or ALT ≥ 2 × baseline level and value ≥ 3 × ULN; or AST or ALT ≥ 8 × ULN<br>• With TBIL increase ≥ 1 × ULN or TBIL ≥ 3 × ULN</td></tr>
</table></td><td>**For abnormal AST and/or ALT levels with abnormal TBIL elevation, if all of the following conditions (1), (2), and (3) are met without other possible causes, the abnormality should be regarded as an important medical event and reported in accordance with SAE procedures.**
<table>
<tr><th>Condition</th><th>Criterion</th></tr>
<tr><td>(1) Abnormal ALT or AST</td><td>Normal at baseline: ALT or AST ≥ 3 × ULN during treatment period;<br>Abnormal at baseline: ALT or AST ≥ 2 × baseline level and value ≥ 3 × ULN during treatment period; or value ≥ 8 × ULN.</td></tr>
<tr><td>(2) Abnormal TBIL</td><td>Normal at baseline: TBIL > 2 × ULN during treatment period;<br>Abnormal at baseline: TBIL increase > 1 × ULN or value > 3 × ULN during treatment period.</td></tr>
<tr><td colspan="2">(3) No hemolysis, and alkaline phosphatase < 2 × ULN (or not available)</td></tr>
</table></td><td>Updated the definition of "hepatic enzyme abnormal" and clarified the methods of tracking and treatment</td></tr>
</table>

| |
|---|
| Protocol Title: An Open-Label, Multicenter Phase II Clinical Study of Anti-PD-1 Antibody SHR-1210 Combined with Famitinib Malate in Patients with Advanced Urinary System Tumors and Gynecological Tumors |
| Protocol No., Version, and Date: SHR-1210-II-213, V2.0, 6 Aug., 2019 |
| Previous Version: V1.1, 30 Sep., 2018 |
| Revised Version: V2.0, 6 Aug., 2019 |

| Page | Content | Before | After | Reason for Revision |
|---|---|---|---|---|
| P84 | ~~8.2.5. Other anti-tumor treatments~~ | **Deleted:**<br>~~8.2.5 Other anti-tumor treatments~~<br>~~SAEs should be recorded from the signing of the ICF until the end of the safety follow-up period (90 days after the last dose of SHR-1210). If a subject is to start another anti-tumor treatment before the end of the safety follow-up period, unless suspected to be related to the investigational products, SAEs except death will be reported until the start of the new anti-tumor treatment.~~ | | Deleted unnecessary expressions (The requirements for collecting safety information after the start of other anti-tumor treatments have been described in Section 8.2.6) |
| P84 | 8.2.5. SAE reporting | SAEs should be collected from the signing of the ICF ~~until the end of the safety follow-up period~~. | SAEs should be collected from the signing of the ICF. | Deleted unnecessary expressions (The SAE collection time limits are related to the last dose and causality determination. The specific requirements have been detailed in Section 8.2.6) |
| P85-86 | 8.2.6. Collection and follow-up of AEs/SAEs | 8.2.~~7~~ Follow-up of AEs/SAEs<br>All AEs/SAEs should be followed up until they are resolved, return to baseline levels or Grade ≤ 1, reach a stable state, or are reasonably explained (e.g., loss to follow-up or death).<br>During each visit, the investigators should ask about the situation of AEs/SAEs that occur after | 8.2.**6** **<u>Collection and</u>** follow-up of AEs/SAEs<br>All AEs/SAEs should be followed up until they are resolved, return to baseline levels or Grade ≤ 1, reach a stable state, or are reasonably explained (e.g., loss to follow-up or death).<br>During each visit, the investigators should ask about the situation of AEs/SAEs that occur after the last | Refined expressions for a clearer statement, and clarified the time limits and requirements for AE/SAE collection to |

| |
|---|
| Protocol Title: An Open-Label, Multicenter Phase II Clinical Study of Anti-PD-1 Antibody SHR-1210 Combined with Famitinib Malate in Patients with Advanced Urinary System Tumors and Gynecological Tumors |
| Protocol No., Version, and Date: SHR-1210-II-213, V2.0, 6 Aug., 2019 |
| Previous Version: V1.1, 30 Sep., 2018 |
| Revised Version: V2.0, 6 Aug., 2019 |

<table>
<tr><th>Page</th><th>Content</th><th>Before</th><th>After</th><th>Reason for Revision</th></tr>
<tr><td></td><td></td><td>the last visit and provide follow-up information in a timely manner based on the sponsor's query request. The time limits of AE/SAE collection ~~and follow-up after the last dose of the subject at the end of study~~ are shown in Table 1 below:<br><br>**Table 1. Time limits of AE/SAE collection ~~and follow-up~~.**
<table>
<tr><th>Classification</th><th>Collection/Documentation Requirement</th><th>Follow-Up Requirement</th></tr>
<tr><td>Non-Treatment-Related AE</td><td>Until the end of safety follow-up period or start of new anti-tumor treatment (whichever comes first)</td><td>Until the end of safety follow-up period</td></tr>
<tr><td>Treatment-Related AE</td><td>Until the end of safety follow-up period</td><td>Until resolved, remission, or recovered to baseline levels or Grade ≤ 1, steady state, or reasonably explained (e.g., loss to follow-up, death)</td></tr>
<tr><td>Non-Treatment-Related SAE and SIE</td><td>Until the end of safety follow-up period or start of new anti-tumor treatment (whichever comes first)</td><td>Until the end of safety follow-up period</td></tr>
<tr><td>Treatment-Related SAE and SIE</td><td>No time limit</td><td>Until resolved, remission, or recovered to baseline levels or Grade ≤ 1, steady state, or reasonably explained (e.g., loss to follow-up, death).</td></tr>
</table>
AE: adverse event; SAE: serious adverse event; SIE: special interest event.</td><td>visit and provide follow-up information in a timely manner based on the sponsor's query request. Refer to Table 2below for the time limits of AE/SAE collection:<br><br>**Table 2. Time limits of AE/SAE collection.**
<table>
<tr><th>Time Limit</th><th>Collection/Documentation Requirement</th></tr>
<tr><td>Up to 30 Days (inclusive) After the Last Dose</td><td>Collect all AEs/SAEs</td></tr>
<tr><td rowspan="2">30 to 90 Days After the Last Dose</td><td>Collect all AEs related to SHR-1210</td></tr>
<tr><td>Collect all SAEs if no new anti-tumor treatment starts;<br>If new anti-tumor treatment starts, only SAEs related to the investigational products will be collected after the start of the new anti-tumor treatment.</td></tr>
<tr><td>After 90 Days Post the Last Dose</td><td>Collect SAEs related to the investigational products</td></tr>
</table></td><td>facilitate understanding and operation.</td></tr>
</table>

| |
|---|
| Protocol Title: An Open-Label, Multicenter Phase II Clinical Study of Anti-PD-1 Antibody SHR-1210 Combined with Famitinib Malate in Patients with Advanced Urinary System Tumors and Gynecological Tumors |
| Protocol No., Version, and Date: SHR-1210-II-213, V2.0, 6 Aug., 2019 |
| Previous Version: V1.1, 30 Sep., 2018 |
| Revised Version: V2.0, 6 Aug., 2019 |

| Page | Content | Before | After | Reason for Revision |
|---|---|---|---|---|
| P86 | 8.3. Pregnancy | During the study, if a female subject becomes pregnant, she should discontinue the study treatment immediately. The investigators must ~~report to the sponsor within~~ 24 h ~~and~~ fill out the "Pregnancy Report/Follow-up Form for Hengrui's Clinical Studies".<br>~~During the study, if the partner of a male subject becomes pregnant, the subject can continue in the study. The investigators must report to the sponsor within 24 h and fill out the "Pregnancy Report/Follow-up Form for Hengrui's Clinical Studies".~~ | If a female subject becomes pregnant during the clinical study, the subject must discontinue study treatment immediately; **if the partner of a male subject becomes pregnant during the clinical study, the subject can continue the study.** The investigators **should** report pregnancy **to the sponsor** within 24 h of knowing the event and **to the ethics committee promptly** by filling out the "Pregnancy Report/Follow-up Form for Hengrui's Clinical Studies". | Simplified expressions and added the requirements for timely reporting to the ethics committee. |
| P86-87 | 8.4. Special Interest Event | • Grade ≥ 3 infusion reaction;<br>~~• Grade ≥ 2 diarrhea/colitis, uveitis, and interstitial pneumonia;~~<br>• Other Grade ≥ 3 irAEs;<br>~~Grade 4 amylase or lipase increased.~~ | **In this study, the sponsor will pay special attention to irAEs. If the subject develops an AE of the following type and severity which is determined as possibly related or related to the investigational products, the investigators should promptly inform the sponsor:**<br>• Grade ≥ 3 infusion reaction;<br>• Other Grade ≥ 3 irAEs; | Updated the content of "special interest event" related to the sponsor. |
| P92-93 | 9.3. Symptomatic Treatment for Famitinib-Related Adverse Reactions | **2) Hypertension**<br>Patients should be strictly screened according to blood pressure requirements in the inclusion and exclusion criteria prior to enrollment. Patients with hypertension can control the blood pressure by adjusting the dose of or adding new antihypertensive drugs before administering the | **2) Hypertension**<br>Patients should be strictly screened according to blood pressure requirements in the inclusion and exclusion criteria prior to enrollment. Patients with hypertension can control the blood pressure by adjusting the dose of or adding new antihypertensive drugs before administering the | Refined expressions to facilitate understanding and operation. |

| | |
|---|---|
| Protocol Title: An Open-Label, Multicenter Phase II Clinical Study of Anti-PD-1 Antibody SHR-1210 Combined with Famitinib Malate in Patients with Advanced Urinary System Tumors and Gynecological Tumors | |
| Protocol No., Version, and Date: SHR-1210-II-213, V2.0, 6 Aug., 2019 | |
| Previous Version: V1.1, 30 Sep., 2018 | |
| Revised Version: V2.0, 6 Aug., 2019 | |

| Page | Content | Before | After | Reason for Revision |
|---|---|---|---|---|
| | | investigational products. The blood pressure must be under 140/90 mmHg (average of 2 blood pressure measurements taken at least 24 h apart) ~~before enrollment~~.<br>Monitoring and handling of such hypertension: Blood pressure should be monitored at least 3 times a week during the first 2 cycles of treatment with the ~~target~~ drug.<br>**4) Proteinuria**<br>All subjects should be closely monitored for proteinuria throughout the entire treatment period, especially for those with a history of hypertension. For those with a urine protein result of ~~++ ~ +++~~ in 2 consecutive tests, a 24-h urine protein assay is required. For those with urine protein of ~~++++ or above~~, a 24-h urine protein assay is required. | investigational products. The blood pressure must be under 140/90 mmHg (average of 2 blood pressure measurements taken at least 24 h apart) **before the first dose**.<br>Monitoring and handling of such hypertension: Blood pressure should be monitored at least 3 times a week during the first 2 cycles of the **study** treatment.<br>**4) Proteinuria**<br>All subjects should be closely monitored for proteinuria throughout the entire treatment period, especially for those with a history of hypertension. For those with a urine protein result of **2+** in 2 consecutive tests, a 24-h urine protein assay is required. For those with urine protein **> 2+**, a 24-h urine protein assay is required. | |
| P101 | 11.5.9. Exploratory analysis | The proportion of PD-L1-positive cells in tumor tissue and circulating tumor cells, ~~tumor mutation burden (TMB), and mismatch repair (MMR) status in tumor tissue and/or peripheral blood (for endometrial cancer only)~~ will be evaluated to explore the relationship of PD-L1 expression and/or other biomarkers (~~such as TMB at baseline~~) with efficacy. | The proportion of PD-L1-positive cells in tumor tissue and circulating tumor cells, **proportion of subjects with dMMR or MSI-H (for endometrial cancer and ovarian cancer only), and proportion of abnormal FGFR2/3 (for urothelial carcinoma only)** will be evaluated to explore the relationship of PD-L1 expression and/or other biomarkers with efficacy **(such as ORR/PFS)**. | Revised based on adjusted biomarker assays. |
| P102 | 11.6. Interim Analysis | The first interim analysis will be conducted after the 2-cycle (6-week) observation (start after the first dose) for the first 12 subjects enrolled in the | The first interim analysis will be conducted after the 2-cycle (6-week) observation (start after the first dose) for the first 12 subjects **(receiving q3w administration)** enrolled in the study is completed. | Refined expressions to avoid ambiguity. |

| |
|---|
| Protocol Title: An Open-Label, Multicenter Phase II Clinical Study of Anti-PD-1 Antibody SHR-1210 Combined with Famitinib Malate in Patients with Advanced Urinary System Tumors and Gynecological Tumors |
| Protocol No., Version, and Date: SHR-1210-II-213, V2.0, 6 Aug., 2019 |
| Previous Version: V1.1, 30 Sep., 2018 |
| Revised Version: V2.0, 6 Aug., 2019 |

| Page | Content | Before | After | Reason for Revision |
|---|---|---|---|---|
| | | study is completed. The dose reduction of famitinib in enrolled subjects and the clinically significant toxicities in subjects who have underwent 2-cycle observation during the first 2 treatment cycles of the study will be analyzed: | The dose reduction of famitinib in enrolled subjects and the clinically significant toxicities in subjects who have underwent 2-cycle observation during the first 2 treatment cycles of the study will be analyzed: | |
| P102 | 11.6. Interim Analysis | ~~Subsequent~~ interim analyses will be performed after enrollment of 22 subjects for a certain tumor type is completed and at least one efficacy evaluation is completed after administration. The efficacy analysis will be based on response rates (including unconfirmed CR/PR). If tumor response (confirmed or unconfirmed) is observed in ≥ 3 of 22 subjects, enrollment of this tumor type will be continued until 33-53 subjects are enrolled. | **A similar analysis will be performed for the 12 subjects receiving q2w administration (in a subsequent interim analysis).** **In addition, other** interim analyses will be performed after enrollment of 22 subjects for a certain tumor type is completed and at least one efficacy evaluation is completed after administration. The efficacy analysis will be based on response rates (including unconfirmed CR/PR). If tumor response (confirmed or unconfirmed) is observed in ≥ 3 of 22 subjects, enrollment of this tumor type will be continued until 33-53 subjects are enrolled. | Revised based on the added tolerability study of SHR-1210 q2w combined with famitinib. |
| P109 | 18. References | / | **Added:** 4. Y. Lin and W.J. Shih (2004), Adaptive Two-Stage Designs for Single-Arm Phase IIA Cancer Clinical Trials, Biometrics 60: 482-490. | Added references. |
| Full Text | Full text | "Interruption", "Discontinuation" | "Discontinuation" | Clerical error. |
| Full Text | Full text | "Patient" | "Subject" | Clerical error. |

| |
|---|
| Protocol Title: An Open-Label, Multicenter Phase II Clinical Study of Anti-PD-1 Antibody SHR-1210 Combined with Famitinib Malate in Patients with Advanced Urinary System Tumors and Gynecological Tumors |
| Protocol No., Version, and Date: SHR-1210-II-213, V2.1, 8 Oct., 2019 |
| Previous Version: V2.0, 6 Aug., 2019 |
| Revised Version: V2.1, 8 Oct., 2019 |

| Page | Content | Before | After | Reason for Revision |
|---|---|---|---|---|
| Cover Page and Full Text | Cover and header (all pages) | 1. Version no.: 2.0;<br>2. Version date: 6 Aug., 2019; | 1. Version no.: 2.1;<br>2. Version date: 8 Oct., 2019; | Updated the version number and version date. |
| Version History/ Revision History | Version history/revision history | None | Version 2.1 \| 8 Oct., 2019 \| 1. Revised the detection methods and sample collection for biomarkers MSI and FGFR2/3. 2. Revised wording. | Updated the version num ber and version date. |
| Sponsor's Signature Page | Sponsor's signature page | 1. Version no.: 2.0;<br>2. Version date: 6 Aug., 2019; | 1. Version no.: 2.1;<br>2. Version date: 8 Oct., 2019; | Updated the version number and version date. |
| P1 | Protocol synopsis | 1. Version no.: 2.0;<br>2. Version date: 6 Aug., 2019; | 1. Version no.: 2.1;<br>2. Version date: 8 Oct., 2018; | Updated the version number and version date. |
| P5 | Protocol synopsis | Tumor tissue samples, in sections or paraffin blocks, after the last treatment prior to this study for biomarker detection will be acquired/collected from subjects at screening. Tests include but are not limited to the following: proportion of PD-L1-positive cells in tumor tissue, MMR (for endometrial cancer and ovarian cancer only), and FGFR2/3 mutation (for urothelial carcinoma only). | Tumor tissue samples, in sections or paraffin blocks, after the last treatment prior to this study, **as well as 5 mL of whole blood** for biomarker detection will be acquired/collected from subjects at screening. Tests include but are not limited to the following: proportion of PD-L1-positive cells in tumor tissue, MMR (for endometrial cancer and ovarian cancer only), and FGFR2/3 mutation (for urothelial carcinoma only). | Refined the description of samples for biomarker detection. |

| |
|---|
| Protocol Title: An Open-Label, Multicenter Phase II Clinical Study of Anti-PD-1 Antibody SHR-1210 Combined with Famitinib Malate in Patients with Advanced Urinary System Tumors and Gynecological Tumors |
| Protocol No., Version, and Date: SHR-1210-II-213, V2.1, 8 Oct., 2019 |
| Previous Version: V2.0, 6 Aug., 2019 |
| Revised Version: V2.1, 8 Oct., 2019 |

| Page | Content | Before | After | Reason for Revision |
|---|---|---|---|---|
| P20<br>P27 | Notes | [26] For endometrial cancer and ovarian cancer, no less than 5 additional sections with a thickness of 4-5 μm should be collected for the detection of dMMR. For urothelial carcinoma, no less than 5 additional sections with a thickness of 4-5 μm should be collected for the detection of FGFR2/3 gene abnormality. Refer to the laboratory manual for tumor biosample acquisition/collection and processing methods. | [26] For endometrial cancer and ovarian cancer, no less than 5 additional sections with a thickness of 4-5 μm and **5 mL of whole blood should be collected for the detection of MSI**. For urothelial carcinoma, no less than 5 additional sections with a thickness of 4-5 μm should be collected for the detection of FGFR2/3 gene abnormality. Refer to the laboratory manual for tumor biosample acquisition/collection, **transportation,** and processing methods. | Refined the description of samples for biomarker detection. |
| P46 | Study design | Existing paraffin-embedded tumor tissue sections will be acquired/collected during the screening period. Prior to the first dose, it is also recommended to collect fresh biopsy specimens (core needle biopsy) and prepare 3 tumor sections with a thickness of 4-5 μm after fixation and embedding for biomarker detection | Existing paraffin-embedded tumor tissue sections will be acquired/collected during the screening period. Prior to the first dose, it is also recommended to collect fresh biopsy specimens (core needle biopsy), prepare 3 tumor sections with a thickness of 4-5 μm after fixation and embedding**, and collect 5 mL of whole blood** for biomarker detection | Refined the description of samples for biomarker detection. |
| P48-49,<br>P67 | 2.3.Acquisition, Collection, and Processing of Biomarkers | 3) For endometrial cancer and ovarian cancer, no less than 5 additional sections with a thickness of 4-5 μm should be collected for the detection of dMMR;<br>4) For urothelial carcinoma, no less than 5 additional sections with a thickness of 4-5 μm should be collected for the detection of FGFR2/3 gene abnormality. | 3) For endometrial cancer and ovarian cancer, no less than 5 **additional** sections with a thickness of 4-5 μm **and 5 mL of whole blood should be collected** for the detection of **MSI**;<br>4) For urothelial carcinoma, no less than 5 additional sections with a thickness of 4-5 μm should be collected for the detection of FGFR2/3 gene abnormality. | Refined the description of samples for biomarker detection. |

| |
|---|
| Protocol Title: An Open-Label, Multicenter Phase II Clinical Study of Anti-PD-1 Antibody SHR-1210 Combined with Famitinib Malate in Patients with Advanced Urinary System Tumors and Gynecological Tumors |
| Protocol No., Version, and Date: SHR-1210-II-213, V2.1, 8 Oct., 2019 |
| Previous Version: V2.0, 6 Aug., 2019 |
| Revised Version: V2.1, 8 Oct., 2019 |

<table>
<tr><th>Page</th><th>Content</th><th>Before</th><th>After</th><th>Reason for Revision</th></tr>
<tr><td></td><td></td><td>
**Table 4. Acquisition and collection of biomarker samples.**
<table>
<tr><th>Sample Type</th><th>Collection Requirement</th></tr>
<tr><td colspan="2">Screening Period</td></tr>
<tr><td>Previously archived samples</td><td>5 unstained sections with a thickness of 4-5 μm</td></tr>
<tr><td>Tumor biopsy samples recommended (before the first dose)</td><td>After fixation and embedding, 3 unstained sections with a thickness of 4-5 μm</td></tr>
<tr><td colspan="2">Collection of Additional Tumor Tissue Samples for Specific Tumor Types</td></tr>
<tr><td>For ovarian cancer, endometrial cancer, or urothelial carcinoma</td><td>No less than 5 additional unstained sections with a thickness of 4-5 μm</td></tr>
</table>
The acquired/collected tumor tissue samples will be transported to the designated central laboratory at room temperature. No processing by the study site is required.
</td><td>
**Table 4. Acquisition and collection of biomarker samples.**
<table>
<tr><th>Sample Type</th><th>Collection Requirement</th></tr>
<tr><td colspan="2">Screening Period</td></tr>
<tr><td>Previously archived samples</td><td>5 unstained sections with a thickness of 4-5 μm</td></tr>
<tr><td>Tumor biopsy samples recommended (before the first dose)</td><td>After fixation and embedding, 3 unstained sections with a thickness of 4-5 μm</td></tr>
<tr><td colspan="2">Collection of Additional Tumor Tissue Samples for Specific Tumor Types</td></tr>
<tr><td>For ovarian cancer, endometrial cancer, or urothelial carcinoma</td><td>No less than 5 additional unstained sections with a thickness of 4-5 μm</td></tr>
<tr><td>Collection of Additional Tumor Blood Samples for Specific Tumor Types</td><td></td></tr>
<tr><td>For ovarian cancer and endometrial cancer</td><td>5 mL of whole blood</td></tr>
</table>
**~~The acquired/collected tumor tissue samples will be transported to the designated central laboratory at room temperature. No processing by the study site is required.~~**
</td><td></td></tr>
<tr><td>P77</td><td>6.4. Biomarker Evaluation</td><td>The expression level of PD-L1 in tumor tissue will be assessed by immunohistochemistry; the MMR of tumor tissue will be assessed by immunohistochemistry (for endometrial cancer and ovarian cancer only); the FGFR 2/3 mutation will be assessed by immunohistochemistry/next-generation sequencing (for urothelial carcinoma only);</td><td>The expression level of PD-L1 in tumor tissue will be assessed by immunohistochemistry; the **MSI** of tumor tissue will be assessed by **~~immunohistochemistry~~next-generation sequencing** (for endometrial cancer and ovarian cancer only); the FGFR2/3 mutation will be assessed by immunohistochemistry/next-generation sequencing (for urothelial carcinoma only);</td><td>Changed the testing method and corresponding parameter.</td></tr>
</table>

# Revision Record of Protocol SHR-1210-II-213

Jiangsu Hengrui Pharmaceuticals Co., Ltd.

| |
|---|
| Protocol Title: An Open-Label, Multicenter Phase II Clinical Study of Anti-PD-1 Antibody SHR-1210 Combined with Famitinib Malate in Patients with Advanced Urinary System Tumors and Gynecological Tumors |
| Protocol No., Version, and Date: SHR-1210-II-213, V3.0, 8 Jul., 2020 |
| Previous Version: V2.1, 8 Oct., 2019 |
| Revised Version: V3.0, 8 Jul., 2020 |

| Page | Content | Before | After | Reason for Revision |
|---|---|---|---|---|
| Cover Page and Full Text | Cover and header<br>Sponsor's signature page<br>Protocol synopsis, P1 | 1. Version no.: 2.1;<br>2. Version date: 8 Oct., 2019; | 1. Version no.: 3.0;<br>2. Version date: 8 Jul., 2020; | Updated the version number and version date. |
| Version History/ Revision History | Version history/revision history | None | Version 3.0 \| 8 Jul., 2020 \| 1. Added Cohorts 6 and 7 to evaluate the efficacy of SHR-1210 monotherapy in urothelial carcinoma and famitinib monotherapy in cervical cancer and urothelial carcinoma.<br>2. Revised wording.<br>3. Deleted the content related to SHR-1210 q2w administration. | Updated the version number and version date. |
| P1, P46 | Primary objectives | None | • To evaluate the efficacy of SHR-1210 monotherapy in subjects with urothelial carcinoma.<br>• To evaluate the efficacy of famitinib monotherapy in subjects with urothelial carcinoma and cervical cancer. | Made changes corresponding to the addition of Cohorts 6 and 7 as recommended by the CDE. |

| |
|---|
| Protocol Title: An Open-Label, Multicenter Phase II Clinical Study of Anti-PD-1 Antibody SHR-1210 Combined with Famitinib Malate in Patients with Advanced Urinary System Tumors and Gynecological Tumors |
| Protocol No., Version, and Date: SHR-1210-II-213, V3.0, 8 Jul., 2020 |
| Previous Version: V2.1, 8 Oct., 2019 |
| Revised Version: V3.0, 8 Jul., 2020 |

| Page | Content | Before | After | Reason for Revision |
|---|---|---|---|---|
| P1-2, P47 | Secondary objectives | • To determine the PK of SHR-1210 combined with famitinib in subjects with various advanced tumors.<br>• To investigate the anti-SHR-1210 antibodies (ADAs) in subjects. | • To evaluate the safety and tolerability of SHR-1210 monotherapy in subjects with urothelial carcinoma.<br>• To evaluate the safety and tolerability of famitinib monotherapy in subjects with urothelial carcinoma and cervical cancer.<br>• To evaluate the PK of SHR-1210 combined with famitinib in subjects with various advanced solid tumors and famitinib monotherapy in subjects with urothelial carcinoma and cervical cancer.<br>• To investigate the anti-SHR-1210 antibodies (ADAs) in subjects receiving combination therapy. | Made changes corresponding to the addition of Cohorts 6 and 7 based on the communication with the CDE. |
| P2-P3, P47-P48 | Secondary endpoints | Pharmacokinetics:<br>• Plasma concentrations and PK parameters of famitinib and its main metabolites, including $C_{max}$, $T_{max}$, $AUC_{0-24\,h}$, CL/F, and V/F.<br>• Blood concentrations of anti-PD-1 antibody SHR- 1210. | Pharmacokinetics:<br>• Plasma concentrations and PK parameters (if applicable) of famitinib and its main metabolites for combination therapy and monotherapy, including $C_{max}$, $T_{max}$, $AUC_{0-24\,h}$, CL/F, and V/F.<br>• Blood concentrations of anti-PD-1 antibody SHR-1210 during combination therapy. | Made changes corresponding to the addition of Cohorts 6 and 7 based on the communication with the CDE. |
| P3, P48 | Secondary endpoints | Others:<br>• Proportion of subjects with anti-SHR-1210 antibodies (ADAs). | Others:<br>• Proportion of subjects with anti-SHR-1210 antibodies (ADAs) during combination therapy. | Made changes corresponding to the addition of Cohorts 6 and 7 based on the communication with the CDE. |

| |
|---|
| Protocol Title: An Open-Label, Multicenter Phase II Clinical Study of Anti-PD-1 Antibody SHR-1210 Combined with Famitinib Malate in Patients with Advanced Urinary System Tumors and Gynecological Tumors |
| Protocol No., Version, and Date: SHR-1210-II-213, V3.0, 8 Jul., 2020 |
| Previous Version: V2.1, 8 Oct., 2019 |
| Revised Version: V3.0, 8 Jul., 2020 |

| Page | Content | Before | After | Reason for Revision |
|---|---|---|---|---|
| P3, P48 | Exploratory endpoints | The proportion of PD-L1-positive cells in tumor tissue, proportion of subjects with dMMR or MSI-H (for endometrial cancer and ovarian cancer only), and proportion of abnormal FGFR2/3 gene (for urothelial carcinoma only) will be evaluated. | The proportion of PD-L1-positive cells in tumor tissue (for subjects receiving SHR-1210 combination therapy or monotherapy only), proportion of subjects with dMMR or MSI-H (for endometrial cancer and ovarian cancer only), and proportion of abnormal FGFR2/3 gene (for urothelial carcinoma treated with combination therapy only) will be evaluated to explore the relationship of PD-L1 expression and/or other biomarkers with efficacy (such as ORR/PFS). | Made changes corresponding to the addition of Cohorts 6 and 7 based on the communication with the CDE. |
| P3, P48 | Study design | Omitted | Revised the design of V2.1 corresponding to the addition of Cohorts 6 and 7. | Made changes corresponding to the addition of Cohorts 6 and 7 based on the communication with the CDE. |

| |
|---|
| Protocol Title: An Open-Label, Multicenter Phase II Clinical Study of Anti-PD-1 Antibody SHR-1210 Combined with Famitinib Malate in Patients with Advanced Urinary System Tumors and Gynecological Tumors |
| Protocol No., Version, and Date: SHR-1210-II-213, V3.0, 8 Jul., 2020 |
| Previous Version: V2.1, 8 Oct., 2019 |
| Revised Version: V3.0, 8 Jul., 2020 |

| Page | Content | Before | After | Reason for Revision |
|---|---|---|---|---|
| P6-P8 | Dosing regimen, clinically significant toxicity, as well as PK and ADA blood sampling | None | (for Cohorts 1-5) or 25 mg (for Cohort 7).<br>Subjects in Cohort 7 who participate in the blood sampling for famitinib PK analysis should take famitinib after blood sampling before C2D1 and C3D1 administration.<br>For famitinib PK analysis, 3 mL of blood sample will be collected (only at sites with conditions for PK blood sampling and processing) from subjects of Cohort 7 at each of the following time points: at 6 (± 1) h post- administration on C1D1, within 30 min pre- administration and at 6 (± 1) h post-administration on C2D1 and C3D1, and the plasma will be separated. If famitinib administration is interrupted on the day of sampling, pre-administration sampling should be continued but post-administration sampling should be skipped. On the sampling day, it is also necessary to record the previous administration time. | Made changes corresponding to the addition of Cohorts 6 and 7 based on the communication with the CDE. |
| P8, P54 | Inclusion criteria | Male or female aged between 18-70 years (for endometrial cancer cohort: 18-75 years old); | Male or female aged 18-75 years old; | Revised based on literature and clinical practice. |

| |
|---|
| Protocol Title: An Open-Label, Multicenter Phase II Clinical Study of Anti-PD-1 Antibody SHR-1210 Combined with Famitinib Malate in Patients with Advanced Urinary System Tumors and Gynecological Tumors |
| Protocol No., Version, and Date: SHR-1210-II-213, V3.0, 8 Jul., 2020 |
| Previous Version: V2.1, 8 Oct., 2019 |
| Revised Version: V3.0, 8 Jul., 2020 |

| Page | Content | Before | After | Reason for Revision |
|---|---|---|---|---|
| P8, P54 | Inclusion criteria | None | Cohorts 6 and 7: Have received at least one platinum-based therapy in the recurrence/metastasis stage, and the disease progressed or recurred. | Made changes corresponding to the addition of Cohorts 6 and 7 based on the communication with the CDE. |
| P11, P57 | Exclusion criteria | 13.The completion (last dose) of prior chemotherapy or surgery was less than 4 weeks from study medication; the completion of prior palliative radiotherapy was less than 2 weeks from study medication; | 13. The completion (last dose) of prior chemotherapy was less than 4 weeks from study medication; the completion of prior surgery or palliative radiotherapy was less than 2 weeks from study medication; | Revised based on project experience and clinical practice |
| P13-P14, P103 | Sample size determination | None | ➢ **Cohorts 6-7:**<br>The Simon's (minimax) two-stage design is adopted to calculate the sample size for each tumor type of Cohorts 6 and 7 based on one-sided alpha = 0.1 and power = 0.7: | Made changes corresponding to the addition of Cohorts 6 and 7 based on the communication with the CDE. |

| Protocol Title: An Open-Label, Multicenter Phase II Clinical Study of Anti-PD-1 Antibody SHR-1210 Combined with Famitinib Malate in Patients with Advanced Urinary System Tumors and Gynecological Tumors |
|---|
| Protocol No., Version, and Date: SHR-1210-II-213, V3.0, 8 Jul., 2020 |
| Previous Version: V2.1, 8 Oct., 2019 |
| Revised Version: V3.0, 8 Jul., 2020 |

| Page | Content | Before | After | Reason for Revision |
|---|---|---|---|---|
| | | | **Cohorts 6-7: Calculation of sample size by Simon's (minimax) two-stage method (one-sided alpha = 0.1, power = 0.7)** | |

| Cohort and Tumor Type | $P_0$ | $P_1$ | Stage I ($r_1/n_1$) | Stage II (r/n) |
|---|---|---|---|---|
| **Cohort 6** Urothelial Carcinoma - SHR-1210 Monotherapy Group | 0.10 | 0.25 | 1/14 | 4/23 |
| **Cohort 7** Urothelial Carcinoma - Famitinib Monotherapy Group | 0.10 | 0.25 | 1/14 | 4/23 |
| **Cohort 7** Cervical Cancer - Famitinib Monotherapy Group | 0.10 | 0.25 | 1/14 | 4/23 |

Note: $P_0$ is the maximum futility boundary, and $P_1$ is the minimum efficacy boundary. $n_1$ is the sample size of Stage I, n is the total sample size of the two stages, $r_1$ is the critical value of CR or PR that needs to be observed in Stage I (not inclusive), and r is the critical value of CR or PR that needs to be observed in the two stages (not inclusive). If, in Stage I, less than or equal to $r_1$ subjects among the $n_1$ subjects achieve CR or PR, the cohort will be terminated; otherwise n - $n_1$ subjects will be additionally enrolled in Stage II.

| |
|---|
| Protocol Title: An Open-Label, Multicenter Phase II Clinical Study of Anti-PD-1 Antibody SHR-1210 Combined with Famitinib Malate in Patients with Advanced Urinary System Tumors and Gynecological Tumors |
| Protocol No., Version, and Date: SHR-1210-II-213, V3.0, 8 Jul., 2020 |
| Previous Version: V2.1, 8 Oct., 2019 |
| Revised Version: V3.0, 8 Jul., 2020 |

| Page | Content | Before | After | Reason for Revision |
|---|---|---|---|---|
| P15-P16<br>P107<br>P108 | Data analysis/ statistical methods;<br>PK analysis;<br>Exploratory analysis | Omitted | Made revisions corresponding to the addition of Cohorts 6 and 7 and revised wording. | Made changes corresponding to the addition of Cohorts 6 and 7 based on the communication with the CDE.<br>Revised wording. |
| P16<br>P116 | Study dates;<br>Clinical study progress | Anticipated enrollment of the last subject: Dec. 2019<br>Anticipated study completion: Jun. 2020 | Anticipated enrollment of the last subject: Dec. 2020<br>Anticipated study completion: Jun. 2021 | Made changes corresponding to the addition of Cohorts 6 and 7 based on the communication with the CDE. |

| |
|---|
| Protocol Title: An Open-Label, Multicenter Phase II Clinical Study of Anti-PD-1 Antibody SHR-1210 Combined with Famitinib Malate in Patients with Advanced Urinary System Tumors and Gynecological Tumors |
| Protocol No., Version, and Date: SHR-1210-II-213, V3.0, 8 Jul., 2020 |
| Previous Version: V2.1, 8 Oct., 2019 |
| Revised Version: V3.0, 8 Jul., 2020 |

| Page | Content | Before | After | Reason for Revision |
|---|---|---|---|---|
| P22 | Note [28] in the Schedule of Activities | For famitinib PK analysis, 3 mL of blood sample will be collected on D1 (± 3 d) of Cycle 3 at each of the following time points: within 30 min pre-administration, and at 2 h ± 5 min, 4 h ± 5 min, 6 h ± 5 min, 8 h ± 5 min, 10 h ± 5 min, and 24 h ± 30 min post-administration (before D2 administration), and the plasma will be separated. | For famitinib PK analysis, 3 mL of blood sample will be collected from the first 12 subjects enrolled in Cohorts 1-5 on D1 (± 3 d) of Cycle 3 at each of the following time points: within 30 min pre-administration, and at 2 h ± 5 min, 4 h ± 5 min, 6 h ± 5 min, 8 h ± 5 min, 10 h ± 5 min, and 24 h ± 30 min post-administration (before D2 administration), and the plasma will be separated. For famitinib PK analysis, 3 mL of blood sample will be collected (only at sites with conditions for PK blood sampling and processing) from subjects of Cohort 7 at each of the following time points: at 6 (± 1) h post-administration on C1D1, within 30 min pre-administration and at 6 (± 1) h post-administration on C2D1 and C3D1, and the plasma will be separated. If famitinib administration is interrupted on the day of sampling, pre-administration sampling should be continued but post-administration sampling should be skipped. On the sampling day, it is also necessary to record the previous administration time. | Made changes corresponding to the addition of Cohorts 6 and 7 based on the communication with the CDE. |

| |
|---|
| Protocol Title: An Open-Label, Multicenter Phase II Clinical Study of Anti-PD-1 Antibody SHR-1210 Combined with Famitinib Malate in Patients with Advanced Urinary System Tumors and Gynecological Tumors |
| Protocol No., Version, and Date: SHR-1210-II-213, V3.0, 8 Jul., 2020 |
| Previous Version: V2.1, 8 Oct., 2019 |
| Revised Version: V3.0, 8 Jul., 2020 |

<table>
<tr><th>Page</th><th>Content</th><th>Before</th><th>After</th><th>Reason for Revision</th></tr>
<tr><td>P23</td><td>Note [29] in the Schedule of Activities</td><td>None</td><td>Additional eligible subjects with urothelial carcinoma (subjects not enrolled in Cohort 2) will be randomized to Cohort 6 or 7 to receive the corresponding investigational products after screening.</td><td>Made changes corresponding to the addition of Cohorts 6 and 7 based on the communication with the CDE.</td></tr>
<tr><td>P24</td><td>Schedule of sampling from subjects in Cohort 7 for famitinib PK analysis</td><td>None</td><td>Schedule of sampling from subjects in Cohort 7 for famitinib PK analysis
<table>
<tr><th>C1D1</th><th>C2D1</th><th>C3D1</th></tr>
<tr><td>——</td><td>Within 30 min pre-administration</td><td>Within 30 min pre-administration</td></tr>
<tr><td>6 (± 1) h post-administration</td><td>6 (± 1) h post-administration</td><td>6 (± 1) h post-administration</td></tr>
</table>
3 mL of blood will be collected at each sampling time point, and the plasma will be separated. If famitinib administration is interrupted on the day of sampling, pre- administration sampling should be continued but post- administration sampling should be skipped. On the sampling day, it is also necessary to record the previous administration time.</td><td>Made changes corresponding to the addition of Cohorts 6 and 7 based on the communication with the CDE.</td></tr>
</table>

| |
|---|
| Protocol Title: An Open-Label, Multicenter Phase II Clinical Study of Anti-PD-1 Antibody SHR-1210 Combined with Famitinib Malate in Patients with Advanced Urinary System Tumors and Gynecological Tumors |
| Protocol No., Version, and Date: SHR-1210-II-213, V3.0, 8 Jul., 2020 |
| Previous Version: V2.1, 8 Oct., 2019 |
| Revised Version: V3.0, 8 Jul., 2020 |

| Page | Content | Before | After | Reason for Revision |
|---|---|---|---|---|
| P42 | Study rationale | None | Anti-PD-1 monoclonal antibody monotherapy is evidently effective as the first-line treatment for cisplatin-intolerant, PD-L1-positive (CPS $\geq$ 10), or platinum-intolerant urothelial carcinoma, and as the second-line treatment for urothelial carcinoma that has progressed during or after prior platinum- based therapy, but no study has been conducted to explore the efficacy of SHR-1210 monotherapy in the treatment of urothelial carcinoma. Multiple studies have shown that targeted anti-angiogenic agents, whether in monotherapy such as cabozantinib or in combination therapy such as ramucirumab combined with paclitaxel, have certain efficacy in the treatment of urothelial carcinoma[4-6], but the efficacy of famitinib in urothelial carcinoma has not been explored in any study. The OS and ORR of bevacizumab combined with chemotherapy as the first-line treatment for recurrent or metastatic cervical cancer were significantly improved compared with chemotherapy alone[7]. Accordingly, the NCCN guidelines recommend this therapy as the first- line treatment for recurrent or metastatic cervical cancer. In addition, bevacizumab monotherapy is also recommended by the NCCN guidelines as the second-line treatment for recurrent or metastatic cervical cancer. | Made changes corresponding to the addition of Cohorts 6 and 7 based on the communication with the CDE. |

| |
|---|
| Protocol Title: An Open-Label, Multicenter Phase II Clinical Study of Anti-PD-1 Antibody SHR-1210 Combined with Famitinib Malate in Patients with Advanced Urinary System Tumors and Gynecological Tumors |
| Protocol No., Version, and Date: SHR-1210-II-213, V3.0, 8 Jul., 2020 |
| Previous Version: V2.1, 8 Oct., 2019 |
| Revised Version: V3.0, 8 Jul., 2020 |

| Page | Content | Before | After | Reason for Revision |
|---|---|---|---|---|
| | | | Therefore, in order to investigate the contribution of each drug in the combination therapy regimen of SHR-1210 and famitinib, an SHR-1210 monotherapy group and<br><br>a famitinib monotherapy group will be added for exploration in urothelial carcinoma and cervical cancer. | |
| P44 | Rationale for dosing regimen design | None | In order to investigate the contribution of each drug in the combination therapy regimen, an SHR-1210 monotherapy group and a famitinib monotherapy group will be added. The 200 mg q3w dose is selected for the SHR-1210 monotherapy group based on the results of the phase II study of SHR-1210 monotherapy in advanced HCC. Patients with metastatic urothelial carcinoma will be enrolled to investigate the efficacy and safety of SHR-1210 monotherapy in urothelial carcinoma. The recommended phase II dose of famitinib monotherapy, 25 mg qd, is selected for the famitinib monotherapy group. Patients with metastatic urothelial carcinoma or recurrent/metastatic cervical cancer will be enrolled to investigate the efficacy and safety of famitinib monotherapy in corresponding tumor types. | Made changes corresponding to the addition of Cohorts 6 and 7 based on the communication with the CDE. |

| |
|---|
| Protocol Title: An Open-Label, Multicenter Phase II Clinical Study of Anti-PD-1 Antibody SHR-1210 Combined with Famitinib Malate in Patients with Advanced Urinary System Tumors and Gynecological Tumors |
| Protocol No., Version, and Date: SHR-1210-II-213, V3.0, 8 Jul., 2020 |
| Previous Version: V2.1, 8 Oct., 2019 |
| Revised Version: V3.0, 8 Jul., 2020 |

| Page | Content | Before | After | Reason for Revision |
|---|---|---|---|---|
| P51 | Randomization | None | 2.1.3 Randomization<br>This is an open-label, multicenter phase II clinical study. Only subjects with urothelial carcinoma for Cohorts 6 and 7 will be randomized. After the completion of enrollment of Cohort 2, eligible subjects with urothelial carcinoma will be assigned at a 1:1 ratio to Cohort 6 or 7 to receive SHR-1210 or famitinib monotherapy, with no stratification factor.<br>For Cohorts 6 and 7, randomization will be performed using an interactive web response system (IWRS). Subjects participating in this study will be assigned subject ID as the unique identification code. Eligible subjects will be assigned a randomization number and a cohort by the IWRS to receive treatment corresponding to the assigned cohort. | Made changes corresponding to the addition of Cohorts 6 and 7 based on the communication with the CDE. |
| P52 | 2.2.1. Study procedure and arrangement of blood sampling points | None | ➢ Cohort 7<br>For famitinib PK analysis, 3 mL of blood sample will be collected (only at sites with conditions for PK blood sampling and processing) at each of the following time points:<br>at 6 (± 1) h post-administration on C1D1, within30 min pre- administration and at 6 (± 1) h post-administration on C2D1 and C3D1, and the plasma will be separated.<br>If famitinib administration is interrupted on the day of sampling, pre-administration sampling should be continued but post-administration sampling should be skipped. On the sampling day, it is also necessary to record the previous administration time. | Made changes corresponding to the addition of Cohorts 6 and 7 based on the communication with the CDE. |

| |
|---|
| Protocol Title: An Open-Label, Multicenter Phase II Clinical Study of Anti-PD-1 Antibody SHR-1210 Combined with Famitinib Malate in Patients with Advanced Urinary System Tumors and Gynecological Tumors |
| Protocol No., Version, and Date: SHR-1210-II-213, V3.0, 8 Jul., 2020 |
| Previous Version: V2.1, 8 Oct., 2019 |
| Revised Version: V3.0, 8 Jul., 2020 |

| Page | Content | Before | After | Reason for Revision |
|---|---|---|---|---|
| P52-P53 | 2.2.2. Processing and testing of blood samples | Blood sampling before and after the administration of SHR-1210: At each blood sampling point before administration, 2 tubes of 4 mL of whole blood will be collected into coagulation tubes, and the coagulation tubes should be inverted several times to mix the blood sample evenly. After being let stand at room temperature for 30 min to 1 h until blood agglutination, the blood sample should be centrifuged at 4 °C and 1500 g (centrifugal force) for 10 min; then, the supernatant in each tube of whole blood will be aliquoted into 2 tubes (not less than 500 µL per tube).<br>At each blood sampling point after administration, 1 tube of 4 mL of whole blood will be collected into a coagulation tube, and the coagulation tube should be inverted several times to mix the blood sample evenly. After being let stand at room temperature for 30 min to 1 h until blood agglutination, the blood sample should be centrifuged at 4 °C and 1500 g (centrifugal force) for 10 min; then, the supernatant will be aliquoted into 2 tubes (not less than 500 µL per tube). | Refer to the laboratory manual for details on blood sampling, processing, and transportation methods. | For convenience, follow the laboratory manual of the study. |

| Protocol Title: An Open-Label, Multicenter Phase II Clinical Study of Anti-PD-1 Antibody SHR-1210 Combined with Famitinib Malate in Patients with Advanced Urinary System Tumors and Gynecological Tumors |
|---|
| Protocol No., Version, and Date: SHR-1210-II-213, V3.0, 8 Jul., 2020 |
| Previous Version: V2.1, 8 Oct., 2019 |
| Revised Version: V3.0, 8 Jul., 2020 |

| Page | Content | Before | After | Reason for Revision |
|---|---|---|---|---|
| | | Blood sampling before and after the administration of famitinib: At each blood sampling point, 3 mL of whole blood will be collected into a heparin-containing anticoagulation tube, and the tube will be inverted several times to mix the blood with the anticoagulant. Within 2 h after sampling, the blood sample should be centrifuged at 4 °C and 1500 g (centrifugal force) for 10 min; then, the supernatant will be aliquoted into 2 tubes (not less than 500 µL per tube).<br>After the separated plasma/serum is well- marked, it should be temporarily stored in a freezer at -20 °C or below, or stored for a long time below -60 °C for future testing.<br>Each sample will be assigned with a unique sample number. The actual date and time of blood sampling as well as the exact time of drug administration should be recorded on the PK blood sampling page of the eCRF. Problems (such as hemolysis) encountered during blood sampling should also be noted in the eCRF. Refer to the laboratory manual for details on sample acquisition, processing, and transportation methods. | | |

| |
|---|
| Protocol Title: An Open-Label, Multicenter Phase II Clinical Study of Anti-PD-1 Antibody SHR-1210 Combined with Famitinib Malate in Patients with Advanced Urinary System Tumors and Gynecological Tumors |
| Protocol No., Version, and Date: SHR-1210-II-213, V3.0, 8 Jul., 2020 |
| Previous Version: V2.1, 8 Oct., 2019 |
| Revised Version: V3.0, 8 Jul., 2020 |

| Page | Content | Before | After | Reason for Revision |
|---|---|---|---|---|
| P61-P62 | Dosage form, appearance, packaging, and label | None | 25 mg/capsule | The newly added Cohort 7 uses famitinib with the strength of 25 mg. |
| P64 | Dose modification | None | Reduction to 20 mg/d and again to 15 mg/d from the starting dose of 25 mg/d | Due to the need for dose modification of famitinib monotherapy for Cohort 7. |
| P74 | Enrollment | None | Additional eligible subjects with urothelial carcinoma (subjects not enrolled in Cohort 2) will be randomized to Cohort 6 or 7 to receive the corresponding investigational products after screening. | Revised based on the randomization design for Cohorts 6 and 7. |
| P76 | Treatment period | None | ➢ Cohort 7:<br>For famitinib PK analysis, 3 mL of blood sample will be collected (only at sites with conditions for PK blood sampling and processing) at each of the following time points: at 6 (± 1) h post-administration on C1D1, within 30 min pre- administration and at 6 (± 1) h post-administration on C2D1 and C3D1, and the plasma will be separated. If famitinib administration is interrupted on the day of sampling, pre- administration sampling should be continued but post- administration sampling should be skipped. On the sampling day, it is also necessary to record the previous administration time. | Revised based on the PK design for Cohort 7. |

| Protocol Title: An Open-Label, Multicenter Phase II Clinical Study of Anti-PD-1 Antibody SHR-1210 Combined with Famitinib Malate in Patients with Advanced Urinary System Tumors and Gynecological Tumors |
|---|
| Protocol No., Version, and Date: SHR-1210-II-213, V3.0, 8 Jul., 2020 |
| Previous Version: V2.1, 8 Oct., 2019 |
| Revised Version: V3.0, 8 Jul., 2020 |

| Page | Content | Before | After | Reason for Revision |
|---|---|---|---|---|
| P77 | Follow-up period | SAEs and irAEs observed within 90 days after the last dose of SHR-1210 should be followed up. If subjects start new anti-tumor treatment, they should be followed up until they start the tumor treatment.<br>[Survival follow-up] After the last study dose, the survival status and subsequent anti-tumor treatment can be collected through clinical or telephone follow-up every 2 months (± 7 d) until death. | All AEs suspected to be related to SHR-1210 should be collected from 30 to 90 days after the last dose. If no new anti-tumor treatment starts during the period, all SAEs should be collected; if new anti-tumor treatment starts, only SAEs related to the investigational products will be collected after the start of the new anti-tumor treatment.<br>[Survival follow-up] After the end of treatment, the survival status and subsequent anti-tumor treatment can be collected through clinical or telephone follow-up every 2 months (± 7 d) until death. | Refined expressions for consistent texts and in favor of implementation. |
| P84 | Adverse event reporting | Omitted | Refer to the tracking version of the protocol for detailed changes. | Revised according to the new version of GCP (effective from 1 Jul., 2020) and the company's internal assessment, and revised wording. |
| P100-P102 | Statistical hypothesis and discriminatory rules | Omitted | Refer to the tracking version of the protocol for detailed changes. | Made changes corresponding to the addition of Cohorts 6 and 7 based on the communication with the CDE. |

| |
|---|
| Protocol Title: An Open-Label, Multicenter Phase II Clinical Study of Anti-PD-1 Antibody SHR-1210 Combined with Famitinib Malate in Patients with Advanced Urinary System Tumors and Gynecological Tumors |
| Protocol No., Version, and Date: SHR-1210-II-213, V3.0, 8 Jul., 2020 |
| Previous Version: V2.1, 8 Oct., 2019 |
| Revised Version: V3.0, 8 Jul., 2020 |

| Page | Content | Before | After | Reason for Revision |
|---|---|---|---|---|
| P104 | Statistical analysis plan | None | The study is also aimed at exploring the efficacy, safety, etc. of SHR-1210 monotherapy in subjects with urothelial carcinoma and famitinib monotherapy in subjects with urothelial carcinoma and cervical cancer. | Made changes corresponding to the addition of Cohorts 6 and 7 based on the communication with the CDE. |
| P104 | Analysis population | Safety set (SS): All enrolled subjects who have received at least one dose of the investigational products and have post-administration safety data. This analysis set will be used for safety analysis, with SS1 to SS5 corresponding to various cohorts. | • Evaluable set (ES): All enrolled subjects who have received at least one dose of the investigational products and have undergone at least one valid post-baseline imaging assessment. This analysis set will be used for the analysis of the primary efficacy endpoint, with ES1 to ES7 (including ES7-1, ES7-2, and ES7) corresponding to various cohorts (Cohorts 1-7). | Made changes corresponding to the addition of Cohorts 6 and 7 based on the communication with the CDE. |

| |
|---|
| Protocol Title: An Open-Label, Multicenter Phase II Clinical Study of Anti-PD-1 Antibody SHR-1210 Combined with Famitinib Malate in Patients with Advanced Urinary System Tumors and Gynecological Tumors |
| Protocol No., Version, and Date: SHR-1210-II-213, V3.0, 8 Jul., 2020 |
| Previous Version: V2.1, 8 Oct., 2019 |
| Revised Version: V3.0, 8 Jul., 2020 |

| Page | Content | Before | After | Reason for Revision |
|---|---|---|---|---|
| P104-P105 | Statistical methods | None | With regard to the efficacy analysis of this study, Cohorts 1-5 are analyzed and summarized on the combination therapy by tumor type, Cohort 6 is analyzed and summarized on SHR-1210 monotherapy for urothelial carcinoma, and Cohort 7 is analyzed and summarized on famitinib monotherapy for urothelial carcinoma, famitinib monotherapy for cervical cancer, and the both as a whole, respectively;<br>With regard to the safety analysis of this study, Cohorts 1-5 are analyzed and summarized on the combination therapy by tumor type and as a whole, Cohort 6 is analyzed and summarized on SHR-1210 monotherapy for urothelial carcinoma, and Cohort 7 is analyzed and summarized on famitinib monotherapy for urothelial carcinoma, famitinib monotherapy for cervical cancer, and the both as a whole, respectively. | Made changes corresponding to the addition of Cohorts 6 and 7 based on the communication with the CDE. |

| |
|---|
| Protocol Title: An Open-Label, Multicenter Phase II Clinical Study of Anti-PD-1 Antibody SHR-1210 Combined with Famitinib Malate in Patients with Advanced Urinary System Tumors and Gynecological Tumors |
| Protocol No., Version, and Date: SHR-1210-II-213, V3.0, 8 Jul., 2020 |
| Previous Version: V2.1, 8 Oct., 2019 |
| Revised Version: V3.0, 8 Jul., 2020 |

| Page | Content | Before | After | Reason for Revision |
|---|---|---|---|---|
| P109 | Interim analysis | None | For each tumor type in Cohorts 6 and 7, other interim analyses will be performed after enrollment of 14 subjects for a certain cohort is completed and at least one efficacy evaluation is completed after administration. The efficacy analysis will be based on response rates (including unconfirmed CR/PR). If tumor response (confirmed or unconfirmed) is observed in ≥ 2 of 14 subjects, enrollment of this cohort will be continued until 23 subjects are enrolled.<br>For the tumor type of a certain cohort where 23 subjects are enrolled and the last subject completes at least 2 efficacy evaluations after treatment observation and enrollment (or at least 18 weeks after the last subject's enrollment), the previously accumulated efficacy data will be analyzed. The efficacy analysis will be based on objective response rate (ORR). Further studies of this cohort will be determined based on efficacy results. | Made changes corresponding to the addition of Cohorts 6 and 7 based on the communication with the CDE. |

| |
|---|
| Protocol Title: An Open-Label, Multicenter Phase II Clinical Study of Anti-PD-1 Antibody SHR-1210 Combined with Famitinib Malate in Patients with Advanced Urinary System Tumors and Gynecological Tumors |
| Protocol No., Version, and Date: SHR-1210-II-213, V3.0, 8 Jul., 2020 |
| Previous Version: V2.1, 8 Oct., 2019 |
| Revised Version: V3.0, 8 Jul., 2020 |

| Page | Content | Before | After | Reason for Revision |
|---|---|---|---|---|
| Full Text | Study design, dosing regimen, definition of clinically significant toxicity, schedule of activities, dose level of famitinib combined with SHR-1210 q2w, overview of study design, packaging and labeling, dosing regimen, treatment period, and criteria for efficacy evaluation | Content related to the dose level of famitinib 20 mg, qd combined with SHR-1210 q2w | Deleted | Based on the interim study data previously obtained, after re- evaluation, the company decided not to conduct the exploration of the dose level of famitinib 20 mg, qd combined with SHR-1210 q2w. Thus, relevant content was deleted. |

| |
|---|
| Protocol Title: An Open-Label, Multicenter Phase II Clinical Study of Anti-PD-1 Antibody SHR-1210 Combined with Famitinib Malate in Patients with Advanced Urinary System Tumors and Gynecological Tumors |
| Protocol No., Version, and Date: SHR-1210-II-213, V4.0, 5 Nov., 2020 |
| Previous Version: V3.0, 8 Jul., 2020 |
| Revised Version: V4.0, 5 Nov., 2020 |

| Page | Content | Before | After | Reason for Revision |
|---|---|---|---|---|
| Cover Page and Full Text | Cover and header<br>Sponsor's signature page<br>Protocol synopsis, P1 | 1. Version no.: 3.0;<br>2. Version date: 8 Jul., 2020; | 1. Version no.: 4.0;<br>2. Version date: 5 Nov., 2020; | Updated the version number and version date. |
| Version History/ Revision History | Version history/revision history | None | Version 4.0 \| 5 Nov., 2020 \| 1. Added Cohort 8 to evaluate the efficacy of SHR-1210 combined with famitinib for the treatment of advanced renal cancer that has progressed after previous anti-PD-1 or PD-L1 antibody therapy;<br>2. Added the description that subjects in Cohorts 6 and 7 can continue treatment with SHR-1210 combined with famitinib after PD.<br>3. Revised logic | Updated the version number and version date. |
| P3, P40 | Study design | Omitted | Added design related to Cohort 8, as well as design related to continuing treatment after PD for Cohorts 6 and 7. | 1. Anti-PD-1 or PD-L1 antibodies combined with targeted anti-angiogenic drugs have been approved for the first-line treatment of advanced renal cancer, making it a clinical problem that needs to be solved urgently how to treat advanced renal |

| |
|---|
| Protocol Title: An Open-Label, Multicenter Phase II Clinical Study of Anti-PD-1 Antibody SHR-1210 Combined with Famitinib Malate in Patients with Advanced Urinary System Tumors and Gynecological Tumors |
| Protocol No., Version, and Date: SHR-1210-II-213, V4.0, 5 Nov., 2020 |
| Previous Version: V3.0, 8 Jul., 2020 |
| Revised Version: V4.0, 5 Nov., 2020 |

| Page | Content | Before | After | Reason for Revision |
|---|---|---|---|---|
| | | | | cancer that has progressed after anti-PD-1 or PD-L1 antibody therapy. The preliminary results of this study showed that SHR-1210 combined with famitinib had a significant effect in the treatment of advanced renal cancer. Therefore, a study of Cohort 8 was designed to explore the efficacy of this combination therapy for advanced renal cancer that has progressed after previous anti-PD-1 or PD-L1 antibody therapy;<br>2. Cohorts 6 and 7 will adopt SHR-1210 and famitinib monotherapy respectively. To ensure the maximum benefit of subjects enrolled in Cohorts 6 and 7, continuing treatment with SHR-1210 combined with famitinib after PD was added. |

| |
|---|
| Protocol Title: An Open-Label, Multicenter Phase II Clinical Study of Anti-PD-1 Antibody SHR-1210 Combined with Famitinib Malate in Patients with Advanced Urinary System Tumors and Gynecological Tumors |
| Protocol No., Version, and Date: SHR-1210-II-213, V4.0, 5 Nov., 2020 |
| Previous Version: V3.0, 8 Jul., 2020 |
| Revised Version: V4.0, 5 Nov., 2020 |

| Page | Content | Before | After | Reason for Revision |
|---|---|---|---|---|
| P6, P55 | Dosing Regimen | Famitinib malate capsules: administered orally once a day before or after a meal (recommended at a fixed time: within 0.5 h after a meal) at a dose of 20 mg (for Cohorts 1-5) or 25 mg (for Cohort 7). The drug should be administered continuously in cycles of 3 weeks. Subjects in Cohort 7 who participate in the blood sampling for famitinib PK analysis should take famitinib after blood sampling before C2D1 and C3D1 administration. | Famitinib malate capsules: administered orally once a day before or after a meal (recommended at a fixed time: within 0.5 h after a meal) at a dose of 20 mg (for subjects in Cohorts 1-5 and 8 and for subjects who continue treatment after PD in Cohort 6) or 25 mg (for Cohort 7). The drug should be administered continuously in cycles of 3 weeks. In Cohort 7, the dose and frequency of famitinib for subjects who continue treatment after PD are the same as those before PD. If the dose of famitinib before PD is 25 mg, the dose of famitinib should be adjusted to 20 mg (continuous administration) for continuing treatment. Subjects in Cohorts 7 and 8 who participate in the blood sampling for famitinib PK analysis should take famitinib after blood sampling before C2D1 and C3D1 administration. | Made revisions corresponding to the added design for Cohort 8 and for continuing treatment after PD in Cohorts 6 and 7. |
| P7, P43 | Blood Sampling for PK and ADA Analysis | ➢ **Cohort 7**:<br>Subjects in Cohort 7 will undergo blood sampling for famitinib PK analysis (only at sites with conditions for PK blood sampling and processing) at the following time points: | ➢ **Cohorts 7 and 8**:<br>Subjects in Cohorts 7 and 8 will undergo blood sampling for famitinib PK analysis (only at sites with conditions for PK blood sampling and processing) at the following time points: | Made revisions corresponding to the added design for Cohort 8. |

| |
|---|
| Protocol Title: An Open-Label, Multicenter Phase II Clinical Study of Anti-PD-1 Antibody SHR-1210 Combined with Famitinib Malate in Patients with Advanced Urinary System Tumors and Gynecological Tumors |
| Protocol No., Version, and Date: SHR-1210-II-213, V4.0, 5 Nov., 2020 |
| Previous Version: V3.0, 8 Jul., 2020 |
| Revised Version: V4.0, 5 Nov., 2020 |

| Page | Content | Before | After | Reason for Revision |
|---|---|---|---|---|
| P7, P45 | Inclusion criteria | Renal cell carcinoma: Histologically or cytologically confirmed advanced clear cell renal cell carcinoma (in the case of mixed tumors, predominant clear cell renal cell carcinoma is required) that has been previously treated with at most one targeted anti-angiogenic drug and failed the treatment (if any); | - Renal cell carcinoma: Histologically or cytologically confirmed advanced clear cell renal cell carcinoma (in the case of mixed tumors, predominant clear cell renal cell carcinoma is required);<br>1) Cohort 1: Have been previously treated with at most one targeted anti-angiogenic drug and failed the treatment (if any);<br>2) Cohort 8: Progression after prior anti-PD-1/PD-L1/CTLA-4 antibody monotherapy or combination therapy (at least 2 doses of anti-PD-1/PD-L1/CTLA-4 antibody); | Made revisions corresponding to the added design for Cohort 8. |
| P11, P48 | Exclusion criteria | 20. Prior treatment with anti-PD-1/PD-L1 antibodies or famitinib; | 20. Prior treatment with anti-PD-1/PD-L1 antibodies (except for cervical cancer in Cohort 7 and Cohort 8) or famitinib; | Revised based on reports from the studies of PD-1 or PD-L1 antibodies in cervical cancer and based on the design for Cohort 8. |
| P11, P52 | Criteria for discontinuation | 2. Imaging examinations show PD; as per RECIST v1.1, a confirmation is required 4-6 weeks after the first documentation of PD (except those with rapid progression or significant clinical progression);<br>3. Accumulated use of SHR-1210 for 2 years (no radiographic | 2. Imaging examinations show PD; as per RECIST v1.1, a confirmation is required 4-6 weeks after the first documentation of PD (except those with rapid progression, with significant clinical progression, or receiving famitinib monotherapy);<br>3. Accumulated use of SHR-1210 monotherapy or combination therapy for 2 years (no radiographic progression). Subjects who | There is no pseudoprogression in patients treated with famitinib monotherapy, and thus no confirmation after the first documentation of PD is required.<br>Revised logic. |

| |
|---|
| Protocol Title: An Open-Label, Multicenter Phase II Clinical Study of Anti-PD-1 Antibody SHR-1210 Combined with Famitinib Malate in Patients with Advanced Urinary System Tumors and Gynecological Tumors |
| Protocol No., Version, and Date: SHR-1210-II-213, V4.0, 5 Nov., 2020 |
| Previous Version: V3.0, 8 Jul., 2020 |
| Revised Version: V4.0, 5 Nov., 2020 |

<table>
<tr><th>Page</th><th>Content</th><th>Before</th><th>After</th><th>Reason for Revision</th></tr>
<tr><td></td><td></td><td>progression). Subjects who achieve radiographically confirmed CR may consider discontinuation after 12 cycles of treatment;</td><td>achieve radiographically confirmed CR may consider discontinuation after 12 cycles of treatment;</td><td></td></tr>
<tr><td>P13, P91</td><td>Sample size determination</td><td>None</td><td>Cohort 8:<br>The Simon's (minimax) two-stage design is adopted to calculate the sample size of Cohort 8 based on one-sided alpha = 0.025 and power = 0.8:<br>Cohort 8: Calculation of sample size by Simon's (minimax) two-stage method (one-sided alpha = 0.025, power = 0.8)<br><table><tr><th>Cohort and Tumor Type</th><th>$P_0$</th><th>$P_1$</th><th>Stage I ($r_1/n_1$)</th><th>Stage II (r/n)</th></tr><tr><td>Cohort 8 Renal Cell Carcinoma</td><td>0.30</td><td>0.50</td><td>6/21</td><td>20/47</td></tr></table>Note: P0 is the maximum futility boundary, and P1 is the minimum efficacy boundary. n1 is the sample size of Stage I, n is the total sample size of the two stages, r1 is the critical value of CR or PR that needs to be observed in Stage I (not inclusive), and r is the critical value of CR or PR that needs to be observed in the two stages (not inclusive). If, in Stage I, less than or equal to r1 subjects among the n1 subjects achieve CR or PR, the cohort will be terminated; otherwise n - n1</td><td>Added Cohort 8.</td></tr>
</table>

| |
|---|
| Protocol Title: An Open-Label, Multicenter Phase II Clinical Study of Anti-PD-1 Antibody SHR-1210 Combined with Famitinib Malate in Patients with Advanced Urinary System Tumors and Gynecological Tumors |
| Protocol No., Version, and Date: SHR-1210-II-213, V4.0, 5 Nov., 2020 |
| Previous Version: V3.0, 8 Jul., 2020 |
| Revised Version: V4.0, 5 Nov., 2020 |

| Page | Content | Before | After | Reason for Revision |
|---|---|---|---|---|
| | | | subjects will be additionally enrolled in Stage II. | |
| P16<br>P103 | Study dates;<br>Clinical study progress | Anticipated enrollment of the last subject: Dec. 2020<br>Anticipated study completion: Jun. 2021 | Anticipated enrollment of the last subject: Jun. 2021<br>Anticipated study completion: Dec. 2021 | Made revisions corresponding to the added design for Cohort 8 and for continuing treatment after PD in Cohorts 6 and 7. |
| P18 | Schedule of activities - notes | None | The safety and imaging visits for subjects who receive continuing treatment after PD in Cohorts 6 and 7 are the same as those before PD. | Made revisions corresponding to the design for continuing treatment after PD in Cohorts 6 and 7. |
| P20 | Note [18] in the Schedule of Activities | Blood pressure monitoring: The blood pressure of patients will be measured by the investigators or study nurse in the screening period. At each blood pressure measurement, smoking and coffee are prohibited within 30 min before measurement, and patients should rest for at least 10 min. The sitting position will be taken at measurement by placing the elbow at the same level as the heart. Each blood pressure measurement should be taken on the same side of the body; | Blood pressure monitoring: The blood pressure of patients will be measured by the investigators or study nurse in the screening period. At each blood pressure measurement, smoking and coffee are prohibited within 30 min before measurement, and patients should rest for at least 10 min. The sitting position will be taken at measurement by placing the elbow at the same level as the heart. Each blood pressure measurement should be taken on the same side of the body. The blood pressure must be under 140/90 mmHg (average of 2 blood pressure measurements taken at least 24 h apart) before the first dose. | Made revisions corresponding to "2) Hypertension" in "9.3 Symptomatic Treatment for Famitinib-Related Adverse Reactions". |
| P20 | Note [20] in the Schedule of Activities | SHR-1210 administration to subjects in Cohorts 1-6: | SHR-1210 administration to subjects in Cohorts 1-6 and 8 and to subjects who continue treatment after PD in Cohort 7: | Made revisions corresponding to the added design for Cohort 8 and for continuing treatment after PD in Cohorts 6 and 7. |

| |
|---|
| Protocol Title: An Open-Label, Multicenter Phase II Clinical Study of Anti-PD-1 Antibody SHR-1210 Combined with Famitinib Malate in Patients with Advanced Urinary System Tumors and Gynecological Tumors |
| Protocol No., Version, and Date: SHR-1210-II-213, V4.0, 5 Nov., 2020 |
| Previous Version: V3.0, 8 Jul., 2020 |
| Revised Version: V4.0, 5 Nov., 2020 |

| Page | Content | Before | After | Reason for Revision |
|---|---|---|---|---|
| P20 | Note [21] in the Schedule of Activities | Famitinib should be administered orally once a day before or after a meal (recommended at a fixed time: within 0.5 h after a meal) at a dose of 20 mg (for Cohorts 1-5) or 25 mg (for Cohort 7). The drug should be administered continuously in cycles of 3 weeks until PD, unacceptable toxicity, or withdrawal from the treatment by the subjects or the investigators. | Famitinib should be administered orally once a day before or after a meal (recommended at a fixed time: within 0.5 h after a meal) at a dose of 20 mg (for subjects in Cohorts 1-5 and 8 and for subjects who continue treatment after PD in Cohort 6) or 25 mg (for Cohort 7). The drug should be administered continuously in cycles of 3 weeks. In Cohort 7, the dose and frequency of famitinib for subjects who continue treatment after PD are the same as those before PD. If the dose of famitinib before PD is 25 mg, the dose of famitinib should be adjusted to 20 mg for continuing treatment. The drug should be administered until PD, unacceptable toxicity, or withdrawal from the treatment by the subjects or the investigators. Subjects in Cohorts 7 and 8 who participate in the blood sampling for famitinib PK analysis should take famitinib after blood sampling before C2D1 and C3D1 administration. | Made revisions corresponding to the added design for Cohort 8 and for continuing treatment after PD in Cohorts 6 and 7. |
| P34 | Study rationale | None | In recent years, remarkable results of studies on anti-PD-1 or PD-L1 antibodies combined with targeted anti-angiogenic drugs as the first-line treatment for advanced renal cancer have been achieved. The study results of pembrolizumab combined with axitinib as the first-line treatment for advanced renal cancer (Keynote-426) showed that, compared with the sunitinib control group, | Added study rationale for the added Cohort 8. |

| |
|---|
| Protocol Title: An Open-Label, Multicenter Phase II Clinical Study of Anti-PD-1 Antibody SHR-1210 Combined with Famitinib Malate in Patients with Advanced Urinary System Tumors and Gynecological Tumors |
| Protocol No., Version, and Date: SHR-1210-II-213, V4.0, 5 Nov., 2020 |
| Previous Version: V3.0, 8 Jul., 2020 |
| Revised Version: V4.0, 5 Nov., 2020 |

| Page | Content | Before | After | Reason for Revision |
|---|---|---|---|---|
| | | | objective response rate (ORR), progression-free survival (PFS), and overall survival (OS) were significantly improved in the combination therapy group. For the first time, the ORR of first-line treatment for advanced renal cancer was increased from 25%-35% in the era of targeted anti-angiogenic therapy to 50%-60%, mPFS was improved from 8-10 months to 13-15 months, and the risk of death was reduced by approximately 30%. Meanwhile, similar results have been achieved in the first-line treatment of advanced renal cancer with avelumab combined with axitinib (Javelin Renal 101)[9]. Based on results from the two studies, in 2019, the FDA approved the marketing applications of pembrolizumab combined with axitinib and avelumab combined with axitinib, ushering in the era of anti-PD-1 or PD-L1 antibodies combined with targeted anti-angiogenic therapy for advanced renal cancer. Encouraged by this, an increasing number of clinical trials of anti-PD-1 or PD-L1 antibodies combined with targeted anti-angiogenic therapy as the first-line treatment for advanced renal cancer are being actively carried out, and some of them have achieved good preliminary results, making it a clinical problem that needs to be solved urgently how to treat advanced renal cancer that has progressed after anti-PD-1 or PD-L1 antibody | |

| |
|---|
| Protocol Title: An Open-Label, Multicenter Phase II Clinical Study of Anti-PD-1 Antibody SHR-1210 Combined with Famitinib Malate in Patients with Advanced Urinary System Tumors and Gynecological Tumors |
| Protocol No., Version, and Date: SHR-1210-II-213, V4.0, 5 Nov., 2020 |
| Previous Version: V3.0, 8 Jul., 2020 |
| Revised Version: V4.0, 5 Nov., 2020 |

| Page | Content | Before | After | Reason for Revision |
|---|---|---|---|---|
| | | | therapy.<br>In order to address this problem, Merck and Eisai have taken the lead in exploring the preliminary efficacy of pembrolizumab combined with lenvatinib for the treatment of advanced renal cancer that had progressed after previous anti-PD-1 or PD-L1 antibody therapy (Keynote146)[10]. The study results showed that the ORR was as high as 59% for patients with advanced renal cancer that had progressed after previous treatment with anti-PD-1 or PD-L1 and targeted anti-angiogenic therapy. This suggested that advanced renal cancer that has progressed after previous treatment with anti-PD-1 or PD-L1 antibodies and targeted anti-angiogenic therapy can still benefit significantly from anti-PD-1 or PD-L1 antibodies combined with targeted anti-angiogenic therapy for the second time. | |
| P76 | Treatment period | ➢ Cohort 7:<br>For famitinib PK analysis, 3 mL of blood sample will be collected (only at sites with conditions for PK blood sampling and processing) at each of the following time points: at 6 (± 1) h post-administration on C1D1, within 30 min pre-administration and at 6 (± 1) h post-administration on | ➢ Cohorts 7 and 8:<br>For famitinib PK analysis, 3 mL of blood sample will be collected (only at sites with conditions for PK blood sampling and processing) at each of the following time points: at 6 (± 1) h post-administration on C1D1, within 30 min pre-administration and at 6 (± 1) h post-administration on C2D1 and C3D1, and the plasma will be separated. If famitinib administration | Revised based on design for the added Cohort 8. |

| |
|---|
| Protocol Title: An Open-Label, Multicenter Phase II Clinical Study of Anti-PD-1 Antibody SHR-1210 Combined with Famitinib Malate in Patients with Advanced Urinary System Tumors and Gynecological Tumors |
| Protocol No., Version, and Date: SHR-1210-II-213, V4.0, 5 Nov., 2020 |
| Previous Version: V3.0, 8 Jul., 2020 |
| Revised Version: V4.0, 5 Nov., 2020 |

| Page | Content | Before | After | Reason for Revision |
|---|---|---|---|---|
| | | C2D1 and C3D1, and the plasma will be separated. If famitinib administration is interrupted on the day of sampling, pre-administration sampling should be continued but post-administration sampling should be skipped. On the sampling day, it is also necessary to record the previous administration time. | is interrupted on the day of sampling, pre-administration sampling should be continued but post-administration sampling should be skipped. On the sampling day, it is also necessary to record the previous administration time. | |
| P67 | None | None | **5.6. Continuing Treatment After Progressive Disease**<br>**5.6.1. Criteria for continuing treatment**<br>Subjects in Cohort 6 (SHR-1210 monotherapy group) and Cohort 7 (famitinib monotherapy group) who can benefit from continuing treatment with the investigational products after PD (radiographic) as per the investigators' judgment may continue the combination therapy with the investigational products (SHR-1210 combined with famitinib) after discussion between the investigators and the sponsor and on the premise of fully informed consent of the subjects, until protocol-specified criteria for discontinuation are met. Subjects whose treatment is discontinued for non-PD reasons will directly proceed to the end-of-treatment visit and subsequent follow-ups. | Made revisions corresponding to the design for continuing treatment after PD in Cohorts 6 and 7. |

| |
|---|
| Protocol Title: An Open-Label, Multicenter Phase II Clinical Study of Anti-PD-1 Antibody SHR-1210 Combined with Famitinib Malate in Patients with Advanced Urinary System Tumors and Gynecological Tumors |
| Protocol No., Version, and Date: SHR-1210-II-213, V4.0, 5 Nov., 2020 |
| Previous Version: V3.0, 8 Jul., 2020 |
| Revised Version: V4.0, 5 Nov., 2020 |

| Page | Content | Before | After | Reason for Revision |
|---|---|---|---|---|
| | | | The visit cycle and efficacy evaluation of subjects who continue treatment after PD are the same as those before PD. AEs/SAEs and concomitant medications/treatments that occur during the period of continuing treatment after PD and within 30 days after the last dose should be recorded and reported in accordance with the time limit (dosing time limit) specified in the protocol.<br>Subjects in Cohorts 6 and 7 who choose to continue treatment after PD need to meet the following criteria:<br>– With radiographically confirmed PD as per RECIST v1.1;<br>– The investigators deem that it is in the best interest of the subject to receive SHR-1210 combined with famitinib, and the subject is not required to start other anti-tumor treatment immediately;<br>– Eligible for all inclusion criteria (except the requirement for the number of prior treatment lines) and none of the exclusion criteria after completing all examinations and assessments specified within 2 weeks before the first dose;<br>– The subject is able to tolerate continued study treatment;<br>– No significant clinical symptoms/signs of tumor progression; | |

| |
|---|
| Protocol Title: An Open-Label, Multicenter Phase II Clinical Study of Anti-PD-1 Antibody SHR-1210 Combined with Famitinib Malate in Patients with Advanced Urinary System Tumors and Gynecological Tumors |
| Protocol No., Version, and Date: SHR-1210-II-213, V4.0, 5 Nov., 2020 |
| Previous Version: V3.0, 8 Jul., 2020 |
| Revised Version: V4.0, 5 Nov., 2020 |

| Page | Content | Before | After | Reason for Revision |
|---|---|---|---|---|
| | | | – No rapid progression and no tumor progression involving vital organs/sites (e.g., spinal cord compression);<br>– Have completed imaging evaluation within 4 weeks before the first dose of SHR-1210 combined with famitinib;<br>– The subject must re-sign the ICF before continuing treatment.<br>**5.6.2. Additional considerations for continuing treatment**<br>The assessment of clinical benefit must consider whether the subject has clinical exacerbations and whether the subject can benefit from continuing treatment. It is recommended that the investigators should discuss with the sponsor whether the subject should continue treatment after PD.<br>The time window for continuing treatment is within 4 weeks after confirmed PD.<br>If it is decided that the subject will continue the study treatment after PD, the subject should continue to be treated, evaluated, and followed up according to the protocol requirements.<br>Subjects who continue treatment after PD must be fully informed and sign the ICF for continuing treatment after PD. | |

| |
|---|
| Protocol Title: An Open-Label, Multicenter Phase II Clinical Study of Anti-PD-1 Antibody SHR-1210 Combined with Famitinib Malate in Patients with Advanced Urinary System Tumors and Gynecological Tumors |
| Protocol No., Version, and Date: SHR-1210-II-213, V4.0, 5 Nov., 2020 |
| Previous Version: V3.0, 8 Jul., 2020 |
| Revised Version: V4.0, 5 Nov., 2020 |

| Page | Content | Before | After | Reason for Revision |
|---|---|---|---|---|
| | | | Continuing treatment will be given until confirmed PD and re-signing of the ICF for continuation is not allowed. | |

| |
|---|
| Protocol Title: An Open-Label, Multicenter Phase II Clinical Study of Anti-PD-1 Antibody SHR-1210 Combined with Famitinib Malate in Patients with Advanced Urinary System Tumors and Gynecological Tumors |
| Protocol No., Version, and Date: SHR-1210-II-213, V4.0, 5 Nov., 2020 |
| Previous Version: V3.0, 8 Jul., 2020 |
| Revised Version: V4.0, 5 Nov., 2020 |

| Page | Content | Before | After | Reason for Revision |
|---|---|---|---|---|
| P88 | Statistical hypothesis and discriminatory rules | Simon's two-stage minimax design will be adopted for each tumor type of Cohorts 6 and 7 in this study.<br>The discriminatory rules for each tumor type of Cohorts 6 and 7 are as follows: $n_1$ is the sample size of Stage I, n is the total sample size of the two stages, $r_1$ is the critical value of CR or PR that needs to be observed in Stage I (not inclusive), and r is the critical value of CR or PR that needs to be observed in the two stages (not inclusive). If, in Stage I, less than or equal to $r_1$ subjects among the $n_1$ subjects achieve CR or PR, the cohort will be terminated; otherwise n - $n_1$ subjects will be additionally enrolled in Stage II. If the number of subjects enrolled in Stage II does not equal to the planned number of enrolled subjects, the new critical value can be calculated using the Koyama and Chen's method, if necessary[9].<br>For each tumor type of Cohorts 6 and 7, when enrollment of Stage I is completed and the minimum number of subjects achieving CR and PR (confirmed or unconfirmed responses | Simon's two-stage minimax design will be adopted for each tumor type of Cohorts 6, 7, and 8 in this study.<br>The discriminatory rules for each tumor type of Cohorts 6, 7, and 8 are as follows: $n_1$ is the sample size of Stage I, n is the total sample size of the two stages, $r_1$ is the critical value of CR or PR that needs to be observed in Stage I (not inclusive), and r is the critical value of CR or PR that needs to be observed in the two stages (not inclusive). If, in Stage I, less than or equal to $r_1$ subjects among the $n_1$ subjects achieve CR or PR, the cohort will be terminated; otherwise n - $n_1$ subjects will be additionally enrolled in Stage II. If the number of subjects enrolled in Stage II does not equal to the planned number of enrolled subjects, the new critical value can be calculated using the Koyama and Chen's method[12], if necessary.<br>For each tumor type of Cohorts 6, 7, and 8, when enrollment of Stage I is completed and the minimum number of subjects achieving CR or PR (Cohorts 6 and 7: confirmed or unconfirmed responses $\geq 2$; Cohort 8: confirmed or unconfirmed responses $\geq 7$) is observed in a cohort, the Stage II enrollment of that cohort can be started. If the minimum number of | Added Cohort 8. |

| |
|---|
| Protocol Title: An Open-Label, Multicenter Phase II Clinical Study of Anti-PD-1 Antibody SHR-1210 Combined with Famitinib Malate in Patients with Advanced Urinary System Tumors and Gynecological Tumors |
| Protocol No., Version, and Date: SHR-1210-II-213, V4.0, 5 Nov., 2020 |
| Previous Version: V3.0, 8 Jul., 2020 |
| Revised Version: V4.0, 5 Nov., 2020 |

| Page | Content | Before | After | Reason for Revision |
|---|---|---|---|---|
| | | ≥ 2) is observed in a cohort, the Stage II enrollment of that cohort can be started. If the minimum number of subjects achieving CR or PR (confirmed or unconfirmed responses ≥ 2) has been observed before the enrollment of Stage I is completed, the enrollment of Stage II can be started immediately after the completion of the Stage I enrollment without being interrupted. If the minimum number of subjects achieving CR or PR (confirmed or unconfirmed responses ≤ 1) has not observed after the enrollment of Stage I is completed, enrollment must be interrupted until the conditions for continuing the enrollment are met. After completion of Stage II, if more than 5 subjects achieve CR or PR among a total of 23 subjects in the two stages, further clinical study is recommended. | subjects achieving CR or PR (Cohorts 6 and 7: confirmed or unconfirmed responses ≥ 2; Cohort 8: confirmed or unconfirmed responses ≥ 7) has been observed before the enrollment of Stage I is completed, the enrollment of Stage II can be started immediately after the completion of the Stage I enrollment without being interrupted. If the minimum number of subjects achieving CR or PR has not observed after the enrollment of Stage I is completed (Cohorts 6 and 7: confirmed or unconfirmed responses ≤ 1; Cohort 8: confirmed or unconfirmed responses ≤ 6), enrollment must be interrupted until the conditions for continuing the enrollment are met. After completion of Stage II, if, for Cohort 6 or 7, more than or equal to 5 subjects achieve CR or PR among a total of 23 subjects in the two stages, and if, for Cohort 8, more than or equal to 21 subjects achieve CR or PR among a total of 47 subjects in the two stages, further clinical study is recommended. | |
| P14, P93 | Sample size | None | The Simon's (minimax) two-stage design is adopted to calculate the sample size of Cohort 8 based on one-sided alpha = 0.025 and power = 0.8: | Added Cohort 8. |

| |
|---|
| Protocol Title: An Open-Label, Multicenter Phase II Clinical Study of Anti-PD-1 Antibody SHR-1210 Combined with Famitinib Malate in Patients with Advanced Urinary System Tumors and Gynecological Tumors |
| Protocol No., Version, and Date: SHR-1210-II-213, V4.0, 5 Nov., 2020 |
| Previous Version: V3.0, 8 Jul., 2020 |
| Revised Version: V4.0, 5 Nov., 2020 |

<table>
<tr><th>Page</th><th>Content</th><th>Before</th><th>After</th><th>Reason for Revision</th></tr>
<tr><td></td><td></td><td></td><td>Table 1~~1~~. Cohorts 8: Calculation of sample size by Simon's (minimax) two-stage method.
<table>
<tr><td>Cohort and Tumor Type</td><td>$P_0$</td><td>$P_1$</td><td>Stage I ($r_1/n_1$)</td><td>Stage II (r/n)</td></tr>
<tr><td>Cohort 8 Renal Cell Carcinoma</td><td>0.30</td><td>0.50</td><td>6/21</td><td>20/47</td></tr>
</table>
Note: $P_0$ is the maximum futility boundary, and $P_1$ is the minimum efficacy boundary. $n_1$ is the sample size of Stage I, n is the total sample size of the two stages, $r_1$ is the critical value of CR or PR that needs to be observed in Stage I (not inclusive), and r is the critical value of CR or PR that needs to be observed in the two stages (not inclusive). If, in Stage I, equal to $r_1$ subjects among the $n_1$ subjects achieve CR or PR, the cohort will be terminated; otherwise n - $n_1$ subjects will be additionally enrolled in Stage II.</td><td></td></tr>
<tr><td>P46</td><td>2.3. Acquisition, Collection, and Processing of Biomarkers</td><td>1) Existing paraffin-embedded tumor tissue sections should be collected and 5 tumor sections with a thickness of 4-5 μm should be used for PD-L1 detection.<br>2) It is recommended that biopsy samples (core needle biopsy) be collected before the first dose. After fixation and embedding,</td><td>1) Existing paraffin-embedded tumor tissue sections will be collected, and 5 tumor sections with a thickness of 4-5 μm will be used for PD-L1 detection (except for Cohort 7).<br>2) It is recommended that biopsy samples (core needle biopsy) be collected before the first dose. After fixation and embedding, 3 tumor sections with a thickness of 4-5 μm will be prepared for PD-L1 detection (except for Cohort 7);</td><td>Revised logic.</td></tr>
</table>

| |
|---|
| Protocol Title: An Open-Label, Multicenter Phase II Clinical Study of Anti-PD-1 Antibody SHR-1210 Combined with Famitinib Malate in Patients with Advanced Urinary System Tumors and Gynecological Tumors |
| Protocol No., Version, and Date: SHR-1210-II-213, V4.0, 5 Nov., 2020 |
| Previous Version: V3.0, 8 Jul., 2020 |
| Revised Version: V4.0, 5 Nov., 2020 |

| Page | Content | Before | After | Reason for Revision |
|---|---|---|---|---|
| | | 3 tumor sections with a thickness of 4-5 μm will be prepared for PD-L1 detection; | | |
| P73 | Criteria for efficacy evaluation | If there is PD as per RECIST v1.1, an imaging examination is required for confirmation 4-6 weeks later (except those with rapid progression or significant clinical progression); | If there is PD as per RECIST v1.1, an imaging examination is required for confirmation 4-6 weeks later (except those with rapid progression, with significant clinical progression, or receiving famitinib monotherapy); | Revised logic. |
