# Supplementary material for: A multicenter phase 2 trial of camrelizumab plus famitinib for women with recurrent or metastatic cervical squamous cell carcinoma
Source: Nat Commun. 2022 Dec 8;13:7581. doi: 10.1038/s41467-022-35133-4 (PMC9732039; doi:10.1038/s41467-022-35133-4)
Supplement: Supplementary file 2 — Reporting Summary [file 41467_2022_35133_MOESM2_ESM.pdf]

nature portfolio

Corresponding author(s): Xiaohua Wu

Last updated by author(s): Oct 28, 2022

Reporting Summary

Nature Portfolio wishes to improve the reproducibility of the work that we publish. This form provides structure for consistency and transparency in reporting. For further information on Nature Portfolio policies, see our Editorial Policies and the Editorial Policy Checklist.

Statistics

For all statistical analyses, confirm that the following items are present in the figure legend, table legend, main text, or Methods section.

n/a	Confirmed	
☐	☒	The exact sample size (n) for each experimental group/condition, given as a discrete number and unit of measurement
☐	☒	A statement on whether measurements were taken from distinct samples or whether the same sample was measured repeatedly
☒	☐	The statistical test(s) used AND whether they are one- or two-sided<br>*Only common tests should be described solely by name; describe more complex techniques in the Methods section.*
☐	☒	A description of all covariates tested
☐	☒	A description of any assumptions or corrections, such as tests of normality and adjustment for multiple comparisons
☐	☒	A full description of the statistical parameters including central tendency (e.g. means) or other basic estimates (e.g. regression coefficient) AND variation (e.g. standard deviation) or associated estimates of uncertainty (e.g. confidence intervals)
☐	☒	For null hypothesis testing, the test statistic (e.g. F, t, r) with confidence intervals, effect sizes, degrees of freedom and P value noted<br>*Give P values as exact values whenever suitable.*
☒	☐	For Bayesian analysis, information on the choice of priors and Markov chain Monte Carlo settings
☒	☐	For hierarchical and complex designs, identification of the appropriate level for tests and full reporting of outcomes
☐	☒	Estimates of effect sizes (e.g. Cohen's d, Pearson's r), indicating how they were calculated

Our web collection on statistics for biologists contains articles on many of the points above.

Software and code

Policy information about availability of computer code

Data collection	Data were collected using HRTAU EDC (version 2.8.4).
Data analysis	All statistical analyses were performed using SAS (version 9.4 or higher) software.

For manuscripts utilizing custom algorithms or software that are central to the research but not yet described in published literature, software must be made available to editors and reviewers. We strongly encourage code deposition in a community repository (e.g. GitHub). See the Nature Portfolio guidelines for submitting code & software for further information.

Data

Policy information about availability of data

All manuscripts must include a data availability statement. This statement should provide the following information, where applicable:

- Accession codes, unique identifiers, or web links for publicly available datasets
- A description of any restrictions on data availability
- For clinical datasets or third party data, please ensure that the statement adheres to our policy

Individual de-identified participant data that underlie the results reported in this article is subject to controlled access. Data may be requested after the product and indication has been approved by major health authorities and 24 months after completion of all the arms of this study. Qualified researchers should submit a proposal to the corresponding author (wu.xh@fudan.edu.cn) outlining the reasons for requiring the data. The leading clinical site and sponsor will check whether the request is subject to any intellectual property or confidentiality obligations. Use of data must also comply with the requirements of Human Genetics Resources Administration of China. A signed data access agreement with the sponsor is required before accessing shared data. The study protocol is provided with the paper.

Human research participants

Policy information about studies involving human research participants and Sex and Gender in Research.

Reporting on sex and gender	This study was conducted in women with pretreated recurrent or metastatic cervical cancer. Patients who had histologically or cytologically confirmed cervical squamous cell carcinoma were enrolled. Therefore, sex- and gender-based analyses was not applicable.
Population characteristics	Eligible patients were aged between 18 and 75 years; had histologically or cytologically confirmed cervical squamous cell carcinoma; had experienced relapse or progression during or after 1-2 lines of systemic therapy for recurrent or metastatic disease (excluding radiotherapy sensitized chemotherapy); had at least one measurable lesion according to the Response Evaluation Criteria in Solid Tumors (RECIST) version 1.1; had an Eastern Cooperative Oncology Group performance status of 0 or 1; had a life expectancy of at least 12 weeks; and had adequate hematological, hepatic, and renal function. If the disease relapsed and progressed within 1 year after standard surgery or 6 months after radiotherapy, neoadjuvant or adjuvant therapy (excluding radiotherapy sensitized chemotherapy) was considered as first-line systemic treatment. A key protocol amendment was made based on the following update: "relapsed or progressed during or after at least 1 line of systemic therapy for recurrent or metastatic disease" (November 30, 2018; version 1.1) to "relapsed or progressed during or after 1-2 lines of systemic therapy for recurrent or metastatic disease" (amendment date August 06, 2019; version 2.0).<br>The key exclusion criteria were as follows: any active autoimmune disease; history of autoimmune disease, immunosuppressive medication intake, or systemic corticosteroid administration within 2 weeks before study drug administration; history of untreated central nervous system metastases, coagulation abnormalities, bleeding event of ≥ grade 2 according to the Common Terminology Criteria for Adverse Events (CTCAE) version 4.03 within 4 weeks before study drug administration; history of treatment with PD-L1 or PD-1 antagonists or famitinib; and known additional malignancies within the last 5 years.
Recruitment	In this study, patients were consecutively recruited from 7 clinical centers using the protocol-prespecified eligibility criteria. As this is an open-label, single-arm trial, neither randomization nor blinding is involved in this study. Therefore, self-selection bias was minimized. Another possible bias may be caused by the fact that patients come from study centers with different economic levels, which leads to differences in the support treatments allowed by the study protocol.
Ethics oversight	The study was conducted in accordance with the Declaration of Helsinki and Good Clinical Practice guidelines. The protocol and all amendments were approved by the Ethics Committee of each study center (see Supplementary Information for the complete list of centers). All patients provided written informed consent.

Note that full information on the approval of the study protocol must also be provided in the manuscript.

Field-specific reporting

Please select the one below that is the best fit for your research. If you are not sure, read the appropriate sections before making your selection.

☒ Life sciences ☐ Behavioural & social sciences ☐ Ecological, evolutionary & environmental sciences

For a reference copy of the document with all sections, see nature.com/documents/nr-reporting-summary-flat.pdf

Life sciences study design

All studies must disclose on these points even when the disclosure is negative.

Sample size	An adaptive two-stage design according to Lin's study (Lin, Y. et al. Biometrics. 2004;60(2):482-90) was adopted for patient enrollment. An objective response rate of 15% was assumed as ineffective, 25% was assumed to represent a low response rate, and 35% was assumed to represent a high response rate. Planned sample sizes of 53 and 33 patients were estimated to provide 70% and 80% power for demonstrating low and high response rates, respectively, with a two-sided α level of 0.1.

	In Lin's study (Lin, Y. et al. Biometrics. 2004;60(2):482-90), a feasible solution for sample size in stage 1 and stage 2 was used, which satisfied the error constraint, and there are four optimality criteria for the feasible solution mentioned. The final selected total sample size in the study is the smallest of four optimality criteria based on the maximum total sample size between low response rate and high response rate and the expected sample size when ineffective response rate is true. In stage 1, 22 patients were enrolled based on recommendation from Lin's study (Lin, Y. et al. Biometrics. 2004;60(2):482-90) according to the smallest selected sample size of 53. Recruitment of stage 2 was planned as follows: if there were ≤2 responders in stage 1, further recruitment would be terminated; in the event of 3-6 responders, an additional 31 patients would be recruited at stage 2 to achieve a total of 53 patients. In the event of ≥7 responders, an additional 11 patients would be recruited at stage 2 to achieve a total of 33 patients.
Data exclusions	Efficacy was assessed in the full-analysis set, which included all patients who received at least one dose of the study drugs. The safety analysis set included all patients who received at least one dose of the study drugs and had at least one post-baseline safety assessment. No data were excluded from the analyses. All the 33 patients we enrolled were included in the efficacy analysis and safety analysis.
Replication	Our study is a phase 2 clinical trial, and the data we presented in this manuscript are clinical results of antitumor efficacy and safety of our study drug. Therefore, the experimental replication which is required in studies containing laboratory experiments is not applicable to this clinical trial.
Randomization	This is a single-arm study, patients were recruited from 7 sites using eligibility criteria pre-specified in the study protocol. Randomization is not relevant to this study.
Blinding	This is a single-arm study, so blinding is not relevant to this study.

Reporting for specific materials, systems and methods

We require information from authors about some types of materials, experimental systems and methods used in many studies. Here, indicate whether each material, system or method listed is relevant to your study. If you are not sure if a list item applies to your research, read the appropriate section before selecting a response.

Materials & experimental systems

n/a	Involved in the study	
☒	☐	Antibodies
☒	☐	Eukaryotic cell lines
☒	☐	Palaeontology and archaeology
☒	☐	Animals and other organisms
☐	☒	Clinical data
☒	☐	Dual use research of concern

Methods

n/a	Involved in the study	
☒	☐	ChIP-seq
☒	☐	Flow cytometry
☒	☐	MRI-based neuroimaging

Clinical data

Policy information about clinical studies

All manuscripts should comply with the ICMJEguidelines for publication of clinical research and a completedCONSORT checklist must be included with all submissions.

Clinical trial registration	ClinicalTrials.gov, number NCT03827837
Study protocol	Submitted with manuscript
Data collection	Thirty-three patients with recurrent or metastatic cervical squamous cell carcinomas were enrolled from 7 hospitals in China (see Supplementary Information for the complete list of centers) between April 04, 2019 and June 08, 2020. Data cutoff date of the analysis was June 08, 2021. All efficacy and safety data were collected from the 7 participating clinical centers.
Outcomes	The primary endpoint was the objective response rate, defined as the proportion of patients with a best overall response of complete or partial response. Secondary endpoints included duration of response (the time from the first complete or partial response to death or progression, whichever occurred first), disease control rate (the proportion of patients with a best overall response of complete response, partial response, or stable disease), time to response (duration between the first dose and first documented tumor response), progression-free survival (duration between the first dose and the first documented radiographic progression or death from any cause), overall survival (duration between the first dose to death from any cause), probability of 12-month survival, and safety.<br>Tumor responses were assessed by the investigator at baseline and every 3 cycles (9 weeks) after the initiation of treatment using computed tomography or magnetic resonance imaging according to RECIST version 1.1. Complete and partial responses were confirmed by subsequent repeat imaging at least 4 weeks after initial response assessment. Disease progression had to be confirmed by imaging examination 4-6 weeks later, and survival was assessed every two months until death.<br>Safety evaluation included assessment for adverse events, vital signs, 12-lead electrocardiograms, and laboratory tests, which were evaluated on days 1 and 7 of the first cycle and on day 1 of every 3-week cycle thereafter. Adverse events were monitored throughout treatment and for up to 30 days after the last dose, and graded according to the CTCAE version 4.03. Only treatment-related serious adverse events were recorded in cases where subsequent anti-cancer treatment was initiated.
